# Synthesis and characterization of chlorotriarylbismuthonium salts†

**DOI:** 10.1039/d4cc03364g

**Published:** 2024-08-27

**Authors:** Jennifer Kuziola, Nils Nöthling, Markus Leutzsch, Josep Cornella

**Affiliations:** a Max-Planck-Institut für Kohlenforschung, Kaiser-Wilhelm-Platz 1 45470 Mülheim an der Ruhr Germany cornella@kofo.mpg.de

## Abstract

This work reports the synthesis and structural study of a family of chlorotriarylbismuthonium salts. The abstraction of a chlorine atom with NaBAr^F^ from triarylbismuth dichloride species leads to monomeric and dimeric chlorotriarylbismuthonium species, which show a distinct behavior in solution and solid-state in comparison to their fluorotriarylbismuthonium analogues.

## Abstract 2

This work fills the synthetic gap in the series of chlorotriarylpnictonium ions, by providing the heavier analogue based on bismuth.

## Graphical abstract

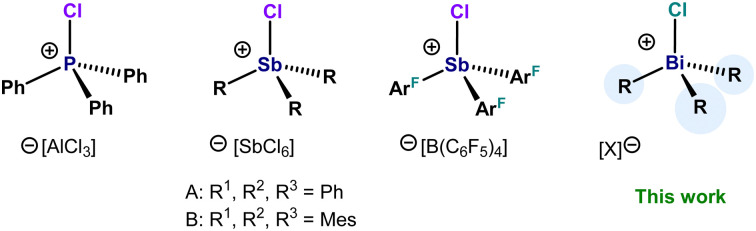

The synthesis and design of novel Lewis acids based on cheap and more abundant main group elements opens the door to sustainable catalysts that can have an impact in organic synthesis and applications thereof. Of particular importance, are those derived from organopnictonium ions. In this regard, numerous fluorophosphonium salts have been synthesized, demonstrating their Lewis acidity in a wide range of organic transformations (Fig. 1A).^1–10^ The heightened Lewis acidity observed in cationic haloorganopnictonium salts is ascribed to the low-lying σ*-orbital positioned *trans* to the halogen atom.^11–13^ In contrast to its lighter congeners, heavier analogues in the group 15 have received comparable less attention despite their well-documented electrophilicity.^14–17^ Remarkable examples in this front are the monomeric fluorotriarylstibonium ions developed by Gabbaï and co-workers where interaction between the cationic Sb(v) and a OTf^−^ anion can be observed in the solid state (Fig. 1A).^12^ Based on our interest in the study of organometallic compounds of bismuth,^18–20^ our group has recently reported a structural study on a series of fluorotriarylbismuthonium salts,^21^ which have shown a distinctive behaviour in comparison with its lighter analogues. In contrast to the monomeric cationic salts based on P and Sb,^12^ we observed the formation of di- and trinuclear fluorobismuthonium compounds. This distinct behaviour was attributed to the highly electropositive Bi(v) center in combination with the strong donor–acceptor interactions between the fluorine and Bi atom. Evaluation of the steric constraints on the aryl moieties eventually led to the isolation of the first monomeric fluorotriarylbismuthonium salt (Fig. 1A). Following this rationale, we realized that in contrast to fluoropnictonium salts, few examples of chlorotriarylpnictonium salts have been reported,^12,17,22–28^ (Fig. 1B). Gabbaï demonstrated a remarkable example of a monomeric chlorotrimesitylstibonium hexachloroantimonate salt, and its application in the polymerization of THF and dimerization of 1,1-diphenylethylene (Fig. 1B).^12^ Recently, Benjamin *et al.* enhanced the Lewis acidity by the introduction of electron-withdrawing aryl ligands, showing also the potential for formation of C–C bonds in a Friedel–Crafts alkylation reactions (Fig. 1B).^28^ Moreover, the same group has demonstrated the formation of a dinuclear chlorotriarylstibonium salt by the use of 0.4 equiv. of [Et_3_Si(C_7_H_8_)][B(C_6_F_5_)_4_] as chloride abstracting agent. Based on our previous work,^21^ we present the synthesis and structural study of chlorotriarylbismuthonium salts, including the first example of a monomeric chlorotriarylbismuthonium salt (Fig. 1C).

**Fig. 1:**
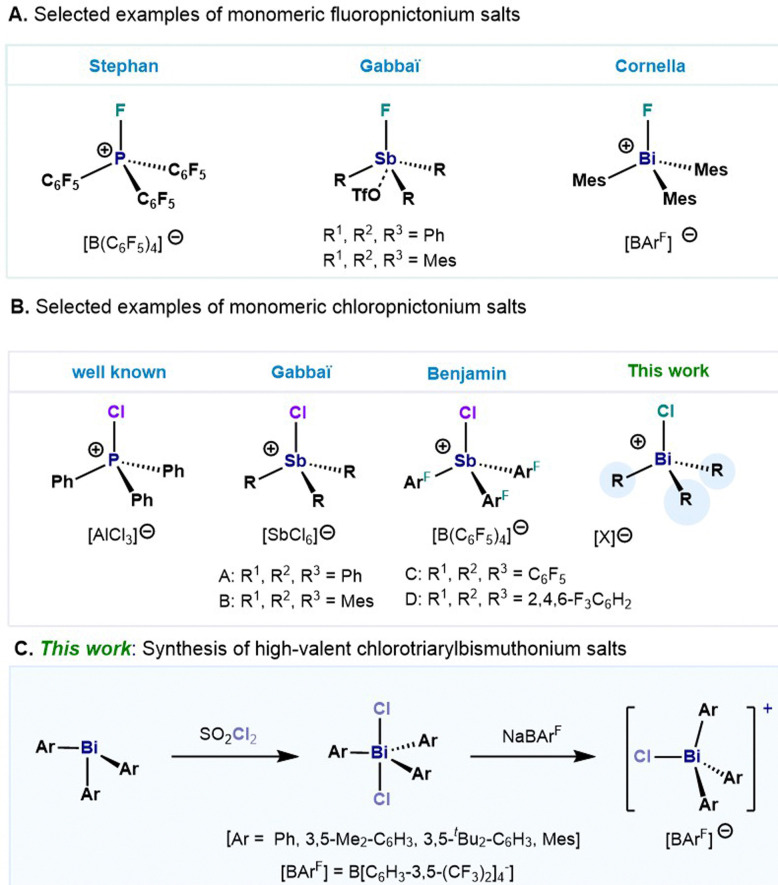
(A) Representative examples of mononuclear fluorotriarylpnictonium salts. (B) Examples of mononuclearchlorotriarylpnictonium salts. (C) This work: synthesis of high-valent chlorotriarylbismuthonium salts.

Our studies of chlorotriarylbismuthonium salts started with the synthesis of triarylbismuth dichlorides. In order to be able to compare the chlorobismuthonium cations with the fluorinated analogues, unsubstituted and substituted aryl ligands bearing ^*t*^Bu and Me groups have been selected. Oxidation of triarylbismuth complexes with 1.3 equiv. of SO_2_Cl_2_ led to the isolation of the corresponding triarylbismuth dichlorides in high yields, which have been characterized by NMR, HRMS and SC-XRD (single crystal X-ray diffraction) (see ESI† for more details) (Scheme 1). Treating Ph_3_BiCl_2_ with 1.0 equiv. of NaBAr^F^ (BAr^F^ = B[C_6_H_3_-3,5-(CF_3_)_2_]_4_^−^) in CH_2_Cl_2_ at 25 °C indicated the presence of a formally monomeric [Ph_3_BiCl][BAr^F^] (9) species in solution as suggested by the integration of the phenyl signals with respect to the BAr^F^ anion in the ^1^H NMR spectrum.

**Scheme 1:**
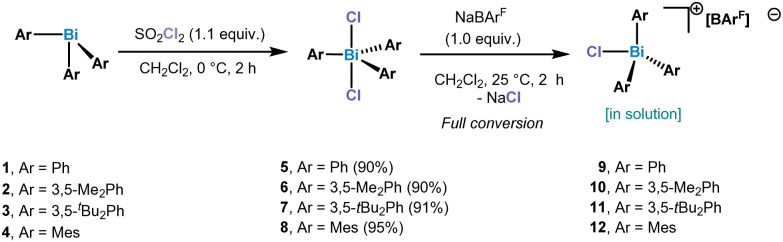
Synthesis of triarylbismuth dichlorides 5–8 and chlorotriaryl bismuthonium salts.

Crystals of compound 9 suitable for SC-XRD analysis could not be obtained and therefore its structure in the solid state remains elusive. However, when Ph_3_BiCl_2_ was treated with 0.5 equiv. of NaBAr^F^ in CH_2_Cl_2_ at 25 °C, the formation of a dinuclear chlorotriphenylbismuthonium salt [(Ph_3_BiCl)_2_Cl][BAr^F^] (9a) was observed in solution and solid-state as analyzed by NMR spectroscopy and SC-XRD (Fig. 2). The different formation of 9 and 9a in solution is in stark contrast to our previous observations with the parent difluorotriphenylbismuth, where the amount of NaBAr^F^ did not affect the reactivity of the halogen abstraction step.^21^ The formation of 9a resembles rather its lighter element homologue, the chlorotriphenylstibonium cation, which was obtained after treating Ph_3_SbCl_2_ with 0.4 equiv. of [Et_3_Si(C_7_H_8_)] [B(C_6_F_5_)_4_].^28^ Solid-state analysis of 9a reveals no additional intermolecular Bi⋯Cl interactions between the individual units. As illustrated in Fig. 2, both Bi centres adopt a trigonal bipyramidal geometry with chlorine ligands in apical and the phenyl groups in equatorial positions. The chlorobismuthonium cation (9a) exhibits a bent Bi1–Cl3–Bi2 geometry (Bi1–Cl3–Bi2: 128.10(2)°), attributed to packing effects during crystallization and the larger atomic size of chlorine compared to fluorine. Whereas the terminal Bi1–Cl1 and Bi2–Cl2 bond lengths were determined to be 2.4856(7) Å and 2.4996(8) Å, the distances between the bridging chloride and the Bi centres are characteristically longer (Bi1–Cl3: 2.7995(7), Bi2–Cl3: 2.8274(7) Å).^21^

**Fig. 2:**
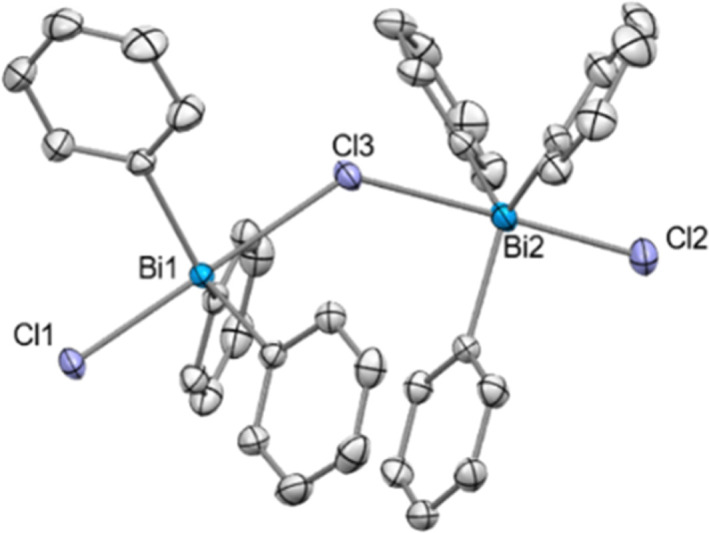
Solid state structure of 9a.^29^ Ellipsoids are drawn at the 50% probability level. H atoms, disordered parts and BAr^F^ are omitted for clarity. Selected bond lengths (Å) and angles (°): Bi1–Cl1: 2.4856(7), Bi1–Cl3: 2.7995(7), Bi2–Cl2: 2.4996(8), Bi2–Cl3: 2.8274(7), Bi1–Cl3–Bi2: 128.10(2); Cl1–Bi1–Cl3: 179.25(2), Cl2–Bi2–Cl3: 177.70(2).

The addition of 1.0 equiv. NaBAr^F^ to (*m*-Xyl)_3_BiCl_2_ (6) (*m*-Xyl = *meta*-xylene) in CH_2_Cl_2_ for 2 h at 25 °C resulted in the formation of mononuclear [(*m*-Xyl)_3_BiCl][BAr^F^] (10) in solution, as judged by the integration in the ^1^H NMR spectrum. Crystals suitable for X-ray diffraction confirmed the isolation of a mononuclear bismuthonium salt as depicted in Fig. 3. The solid state structure reveals that the Bi atoms adopts a distorted tetrahedral geometry (*τ*_4_ = 0.89).^30^ The Bi–Cl bond length of 2.4028(8) Å is slightly shorter in complex 10 compared to the terminal Bi–Cl length of complex 9a. Interestingly, the presence of the less electronegative chlorine atom prevents dimerization or trimerization processes, and stands in contrast to the observations for its fluorinated analogues, where sterics controlled these processes.^21^

**Fig. 3:**
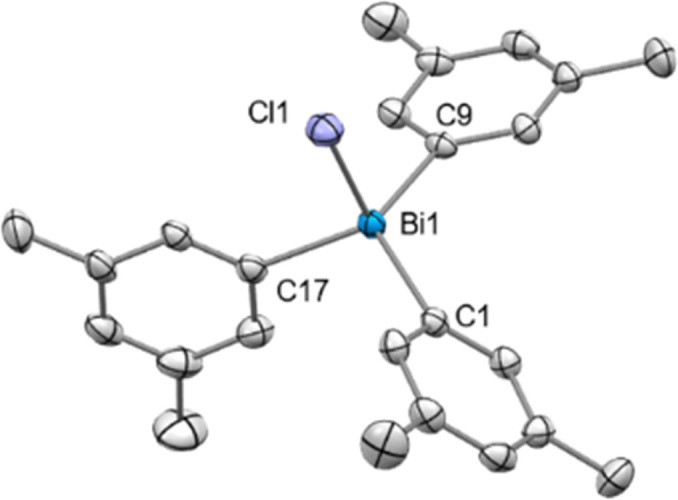
Solid state structure of 10. Ellipsoids are drawn at the 50% probability level. H atoms and BAr^F^ are omitted for clarity. Selected bond lengths (Å) and angles (°): Bi1–Cl1: 2.4028(8), Bi1–C1: 2.185(3), Bi1–C9: 2.180(3), Bi1–C17: 2.189(3), C1–Bi1–C9: 121.63(11), C1–Bi2–C17: 111.32(10), C9–Bi2–C17: 112.55(11).

After treating 6 with 0.5 equiv. of NaBAr^F^ a new set of ^1^H NMR signals was observed that is in line with a formally formation of a dinuclear bismuthonium [((*m*-Xyl)_3_BiCl)_2_Cl][BAr^F^] species (10a) in solution as judged by ^1^H NMR. However, contrary to our expectations, crystallization of this compound led to an SC-XRD of (*m*-Xyl)_3_BiCl_2_ (6). We hypothesize that the chloride abstraction with 0.5 equiv. NaBAr^F^ leads to the formation of a monomeric chlorobismuthonium cation 10, which reacts with a neutral triarylbismuth dichloride (6) resulting in a reversible equilibrium with dinuclear chlorobismuthonium salt (10a) in solution (Scheme 2). Further crystallization leads to the precipitation of complex 6. A similar equilibrium was observed with the trimeric fluorobismuthonium salt bearing phenyl as ligands using 1.0 equiv. of BAr^F^.^21^

**Scheme 2:**
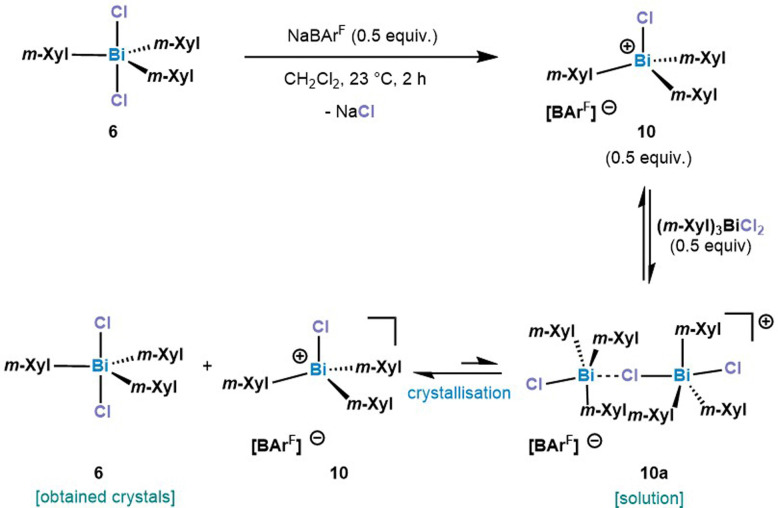
Proposed behaviour of 10a in solution and solid state. *m*-Xyl = 3,5-Me_2_-C_6_H_3_.

Increasing the steric bulk by replacing the Me groups by ^*t*^Bu, a different behaviour in the formation of the bismuthonium salt could be observed. The addition of 1.0 equiv. of NaBAr^F^ to complex 7 leads to a new set of ^1^H NMR signals that are in line with a formally monomeric chlorotriarylbismuthonium salt [^*t*Bu^Ar_3_BiCl][BAr^F^] (11). However, crystallization of complex 11 revealed the formation of a dimeric chlorobismuthonium salt (11a) as illustrated in Fig. 4, resembling the product and structure of reported fluorine analogue.^21^ The solid-state structure of 11 reveals that both Bi atoms adopt a trigonal bipyramidal geometry with two chloride ligands in apical positions and aromatic rings in equatorial positions (Fig. 4A). Both Bi atoms are united by a bridging chloride ligand, generating a Bi1–Cl2–Bi1 angle of 180.0°. Whereas the terminal Bi1–Cl1 bond lengths are shorter compared to the neutral parent complex 7 [11, Bi1–Cl1: 2.4857(7) Å; 7, Bi1–Cl1: 2.5954(5) Å] (see ESI† for more details), the bond length between the bridging chlorine and both Bi atoms are increased to 2.80068(13) Å. While the Bi1–Cl2–Bi1 axis displays an angle of 180.0°, the angle of a single ^*t*Bu^Ar_3_BiCl_2_ unit is slightly deviated from linearity [Cl1–Bi1–Cl2: 173.722(17)°]. Moreover, the presence of the chlorine atoms in the bismuthonium salt 11 allows the aromatic ligands a greater flexibility due to the longer Bi1–Cl2 bond lengths [Bi1–Cl2: 2.80068(13) Å] compared to the fluorinated analogue [Bi1–F1: 2.282(3) Å],^21^ leading to a propeller-like conformation of the aromatic ligands (Fig. 4B). The addition of 0.5 equiv. of NaBAr^F^ to 7 resulted in a dimeric bismuthonium salt 11a [((*m*-Xyl)_3_BiCl)_2_Cl][BAr^F^] in solution and solid state (see ESI† for more details).

**Fig. 4:**
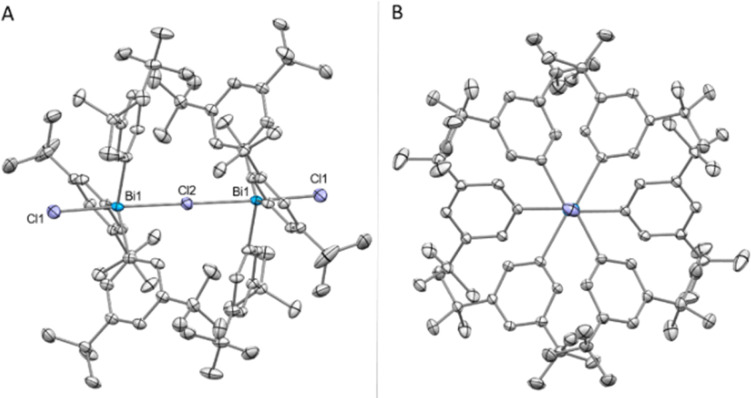
(A) Solid state structure of 11a.^29^ Ellipsoids are drawn at the 50% probability level. H atoms and BAr^F^ are omitted for clarity. Selected bond lengths (Å) and angles (°): Bi1–Cl1: 2.4857(7), Bi1–Cl2: 2.80068(13), Bi1–Cl2–Bi1: 180.0, Cl1–Bi1–Cl2: 173.722(17). (B) View along the Cl–Bi–Cl–Bi–Cl axis.

When comparing the formation of complexes 10 and 11a in the solid state, it can be observed that the *meta tert*-butyl groups might exert an attractive effect as a consequence of the London-dispersion forces. Although the *m*-^*t*^Bu substituents are more sterically demanding in comparison to the *m*-Me groups, they serve as dispersion energy donors,^31^ leading to the formation of a thermodynamically more stable dimeric species 11a in solid state. The influence on the stability by *m*-^*t*^Bu groups through attractive dispersion interactions has been previously observed in the literature.^31^

Increasing the sterics at the *ortho*-position by adding Me substituents, resulted in the formation of a monomeric chlorotrimesitylbismuthonium salt 12 in solution. Despite several crystallization attempts, suitable crystals could not be obtained. In order to get more insight into the potential structure, we reacted 12 with 1.0 equiv. of pyridine-*N*-oxide for 2 h at 25 °C in CH_2_Cl_2_. This reaction led to the isolation of complex 13 which was confirmed by NMR and SC-XRD (Scheme 3).

**Scheme 3:**
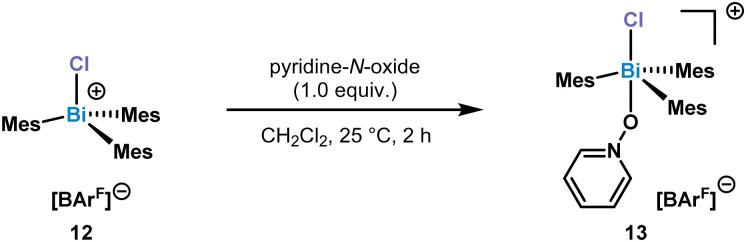
Synthesis of complex 13.

A broadening of the ^13^C NMR signals of the pyridine ring and the ^15^N NMR shift (*δ* = −108.0 ppm) indicated a weak Bi–O bond which was also supported by the results of the HRMS data, as only the chlorobismuthonium salt has been detected. The solid-state structure of 13 displays a coordination of the pyridine-*N*-oxide *via* the O atom to the Bi centre, displaying a Bi–O bond length of 2.404(6) Å (Fig. 5). Moreover, the Bi center adopts a trigonal bipyramidal geometry with the chlorine and oxygen atom in apical positions as well as Mes ligands in equatorial positions. Complex 13 exhibits a Cl–Bi–O angle of 173.5(2)°.

**Fig. 5:**
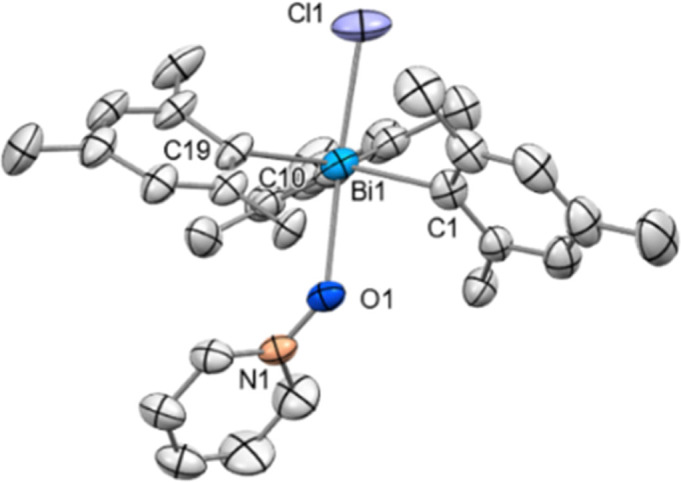
Solid state structure of 13. Ellipsoids are drawn at the 50% probability level. H atoms and BAr^F^ are omitted for clarity. Selected bond lengths (Å) and angles (°): Bi1–Cl1: 2.507(7), Bi1–O1: 2.404(6), Bi1–C1: 2.229(6), Bi1−C10: 2.226(5), Bi1–C19: 2.236(6), N1–O1: 1.334(8), C1–Bi1–C10: 117.9(2), C1–Bi1–C19: 121.7(2), C10–Bi1–C19: 119.3(2), Cl1–Bi1–O1: 173.5(2), N1–O1–Bi: 136.7(4).

In conclusion, we have reported a series of chlorotriarylbismuthonium salts with BAr^F^ as a weakly coordinating anion and extended the library of halotriarylbismuthonium salts. Contrary to our previous work on fluorobismuthonium salts, where the formation of either mono-, di- and trinuclear formation were controlled by steric factors, the use of a chloride ligand has displayed a distinctive behaviour, leading to fast dynamic exchanges in solution. The less electronegative chlorine ligands generate more labile bismuthonium units, allowing easier access to monomeric halobismuthonium salts. However, London dispersion forces assist in the formation of dimeric species in solid-state.

Financial support for this work was provided by Max-Planck-Gesellschaft, Max-Planck-Institut für Kohlenforschung, and the Deutsche Forschungsgemeinschaft (DFG, German Research Foundation) under Germany's Excellence Strategy - EXC 2033 - 390677874 - RESOLV. This project has received funding from European Union's Horizon 2020 research and innovation programme under Agreement No. 850496 (ERC Starting Grant, J.C.). We thank MS, GC and X-ray departments of Max-Planck-Institut für Kohlenforschung for analytic support. We thank Prof. Dr A. Fürstner for insightful discussions and generous support. Open Access funding provided by the Max Planck Society.

## Data availability

The data supporting this article have been included as part of the ESI.† Crystallographic data for 6-8, 9a-11 and 13 have been deposited at the CCDC under CCDC numbers 2356069–2356071 (6, 10, 13) and 2289340 (7), 2296787 (8), 2289343 (9a), 2296786 (11a).

## Conflicts of interest

There are no conflicts to declare.

## Supplementary Material

CC-060-D4CC03364G-s001

CC-060-D4CC03364G-s002

## References

[cit1] Caputo C. B., Hounjet L. J., Dobrovetsky R., Stephan D. W. (2013). Science.

[cit2] Pérez M., Hounjet L. J., Caputo C. B., Dobrovetsky R., Stephan D. W. (2013). J. Am. Chem. Soc..

[cit3] Caputo C. B., Winkelhaus D., Dobrovetsky R., Hounjet L. J., Stephan D. W. (2015). Dalton Trans..

[cit4] Mehta M., Holthausen M. H., Mallov I., Pérez M., Qu Z.-W., Grimme S., Stephan D. W. (2015). Angew. Chem., Int. Ed..

[cit5] Pérez M., Mahdi T., Hounjet L. J., Stephan D. W. (2015). Chem. Commun..

[cit6] Holthausen M. H., Mehta M., Stephan D. W. (2014). Angew. Chem., Int. Ed..

[cit7] Mehta M., Garcia de la Arada I., Perez M., Porwal D., Oestreich M., Stephan D. W. (2016). Organometallics.

[cit8] Mallov I., Stephan D. W. (2016). Dalton Trans..

[cit9] Postle S., Podgorny V., Stephan D. W. (2016). Dalton Trans..

[cit10] Vogler M., Süsse L., LaFortune J. H. W., Stephan D. W., Oestreich M. (2018). Organometallics.

[cit11] Benz S., Poblador-Bahamonde A. I., Low-Ders N., Matile S. (2018). Angew. Chem. Int. Ed..

[cit12] Yang M., Gabbaï F. P. (2017). Inorg. Chem..

[cit13] Thorwart T. and Greb L., in Encyclopedia of Inorganic and Bioinorganic Chemistry, ed. R. A. Scott, Wiley, 2021, pp. 1–26

[cit14] Murray J. S., Lane P., Politzer P. A. (2007). Int. J. Quantum Chem..

[cit15] Bauzá A., Mooibroek T. J., Frontera A. (2015). Chem. Phys. Chem..

[cit16] Murray J. S., Lane P., Clark T., Riley K. E., Politzer P. (2012). J. Mol. Model..

[cit17] García-Monforte M. A., Baya M., Joven-Sancho D., Ara I., Martín A., Menjón B. (2019). J. Organomet. Chem..

[cit18] Pang Y., Nöthling N., Leutzsch M., Kang L., Bill E., Van Gastel M., Reijerse E., Goddard R., Wagner L., SantaLucia D., DeBeer S., Neese F., Cornella J. (2023). Science.

[cit19] Yang X., Reijerse E. J., Nöthling N., SantaLucia D. J., Leutzsch M., Schnegg A., Cornella J. (2023). J. Am. Chem. Soc..

[cit20] Magre M., Kuziola J., Nöthling N., Cornella J. (2021). Org. Biomol. Chem..

[cit21] Kuziola J., Magre M., Nöthling N., Cornella J. (2022). Organometallics.

[cit22] Sowerby D. B. (1983). J. Chem. Soc., Dalton Trans..

[cit23] Godfrey S. M., McAuliffe C. A., Pritchard R. G., Sheffield J. M. (1996). Chem. Commun..

[cit24] Nikitin K., Jennings E. V., Al Sulaimi S., Ortin Y., Gilheany D. G. (2018). Angew. Chem..

[cit25] Kapuśniak Ł., Plessow P. N., Trzybiński D., Woźniak K., Hofmann P., Jolly P. I. (2021). Organometallics.

[cit26] Pal S., Hong L., Freire R. V. M., Farooq S., Salentinig S., Kilbinger A. F. M. (2023). Macromolecules.

[cit27] Akiba K., Okada K., Ohkata K. (1986). Tetrahedron Lett..

[cit28] Coughlin O., Krämer T., Benjamin S. L. (2023). Organometallics.

[cit29] Kuziola J. Dissertation: Synthesis and Characterization of Mono- and Bimetallic Organobismuth(v) Compounds, Ruhr-Universität Bochum, Max-Planck Institut für Kohlenforschung, 2024

[cit30] Yang L., Powell D. R., Houser R. P. (2007). Dalton Trans..

[cit31] Grimme S., Schreiner P. R. (2011). Angew. Chem., Int. Ed..

