# Supplementary material for: Synthesis and characterization of chlorotriarylbismuthonium salts
Source: Chem Commun (Camb). 2024 Aug 27;60(76):10532–5. doi: 10.1039/d4cc03364g (PMC11372555; doi:10.1039/d4cc03364g)

Supplementary Information (SI) for ChemComm.
This journal is © The Royal Society of Chemistry 2024

Supporting Information

Synthesis and Characterization of Chlorotriarylbismuthonium Salts

Jennifer Kuziola, Nils Nöthling, Markus Leutzsch, Josep Cornella

Max-Planck-Institut für Kohlenforschung, Kaiser-Wilhelm-Platz 1,

Mülheim an der Ruhr, 45470, Germany

cornella@kofo.mpg.de

Table of Contents

1. General methods **3**

2. Synthesis of Triarylbismuth Complex **4**

2.1 Synthesis and characterization data of tris(3,5-di-methylphenyl)bismuthane (**2**) 4

3. Synthesis of Triarylbismuth(V) dichlorides **5**

3.1 Characterization data of triphenylbismuth dichloride (**5**) 5

3.2 Characterization data of tris(3,5-di-methylphenyl)bismuth dichloride (**6**) 6

3.3 Characterization data of tris(3,5-di-tert-butylphenyl)bismuth dichloride (**7**) 6

3.4 Characterization data of trimesitylbismuth dichloride (**8**) 6

3.5 SC-XRD of triaryldibismuth dichlorides **6**, **7** and **8** 7

4. Synthesis of high-valent chlorobismuthonium tetrakis [3,5 bis(trifluoromethyl) phenyl]borate salts **8**

4.1 Characterization data of [Ph_3BiCl][BAr^F] (**9**) and [(Ph_3BiCl_2)Cl][BAr^F] (**9a**) 8

4.2 Characterization data of [*m*-Xyl_3BiCl][BAr^F] (**10**) and [(*m*-Xyl_3BiCl_2)Cl][BAr^F] (**10a**) 9

4.3 Characterization data of [3,5-$^{tBu}Ar_3BiCl$][BAr^F] (**11**) and [(3,5-$^{tBu}Ar_3BiCl_2$)Cl][BAr^F] (**11a**) 10

4.4 Characterization data of [Mes_3BiCl][BAr^F] (**12**) 11

5. Reactivity of Chlorotrimesitylbismuthonium salt (13) **12**

6. DOSY Experiments **13**

7. Determination of Lewis acidity **16**

8. Polymerization of THF-*d*$_8$ **19**

9. X-ray Data **22**

10. References **84**

11. NMR Data **85**

1. General methods

Unless otherwise stated, all manipulations were performed using standard Schlenk techniques under dry argon in flame-dried glassware or in a MBraun argon-filled glove box.

Anhydrous solvents were distilled from appropriate drying agents and were transferred under Ar (DCM and *n*-pentane (CaH_2), $CDCl_3$ (molecular sieves)) and stored over 4 Å molecular sieves under argon prior to use. Anhydrous $BiCl_3$ (99.9%, trace metal basis) and $NaBAr^F$ were purchased from Alfa Aesar, $BiPh_3$ was purchased from abcr. Trimesitylbismuth and tris(3,5-di-tert-butylphenyl)bismuthane have been synthesized by the reported method.[1]

NMR data were recorded using a Bruker AVIII HD 300 MHz, Bruker AVIII HD 400 MHz, Bruker AVIII 500 MHz or Bruker AVNeo 600 MHz NMR spectrometer. Chemical shifts (δ) are given in ppm, relative to deuterated solvent residual peak, and coupling constants (*J*) provided in Hz. ^{1}H and ^{13}C chemical shifts δ are reported relative to the solvent residual peaks as an internal reference. For ^{1}H NMR the following residual proton peaks of the deuterated solvents were used: $CDCl_3$, δ_H = 7.260 ppm, CD_2Cl_2 = 5.320 ppm and for ^{13}C NMR: $CDCl_3$, δ = 77.16 ppm. ^{19}F NMR data measured at 585 MHz was generally measured without ^{1}H decoupling.

2. Synthesis of Triarylbismuth Complex

Trimesitylbismuth and tris(3,5-di-*tert*-butylphenyl)bismuthane have been synthesized by the reported method,[1] while triphenylbismuth was commercially available.

2.1 Synthesis and characterization data of tris(3,5-di-methylphenyl)bismuthane (2)

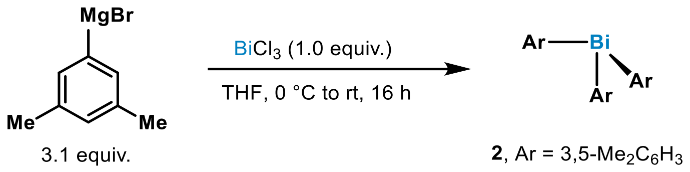

Procedure: A flame-dried Schlenk-flask was charged with activated magnesium turnings (214.7 mg, 8.8 mmol, 1.2 equiv.) and dissolved in in anhydrous THF (0.5 mL), followed by the addition of iodine and 0.5 mL of 5-bromo-xylene (0.37 mmol). The mixture was gently heated with a heat gun (70 °C) and a solution of the remaining 5-bromo-xylene (0.95 mL, 7 mmol, in total: 1.0 equiv.) in anhydrous THF (15 mL) was slowly added. The mixture was placed in an oil bath and heated at 70 °C for 3 h. Then, the solution was cooled to room temperature, additional 10 mL of anhydrous THF were added. The mixture was cooled to 0 °C and $BiCl_3$ was added in one portion. The reaction was allowed to warm up to room temperature and stirred overnight. The solvent was evaporated under reduced pressure and the crude mixture was extracted with anhydrous *n*-pentane (3× 10 mL). The removal of pentane under vacuum afforded tris(3,5-di-methylphenyl)bismuth (**2**). Yield: 88% (1.02 g)

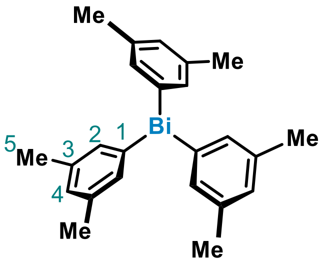

^{1}H NMR (300 MHz, $CDCl_3$) δ 7.41 (s, 6H, *H*-2), 6.95 (s, 3H, *H*-4), 2.27 (s, 18H, *H*-5).

^{13}C NMR (75 MHz, $CDCl_3$) δ 154.9 (*C*-1), 139.7 (*C*-3), 135.32 (*C*-4), 129.6 (*C*-2), 21.5 (*C*-5).

HRMS (ESI) calc'd for $C_{24}H_{27}Bi_1$ $[M]^+$ 524.191160, found 524.191148.

SC-XRD: Crystal of sufficient quality for SC-XRD were obtained *via* liquid-liquid diffusion of pentane into a concentrated solution of complex **2** in CH_2Cl_2 at +23 °C.

3. Synthesis of Triarylbismuth(V) dichlorides

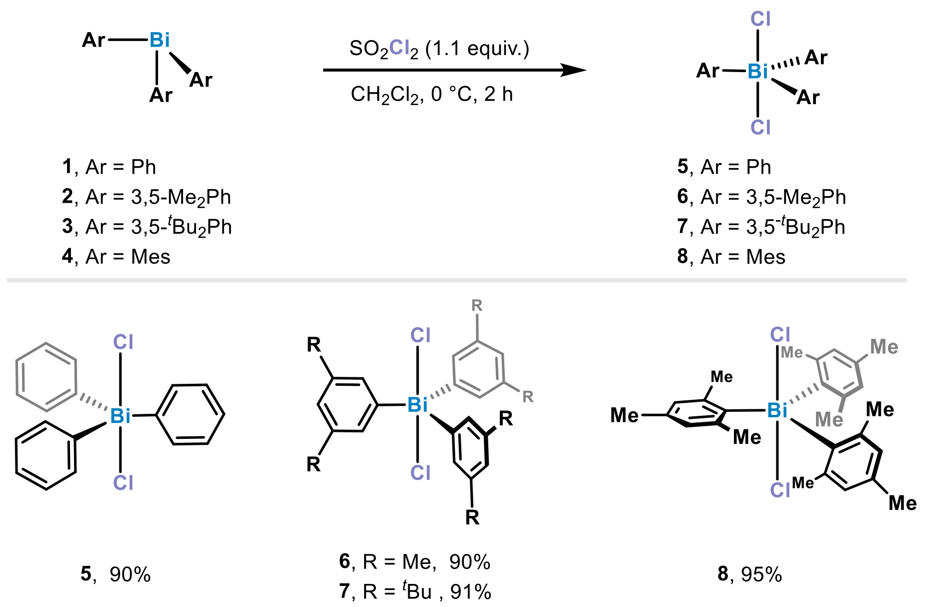

Scheme S1. Overview of the synthesized triarylbismuth dichlorides.

General procedure: An oven dried vial with a septum cap was charged with the corresponding triarylbismuthane (1.0 equiv.) under an Ar atmosphere and dissolved in anhydrous CH_2Cl_2, followed by the addition of SO_2Cl_2 (1.3 equiv.). The reaction was stirred for 2 h at room temperature and the solvent was evaporated via schlenk vaccum, affording the corresponding triarylbismuth dichloride.

3.1 Characterization data of triphenylbismuth dichloride (5)

Compound **5** was prepared following the general procedure from triphenylbismuth (**1**) (1.0 g, 2.38 mmol, 1.0 equiv.) and SO_2Cl_2 (0.3 mL, 3.1 mmol, 1.3 equiv.) in 20 mL of anhydrous CH_2Cl_2. Yield: 90% (1.1 g)

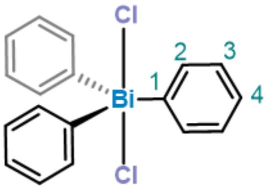

^{1}H NMR (300 MHz, $CDCl_3$) δ 8.63 – 8.46 (m, 6H, H-*Ar*), 7.67 (m, 6H, H-*Ar*), 7.62 – 7.44 (m, 3H, *H*-4).

^{13}C NMR (75 MHz, $CDCl_3$) δ 156.1 (*C*-1), 134.7 (C-*Ar*), 131.9 (C-*Ar*). 131.7 (C-*4*).

HRMS (ESI) calc'd for $C_{18}H_{15}BiCl_2Na_1$ $[M+Na]^+$ 533.02526, found 533.02470

3.2 Characterization data of tris(3,5-di-methylphenyl)bismuth dichloride (6)

Compound **6** was prepared following the general procedure from tris(3,5-di-methylphenyl)bismuth (**2**) (120 mg, 0.22 mmol, 1.0 equiv.) and SO_2Cl_2 (24.2 μL, 0.3 mmol, 1.3 equiv.) in 12 mL of anhydrous CH_2Cl_2. Yield: 90% (123 mg)

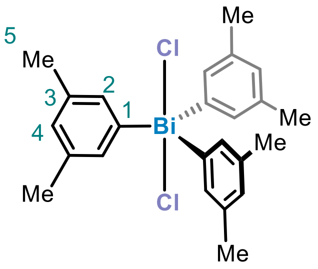

^{1}H NMR (500 MHz, $CDCl_3$) δ 8.06 (s, 6H, *H*-2), 7.14 (s, 3H, *H*-4), 2.39 (s, 18H, *H*-5).

^{13}C NMR (101 MHz, $CDCl_3$) δ 155.8 (*C*-1), 141.7 (*C*-3), 133.3 (*C*-4), 131.9 (*C*-2), 21.9 (*C*-5).

HRMS (ESI) calc'd for $C_{24}H_{27}BiCl_2Na_1$ $[M+Na]^+$ 617.11917, found 617.11860.

SC-XRD: Crystal of sufficient quality for SC-XRD were obtained *via* liquid-liquid diffusion of pentane into a concentrated solution of complex **6** in dichloromethane at +23 °C.

3.3 Characterization data of tris(3,5-di-tert-butylphenyl)bismuth dichloride (7)

Compound **7** was prepared following the general procedure from tris(3,5-di-tert-butylphenyl)bismuth (**3**) (52 mg, 0.07 mmol, 1.0 equiv.) and SO_2Cl_2 (7.3 μL, 0.09 mmol, 1.3 equiv.) in 5 mL of anhydrous CH_2Cl_2. Yield: 95% (53.7 mg).

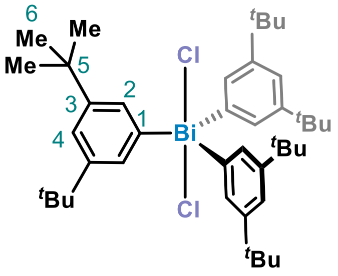

^{1}H NMR (300 MHz, $CDCl_3$) δ 8.40 (d, *J* = 1.6 Hz, 6H, *H*-2), 7.53 (t, *J* = 1.6 Hz, 3H, *H*-4), 1.37 (s, 27H, *H*-5).

^{13}C NMR (75 MHz, $CDCl_3$) δ 157.4 (*C*-1), 154.4 (*C*-3), 129.0 (*C*-4), 125.4 (*C*-2), 36.1 (*C*-5), 31.5 (*C*-6).

HRMS (ESI) calc'd for $C_{42}H_{63}BiCl_2Na_1$ $[M+Na]^+$ 869.39991, found 869.40030.

SC-XRD: Crystal of sufficient quality for SC-XRD were obtained *via* liquid-liquid diffusion of pentane into a concentrated solution of complex **7** in CH_2Cl_2 at +23 °C.

3.4 Characterization data of trimesitylbismuth dichloride (8)

Compound **8** was prepared following the general procedure from trimesitylbismuth (**4**) (70 mg, 1.23 mmol, 1.0 equiv.) and SO_2Cl_2 (16 μL, 0.16 mmol, 1.3 equiv.) in 7 mL of anhydrous CH_2Cl_2. Yield: 90% (71 mg).

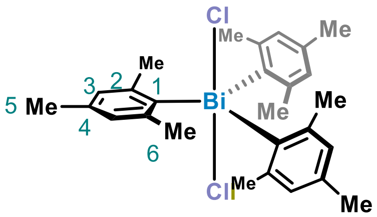

^{1}H NMR (400 MHz, $CDCl_3$) δ 7.16 (s, 6H, *H*-3), 2.71 (s, 18H *H*-6), 2.32 (s, 9H, *H*-5).

^{13}C NMR (101 MHz, $CDCl_3$) δ 167.8 (*C*-1), 142.0 (C-2), 141.3 (C-4), 132.3 (*C*-3), 25.6 (*C*-6), 21.0 (*C*-5).

HRMS (ESI) calc'd for $C_{27}H_{32}BiCl_2$ [M] 635.16742, found 635.16796.

SC-XRD: Crystal of sufficient quality for SC-XRD were obtained *via* liquid-liquid diffusion of pentane into a concentrated solution of complex **8** in CH_2Cl_2 at +23 °C.

3.5 SC-XRD of triaryldibismuth dichlorides 6, 7 and 8

Complexes **5** and **8** are known,[2,3] **6** and **7** represent new structures.

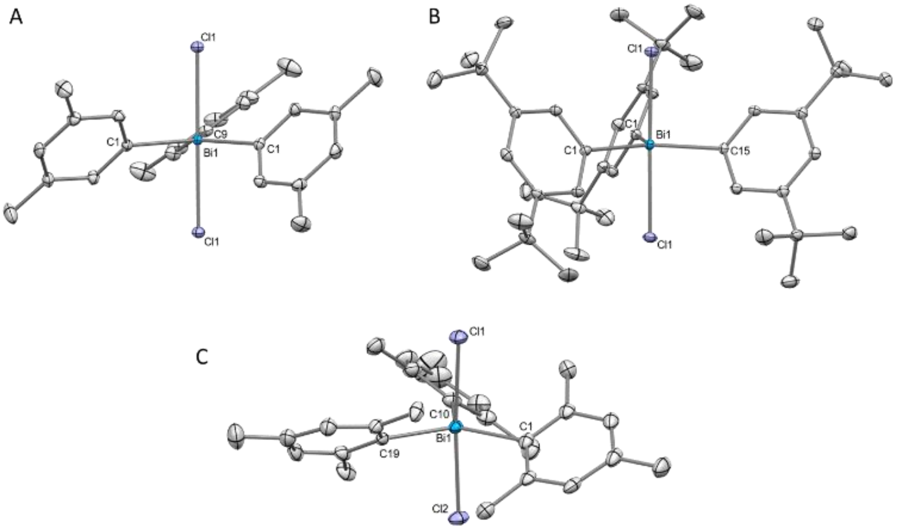

Figure S1. Solid state of structure of **6** (A), **7** (B)[4] and **8** (C)[4]. Ellipsoids are drawn at the 50% probability level. H atoms are omitted for clarity. Selected bond lengths (Å) and angles (°): (A) **6**, Bi1-C1: 2.1980(15), Bi1-C9: 2.202(2), Bi1-Cl1: 2.5898(4), Cl1–Bi1–Cl1, 177.391(18). (B) **7**, Bi1-C1: 2.221(2), Bi1-C15: 2.212(3), Bi1–Cl1, 2.5954(5), Cl1–Bi1–Cl1, 178.59(3). Selected bond lengths (Å) and angles (°) are the average of two molecules in the asymmetric unit cell: (C), **8**: Bi1-C1:2.239(3), Bi1-C19: 2.2315(3), Bi1-C10: 2.235(3), Bi1-Cl1: 2.5977(8), Bi1-Cl2: 2.63265(8), Cl1-Bi1-Cl2: 175.94(3).

Structures **6**, **7** and **8** adopt a slightly distorted trigonal bipyramidal geometry with the chloride atoms occupying the apical positions. The Bi1–Cl1 bond lengths of **6**, **7** and **8** are in the range of the unsubstituted Ph_3BiCl_2 [**5**: 2.5896(10) Å;[2] **6**: 2.5898(4); **7**: Bi1–Cl1 = 2.5954(5) Å; 8: 2.6151(8) Å;]. Whereas the fluorinated analogues $^{tBu}Ar_3BiF_2$ (^{tBu}Ar = 3,5-tBu$_2$-C_6H_3) and Mes_3BiF_2 (Mes = 2,4,6-Me_3-C_6H_3) display a quasi-linear F–Bi–F axis [$^{tBu}Ar_3BiF_2$: 179.1(2)°; Mes_3BiF_2: 179.00(5)°],[1] complexes **6**, **7** and **8** exhibit deviations from linearity with Cl–Bi–Cl angles of 177.391(18)° (**6**), 178.59(3)° (**7**) and 175.94(3)° (**8**). The replacement of the more electronegative fluoride ligands with chlorine atoms leads to a distinct organization of the aryl groups, adopting rather a propeller-like configuration in all cases.

This effect can be attributed to the weaker interactions of the *ortho* C–H bonds with the Cl as a result of the lower electronegativity compared to fluorine.

4. Synthesis of high-valent chlorobismuthonium tetrakis [3,5 bis(trifluoromethyl) phenyl]borate salts

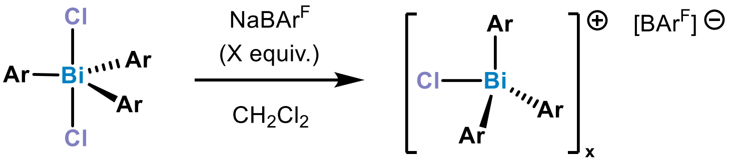

[Ar = Ph, 3,5-Me$_2$-C$_6$H$_3$, 3,5-^{t}Bu$_2$-C$_6$H$_3$, Mes]

General procedure: In the glovebox, an oven dried vial with a septum cap was charged with the corresponding triarylbismuth dichloride (1.0 equiv.) and either 1.0 or 0.5 equiv. of NaBAr^F, followed by the addition of 5 mL of anhydrous dichloromethane. The reaction was stirred for 2 h at room temperature and the precipitate was filtered off via a syringe filter. The solvent was evaporated under reduced pressure via the Schlenk line, affording the corresponding triarylbismuth dichloride.

4.1 Characterization data of [Ph$_3$BiCl][BAr^F] (9) and [(Ph$_3$BiCl$_2$)Cl][BAr^F] (9a)

Addition of 1.0 equiv. of NaBAr^F: Prepared according to the general procedure from triphenylbismuth dichloride (**1**) (50 mg, 0.08 mmol, 1.0 equiv.) and NaBAr^F (52 mg, 0.06 mmol, 1.0 equiv.) in 5 mL of anhydrous dichloromethane. The analytical data refer to the formal monomeric chlorobismuthonium species (**9**) in solution. Yield: 48 mg

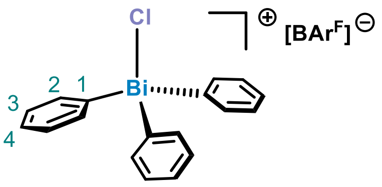

^{1}H NMR (300 MHz, CDCl$_3$) δ 7.83 – 7.62 (m, 23H, BAr^F, *H*-1/ *H*-2/ *H*-3/*H*-4), 7.47 (s, 4H, *BAr^F*).

^{13}C NMR (101 MHz, CDCl$_3$) δ 161.8 (q, *J* = 49.8 Hz, *BAr^F*), 148.3 (*C*-1), 134.9 (*BAr^F*), 134.5 (*C*-Ar), 134.3 (*C*-4), 133.9 (*C*-Ar), 129.6 – 128.8 (m) (*BAr^F*), 124.7 (q, *J* = 272.6 Hz, *BAr^F*), 118.2 – 116.9 (m) (*BAr^F*).

^{19}F NMR (282 MHz, CDCl$_3$) δ –62.3.

HRMS (ESI): calc'd for $C_{18}H_{15}Bi_1Cl_1$ [M]$^+$ 475.06608, found 475.06619.

Addition of 0.5 equiv. of NaBAr^F: Prepared according to the general procedure from triphenylbismuth dichloride (**1**) (50 mg, 0.08 mmol, 1.0 equiv.) and NaBAr^F (17.3 mg, 0.09 mmol, 0.5 equiv.) in 5 mL of anhydrous dichloromethane. The analytical data refer to the dimeric chlorobismuthonium species (**9a**) in solution and solid-state. Yield: 87% (43.3 mg)

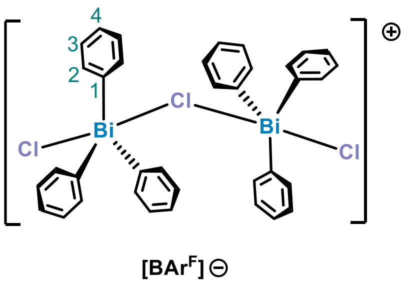

^{1}H NMR (600 MHz, $CDCl_3$) δ 7.99 – 7.94 (m, 12H, *H*-2), 7.72 – 7.69 (m, 8H, *BAr^F*), 7.61 (m, 12H, *H*-3), 7.59 – 7.54 (m, 6H, *H*-4), 7.49 (s, 4H, *BAr^F*).

^{13}C NMR (151 MHz, $CDCl_3$) δ 161.9 (q, *J* = 49.8 Hz, *BAr^F*), 153.7 (*C*-1), 134.9 (*BAr^F*), 134.3 (*C*-2), 133.0 (*C*-3), 132.9 (*C*-4), 129.0 (qq, *J* = 31.5, 2.8 Hz, *BAr^F*), 124.7 (q, *J* = 272.5 Hz, *BAr^F*), 117.6 (hept, *J* = 4.0 Hz, *BAr^F*).

^{19}F NMR (565 MHz, $CDCl_3$) δ –62.4.

HRMS (ESI): calc'd for $C_{36}H_{30}Bi_2Cl_3$ $[M]^+$ 985.10224, found 958.10156.

SC-XRD: Crystal of sufficient quality for SC-XRD was obtained *via* liquid-liquid diffusion of pentane into a concentrated solution of complex **9a** in CH_2Cl_2 at +23 °C.

4.2 Characterization data of [*m*-Xyl$_3$BiCl][BAr^F] (**10**) and [(*m*-Xyl$_3$BiCl$_2$)Cl][BAr^F] (**10a**)

Addition of <u>1.0 equiv.</u> of NaBAr^F: Prepared according to the general procedure from **6** (50 mg, 0.084 mmol, 1.0 equiv.) and NaBAr^F (78.1 mg, 0.088 mmol, 1.05 equiv.) in 5 mL of anhydrous dichloromethane. The analytical data refer to the monomeric chlorobismuthonium species in solution and solid-state. Yield: 91.4 % (64 mg)

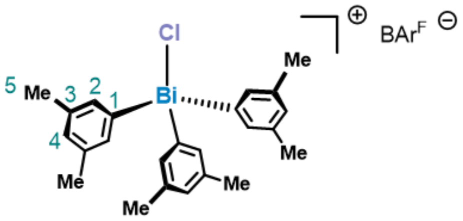

^{1}H NMR (600 MHz, $CDCl_3$) δ 7.73 – 7.61 (m, 8H, *BAr^F*), 7.47 (s, 4H, *BAr^F*), 7.38 (s, 6H, *H*-2), 7.33 – 7.29 (m, 3H, *H*-4), 2.38 – 2.36 (m, 18H, *H*-5).

^{13}C NMR (151 MHz, $CDCl_3$) δ 161.8 (q, *J* = 49.9 Hz, *BAr^F*), 146.1–145.1 (*C*-1), 144.8 (C-3), 136.5 (*C*-4), 134.9 (*BAr^F*), 131.3 (*C*-2), 129.9 – 128.5 (m) (*BAr^F*), 124.6 (q, *J* = 272.6 Hz, *BAr^F*), 118.5 – 115.9 (m) (*BAr^F*), 21.8 (*C*-5).

^{19}F NMR (565 MHz, $CDCl_3$) δ –62.4.

HRMS (ESI): calc'd for $C_{24}H_{27}Bi_1Cl_1$ $[M]^+$ 559.16011, found 559.15998.

SC-XRD: Crystal of sufficient quality for SC-XRD was obtained *via* liquid-liquid diffusion of pentane into a concentrated solution of complex **10** in CH_2Cl_2 at –18 °C.

Addition of <u>0.5 equiv.</u> of NaBAr^F: Prepared according to the general procedure from **6** (47 mg, 0.08 mmol, 1.0 equiv.) and NaBAr^F (35.1 mg, 0.04 mmol, 0.5 equiv.) in 5 mL of anhydrous dichloromethane. The analytical data refer to the formal dimeric chlorobismuthonium species (**10a**) in solution. Yield: 62 mg

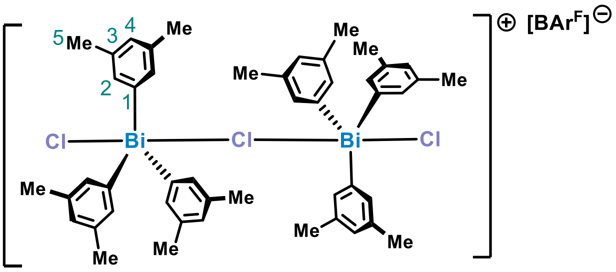

^{1}H NMR (400 MHz, $CDCl_3$) δ 7.74 – 7.68 (m, 8H, *BAr^F*), 7.61 (s, 12H, *H*-2), 7.50 (s, 4H, *BAr^F*), 7.16 (s, 6H, *H*-4), 2.26 (s, 36H, *H*-5).

^{13}C NMR (101 MHz, $CDCl_3$) δ 161.8 (q, *J* = 49.9 Hz, *BAr^F*), 153.4 (*C*-1), 143.0 (*C*-3), 135.0 (*BAr^F*), 134.4 (*C*-4), 131.4 (*C*-2), 129.7 – 128.3 (m) (*BAr^F*), 124.7 (q, *J* = 272.4 Hz, *BAr^F*), 118.0 – 116.9 (m) (*BAr^F*), 21.7 (*C*-5).

^{19}F NMR (282 MHz, $CDCl_3$) δ –62.3.

Based on the HRMS analysis several species have been detected:

1. HRMS (ESI): calc'd for $C_{24}H_{27}Bi_1Cl_1$ $[M]^+$ 559.15995, found 559.15998.

2. HRMS (ESI): calc'd for $C_{48}H_{54}Bi_2Cl_3$ $[M]^+$ 1153.28936, found 1153.28963.

4.3 Characterization data of [3,5-$^{tBu}Ar_3$BiCl][BAr^F] (**11**) and [(3,5-$^{tBu}Ar_3BiCl_2$)Cl][BAr^F] (**11a**)

Addition of 1.0 equiv. of NaBAr^F: Prepared according to the general procedure from complex **7** (50 mg, 0.06 mmol, 1.0 equiv.) and NaBAr^F (52mg, 0.06 mmol, 1.0 equiv.) in 5 mL of anhydrous dichloromethane. The analytical data refer to the monomeric chlorobismuthonium species (**11**) in solution and the yield is based on the dinuclear structure in solid state. Yield: 79 % (59 mg)

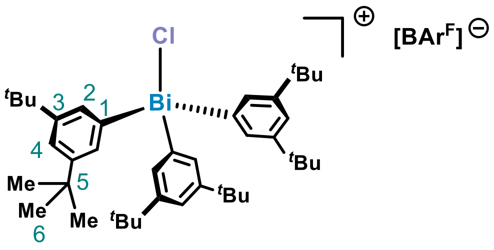

^{1}H NMR (600 MHz, $CDCl_3$) δ 7.80 (t, *J* = 1.6 Hz, 3H, *H*-4), 7.69 – 7.64 (m, 8H, *BAr^F*), 7.62 (d, *J* = 1.6 Hz, 6H, *H*-2), 7.46 (s, 4H, *BAr^F*), 1.34 (s, 54H, *H*-6).

^{13}C NMR (151 MHz, $CDCl_3$) δ 161.83 (q, *J* = 49.9 Hz, *BAr^F*), 157.9 (*C*-3), 147.5 (bs, *C*-1), 134.9 (*BAr^F*), 129.1 (*C*-4), 128.9 (qq, *J* = 31.5, 2.8 Hz, *BAr^F*), 128.1 (*C*-2), 124.7 (q, *J* = 272.6 Hz, *BAr^F*), 117.5 (hept, *J* = 4.0 Hz, *BAr^F*), 36.5 (*C*-5), 31.2 (*C*-6).

^{19}F NMR (564 MHz, $CDCl_3$) δ –62.4.

Based on the HRMS analysis several species have been detected:

1. HRMS (ESI): 811.44 = $[C_{42}H_{63}Bi_1Cl_1]^+$ (**11**) equal to $[C_{42}H_{63}Bi_1Cl_2 – Cl]^+$ (**7**);

HRMS (ESI): calc'd for $C_{42}H_{63}Bi_1Cl_1$ $[M]^+$ 811.44131, found 811.44168.

2. HRMS (ESI): calc'd for $C_{84}H_{126}Bi_2Cl_3$ $[M]^+$ 1657.85177, found 1657.85276. (**11a**)

SC-XRD: Crystal of sufficient quality for SC-XRD were obtained *via* liquid-liquid diffusion of pentane into a concentrated solution of complex **11** in CH_2Cl_2 at -18 °C, affording dimeric Bi complex **11a**.

Addition of 0.5 equiv. of NaBAr^F: Prepared according to the general procedure from complex **7** (30 mg, 0.035 mmol, 1.0 equiv.) and NaBAr^F (15.7mg, 0.017 mmol, 0.5 equiv.) in 5 mL of anhydrous dichloromethane. The analytical data refer to the dimeric chlorobismuthonium species in solution and the yield is based on the dinuclear structure in solid state. Yield: 88% (45 mg)

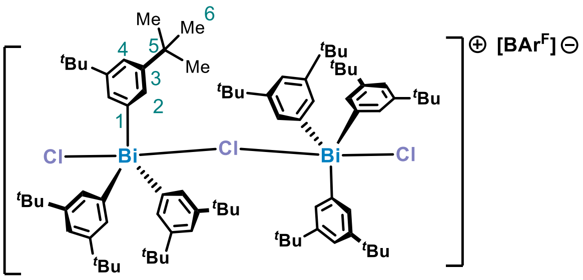

^{1}H NMR (600 MHz, $CDCl_3$) δ 7.94 (s, 12H, *H*-2), 7.68 (m, *J* = 5.2, 2.2 Hz, 8H, *BAr^F*), 7.63 (d, *J* = 1.6 Hz, 6H, *H*-4), 7.48 (s, 4H, *BAr^F*), 1.29 (s, 108H, *H*-6).

^{13}C NMR (151 MHz, $CDCl_3$) δ 161.9 (q, *J* = 49.9 Hz, *BAr^F*), 156.2 (*C*-3), 153.3 (*C*-1), 134.9 (*BAr^F*), 129.0 (qq, *J* = 31.4, 2.9 Hz, *BAr^F*), 128.6 (*C*-2), 127.1 (*C*-4), 124.7 (q, *J* = 272.4 Hz, *BAr^F*), 119.7 – 116.6 (m) (*BAr^F*), 36.3 (*C*-5), 31.4 (*C*-6).

^{19}F NMR (564 MHz, $CDCl_3$) δ –62.3.

Based on the HRMS analysis several species have been detected:

1. HRMS (ESI): 811.44 = $[C_{42}H_{63}Bi_1Cl_1]^+$ (**11**) equal to $[C_{42}H_{63}Bi_1Cl_2 - Cl]^+$ (**7**);

HRMS (ESI): calc'd for $C_{42}H_{63}Bi_1Cl_1$ $[M]^+$ 811.44131, found 811.44168.

2. HRMS (ESI): calc'd for $C_{84}H_{126}Bi_2Cl_3$ $[M]^+$ 1657.85177, found 1657.85276. (**11a**)

4.4 Characterization data of [Mes_3BiCl][BAr^F] (12)

Addition of 1.0 equiv. of NaBAr^F: Prepared according to the general procedure from complex **8** (50 mg, 0.08 mmol, 1.0 equiv.) and NaBAr^F (73 mg, 0.08 mmol, 1.0 equiv.) in 5 mL of anhydrous dichloromethane. Yield: 90% (104 mg)

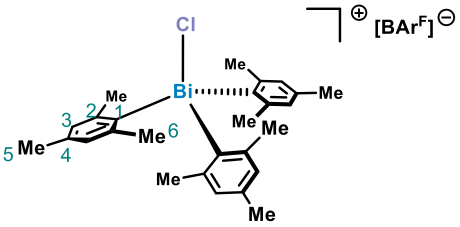

^{1}H NMR (400 MHz, $CDCl_3$) δ 7.71 – 7.66 (m, 8H, *BAr^F*), 7.49 (s, 4H, *BAr^F*), 7.27 (s, 6H, *H*-3), 2.42 (s, 18H, *H*-6), 2.34 (s, 9H, *H*-5).

^{13}C NMR (101 MHz, $CDCl_3$) δ 161.8 (q, *J* = 49.9 Hz, *BAr^F*), 157.5 (*C*-1), 145.5, (*C*-4), 141.9 (*C*-2), 134.9 (*BAr^F*), 133.8 (*C*-3), 129.1 (qq, *J* = 31.6, 2.8 Hz, *BAr^F*), 124.6 (q, *J* = 272.5 Hz, *BAr^F*), 117.6 (hept, *J* = 4.0 Hz, *BAr^F*), 24.6 (*C*-6), 21.1 (*C*-5).

^{19}F NMR (282 MHz, $CDCl_3$) δ –62.4.

HRMS (ESI) calc'd for $C_{27}H_{33}BiCl_1$ [M-BAr^F]$^+$ 601.20621, found 601.20693.

5. Reactivity of Chlorotrimesitylbismuthonium salt (13)

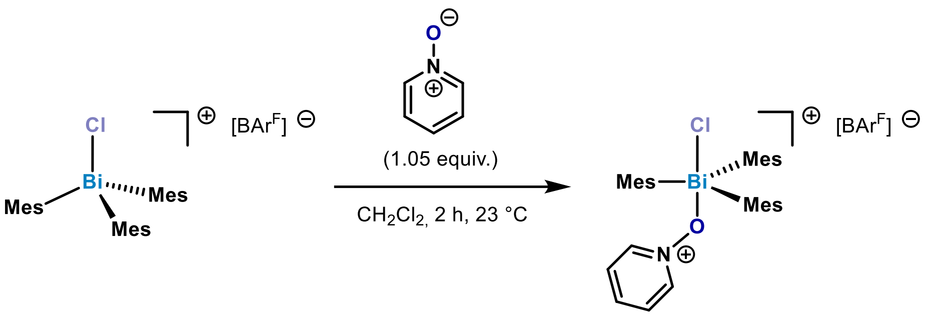

Procedure: In the glovebox, an oven dried vial with a septum cap was charged with the corresponding chlorotrimesitylbismuthonium salt (**12**) (50 mg, 0.03 mmol, 1.0 equiv.) and pyridine-N-oxide (3.4 mg, 0.04 mmol, 1.05 equiv.), followed by the addition of 5 mL of anhydrous dichloromethane. The reaction was stirred for 2 h at room temperature, and afterwards the solvent was evaporated under reduced pressure using the Schlenk line, affording a light yellowish solid. Yield: 87.7 % (46.7 mg)

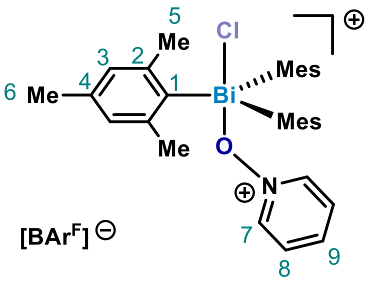

^{1}H NMR (600 MHz, $CDCl_3$) δ 7.98 (d, *J* = 6.1 Hz, 2H, *H*-7), 7.72 – 7.66 (m, 8H, *BAr^F*), 7.52 (t, *J* = 7.7 Hz, 1H, *H*-9), 7.50 (s, 4H, *BAr^F*), 7.38 – 7.31 (m, 2H, *H*-8), 7.22 (s, 6H, *H*-3), 2.55 (s, 18H, *H*-5), 2.33 (s, 9H, *H*-6).

^{13}C NMR (151 MHz, $CDCl_3$) δ 163.3 (*C*-1), 161.8 (q, *J* = 49.9 Hz, *BAr^F*), 143.4 (*C*-4), 142.0 (*C*-2), 139.0 (*C*-7), 134.9 (m) (*BAr^F*), 133.1 (*C*-3), 132.8 (bs) (*C*-9), 129.1 (qq, *J* = 31.5, 2.8 Hz, *BAr^F*), 127.3 (*C*-8), 124.6 (q, *J* = 272.7 Hz, *BAr^F*), 117.6 (hept, *J* = 4.0 Hz, *BAr^F*), 25.0 (*C*-5), 21.0 (*C*-6).

^{19}F NMR (565 MHz, $CDCl_3$) δ –62.5.

The connectivity of the pyridine-N-oxide with the Bi-species is supported based on a NOESY cross peak between *H*-5 and *H*-7. The carbon signal of *C*-9 is surprisingly broad. The observed ^{15}N NMR shift (-108.0 ppm) is lower compared to the value of the free oxide (-87.5 ppm, DMSO), but higher compared to the pyr-NO*TFA (-135.7ppm).[5] As also the MS department could not find the mass of the cationic species, the binding of the N-oxide might be weak and might also explain the observations from the NMR data.

HRMS: MS department could not find the mass of the cationic species (**13**), but rather compound **12**.

SC-XRD: Crystal of sufficient quality for SC-XRD was obtained *via* liquid-liquid diffusion of pentane into a concentrated solution of complex **13** in CH_2Cl_2 at –18 °C

6. DOSY Experiments

After we observed that the addition of 1.0 equiv. of NaBAr^F to complex **7** resulted in the formation of a formally monomeric chlorobismuthonium salt **11** in solution and a dimeric bismuthonium salt **11a** in solid-state, we repeated the reaction with 0.5 equiv. of NaBAr^F (Scheme S2). This reaction led to the formation of a dimeric chlorobismuthonium salt **11a** in solution and solid state. However, the ^{1}H NMR spectrum of this reaction displays broader signals in comparison to the addition of 1.0 equiv. of NaBAr^F (Figure S2,), indicating a fast dynamic exchange between different species in solution.

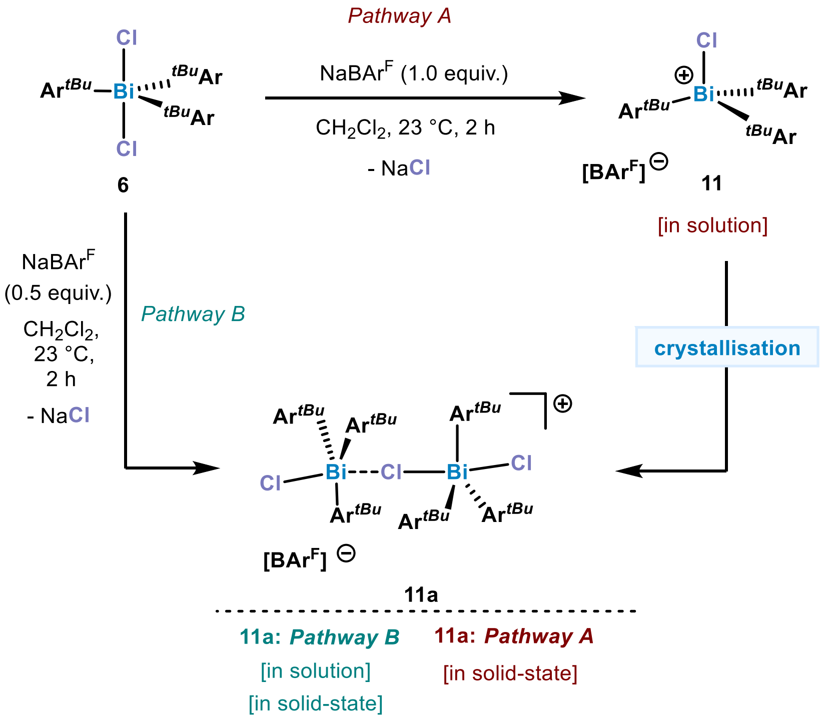

Scheme S2. Synthesis and crystallization of chlorotriaryl bismuthonium salts **11** and **11a**.

In contrast to the reaction of **6** with 0.5 equiv. of NaBAr^F where compound **6** crystallized, the installation of ^{t}Bu-substituents in *meta*-position might facilitate the formation of the dimeric structure *via* London dispersion forces.[6–8] In order to understand the dynamic behaviour of this chlorobismuthonium species **11** and **11a**, we carried out this reaction with different amounts of NaBAr^F (1.0, 0.85, 0.5 and 0.25 equiv.). As depicted in Figure 2, a trend can be clearly observed: as the amounts of NaBAr^F decrease, the signals of the observed bismuth species are more similar to **6**, however, separate Bi species were not observed at any time in the ^{1}H NMR spectra.

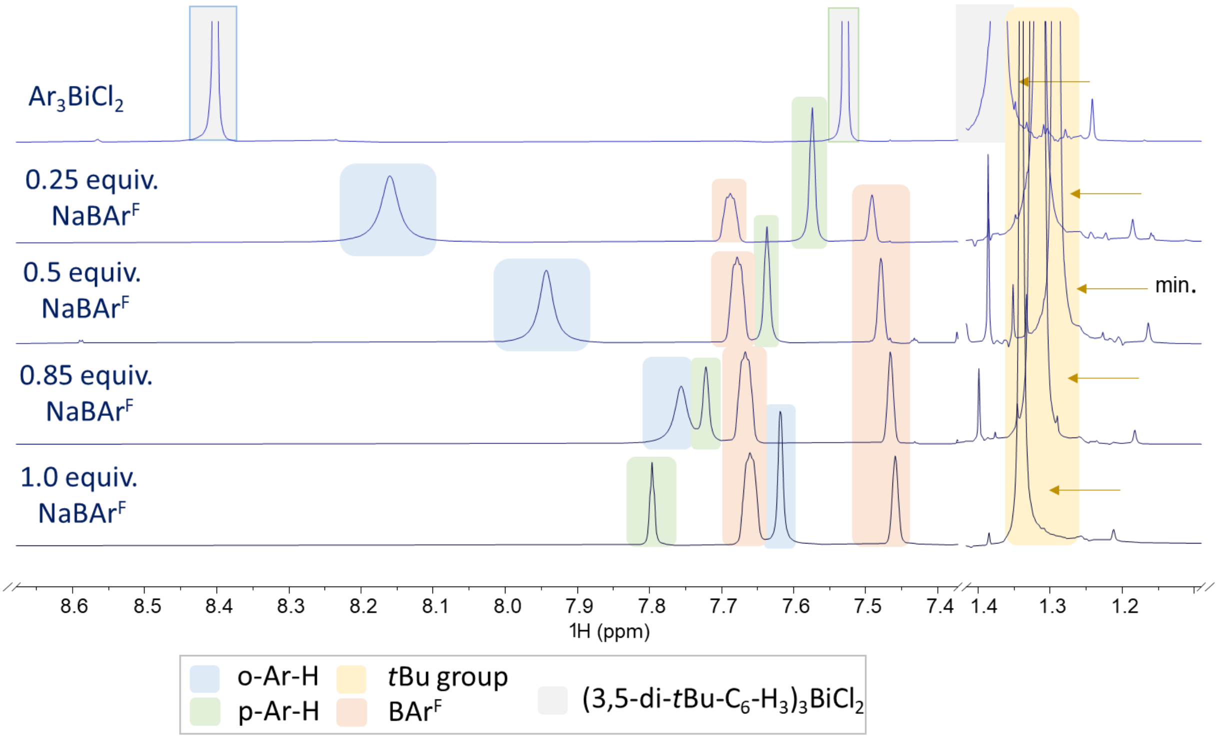

Figure S2. Stacked ^{1}H NMR spectra of the reaction of complex **7** with various amount of NaBAr^{F} (1.0, 0.85, 0.5, 0.25 equiv.) in CH_2Cl_2 at 25 °C for 2 h. The precipitate was filtered off and the ^{1}H NMR spectra were recorded (500 MHZ, $CDCl_3$).

Furthermore, the addition of 1.0 equiv. of NaBAr^{F} leads to sharper signals, whereas a reduced amount of the halide scavenger results in broader signals. Whereas all the aromatic H signals experience a shift with an increased amount of NaBAr^{F}, the chemical shift of the ^{t}Bu groups reaches a minimum at 0.5 equiv.. This indicates that a fast and reversible equilibrium between at least three different species is present in solution after the abstraction of a chloride from the Bi. These results again, are in stark contrast to the behaviour of the analogous fluorobismuthonium salt.[1]

To gain deeper insights into the distinct behavior of chlorobismuthonium salts in both solution and solid-state, we conducted DOSY (Diffusion Ordered Spectroscopy) experiments. For this, different amount of equivalents of NaBAr^{F} (1.0, 0.85, 0.5, 0.25 equiv.) were added to **7**, and the reaction was left to stir for 2 h at room temperature. After this time, the precipitate was removed through filtration, and subsequent evaporation under Schlenk vacuum conditions yielded the corresponding chlorobismuthonium salts, for which ^{1}H NMR spectra were recorded (see Figure S2). Based on these, the self-diffusion coefficients D_{self} were determined (Figure S3).

Figure S3 illustrates the self-diffusion coefficients. Increased quantities of $NaBAr^F$ result in decreased D_{self} values, suggesting the formation of molecules with higher molecular masses, potentially as dimeric species. However, it cannot be ruled out that the presence of BAr^F as anion might distort the values in the solution.

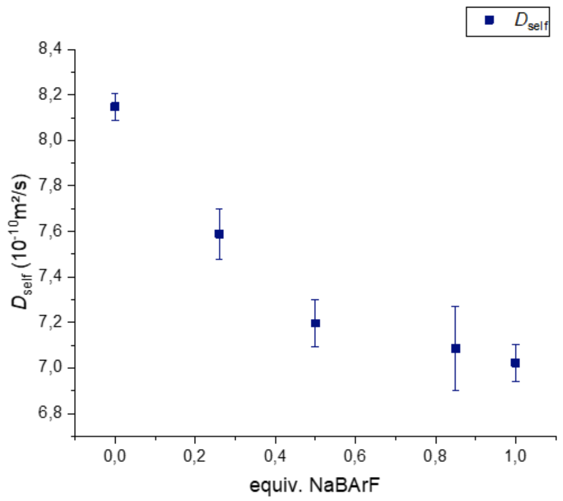

Figure S3. D_{self} values of chlorobismuthonium salts after the addition of different equivalents of $NaBAr^F$.

7. Determination of Lewis acidity

The Lewis acidity of the chlorotriarylbismuthonium salts was determined by the Gutmann-Beckett-method. This method is based on the use of trietylphospine oxide ($Et_3P{=}O$) as Lewis base which interacts with the Lewis acid, resulting in a chemical shift (δ) of the phosphine oxide in the ^{31}P NMR spectrum. The strength of the Lewis acidity is determined by the acceptor number (AN), which is calculated as followed:

$$AN\ (Et_3PO) = 2.21 \cdot (\delta_{P(LA)} - 41.0)$$

Table S1. Determination of the Lewis acidity by the acceptor number in CD_2Cl_2. Equimolar amounts of the corresponding Lewis acid and Lewis base (Et_3PO) were dissolved in CD_2Cl_2. The ^{31}P NMR spectra were recorded at 121.5 MHz at 298 K. Stacked ^{31}P NMR spectra of the corresponding $Ar_3BiCl{\cdot}OPEt_3$ adduct are shown in Figure S8 and S9.

**Entry**	**Compound**		**δ ^{31}P (ppm)**	**AN**
1	Et_3PO		50.45	
Formally monomeric Bi species:				
2	$[Ph_3BiCl][BAr^F]$ (**9**)		69.86	63.78
3	$[3,5\text{-}Me_2\text{-}C_6H_3\text{-}BiCl][BAr^F]$ (**10**)		68.29	60.31
4	$[3,5\text{-}t\text{Bu}_2\text{-}C_6H_3\text{-}BiCl][BAr^F]$ (**11**)		67.67	58.94
5	$[Mes_3BiCl][BAr^F]$ (**12**)		60.85	43.86
Formally dimeric Bi species:				
6	$[(Ph_3BiCl)_2][BAr^F]$ (**9a**)		69.88	63.82
7	$[((3,5\text{-}Me_2\text{-}C_6H_3)_3\text{-}BiCl)_2][BAr^F]$ (**10a**)		68.30	60.33
8	$[((3,5\text{-}t\text{Bu}_2\text{-}C_6H_3)_3\text{-}BiCl)_2][BAr^F]$ (**11a**)		67.67	58.94

Table S1 displays the ^{31}P NMR chemical shifts and acceptor numbers of the chlorotriarylbismuthonium salts (9-12 and 9a-11a). A closer look into the chemical shifts and acceptor numbers of the formally monomeric Bi species indicates a ligand effect on the Lewis acidity. The more steric the aryl-ligand, the lower the AN, resulting in a lower Lewis acidity. The ^{31}P NMR of complex **9** displays three signals at δ_P =78.65, δ_P = 69.86 and δ_P = 55.64 ppm in CD_2Cl_2.

The experimental observation on the formally dimeric Bi species show the same trend as the formally monomeric. Furthermore, the dimeric species show identical ^{31}P NMR shifts as the corresponding monomeric structures, indicating the formation of a monomeric $Ar_3BiCl{\cdot}OPEt_3$ adduct and Ar_3BiCl_2, which is further confirmed by the ^{1}H NMR spectra, emphasizing a dynamic equilibrium in solution (Figure S4).

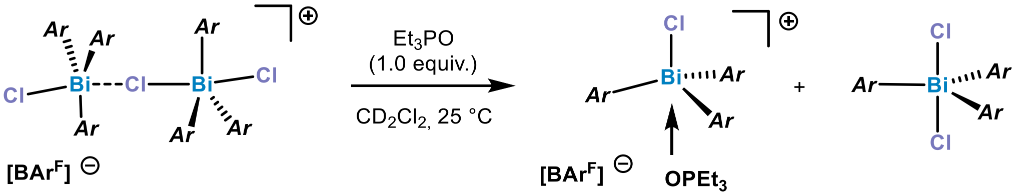

Figure S4. Formation of $Ar_3BiCl{\cdot}OPEt_3$ adduct and Ar_3BiCl_2 after the addition of Et_3PO to dimeric chlorobismuthonium salts (**9a-11a**).

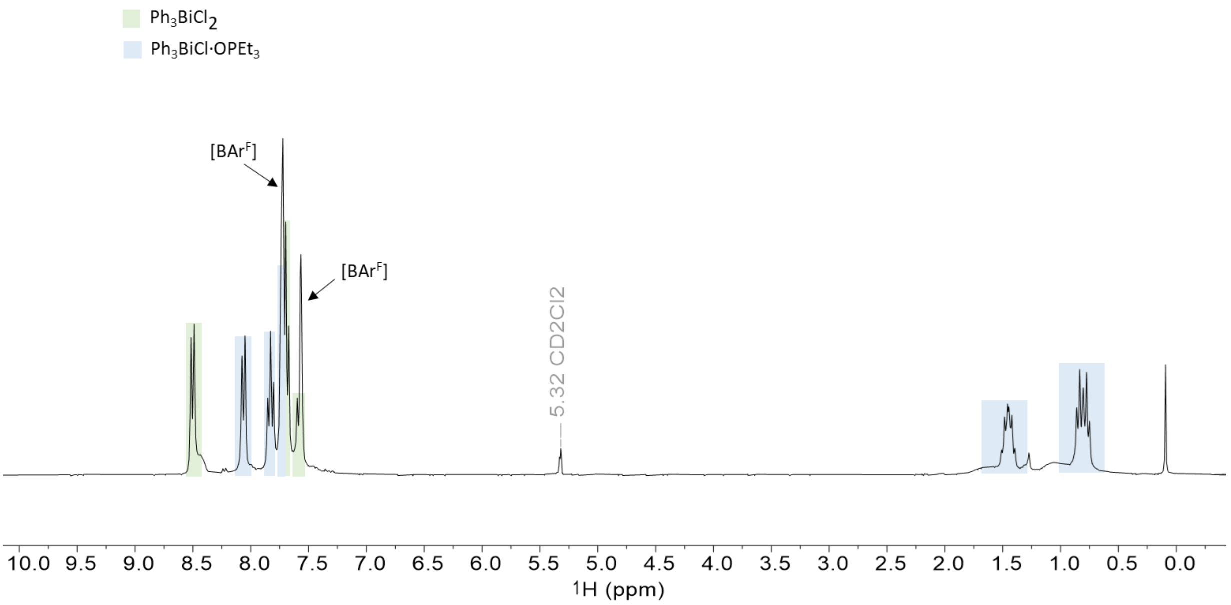

Figure S5. ^{1}H NMR spectra of the mixture of Lewis acid **9a** with Et_3PO (121.5 MHz, 298K, CD_2Cl_2).

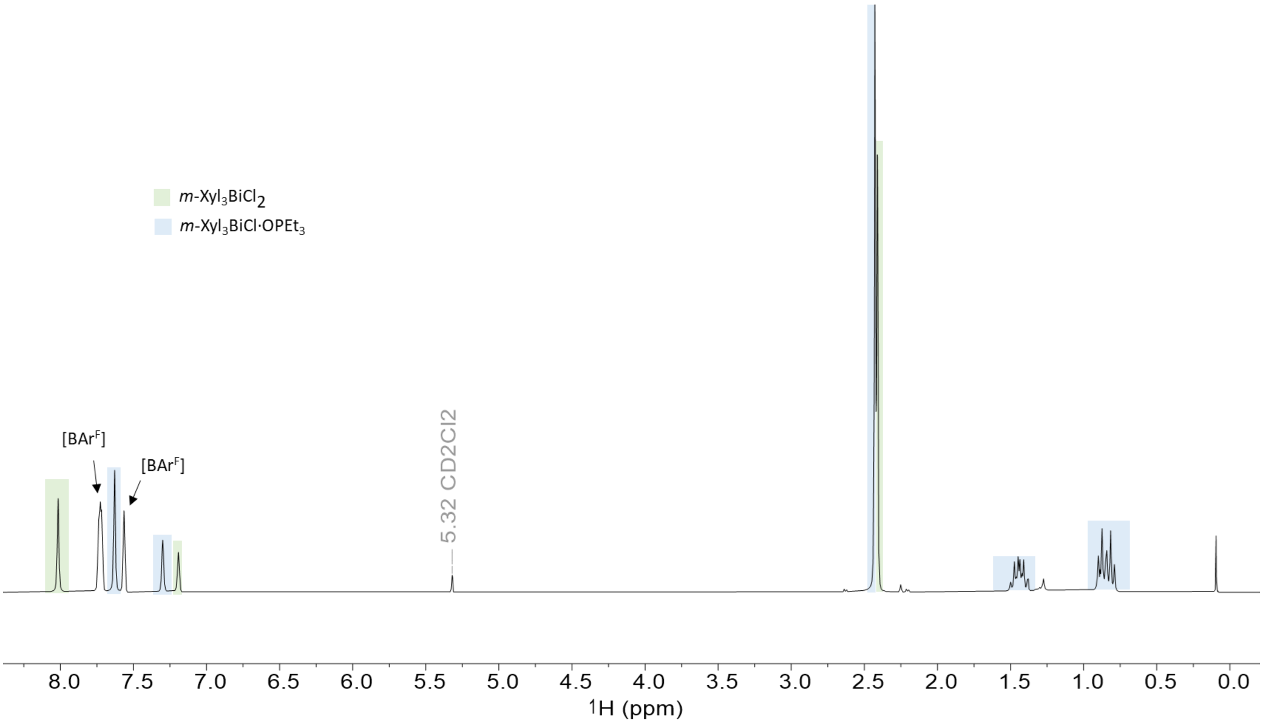

Figure S6. ^{1}H NMR spectra of the mixture of Lewis acid **10a** with Et_3PO (121.5 MHz, 298K, CD_2Cl_2).

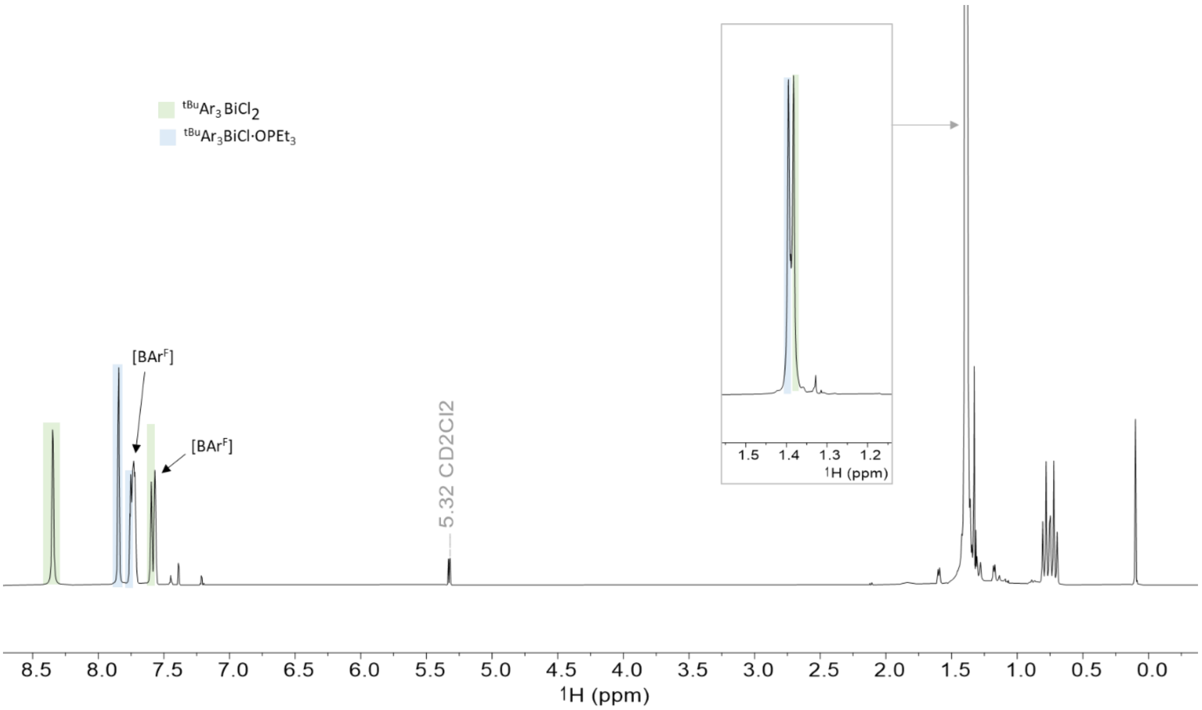

Figure S7. ^{1}H NMR spectra of the mixture of Lewis acid **11a** with Et_3PO (121.5 MHz, 298K, CD_2Cl_2).

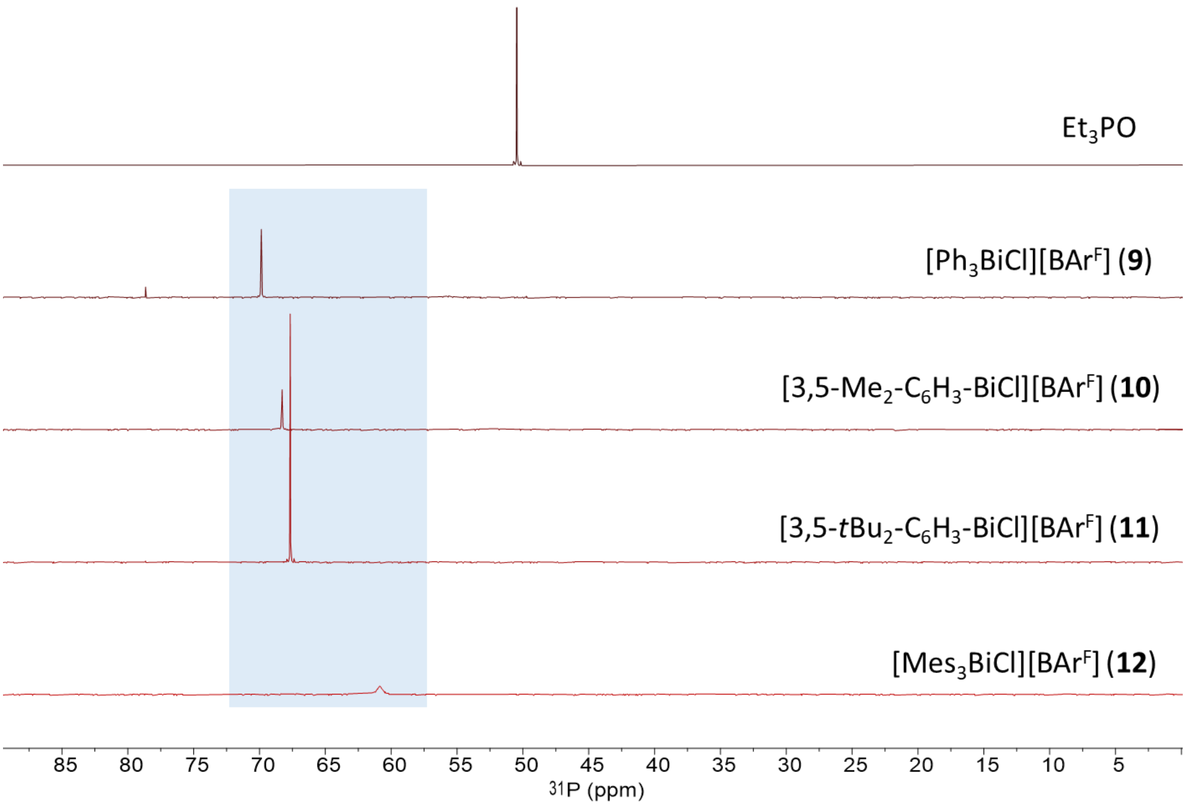

Figure S8. Stacked ^{31}P NMR spectra of the mixture of Lewis acid **9-12** with Et_3PO (121.5 MHz, 298K, CD_2Cl_2).

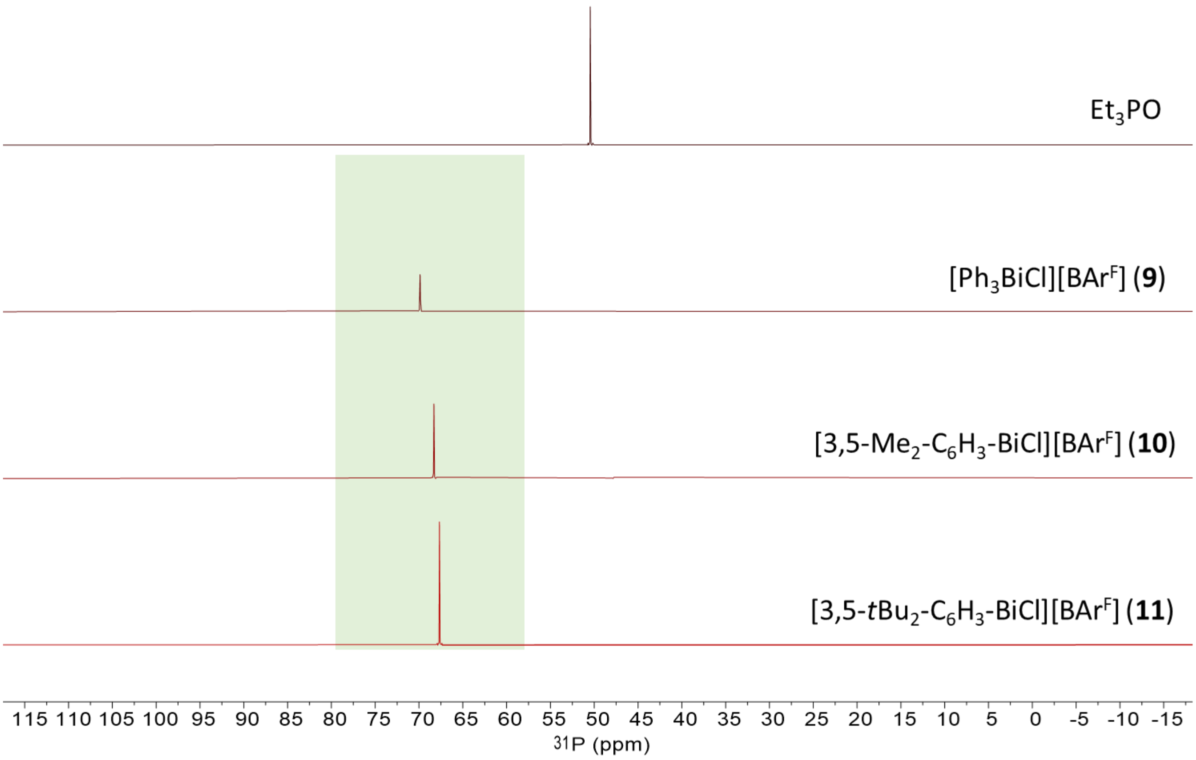

Figure S9. Stacked ^{31}P NMR spectra of the mixture of Lewis acid **9a-11a** with Et_3PO (121.5 MHz, 298K, CD_2Cl_2).

8. Polymerization of THF-*d8*

General procedure: A flame-dried *J*-Young tube was charged with the corresponding chlorotriarylbismuthonium salt (0.01 mmol) and dissolved in 0.5 mL anhydrous THF-d_8. ^{1}H NMR spectra of the reactions were measured by NMR spectroscopy upon mixing and after 12 h (see figure S10 – S13).

a) Reaction of **9** with THF:

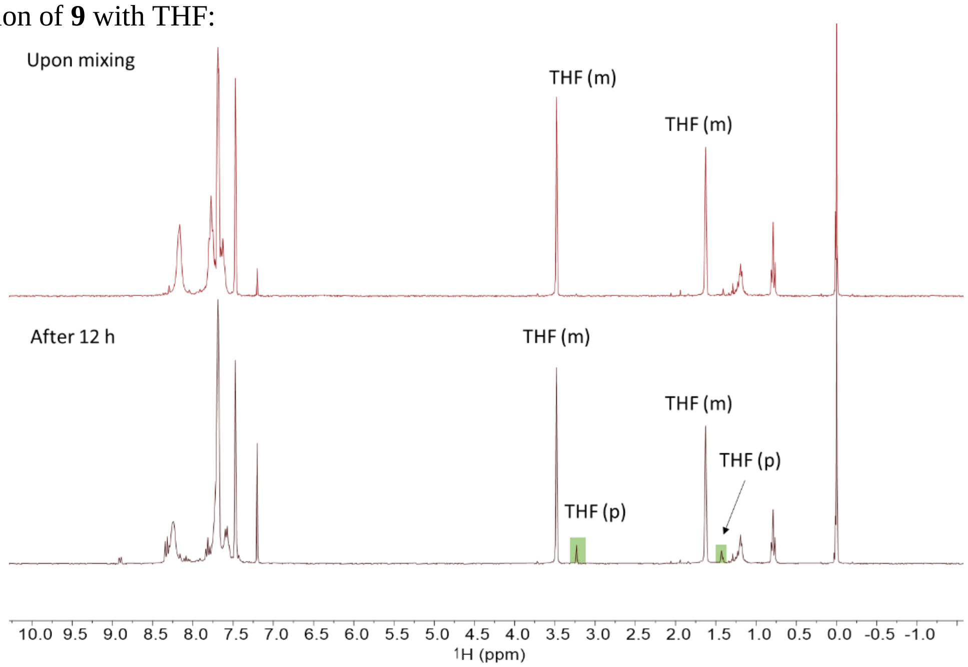

Figure S10. Stacked ^{1}H NMR spectra of the reaction mixture of **9** with THF-d_8 upon mixing and after 12 h (300 MHz, 298K). THF (m) = monomer, THF (p) = polymer.

b) Reaction of **10** with THF-d_8

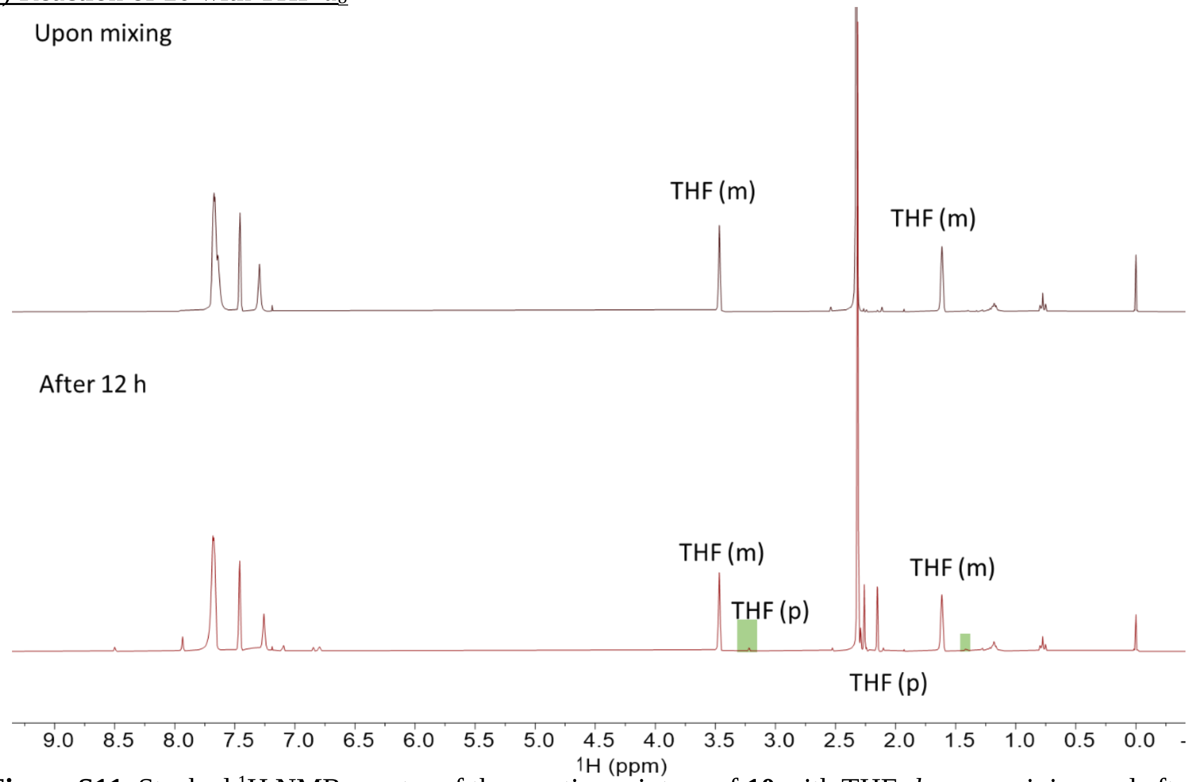

Figure S11. Stacked ^{1}H NMR spectra of the reaction mixture of **10** with THF-d_8 upon mixing and after 12 h (300 MHz, 298K). THF (m) = monomer, THF (p) = polymer.

c) Reaction of **12** with THF-d_8

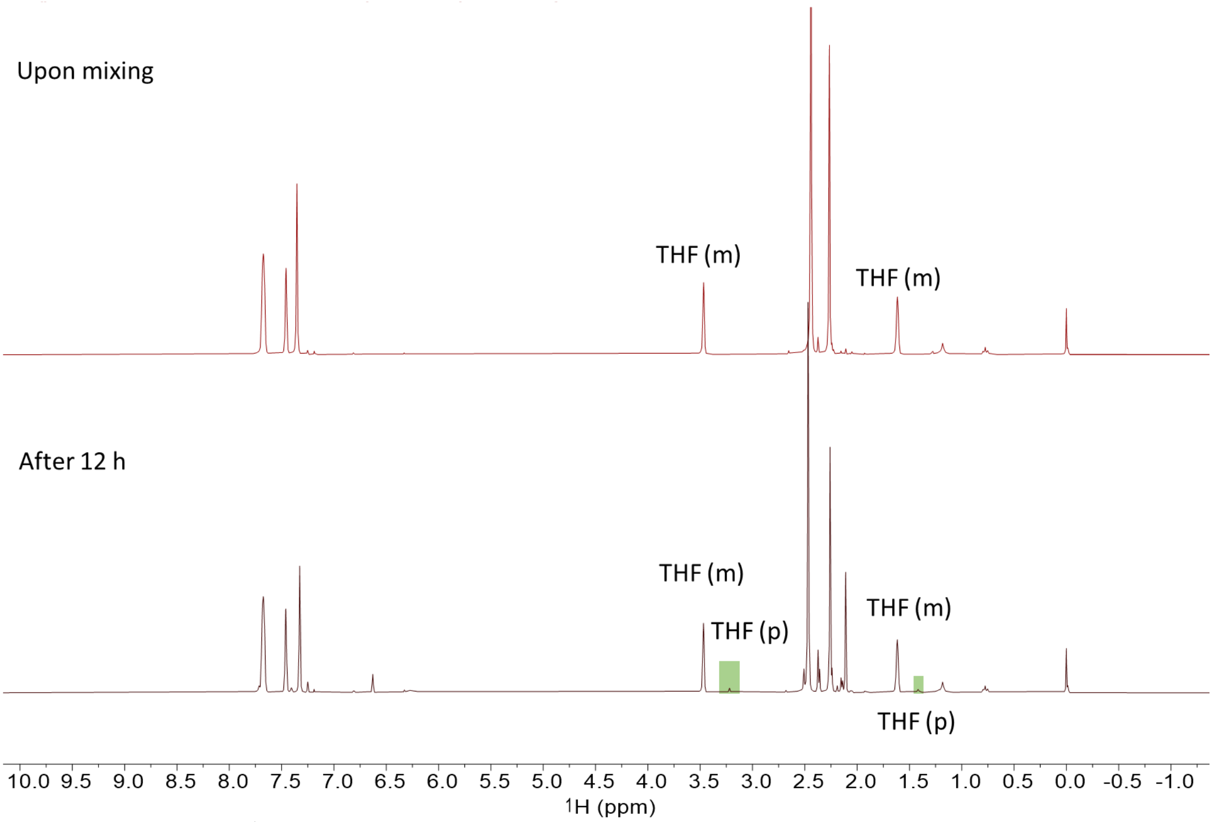

Figure S12. Stacked ^{1}H NMR spectra of the reaction mixture of **12** with THF-d_8 upon mixing and after 12 h (300 MHz, 298K). THF (m) = monomer, THF (p) = polymer.

d) Reaction of **9a** with THF-d_8

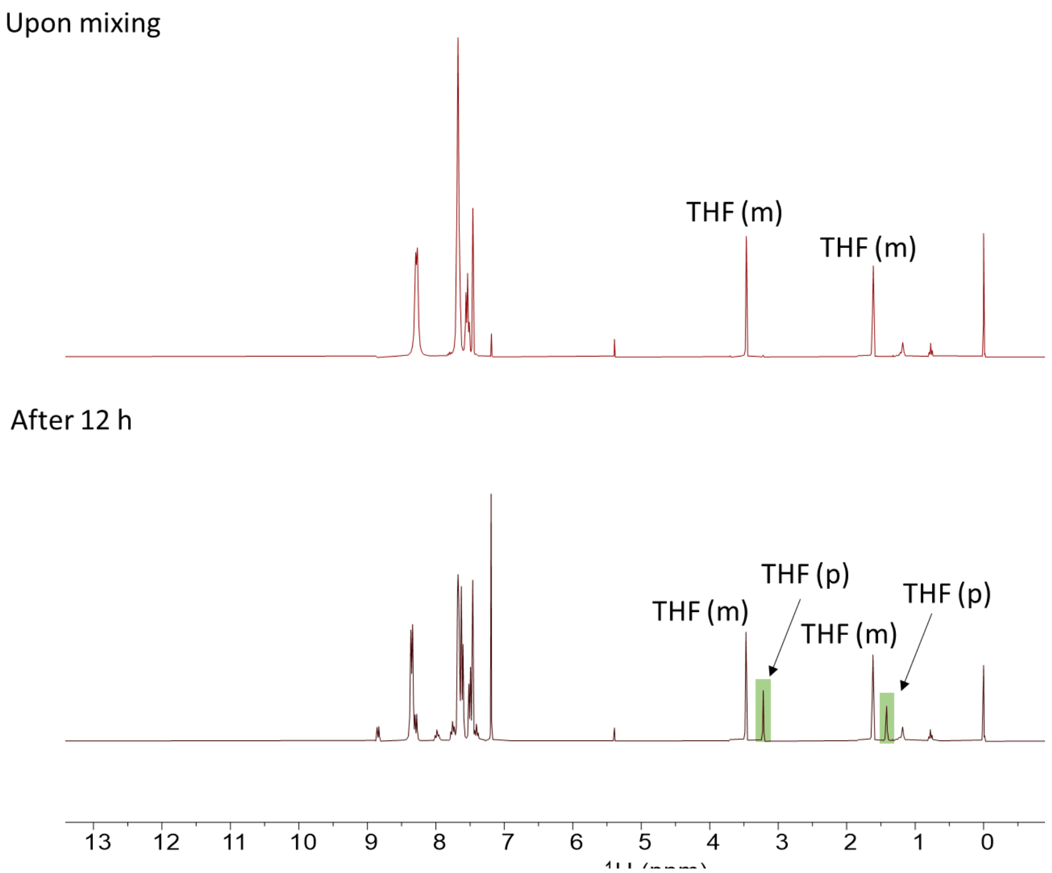

Figure S13. Stacked ^{1}H NMR spectra of the reaction mixture of **9**a with THF-d_8 upon mixing and after 12 h (300 MHz, 298K). THF (m) = monomer, THF (p) = polymer.

The reaction of chlorobismuthonium salts **11, 10a, 11a** with THF-d_8 did not show any reactivity towards polymerized THF-d_8.

9. X-ray Data

9. 1 Single crystal structure analysis of tris(3,5-diemthylphenyl)bimuth dichloride (6)

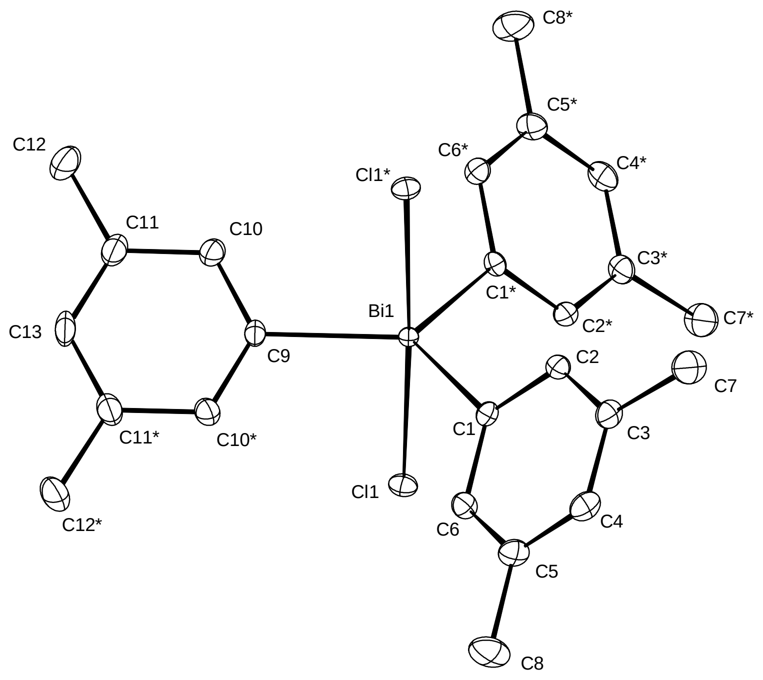

Figure S14: The solid state structure of **6**. H atoms have been removed for clarity.

X-ray Crystal Structure Analysis:

C_{24} H_{27} Bi Cl_2, M_r = 595.33 g mol^{-1}, colourless prism, crystal size 0.091 x 0.072 x 0.043 mm^3, orthorhombic, space group *Pbcn* [60], a = 15.9412(6) Å, b = 10.4632(4) Å, c = 13.6006(5) Å, V = 2268.53(15) Å^3,T = 100(2) K, Z = 4, D_{calc} = 1.743 g·cm^3, λ = 0.71073 Å, μ(*Mo-K*$_\alpha$) = 8.014 mm^{-1}, Gaussian correction ($T_{\min}$ = 0.60986, $T_{\max}$ = 0.81139), Bruker-AXS Kappa Mach3 with APEX-II detector and IµS micro focus Mo-anode X-ray source, 2.328 < θ < 34.958°, 85206 measured reflections, 4976 independent reflections, 3558 reflections with $I > 2\sigma(I)$, R_{int} = 0.0451. The structure was solved by *SHELXT* and refined by full-matrix least-squares (*SHELXL*) against F^2 to R_1 = 0.0163 [$I > 2\sigma(I)$], wR_2 = 0.0330 [all data] with 127 parameters and 0 restraints.

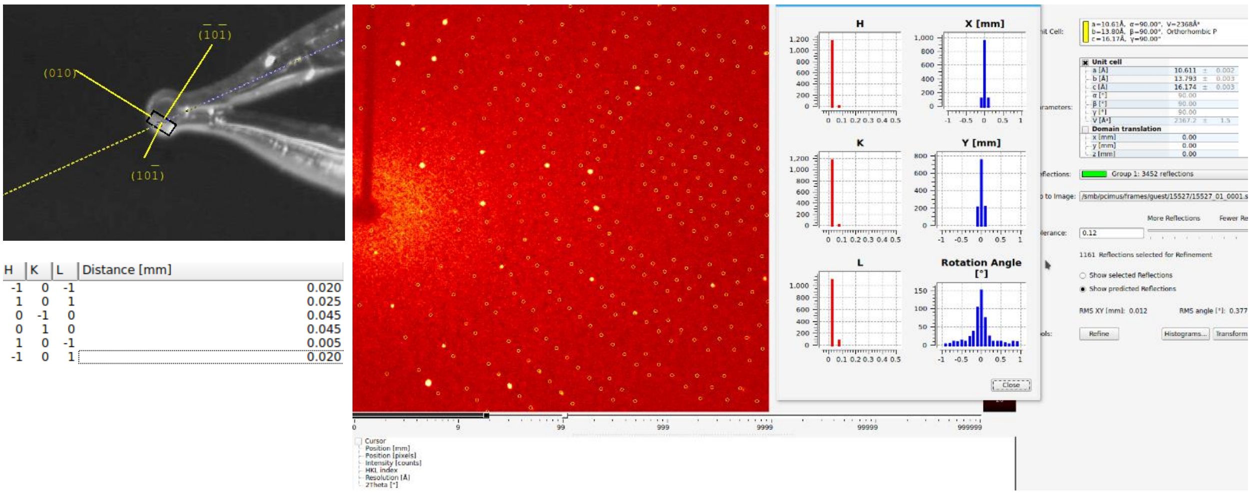

Figure S15: Crystal faces and unit cell determination/refinement of **6**.

INTENSITY STATISTICS FOR DATASET

Resolution	#Data	#Theory	%Complete	Redundancy	Mean I	Mean I/s	Rmerge	Rsigma
Inf - 2.80	83	83	100.0	24.83	129.09	92.99	0.0211	0.0061
2.80 - 1.78	196	196	100.0	30.30	118.30	93.41	0.0213	0.0060
1.78 - 1.39	276	276	100.0	31.94	77.23	82.47	0.0261	0.0067
1.39 - 1.20	286	286	100.0	32.09	64.70	71.46	0.0310	0.0075
1.20 - 1.09	263	263	100.0	30.01	55.01	63.75	0.0370	0.0091
1.09 - 1.00	302	302	100.0	22.29	49.45	49.42	0.0398	0.0115
1.00 - 0.94	270	270	100.0	17.95	40.91	38.05	0.0475	0.0152
0.94 - 0.89	286	286	100.0	15.60	32.96	31.24	0.0548	0.0187
0.89 - 0.85	271	271	100.0	14.51	32.21	28.27	0.0584	0.0209
0.85 - 0.81	322	322	100.0	13.83	31.35	26.38	0.0601	0.0223
0.81 - 0.79	201	201	100.0	13.05	22.24	19.60	0.0820	0.0306
0.79 - 0.76	319	319	100.0	12.96	26.17	21.77	0.0745	0.0279
0.76 - 0.74	252	252	100.0	12.15	23.34	19.14	0.0836	0.0330
0.74 - 0.72	263	263	100.0	12.04	20.90	17.39	0.0942	0.0367
0.72 - 0.70	308	308	100.0	11.54	22.11	17.43	0.0914	0.0368
0.70 - 0.68	344	344	100.0	11.19	17.43	13.97	0.1177	0.0470
0.68 - 0.67	183	183	100.0	10.73	18.19	14.46	0.1126	0.0470
0.67 - 0.65	404	404	100.0	10.39	16.13	12.38	0.1280	0.0545
0.65 - 0.64	219	219	100.0	10.25	14.40	11.14	0.1391	0.0605
0.64 - 0.63	239	239	100.0	9.73	14.31	10.82	0.1513	0.0644
0.63 - 0.62	232	242	95.9	6.95	14.53	8.75	0.1550	0.0912
0.72 - 0.62	1929	1939	99.5	10.22	16.90	12.90	0.1210	0.0541
Inf - 0.62	5519	5529	99.8	16.48	36.10	32.88	0.0437	0.0204

Complete .cif-data of the compound are available under the CCDC number CCDC-**2356069**.

Table S2: Crystal data and structure refinement of **6**.

Identification code	15527	
Empirical formula	$C_{24} H_{27} Bi Cl_2$	
Color	colourless	
Formula weight	595.33 g·mol^{-1}	
Temperature	100(2) K	
Wavelength	0.71073 Å	
Crystal system	Orthorhombic	
Space group	*Pbcn*, (no. 60)	
Unit cell dimensions	a = 15.9412(6) Å	α= 90°.
	b = 10.4632(4) Å	β= 90°.
	c = 13.6006(5) Å	γ = 90°.
Volume	2268.53(15) Å^3	
Z	4	
Density (calculated)	1.743 Mg·m^{-3}	
Absorption coefficient	8.014 mm^{-1}	
F(000)	1152 e	
Crystal size	0.091 x 0.072 x 0.043 mm^3	
θ range for data collection	2.328 to 34.958°.	
Index ranges	-25 ≤ h ≤ 25, -16≤ k ≤ 16, -21 ≤ l ≤ 21	
Reflections collected	85206	
Independent reflections	4976 [R_{int} = 0.0451]	
Reflections with I>2σ(I)	3558	
Completeness to θ = 25.242°	100.0 %	
Absorption correction	Gaussian	
Max. and min. transmission	0.83538 and 0.52512	
Refinement method	Full-matrix least-squares on F^2	
Data / restraints / parameters	4976 / 0 / 127	
Goodness-of-fit on F^2	1.013	
Final R indices [I>2σ(I)]	R_1 = 0.0163	wR^2 = 0.0291
R indices (all data)	R_1 = 0.0338	wR^2 = 0.0330
Extinction coefficient	n/a	
Largest diff. peak and hole	0.830 and -0.748 e·Å^{-3}	

Table S3: Bond lengths [Å] and angles [°] of **6**.

Bi(1)-Cl(1)	2.5898(4)	Bi(1)-Cl(1)#1	2.5898(4)
Bi(1)-C(1)	2.1980(15)	Bi(1)-C(1)#1	2.1980(15)
Bi(1)-C(9)	2.202(2)	C(1)-C(2)	1.387(2)
C(1)-C(6)	1.382(2)	C(2)-H(2)	0.9500
C(2)-C(3)	1.390(2)	C(3)-C(4)	1.395(3)
C(3)-C(7)	1.504(2)	C(4)-H(4)	0.9500
C(4)-C(5)	1.389(2)	C(5)-C(6)	1.399(2)
C(5)-C(8)	1.503(3)	C(6)-H(6)	0.9500
C(7)-H(7A)	0.9800	C(7)-H(7B)	0.9800
C(7)-H(7C)	0.9800	C(8)-H(8A)	0.9800
C(8)-H(8B)	0.9800	C(8)-H(8C)	0.9800
C(9)-C(10)#1	1.379(2)	C(9)-C(10)	1.379(2)
C(10)-H(10)	0.9500	C(10)-C(11)	1.402(3)
C(11)-C(12)	1.505(3)	C(11)-C(13)	1.394(2)
C(12)-H(12A)	0.9800	C(12)-H(12B)	0.9800
C(12)-H(12C)	0.9800	C(13)-H(13)	0.9500
Cl(1)#1-Bi(1)-Cl(1)	177.391(18)	C(1)-Bi(1)-Cl(1)	89.86(4)
C(1)#1-Bi(1)-Cl(1)#1	89.86(4)	C(1)-Bi(1)-Cl(1)#1	91.55(4)
C(1)#1-Bi(1)-Cl(1)	91.55(4)	C(1)#1-Bi(1)-C(1)	114.70(8)
C(1)-Bi(1)-C(9)	122.65(4)	C(1)#1-Bi(1)-C(9)	122.65(4)
C(9)-Bi(1)-Cl(1)	88.696(9)	C(9)-Bi(1)-Cl(1)#1	88.695(9)
C(2)-C(1)-Bi(1)	117.68(11)	C(6)-C(1)-Bi(1)	119.55(11)
C(6)-C(1)-C(2)	122.76(15)	C(1)-C(2)-H(2)	120.6
C(1)-C(2)-C(3)	118.84(15)	C(3)-C(2)-H(2)	120.6
C(2)-C(3)-C(4)	118.61(15)	C(2)-C(3)-C(7)	120.60(16)
C(4)-C(3)-C(7)	120.78(16)	C(3)-C(4)-H(4)	118.8
C(5)-C(4)-C(3)	122.46(16)	C(5)-C(4)-H(4)	118.8
C(4)-C(5)-C(6)	118.56(16)	C(4)-C(5)-C(8)	121.54(16)
C(6)-C(5)-C(8)	119.89(16)	C(1)-C(6)-C(5)	118.74(15)
C(1)-C(6)-H(6)	120.6	C(5)-C(6)-H(6)	120.6
C(3)-C(7)-H(7A)	109.5	C(3)-C(7)-H(7B)	109.5
C(3)-C(7)-H(7C)	109.5	H(7A)-C(7)-H(7B)	109.5
H(7A)-C(7)-H(7C)	109.5	H(7B)-C(7)-H(7C)	109.5
C(5)-C(8)-H(8A)	109.5	C(5)-C(8)-H(8B)	109.5
C(5)-C(8)-H(8C)	109.5	H(8A)-C(8)-H(8B)	109.5

H(8A)-C(8)-H(8C)	109.5	H(8B)-C(8)-H(8C)	109.5
C(10)#1-C(9)-Bi(1)	118.11(10)	C(10)-C(9)-Bi(1)	118.11(10)
C(10)#1-C(9)-C(10)	123.8(2)	C(9)-C(10)-H(10)	120.8
C(9)-C(10)-C(11)	118.34(17)	C(11)-C(10)-H(10)	120.8
C(10)-C(11)-C(12)	119.88(18)	C(13)-C(11)-C(10)	118.52(18)
C(13)-C(11)-C(12)	121.60(17)	C(11)-C(12)-H(12A)	109.5
C(11)-C(12)-H(12B)	109.5	C(11)-C(12)-H(12C)	109.5
H(12A)-C(12)-H(12B)	109.5	H(12A)-C(12)-H(12C)	109.5
H(12B)-C(12)-H(12C)	109.5	C(11)#1-C(13)-C(11)	122.5(2)
C(11)#1-C(13)-H(13)	118.8	C(11)-C(13)-H(13)	118.8

Symmetry transformations used to generate equivalent atoms:

#1 -x+1,y,-z+3/2

9.2 Single crystal structure analysis of chloro-tris(3,5-dimethylphenyl)bismuthonium tetrakis(3,5-bis(trifluoromethyl)phenyl)borate (10)

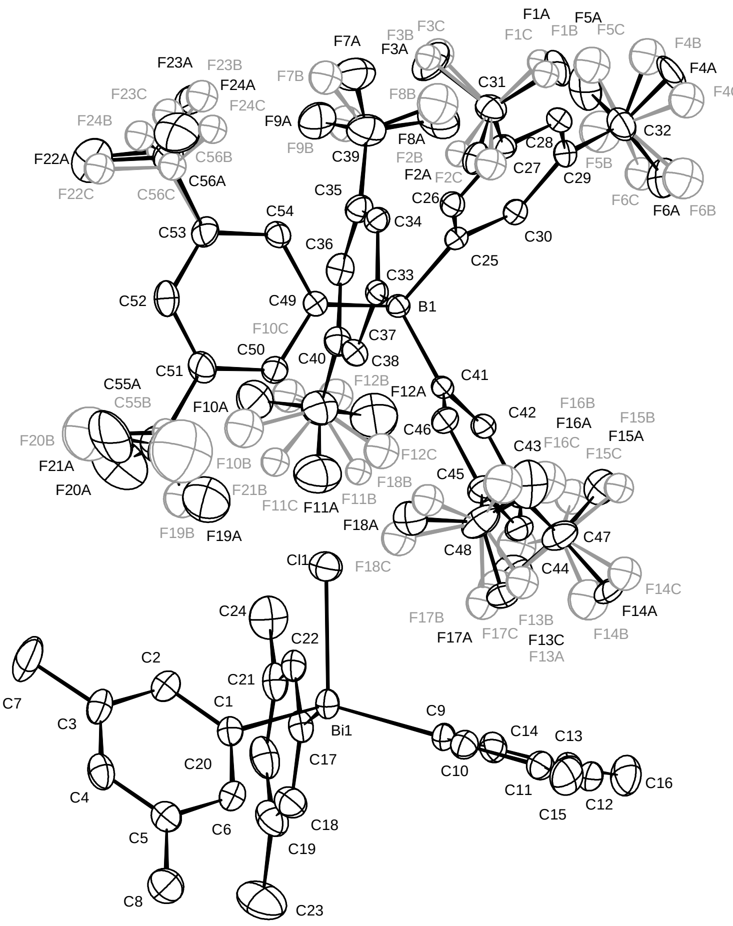

Figure S16: The solid state structure of **10**. H atoms have been removed for clarity and disordered parts shown in grey.

X-ray Crystal Structure Analysis:

C_{56} H_{39} B Bi Cl F_{24}, M_r = 1423.11 g mol^{-1}, colourless needle, crystal size 0.422 x 0.15 x 0.083 mm^3, monoclinic, space group $P2_1/c$ [14], a = 18.1228(8) Å, b = 15.8254(6) Å, c = 20.0417(9) Å, β = 106.109(2)°, V = 5522.3(4) Å^3,T = 150(2) K, Z = 4, D_{calc} = 1.712 g·cm^3, λ = 0.71073 Å, $\mu(Mo\text{-}K_{\alpha})$ = 3.360 mm^{-1}, Gaussian correction ($T_{\min}$ = 0.47600, $T_{\max}$ = 0.83841), Bruker-AXS Kappa Mach3 with APEX-II detector and IµS micro focus Mo-anode X-ray source, 1.169 < θ < 30.508°, 179506 measured reflections, 16869 independent reflections, 13456 reflections with $I > 2\sigma(I)$, R_{int} = 0.0466. The structure was solved by *SHELXT* and refined by full-matrix least-squares (*SHELXL*) against F^2 to R_1 = 0.0329 [$I > 2\sigma(I)$], wR_2 = 0.0809 [all data] with 934 parameters and 102 restraints.

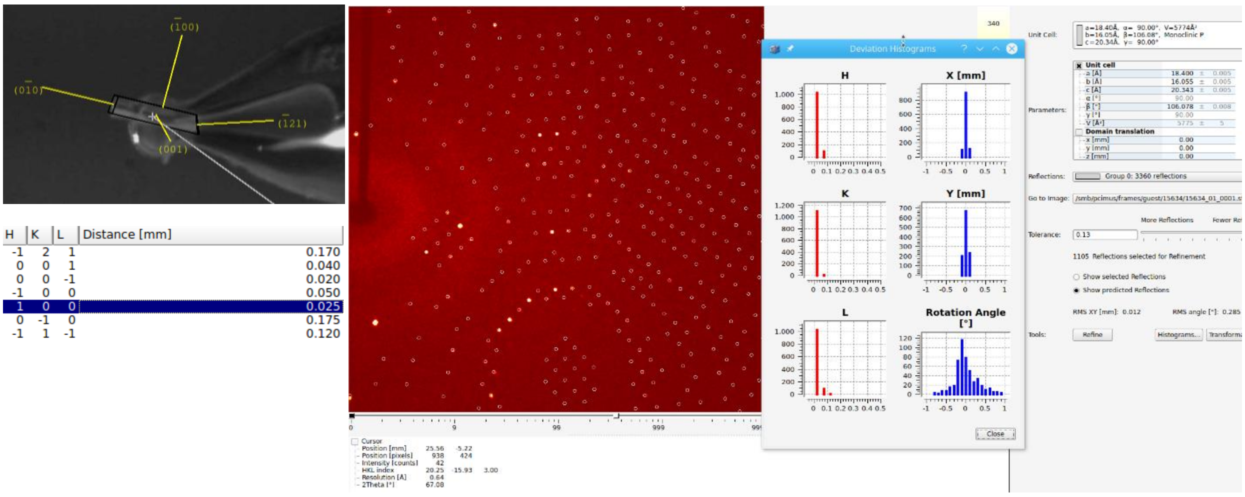

Figure S17: Crystal faces and unit cell determination/refinement of **10**.

INTENSITY STATISTICS FOR DATASET

Resolution	#Data	#Theory	%Complete	Redundancy	Mean I	Mean I/s	Rmerge	Rsigma
Inf - 2.82	288	288	100.0	16.49	63.66	86.74	0.0240	0.0082
2.82 - 1.87	677	677	100.0	18.05	42.34	78.37	0.0227	0.0086
1.87 - 1.48	958	958	100.0	18.24	26.53	66.88	0.0271	0.0096
1.48 - 1.28	997	997	100.0	18.18	18.88	55.58	0.0330	0.0112
1.28 - 1.16	991	991	100.0	17.59	14.89	48.71	0.0420	0.0135
1.16 - 1.08	936	936	100.0	15.74	12.69	41.45	0.0489	0.0165
1.08 - 1.01	1034	1034	100.0	12.13	10.82	32.23	0.0566	0.0215
1.01 - 0.96	943	943	100.0	10.33	9.49	26.49	0.0650	0.0264
0.96 - 0.92	932	932	100.0	9.07	7.44	20.67	0.0766	0.0341
0.92 - 0.88	1081	1081	100.0	8.15	6.78	18.35	0.0844	0.0398
0.88 - 0.85	964	964	100.0	7.74	5.57	15.30	0.1025	0.0491
0.85 - 0.82	1103	1103	100.0	7.43	4.98	13.59	0.1155	0.0570
0.82 - 0.80	812	812	100.0	7.22	4.35	11.76	0.1281	0.0664
0.80 - 0.78	937	937	100.0	7.01	4.03	11.00	0.1440	0.0738
0.78 - 0.76	1006	1006	100.0	6.81	3.69	9.83	0.1589	0.0829
0.76 - 0.74	1133	1133	100.0	6.63	3.36	8.80	0.1740	0.0944
0.74 - 0.73	596	596	100.0	6.39	2.87	7.51	0.1969	0.1120
0.73 - 0.71	1329	1329	100.0	6.28	2.84	7.19	0.2098	0.1181
0.71 - 0.70	720	720	100.0	6.14	2.45	6.22	0.2297	0.1383
0.70 - 0.68	1726	1919	89.9	4.50	2.27	5.05	0.2570	0.1908
0.78 - 0.68	6510	6703	97.1	5.90	2.87	7.23	0.1997	0.1229
Inf - 0.68	19163	19356	99.0	9.92	9.87	24.98	0.0470	0.0298

Complete .cif-data of the compound are available under the CCDC number CCDC-**2356071**.

A resolution cut-off (SHEL 999 0.70) was applied to the data set to exclude the poorly determined reflections at high diffraction angles. The terminal –CF_3 groups show strong rotational disorders over two or more positions. The ISOR instruction was applied to several F atoms in the main part of the -CF_3 rotational disorders. Atomic occupancies have been refined with fixed values and isotropic displacement parameters are applied to atoms in the minor parts of the disorder.

Table S4: Crystal data and structure refinement of **10**.

Identification code	15634	
Empirical formula	$C_{56} H_{39} B Bi Cl F_{24}$	
Color	colourless	
Formula weight	1423.11 g·mol^{-1}	
Temperature	150(2) K	
Wavelength	0.71073 Å	
Crystal system	Monoclinic	
Space group	$P2_1/c$, (no. 14)	
Unit cell dimensions	a = 18.1228(8) Å	α= 90°.
	b = 15.8254(6) Å	β= 106.109(2)°.
	c = 20.0417(9) Å	γ = 90°.
Volume	5522.3(4) Å^3	
Z	4	
Density (calculated)	1.712 Mg·m^{-3}	
Absorption coefficient	3.360 mm^{-1}	
F(000)	2784 e	
Crystal size	0.422 x 0.15 x 0.083 mm^3	
θ range for data collection	1.169 to 30.508°.	
Index ranges	-25 ≤ h ≤ 25, -22≤ k ≤ 22, -28 ≤ l ≤ 28	
Reflections collected	179506	
Independent reflections	16869 [R_{int} = 0.0466]	
Reflections with I>2σ(I)	13456	
Completeness to θ = 25.242°	100.0 %	
Absorption correction	Gaussian	
Max. and min. transmission	0.83841 and 0.47600	
Refinement method	Full-matrix least-squares on F^2	
Data / restraints / parameters	16869 / 102 / 934	
Goodness-of-fit on F^2	1.027	
Final R indices [I>2σ(I)]	R_1 = 0.0329	wR^2 = 0.0741
R indices (all data)	R_1 = 0.0486	wR^2 = 0.0809
Extinction coefficient	n/a	
Largest diff. peak and hole	1.721 and -1.012 e·Å^{-3}	

Table S5: Bond lengths [Å] and angles [°] of **10**.

Bi(1)-Cl(1)	2.4028(8)	Bi(1)-C(1)	2.185(3)
Bi(1)-C(9)	2.180(3)	Bi(1)-C(17)	2.189(3)
C(1)-C(2)	1.382(4)	C(1)-C(6)	1.384(4)
C(2)-H(2)	0.9500	C(2)-C(3)	1.390(4)
C(3)-C(4)	1.395(5)	C(3)-C(7)	1.513(5)
C(4)-H(4)	0.9500	C(4)-C(5)	1.389(4)
C(5)-C(6)	1.396(4)	C(5)-C(8)	1.498(4)
C(6)-H(6)	0.9500	C(7)-H(7A)	0.9800
C(7)-H(7B)	0.9800	C(7)-H(7C)	0.9800
C(8)-H(8A)	0.9800	C(8)-H(8B)	0.9800
C(8)-H(8C)	0.9800	C(9)-C(10)	1.381(4)
C(9)-C(14)	1.383(4)	C(10)-H(10)	0.9500
C(10)-C(11)	1.399(4)	C(11)-C(12)	1.389(5)
C(11)-C(15)	1.501(4)	C(12)-H(12)	0.9500
C(12)-C(13)	1.389(5)	C(13)-C(14)	1.389(4)
C(13)-C(16)	1.507(5)	C(14)-H(14)	0.9500
C(15)-H(15A)	0.9800	C(15)-H(15B)	0.9800
C(15)-H(15C)	0.9800	C(16)-H(16A)	0.9800
C(16)-H(16B)	0.9800	C(16)-H(16C)	0.9800
C(17)-C(18)	1.387(4)	C(17)-C(22)	1.380(4)
C(18)-H(18)	0.9500	C(18)-C(19)	1.392(5)
C(19)-C(20)	1.387(5)	C(19)-C(23)	1.512(5)
C(20)-H(20)	0.9500	C(20)-C(21)	1.387(5)
C(21)-C(22)	1.395(4)	C(21)-C(24)	1.503(5)
C(22)-H(22)	0.9500	C(23)-H(23A)	0.9800
C(23)-H(23B)	0.9800	C(23)-H(23C)	0.9800
C(24)-H(24A)	0.9800	C(24)-H(24B)	0.9800
C(24)-H(24C)	0.9800	C(25)-C(26)	1.398(4)
C(25)-C(30)	1.398(4)	C(25)-B(1)	1.645(4)
C(26)-H(26)	0.9500	C(26)-C(27)	1.397(4)
C(27)-C(28)	1.382(4)	C(27)-C(31)	1.497(4)
C(28)-H(28)	0.9500	C(28)-C(29)	1.387(4)
C(29)-C(30)	1.392(4)	C(29)-C(32)	1.488(4)
C(30)-H(30)	0.9500	C(31)-F(1A)	1.408(8)
C(31)-F(2A)	1.339(8)	C(31)-F(3A)	1.332(17)
C(31)-F(1B)	1.26(4)	C(31)-F(2B)	1.35(3)

C(31)-F(3B)	1.32(6)	C(31)-F(1C)	1.23(3)
C(31)-F(2C)	1.40(4)	C(31)-F(3C)	1.41(4)
C(32)-F(4A)	1.344(5)	C(32)-F(5A)	1.371(5)
C(32)-F(6A)	1.320(5)	C(32)-F(4B)	1.295(16)
C(32)-F(5B)	1.292(14)	C(32)-F(6B)	1.430(18)
C(32)-F(4C)	1.333(17)	C(32)-F(5C)	1.342(17)
C(32)-F(6C)	1.359(16)	C(33)-C(34)	1.399(4)
C(33)-C(38)	1.398(4)	C(33)-B(1)	1.648(4)
C(34)-H(34)	0.9500	C(34)-C(35)	1.390(4)
C(35)-C(36)	1.376(4)	C(35)-C(39)	1.496(4)
C(36)-H(36)	0.9500	C(36)-C(37)	1.380(4)
C(37)-C(38)	1.390(4)	C(37)-C(40)	1.487(4)
C(38)-H(38)	0.9500	C(39)-F(7A)	1.283(6)
C(39)-F(8A)	1.361(6)	C(39)-F(9A)	1.366(5)
C(39)-F(7B)	1.430(7)	C(39)-F(8B)	1.332(9)
C(39)-F(9B)	1.307(8)	C(40)-F(10A)	1.334(5)
C(40)-F(11A)	1.307(6)	C(40)-F(12A)	1.367(6)
C(40)-F(10B)	1.369(12)	C(40)-F(11B)	1.341(9)
C(40)-F(12B)	1.367(11)	C(40)-F(10C)	1.265(11)
C(40)-F(11C)	1.430(10)	C(40)-F(12C)	1.343(11)
C(41)-C(42)	1.395(4)	C(41)-C(46)	1.405(4)
C(41)-B(1)	1.642(4)	C(42)-H(42)	0.9500
C(42)-C(43)	1.394(4)	C(43)-C(44)	1.383(4)
C(43)-C(47)	1.493(4)	C(44)-H(44)	0.9500
C(44)-C(45)	1.388(4)	C(45)-C(46)	1.387(4)
C(45)-C(48)	1.497(4)	C(46)-H(46)	0.9500
C(47)-F(13A)	1.300(6)	C(47)-F(14A)	1.356(6)
C(47)-F(15A)	1.342(5)	C(47)-F(13B)	1.310(15)
C(47)-F(14B)	1.244(17)	C(47)-F(15B)	1.397(13)
C(47)-F(13C)	1.561(17)	C(47)-F(14C)	1.396(15)
C(47)-F(15C)	1.334(12)	C(48)-F(16A)	1.341(9)
C(48)-F(17A)	1.345(6)	C(48)-F(18A)	1.358(6)
C(48)-F(16B)	1.49(4)	C(48)-F(17B)	1.42(2)
C(48)-F(18B)	1.250(17)	C(48)-F(16C)	1.18(2)
C(48)-F(17C)	1.29(2)	C(48)-F(18C)	1.488(17)
C(49)-C(50)	1.394(4)	C(49)-C(54)	1.400(4)
C(49)-B(1)	1.644(4)	C(50)-H(50)	0.9500
C(50)-C(51)	1.388(4)	C(51)-C(52)	1.379(5)

C(51)-C(55A)	1.560(7)	C(51)-C(55B)	1.440(10)
C(52)-H(52)	0.9500	C(52)-C(53)	1.387(4)
C(53)-C(54)	1.389(4)	C(53)-C(56A)	1.491(9)
C(53)-C(56B)	1.646(18)	C(53)-C(56C)	1.41(2)
C(54)-H(54)	0.9500	F(19A)-C(55A)	1.339(7)
F(20A)-C(55A)	1.208(8)	F(21A)-C(55A)	1.384(7)
F(22A)-C(56A)	1.348(10)	F(23A)-C(56A)	1.389(12)
F(24A)-C(56A)	1.309(11)	F(19B)-C(55B)	1.497(13)
F(20B)-C(55B)	1.350(17)	F(21B)-C(55B)	1.184(18)
F(22B)-C(56B)	1.297(19)	F(23B)-C(56B)	1.26(3)
F(24B)-C(56B)	1.38(2)	F(22C)-C(56C)	1.32(2)
F(23C)-C(56C)	1.39(2)	F(24C)-C(56C)	1.35(2)
C(1)-Bi(1)-Cl(1)	101.22(8)	C(1)-Bi(1)-C(17)	111.32(10)
C(9)-Bi(1)-Cl(1)	105.68(7)	C(9)-Bi(1)-C(1)	121.63(11)
C(9)-Bi(1)-C(17)	112.55(11)	C(17)-Bi(1)-Cl(1)	101.47(8)
C(2)-C(1)-Bi(1)	116.4(2)	C(2)-C(1)-C(6)	123.4(3)
C(6)-C(1)-Bi(1)	119.8(2)	C(1)-C(2)-H(2)	120.8
C(1)-C(2)-C(3)	118.4(3)	C(3)-C(2)-H(2)	120.8
C(2)-C(3)-C(4)	118.7(3)	C(2)-C(3)-C(7)	119.9(3)
C(4)-C(3)-C(7)	121.4(3)	C(3)-C(4)-H(4)	118.7
C(5)-C(4)-C(3)	122.6(3)	C(5)-C(4)-H(4)	118.7
C(4)-C(5)-C(6)	118.4(3)	C(4)-C(5)-C(8)	121.0(3)
C(6)-C(5)-C(8)	120.6(3)	C(1)-C(6)-C(5)	118.5(3)
C(1)-C(6)-H(6)	120.7	C(5)-C(6)-H(6)	120.7
C(3)-C(7)-H(7A)	109.5	C(3)-C(7)-H(7B)	109.5
C(3)-C(7)-H(7C)	109.5	H(7A)-C(7)-H(7B)	109.5
H(7A)-C(7)-H(7C)	109.5	H(7B)-C(7)-H(7C)	109.5
C(5)-C(8)-H(8A)	109.5	C(5)-C(8)-H(8B)	109.5
C(5)-C(8)-H(8C)	109.5	H(8A)-C(8)-H(8B)	109.5
H(8A)-C(8)-H(8C)	109.5	H(8B)-C(8)-H(8C)	109.5
C(10)-C(9)-Bi(1)	120.3(2)	C(10)-C(9)-C(14)	124.2(3)
C(14)-C(9)-Bi(1)	115.5(2)	C(9)-C(10)-H(10)	121.3
C(9)-C(10)-C(11)	117.4(3)	C(11)-C(10)-H(10)	121.3
C(10)-C(11)-C(15)	120.3(3)	C(12)-C(11)-C(10)	118.8(3)
C(12)-C(11)-C(15)	120.9(3)	C(11)-C(12)-H(12)	118.5
C(11)-C(12)-C(13)	123.0(3)	C(13)-C(12)-H(12)	118.5
C(12)-C(13)-C(14)	118.3(3)	C(12)-C(13)-C(16)	121.2(3)

C(14)-C(13)-C(16)	120.4(3)	C(9)-C(14)-C(13)	118.3(3)
C(9)-C(14)-H(14)	120.9	C(13)-C(14)-H(14)	120.9
C(11)-C(15)-H(15A)	109.5	C(11)-C(15)-H(15B)	109.5
C(11)-C(15)-H(15C)	109.5	H(15A)-C(15)-H(15B)	109.5
H(15A)-C(15)-H(15C)	109.5	H(15B)-C(15)-H(15C)	109.5
C(13)-C(16)-H(16A)	109.5	C(13)-C(16)-H(16B)	109.5
C(13)-C(16)-H(16C)	109.5	H(16A)-C(16)-H(16B)	109.5
H(16A)-C(16)-H(16C)	109.5	H(16B)-C(16)-H(16C)	109.5
C(18)-C(17)-Bi(1)	117.0(2)	C(22)-C(17)-Bi(1)	119.4(2)
C(22)-C(17)-C(18)	123.6(3)	C(17)-C(18)-H(18)	120.8
C(17)-C(18)-C(19)	118.3(3)	C(19)-C(18)-H(18)	120.8
C(18)-C(19)-C(23)	120.3(4)	C(20)-C(19)-C(18)	118.2(3)
C(20)-C(19)-C(23)	121.5(3)	C(19)-C(20)-H(20)	118.4
C(19)-C(20)-C(21)	123.3(3)	C(21)-C(20)-H(20)	118.4
C(20)-C(21)-C(22)	118.5(3)	C(20)-C(21)-C(24)	121.7(3)
C(22)-C(21)-C(24)	119.8(3)	C(17)-C(22)-C(21)	118.1(3)
C(17)-C(22)-H(22)	121.0	C(21)-C(22)-H(22)	121.0
C(19)-C(23)-H(23A)	109.5	C(19)-C(23)-H(23B)	109.5
C(19)-C(23)-H(23C)	109.5	H(23A)-C(23)-H(23B)	109.5
H(23A)-C(23)-H(23C)	109.5	H(23B)-C(23)-H(23C)	109.5
C(21)-C(24)-H(24A)	109.5	C(21)-C(24)-H(24B)	109.5
C(21)-C(24)-H(24C)	109.5	H(24A)-C(24)-H(24B)	109.5
H(24A)-C(24)-H(24C)	109.5	H(24B)-C(24)-H(24C)	109.5
C(26)-C(25)-C(30)	115.7(2)	C(26)-C(25)-B(1)	124.0(2)
C(30)-C(25)-B(1)	120.4(2)	C(25)-C(26)-H(26)	119.1
C(27)-C(26)-C(25)	121.8(2)	C(27)-C(26)-H(26)	119.1
C(26)-C(27)-C(31)	118.7(2)	C(28)-C(27)-C(26)	121.3(2)
C(28)-C(27)-C(31)	119.8(2)	C(27)-C(28)-H(28)	121.0
C(27)-C(28)-C(29)	118.0(2)	C(29)-C(28)-H(28)	121.0
C(28)-C(29)-C(30)	120.4(2)	C(28)-C(29)-C(32)	120.4(3)
C(30)-C(29)-C(32)	119.1(3)	C(25)-C(30)-H(30)	118.6
C(29)-C(30)-C(25)	122.8(2)	C(29)-C(30)-H(30)	118.6
F(1A)-C(31)-C(27)	109.8(5)	F(2A)-C(31)-C(27)	112.7(4)
F(2A)-C(31)-F(1A)	107.0(11)	F(3A)-C(31)-C(27)	111.8(8)
F(3A)-C(31)-F(1A)	110.3(12)	F(3A)-C(31)-F(2A)	105.1(9)
F(1B)-C(31)-C(27)	120.7(11)	F(1B)-C(31)-F(2B)	106.1(16)
F(1B)-C(31)-F(3B)	102(3)	F(2B)-C(31)-C(27)	114.0(12)
F(3B)-C(31)-C(27)	111(2)	F(3B)-C(31)-F(2B)	100(2)

F(1C)-C(31)-C(27)	116.7(16)	F(1C)-C(31)-F(2C)	105(2)
F(1C)-C(31)-F(3C)	96.8(18)	F(2C)-C(31)-C(27)	109.3(14)
F(2C)-C(31)-F(3C)	115.7(18)	F(3C)-C(31)-C(27)	113.1(13)
F(4A)-C(32)-C(29)	113.9(3)	F(4A)-C(32)-F(5A)	106.1(4)
F(5A)-C(32)-C(29)	111.2(3)	F(6A)-C(32)-C(29)	114.0(3)
F(6A)-C(32)-F(4A)	105.7(4)	F(6A)-C(32)-F(5A)	105.3(4)
F(4B)-C(32)-C(29)	113.4(7)	F(4B)-C(32)-F(6B)	109.9(9)
F(5B)-C(32)-C(29)	114.3(7)	F(5B)-C(32)-F(4B)	113.7(10)
F(5B)-C(32)-F(6B)	98.6(9)	F(6B)-C(32)-C(29)	105.4(7)
F(4C)-C(32)-C(29)	112.7(7)	F(4C)-C(32)-F(5C)	107.6(9)
F(4C)-C(32)-F(6C)	107.0(9)	F(5C)-C(32)-C(29)	110.6(8)
F(5C)-C(32)-F(6C)	102.5(9)	F(6C)-C(32)-C(29)	115.7(7)
C(34)-C(33)-B(1)	122.5(2)	C(38)-C(33)-C(34)	115.4(2)
C(38)-C(33)-B(1)	122.1(2)	C(33)-C(34)-H(34)	118.8
C(35)-C(34)-C(33)	122.4(3)	C(35)-C(34)-H(34)	118.8
C(34)-C(35)-C(39)	119.1(3)	C(36)-C(35)-C(34)	120.9(3)
C(36)-C(35)-C(39)	119.8(3)	C(35)-C(36)-H(36)	121.0
C(35)-C(36)-C(37)	117.9(3)	C(37)-C(36)-H(36)	121.0
C(36)-C(37)-C(38)	121.2(3)	C(36)-C(37)-C(40)	118.8(3)
C(38)-C(37)-C(40)	119.9(3)	C(33)-C(38)-H(38)	119.0
C(37)-C(38)-C(33)	122.0(3)	C(37)-C(38)-H(38)	119.0
F(7A)-C(39)-C(35)	116.0(3)	F(7A)-C(39)-F(8A)	105.0(4)
F(7A)-C(39)-F(9A)	106.2(4)	F(8A)-C(39)-C(35)	110.4(3)
F(8A)-C(39)-F(9A)	107.7(4)	F(9A)-C(39)-C(35)	111.0(3)
F(7B)-C(39)-C(35)	108.7(4)	F(8B)-C(39)-C(35)	113.3(4)
F(8B)-C(39)-F(7B)	103.9(5)	F(9B)-C(39)-C(35)	115.5(4)
F(9B)-C(39)-F(7B)	110.8(5)	F(9B)-C(39)-F(8B)	103.9(5)
F(10A)-C(40)-C(37)	113.3(3)	F(10A)-C(40)-F(12A)	103.4(4)
F(11A)-C(40)-C(37)	115.1(3)	F(11A)-C(40)-F(10A)	108.5(4)
F(11A)-C(40)-F(12A)	104.9(4)	F(12A)-C(40)-C(37)	110.8(3)
F(10B)-C(40)-C(37)	112.0(6)	F(11B)-C(40)-C(37)	114.2(4)
F(11B)-C(40)-F(10B)	104.7(7)	F(11B)-C(40)-F(12B)	104.3(7)
F(12B)-C(40)-C(37)	113.5(5)	F(12B)-C(40)-F(10B)	107.3(8)
F(10C)-C(40)-C(37)	118.3(6)	F(10C)-C(40)-F(11C)	109.6(7)
F(10C)-C(40)-F(12C)	106.1(8)	F(11C)-C(40)-C(37)	109.5(4)
F(12C)-C(40)-C(37)	112.2(5)	F(12C)-C(40)-F(11C)	99.3(7)
C(42)-C(41)-C(46)	115.3(2)	C(42)-C(41)-B(1)	123.9(2)
C(46)-C(41)-B(1)	120.8(2)	C(41)-C(42)-H(42)	118.8

C(43)-C(42)-C(41)	122.4(3)	C(43)-C(42)-H(42)	118.8
C(42)-C(43)-C(47)	119.2(3)	C(44)-C(43)-C(42)	120.9(3)
C(44)-C(43)-C(47)	119.8(3)	C(43)-C(44)-H(44)	121.0
C(43)-C(44)-C(45)	117.9(3)	C(45)-C(44)-H(44)	121.0
C(44)-C(45)-C(48)	119.7(3)	C(46)-C(45)-C(44)	120.7(3)
C(46)-C(45)-C(48)	119.6(3)	C(41)-C(46)-H(46)	118.7
C(45)-C(46)-C(41)	122.6(3)	C(45)-C(46)-H(46)	118.7
C(43)-C(47)-F(13C)	104.8(7)	F(13A)-C(47)-C(43)	113.1(3)
F(13A)-C(47)-F(14A)	103.5(4)	F(13A)-C(47)-F(15A)	107.7(4)
F(14A)-C(47)-C(43)	112.8(3)	F(15A)-C(47)-C(43)	112.1(3)
F(15A)-C(47)-F(14A)	107.0(4)	F(13B)-C(47)-C(43)	113.7(7)
F(13B)-C(47)-F(15B)	104.5(8)	F(14B)-C(47)-C(43)	118.6(8)
F(14B)-C(47)-F(13B)	104.2(10)	F(14B)-C(47)-F(15B)	107.3(9)
F(15B)-C(47)-C(43)	107.5(6)	F(14C)-C(47)-C(43)	109.7(7)
F(14C)-C(47)-F(13C)	114.2(9)	F(15C)-C(47)-C(43)	118.1(6)
F(15C)-C(47)-F(13C)	94.9(8)	F(15C)-C(47)-F(14C)	114.0(8)
F(16A)-C(48)-C(45)	112.8(5)	F(16A)-C(48)-F(17A)	105.4(5)
F(16A)-C(48)-F(18A)	105.8(6)	F(17A)-C(48)-C(45)	113.2(4)
F(17A)-C(48)-F(18A)	106.4(4)	F(18A)-C(48)-C(45)	112.6(3)
F(16B)-C(48)-C(45)	105.3(14)	F(17B)-C(48)-C(45)	109.2(9)
F(17B)-C(48)-F(16B)	117.6(17)	F(18B)-C(48)-C(45)	116.8(8)
F(18B)-C(48)-F(16B)	97.6(16)	F(18B)-C(48)-F(17B)	110.3(11)
F(16C)-C(48)-C(45)	119.6(13)	F(16C)-C(48)-F(17C)	101.1(14)
F(16C)-C(48)-F(18C)	106.7(12)	F(17C)-C(48)-C(45)	114.0(10)
F(17C)-C(48)-F(18C)	110.8(10)	F(18C)-C(48)-C(45)	104.5(7)
C(50)-C(49)-C(54)	115.7(2)	C(50)-C(49)-B(1)	122.6(2)
C(54)-C(49)-B(1)	121.4(2)	C(49)-C(50)-H(50)	118.9
C(51)-C(50)-C(49)	122.1(3)	C(51)-C(50)-H(50)	118.9
C(50)-C(51)-C(55A)	117.1(3)	C(50)-C(51)-C(55B)	123.6(5)
C(52)-C(51)-C(50)	121.4(3)	C(52)-C(51)-C(55A)	120.9(3)
C(52)-C(51)-C(55B)	113.7(5)	C(51)-C(52)-H(52)	121.2
C(51)-C(52)-C(53)	117.6(3)	C(53)-C(52)-H(52)	121.2
C(52)-C(53)-C(54)	120.9(3)	C(52)-C(53)-C(56A)	119.1(4)
C(52)-C(53)-C(56B)	119.6(6)	C(52)-C(53)-C(56C)	112.5(8)
C(54)-C(53)-C(56A)	119.9(4)	C(54)-C(53)-C(56B)	118.0(7)
C(54)-C(53)-C(56C)	124.6(8)	C(49)-C(54)-H(54)	118.9
C(53)-C(54)-C(49)	122.2(3)	C(53)-C(54)-H(54)	118.9
C(25)-B(1)-C(33)	110.0(2)	C(41)-B(1)-C(25)	106.3(2)

C(41)-B(1)-C(33)	112.2(2)	C(41)-B(1)-C(49)	111.3(2)
C(49)-B(1)-C(25)	112.3(2)	C(49)-B(1)-C(33)	104.8(2)
F(19A)-C(55A)-C(51)	109.6(5)	F(19A)-C(55A)-F(21A)	102.5(5)
F(20A)-C(55A)-C(51)	116.6(5)	F(20A)-C(55A)-F(19A)	112.4(6)
F(20A)-C(55A)-F(21A)	108.7(6)	F(21A)-C(55A)-C(51)	106.1(5)
F(22A)-C(56A)-C(53)	110.9(6)	F(22A)-C(56A)-F(23A)	103.1(8)
F(23A)-C(56A)-C(53)	113.1(6)	F(24A)-C(56A)-C(53)	113.6(8)
F(24A)-C(56A)-F(22A)	105.7(7)	F(24A)-C(56A)-F(23A)	109.6(7)
C(51)-C(55B)-F(19B)	106.2(8)	F(20B)-C(55B)-C(51)	112.8(9)
F(20B)-C(55B)-F(19B)	94.2(9)	F(21B)-C(55B)-C(51)	121.3(12)
F(21B)-C(55B)-F(19B)	106.5(11)	F(21B)-C(55B)-F(20B)	111.7(13)
F(22B)-C(56B)-C(53)	113.7(12)	F(22B)-C(56B)-F(24B)	102.9(12)
F(23B)-C(56B)-C(53)	110.8(14)	F(23B)-C(56B)-F(22B)	121.4(18)
F(23B)-C(56B)-F(24B)	101.9(14)	F(24B)-C(56B)-C(53)	103.3(13)
F(22C)-C(56C)-C(53)	118.5(15)	F(22C)-C(56C)-F(23C)	99.7(15)
F(22C)-C(56C)-F(24C)	109.4(16)	F(23C)-C(56C)-C(53)	103.8(15)
F(24C)-C(56C)-C(53)	118.1(16)	F(24C)-C(56C)-F(23C)	104.2(14)

9.3 Single crystal structure analysis of ((chloromesityl-λ^5-bismuth)oxy)pyridinium tetrakis(3,5-bis(trifluoromethyl)phenyl)borate (13)

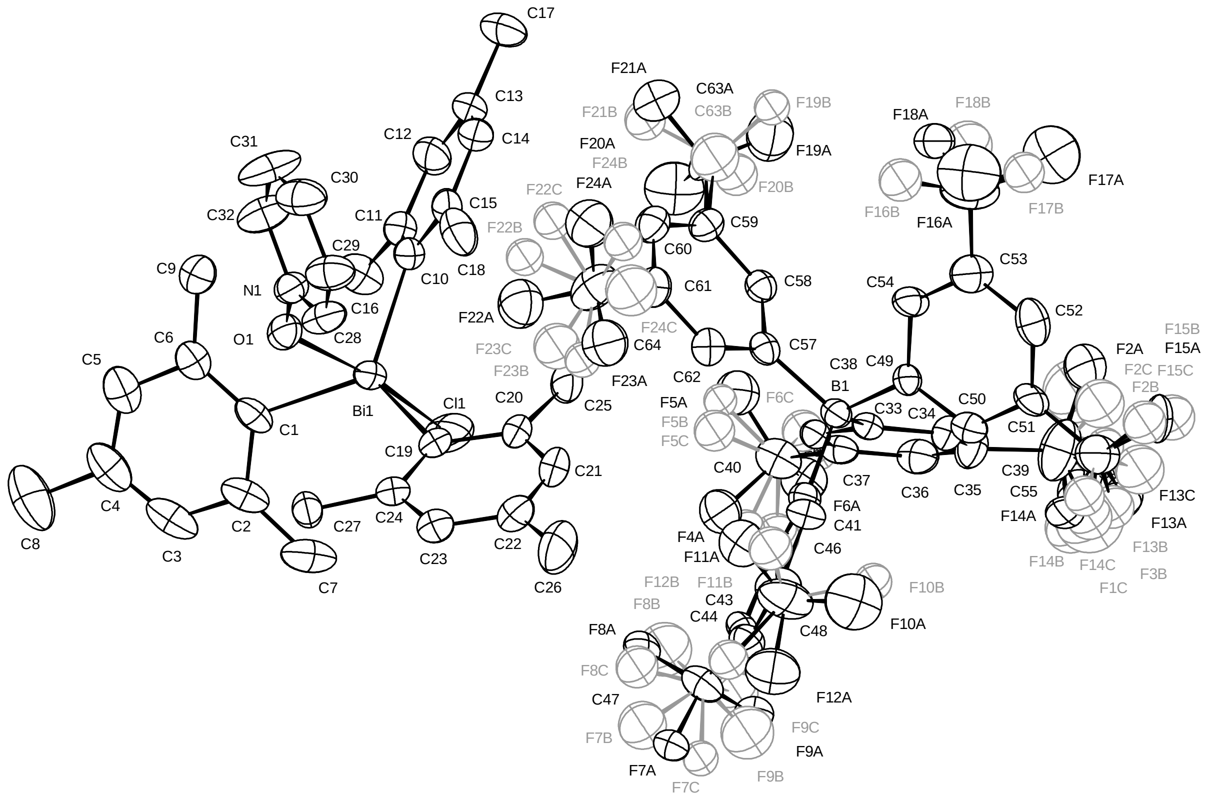

Figure S18: The solid state structure of **13**. H atoms have been removed for clarity and disordered parts shown in grey.

X-ray Crystal Structure Analysis:

$C_{64}H_{50}BBiClF_{24}NO$, M_r = 1560.29 g mol^{-1}, colourless prism, crystal size 0.284 x 0.122 x 0.119 mm^3, triclinic, space group $P1$ [1], a = 10.8125(4) Å, b = 12.0526(4) Å, c = 13.0336(5) Å, α = 91.274(2)°, β = 104.828(2)°, γ = 99.677(2), V = 1614.80(10) Å^3,T = 150(2) K, Z = 1, D_{calc} = 1.604g·cm^3, λ = 0.71073 Å, $\mu(Mo\text{-}K_\alpha)$ = 2.882 mm^{-1}, Gaussian correction ($T_{\min}$ = 0.52512, $T_{\max}$ = 0.83538), Bruker-AXS Kappa Mach3 with APEX-II detector and IµS micro focus Mo-anode X-ray source, 1.620 < θ < 30.032°, 45758 measured reflections, 17115 independent reflections, 15711 reflections with $I > 2\sigma(I)$, R_{int} = 0.0352. The structure was solved by *SHELXT* and refined by full-matrix least-squares (*SHELXL*) against F^2 to R_1 = 0.0371 [$I > 2\sigma(I)$], wR_2 = 0.0806 [all data] with 1007 parameters, 148 restraints and an absolute structure parameter x = 0.003(3).

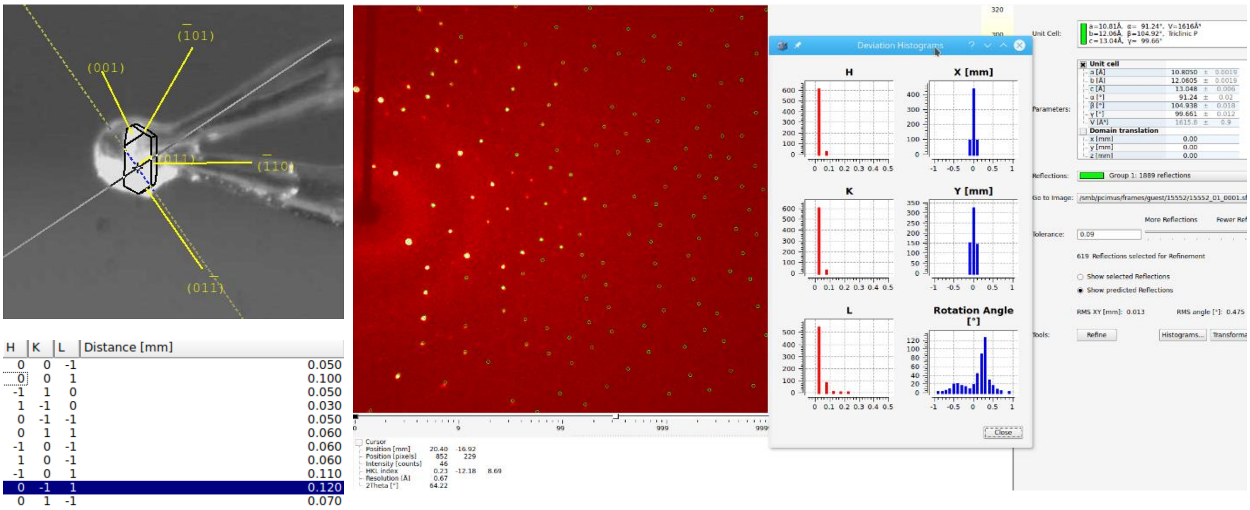

Figure S19: Crystal faces and unit cell determination/refinement of **13**.

INTENSITY STATISTICS FOR DATASET

Resolution	#Data	#Theory	%Complete	Redundancy	Mean I	Mean I/s	Rmerge	Rsigma
Inf - 2.79	155	156	99.4	7.27	181.93	34.81	0.0564	0.0294
2.79 - 1.86	364	366	99.5	7.59	101.75	34.92	0.0495	0.0293
1.86 - 1.48	519	520	99.8	7.73	57.33	33.92	0.0468	0.0301
1.48 - 1.29	536	537	99.8	7.88	39.17	32.63	0.0565	0.0312
1.29 - 1.17	532	532	100.0	7.67	28.12	29.56	0.0660	0.0338
1.17 - 1.09	490	490	100.0	6.96	23.54	26.21	0.0722	0.0382
1.09 - 1.02	592	592	100.0	5.66	19.24	21.68	0.0754	0.0463
1.02 - 0.97	503	503	100.0	5.00	15.37	18.65	0.0819	0.0526
0.97 - 0.93	493	494	99.8	4.36	12.25	15.72	0.0894	0.0632
0.93 - 0.89	624	624	100.0	4.08	9.86	13.54	0.0993	0.0725
0.89 - 0.86	489	489	100.0	3.74	8.12	11.21	0.1150	0.0885
0.86 - 0.84	418	418	100.0	3.69	7.08	9.94	0.1301	0.0986
0.84 - 0.81	642	644	99.7	3.52	6.42	8.84	0.1336	0.1126
0.81 - 0.79	498	498	100.0	3.51	5.27	7.47	0.1571	0.1334
0.79 - 0.77	531	532	99.8	3.34	4.84	6.59	0.1715	0.1516
0.77 - 0.75	621	622	99.8	3.18	4.06	5.39	0.1932	0.1873
0.75 - 0.74	346	347	99.7	3.27	3.44	4.72	0.2190	0.2173
0.74 - 0.72	689	692	99.6	3.07	3.23	4.12	0.2341	0.2470
0.72 - 0.71	387	391	99.0	2.93	2.64	3.36	0.2582	0.3070
0.71 - 0.70	414	418	99.0	2.93	2.57	3.14	0.2802	0.3260
0.70 - 0.69	488	538	90.7	2.46	2.27	2.65	0.2946	0.3931
0.79 - 0.69	3476	3540	98.2	3.02	3.37	4.38	0.2201	0.2364
Inf - 0.69	10331	10403	99.3	4.64	19.28	14.95	0.0650	0.0522

Complete .cif-data of the compound are available under the CCDC number CCDC-**2356070**.

A resolution cut-off (SHEL 999 0.71) was applied to the data set to exclude the poorly determined reflections at high diffraction angles. The terminal –CF_3 groups show strong rotational disorders over two or more positions. They were partially described using the DSR tool plug-in in Olex2.[9,10] For the minor parts of the disorders isotropic displacement parameters were used and atoms were refined with

fixed occupancy values. The ISOR instruction was applied to several F atoms in the main part of the -CF_3 rotational disorders. In addition, the DFIX instruction was used to fix the C…F distances.

Table S6: Crystal data and structure refinement of **13**.

Identification code	15552	
Empirical formula	$C_{64} H_{50} B Bi Cl F_{24} N O$	
Color	colourless	
Formula weight	1560.29 g·mol^{-1}	
Temperature	150(2) K	
Wavelength	0.71073 Å	
Crystal system	Triclinic	
Space group	*P*1, (no. 1)	
Unit cell dimensions	a = 10.8125(4) Å	α= 91.274(2)°.
	b = 12.0526(4) Å	β= 104.828(2)°.
	c = 13.0336(5) Å	γ = 99.677(2)°.
Volume	1614.80(10) Å^3	
Z	1	
Density (calculated)	1.604 Mg·m^{-3}	
Absorption coefficient	2.882 mm^{-1}	
F(000)	770 e	
Crystal size	0.284 x 0.122 x 0.119 mm^3	
θ range for data collection	1.620 to 30.032°.	
Index ranges	-15 ≤ h ≤ 15, -16≤ k ≤ 16, -18 ≤ l ≤ 18	
Reflections collected	45758	
Independent reflections	17115 [R_{int} = 0.0352]	
Reflections with I>2σ(I)	15711	
Completeness to θ = 25.242°	99.9 %	
Absorption correction	Gaussian	
Max. and min. transmission	0.81139 and 0.60986	
Refinement method	Full-matrix least-squares on F^2	
Data / restraints / parameters	17115 / 148 / 1007	
Goodness-of-fit on F^2	0.980	
Final R indices [I>2σ(I)]	R_1 = 0.0371	wR^2 = 0.0777
R indices (all data)	R_1 = 0.0437	wR^2 = 0.0806
Absolute structure parameter	0.003(3)	
Extinction coefficient	n/a	
Largest diff. peak and hole	1.170 and -0.797 e·Å^{-3}	

Table S7: Bond lengths [Å] and angles [°] of **13**.

Bi(1)-Cl(1)	2.507(3)	Bi(1)-O(1)	2.404(6)
Bi(1)-C(1)	2.229(6)	Bi(1)-C(10)	2.226(5)
Bi(1)-C(19)	2.236(6)	O(1)-N(1)	1.334(8)
N(1)-C(28)	1.340(8)	N(1)-C(32)	1.337(9)
C(1)-C(2)	1.400(9)	C(1)-C(6)	1.399(11)
C(2)-C(3)	1.384(11)	C(2)-C(7)	1.497(14)
C(3)-H(3)	0.9500	C(3)-C(4)	1.386(13)
C(4)-C(5)	1.369(12)	C(4)-C(8)	1.512(12)
C(5)-H(5)	0.9500	C(5)-C(6)	1.388(9)
C(6)-C(9)	1.490(11)	C(7)-H(7A)	0.9800
C(7)-H(7B)	0.9800	C(7)-H(7C)	0.9800
C(8)-H(8A)	0.9800	C(8)-H(8B)	0.9800
C(8)-H(8C)	0.9800	C(9)-H(9A)	0.9800
C(9)-H(9B)	0.9800	C(9)-H(9C)	0.9800
C(10)-C(11)	1.396(9)	C(10)-C(15)	1.408(9)
C(11)-C(12)	1.392(9)	C(11)-C(16)	1.504(11)
C(12)-H(12)	0.9500	C(12)-C(13)	1.403(10)
C(13)-C(14)	1.363(11)	C(13)-C(17)	1.512(9)
C(14)-H(14)	0.9500	C(14)-C(15)	1.381(10)
C(15)-C(18)	1.507(11)	C(16)-H(16A)	0.9800
C(16)-H(16B)	0.9800	C(16)-H(16C)	0.9800
C(17)-H(17A)	0.9800	C(17)-H(17B)	0.9800
C(17)-H(17C)	0.9800	C(18)-H(18A)	0.9800
C(18)-H(18B)	0.9800	C(18)-H(18C)	0.9800
C(19)-C(20)	1.404(8)	C(19)-C(24)	1.396(9)
C(20)-C(21)	1.386(10)	C(20)-C(25)	1.506(10)
C(21)-H(21)	0.9500	C(21)-C(22)	1.377(11)
C(22)-C(23)	1.390(9)	C(22)-C(26)	1.501(11)
C(23)-H(23)	0.9500	C(23)-C(24)	1.388(10)
C(24)-C(27)	1.488(9)	C(25)-H(25A)	0.9800
C(25)-H(25B)	0.9800	C(25)-H(25C)	0.9800
C(26)-H(26A)	0.9800	C(26)-H(26B)	0.9800
C(26)-H(26C)	0.9800	C(27)-H(27A)	0.9800
C(27)-H(27B)	0.9800	C(27)-H(27C)	0.9800
C(28)-H(28)	0.9500	C(28)-C(29)	1.367(10)
C(29)-H(29)	0.9500	C(29)-C(30)	1.376(11)

C(30)-H(30)	0.9500	C(30)-C(31)	1.342(13)
C(31)-H(31)	0.9500	C(31)-C(32)	1.386(13)
C(32)-H(32)	0.9500	C(33)-C(34)	1.398(7)
C(33)-C(38)	1.396(7)	C(33)-B(1)	1.636(8)
C(34)-H(34)	0.9500	C(34)-C(35)	1.384(10)
C(35)-C(36)	1.379(10)	C(35)-C(39)	1.526(12)
C(36)-H(36)	0.9500	C(36)-C(37)	1.375(9)
C(37)-C(38)	1.384(8)	C(37)-C(40)	1.490(9)
C(38)-H(38)	0.9500	C(39)-F(1A)	1.277(16)
C(39)-F(2A)	1.491(19)	C(39)-F(3A)	1.355(18)
C(39)-F(1B)	1.35(2)	C(39)-F(2B)	1.10(2)
C(39)-F(3B)	1.55(4)	C(39)-F(1C)	1.48(3)
C(39)-F(2C)	1.41(4)	C(39)-F(3C)	1.11(2)
C(40)-F(4A)	1.374(12)	C(40)-F(5A)	1.324(17)
C(40)-F(6A)	1.288(12)	C(40)-F(4B)	1.313(18)
C(40)-F(5B)	1.40(2)	C(40)-F(6B)	1.42(2)
C(40)-F(4C)	1.36(3)	C(40)-F(5C)	1.20(2)
C(40)-F(6C)	1.39(2)	C(41)-C(42)	1.402(7)
C(41)-C(46)	1.386(8)	C(41)-B(1)	1.650(7)
C(42)-H(42)	0.9500	C(42)-C(43)	1.405(8)
C(43)-C(44)	1.385(9)	C(43)-C(47)	1.484(8)
C(44)-H(44)	0.9500	C(44)-C(45)	1.318(12)
C(45)-C(46)	1.411(10)	C(45)-C(48)	1.501(10)
C(46)-H(46)	0.9500	C(47)-F(7A)	1.346(10)
C(47)-F(8A)	1.361(13)	C(47)-F(9A)	1.356(13)
C(47)-F(7B)	1.348(19)	C(47)-F(8B)	1.36(3)
C(47)-F(9B)	1.28(3)	C(47)-F(7C)	1.411(17)
C(47)-F(8C)	1.19(2)	C(47)-F(9C)	1.31(2)
C(48)-F(10A)	1.238(13)	C(48)-F(11A)	1.377(13)
C(48)-F(12A)	1.309(10)	C(48)-F(10B)	1.574(17)
C(48)-F(11B)	1.22(2)	C(48)-F(12B)	1.459(15)
C(49)-C(50)	1.397(10)	C(49)-C(54)	1.400(9)
C(49)-B(1)	1.635(7)	C(50)-H(50)	0.9500
C(50)-C(51)	1.402(10)	C(51)-C(52)	1.384(14)
C(51)-C(55)	1.497(10)	C(52)-H(52)	0.9500
C(52)-C(53)	1.375(13)	C(53)-C(54)	1.397(8)
C(53)-C(56)	1.472(11)	C(54)-H(54)	0.9500
C(55)-F(13A)	1.407(19)	C(55)-F(14A)	1.468(17)

C(55)-F(15A)	1.336(11)	C(55)-F(13B)	1.21(2)
C(55)-F(14B)	1.21(3)	C(55)-F(15B)	1.42(3)
C(55)-F(13C)	1.33(3)	C(55)-F(14C)	1.16(3)
C(55)-F(15C)	1.40(2)	C(56)-F(16A)	1.138(13)
C(56)-F(17A)	1.556(16)	C(56)-F(18A)	1.324(10)
C(56)-F(16B)	1.524(16)	C(56)-F(17B)	1.314(12)
C(56)-F(18B)	1.222(17)	C(57)-C(58)	1.392(7)
C(57)-C(62)	1.401(7)	C(57)-B(1)	1.639(8)
C(58)-H(58)	0.9500	C(58)-C(59)	1.401(9)
C(59)-C(60)	1.358(10)	C(59)-C(63A)	1.501(13)
C(59)-C(63B)	1.46(4)	C(60)-H(60)	0.9500
C(60)-C(61)	1.399(9)	C(61)-C(62)	1.379(9)
C(61)-C(64)	1.496(9)	C(62)-H(62)	0.9500
C(64)-F(22A)	1.381(18)	C(64)-F(23A)	1.300(13)
C(64)-F(24A)	1.287(17)	C(64)-F(22B)	1.336(17)
C(64)-F(23B)	1.355(17)	C(64)-F(24B)	1.406(18)
C(64)-F(22C)	1.34(2)	C(64)-F(23C)	1.19(2)
C(64)-F(24C)	1.31(2)	F(19A)-C(63A)	1.240(14)
F(20A)-C(63A)	1.38(2)	F(21A)-C(63A)	1.363(12)
F(19B)-C(63B)	1.32(2)	F(20B)-C(63B)	1.32(2)
F(21B)-C(63B)	1.35(2)		
O(1)-Bi(1)-Cl(1)	173.5(2)	C(1)-Bi(1)-Cl(1)	95.0(2)
C(1)-Bi(1)-O(1)	79.7(2)	C(1)-Bi(1)-C(19)	121.7(2)
C(10)-Bi(1)-Cl(1)	92.04(17)	C(10)-Bi(1)-O(1)	93.7(2)
C(10)-Bi(1)-C(1)	117.9(2)	C(10)-Bi(1)-C(19)	119.3(2)
C(19)-Bi(1)-Cl(1)	93.3(2)	C(19)-Bi(1)-O(1)	86.5(2)
N(1)-O(1)-Bi(1)	136.7(4)	O(1)-N(1)-C(28)	120.5(5)
O(1)-N(1)-C(32)	119.2(6)	C(32)-N(1)-C(28)	120.3(6)
C(2)-C(1)-Bi(1)	117.7(5)	C(6)-C(1)-Bi(1)	119.1(5)
C(6)-C(1)-C(2)	123.2(6)	C(1)-C(2)-C(7)	125.1(7)
C(3)-C(2)-C(1)	116.5(8)	C(3)-C(2)-C(7)	118.4(7)
C(2)-C(3)-H(3)	118.4	C(2)-C(3)-C(4)	123.2(7)
C(4)-C(3)-H(3)	118.4	C(3)-C(4)-C(8)	119.7(9)
C(5)-C(4)-C(3)	117.2(6)	C(5)-C(4)-C(8)	123.1(10)
C(4)-C(5)-H(5)	117.9	C(4)-C(5)-C(6)	124.2(8)
C(6)-C(5)-H(5)	117.9	C(1)-C(6)-C(9)	126.2(6)
C(5)-C(6)-C(1)	115.8(7)	C(5)-C(6)-C(9)	118.0(7)

C(2)-C(7)-H(7A)	109.5	C(2)-C(7)-H(7B)	109.5
C(2)-C(7)-H(7C)	109.5	H(7A)-C(7)-H(7B)	109.5
H(7A)-C(7)-H(7C)	109.5	H(7B)-C(7)-H(7C)	109.5
C(4)-C(8)-H(8A)	109.5	C(4)-C(8)-H(8B)	109.5
C(4)-C(8)-H(8C)	109.5	H(8A)-C(8)-H(8B)	109.5
H(8A)-C(8)-H(8C)	109.5	H(8B)-C(8)-H(8C)	109.5
C(6)-C(9)-H(9A)	109.5	C(6)-C(9)-H(9B)	109.5
C(6)-C(9)-H(9C)	109.5	H(9A)-C(9)-H(9B)	109.5
H(9A)-C(9)-H(9C)	109.5	H(9B)-C(9)-H(9C)	109.5
C(11)-C(10)-Bi(1)	117.7(4)	C(11)-C(10)-C(15)	122.0(6)
C(15)-C(10)-Bi(1)	120.1(5)	C(10)-C(11)-C(16)	125.7(6)
C(12)-C(11)-C(10)	117.4(6)	C(12)-C(11)-C(16)	116.9(6)
C(11)-C(12)-H(12)	119.1	C(11)-C(12)-C(13)	121.8(6)
C(13)-C(12)-H(12)	119.1	C(12)-C(13)-C(17)	118.9(7)
C(14)-C(13)-C(12)	118.4(5)	C(14)-C(13)-C(17)	122.7(7)
C(13)-C(14)-H(14)	118.5	C(13)-C(14)-C(15)	122.9(6)
C(15)-C(14)-H(14)	118.5	C(10)-C(15)-C(18)	123.8(6)
C(14)-C(15)-C(10)	117.5(6)	C(14)-C(15)-C(18)	118.7(6)
C(11)-C(16)-H(16A)	109.5	C(11)-C(16)-H(16B)	109.5
C(11)-C(16)-H(16C)	109.5	H(16A)-C(16)-H(16B)	109.5
H(16A)-C(16)-H(16C)	109.5	H(16B)-C(16)-H(16C)	109.5
C(13)-C(17)-H(17A)	109.5	C(13)-C(17)-H(17B)	109.5
C(13)-C(17)-H(17C)	109.5	H(17A)-C(17)-H(17B)	109.5
H(17A)-C(17)-H(17C)	109.5	H(17B)-C(17)-H(17C)	109.5
C(15)-C(18)-H(18A)	109.5	C(15)-C(18)-H(18B)	109.5
C(15)-C(18)-H(18C)	109.5	H(18A)-C(18)-H(18B)	109.5
H(18A)-C(18)-H(18C)	109.5	H(18B)-C(18)-H(18C)	109.5
C(20)-C(19)-Bi(1)	117.8(4)	C(24)-C(19)-Bi(1)	119.1(4)
C(24)-C(19)-C(20)	123.0(6)	C(19)-C(20)-C(25)	126.0(7)
C(21)-C(20)-C(19)	116.5(6)	C(21)-C(20)-C(25)	117.6(6)
C(20)-C(21)-H(21)	118.4	C(22)-C(21)-C(20)	123.2(6)
C(22)-C(21)-H(21)	118.4	C(21)-C(22)-C(23)	117.6(6)
C(21)-C(22)-C(26)	121.3(6)	C(23)-C(22)-C(26)	121.1(7)
C(22)-C(23)-H(23)	118.5	C(24)-C(23)-C(22)	122.9(6)
C(24)-C(23)-H(23)	118.5	C(19)-C(24)-C(27)	126.0(6)
C(23)-C(24)-C(19)	116.6(6)	C(23)-C(24)-C(27)	117.4(6)
C(20)-C(25)-H(25A)	109.5	C(20)-C(25)-H(25B)	109.5
C(20)-C(25)-H(25C)	109.5	H(25A)-C(25)-H(25B)	109.5

H(25A)-C(25)-H(25C)	109.5	H(25B)-C(25)-H(25C)	109.5
C(22)-C(26)-H(26A)	109.5	C(22)-C(26)-H(26B)	109.5
C(22)-C(26)-H(26C)	109.5	H(26A)-C(26)-H(26B)	109.5
H(26A)-C(26)-H(26C)	109.5	H(26B)-C(26)-H(26C)	109.5
C(24)-C(27)-H(27A)	109.5	C(24)-C(27)-H(27B)	109.5
C(24)-C(27)-H(27C)	109.5	H(27A)-C(27)-H(27B)	109.5
H(27A)-C(27)-H(27C)	109.5	H(27B)-C(27)-H(27C)	109.5
N(1)-C(28)-H(28)	119.7	N(1)-C(28)-C(29)	120.6(6)
C(29)-C(28)-H(28)	119.7	C(28)-C(29)-H(29)	120.1
C(28)-C(29)-C(30)	119.9(7)	C(30)-C(29)-H(29)	120.1
C(29)-C(30)-H(30)	120.6	C(31)-C(30)-C(29)	118.7(7)
C(31)-C(30)-H(30)	120.6	C(30)-C(31)-H(31)	119.7
C(30)-C(31)-C(32)	120.7(7)	C(32)-C(31)-H(31)	119.7
N(1)-C(32)-C(31)	119.8(7)	N(1)-C(32)-H(32)	120.1
C(31)-C(32)-H(32)	120.1	C(34)-C(33)-B(1)	123.9(5)
C(38)-C(33)-C(34)	114.8(5)	C(38)-C(33)-B(1)	121.3(4)
C(33)-C(34)-H(34)	118.9	C(35)-C(34)-C(33)	122.1(6)
C(35)-C(34)-H(34)	118.9	C(34)-C(35)-C(39)	118.7(7)
C(36)-C(35)-C(34)	121.5(6)	C(36)-C(35)-C(39)	119.7(7)
C(35)-C(36)-H(36)	121.1	C(37)-C(36)-C(35)	117.7(6)
C(37)-C(36)-H(36)	121.1	C(36)-C(37)-C(38)	120.6(5)
C(36)-C(37)-C(40)	120.2(5)	C(38)-C(37)-C(40)	119.2(5)
C(33)-C(38)-H(38)	118.4	C(37)-C(38)-C(33)	123.2(5)
C(37)-C(38)-H(38)	118.4	C(35)-C(39)-F(3B)	102.8(15)
F(1A)-C(39)-C(35)	112.9(12)	F(1A)-C(39)-F(2A)	110.8(12)
F(1A)-C(39)-F(3A)	112.2(13)	F(2A)-C(39)-C(35)	106.4(9)
F(3A)-C(39)-C(35)	112.8(9)	F(3A)-C(39)-F(2A)	100.9(14)
F(1B)-C(39)-C(35)	112.4(12)	F(1B)-C(39)-F(3B)	115(2)
F(2B)-C(39)-C(35)	121.8(16)	F(2B)-C(39)-F(1B)	105.9(17)
F(2B)-C(39)-F(3B)	99(2)	F(1C)-C(39)-C(35)	103.4(14)
F(2C)-C(39)-C(35)	107.2(16)	F(2C)-C(39)-F(1C)	108(2)
F(3C)-C(39)-C(35)	123.0(14)	F(3C)-C(39)-F(1C)	106.3(18)
F(3C)-C(39)-F(2C)	108(2)	F(4A)-C(40)-C(37)	113.0(6)
F(5A)-C(40)-C(37)	110.4(8)	F(5A)-C(40)-F(4A)	101.4(10)
F(6A)-C(40)-C(37)	116.2(8)	F(6A)-C(40)-F(4A)	106.0(9)
F(6A)-C(40)-F(5A)	108.8(9)	F(4B)-C(40)-C(37)	112.5(9)
F(4B)-C(40)-F(5B)	109.6(14)	F(4B)-C(40)-F(6B)	106.9(14)
F(5B)-C(40)-C(37)	113.2(9)	F(5B)-C(40)-F(6B)	106.8(13)

F(6B)-C(40)-C(37)	107.3(9)	F(4C)-C(40)-C(37)	107.6(12)
F(4C)-C(40)-F(6C)	96.7(16)	F(5C)-C(40)-C(37)	119.7(10)
F(5C)-C(40)-F(4C)	112.5(17)	F(5C)-C(40)-F(6C)	104.4(14)
F(6C)-C(40)-C(37)	113.5(10)	C(42)-C(41)-B(1)	123.1(4)
C(46)-C(41)-C(42)	115.2(5)	C(46)-C(41)-B(1)	121.6(5)
C(41)-C(42)-H(42)	118.7	C(41)-C(42)-C(43)	122.7(5)
C(43)-C(42)-H(42)	118.7	C(42)-C(43)-C(47)	118.5(5)
C(44)-C(43)-C(42)	119.1(6)	C(44)-C(43)-C(47)	122.3(6)
C(43)-C(44)-H(44)	120.4	C(45)-C(44)-C(43)	119.3(6)
C(45)-C(44)-H(44)	120.4	C(44)-C(45)-C(46)	122.5(6)
C(44)-C(45)-C(48)	121.0(7)	C(46)-C(45)-C(48)	116.5(7)
C(41)-C(46)-C(45)	121.1(5)	C(41)-C(46)-H(46)	119.4
C(45)-C(46)-H(46)	119.4	F(7A)-C(47)-C(43)	111.5(6)
F(7A)-C(47)-F(8A)	106.3(9)	F(7A)-C(47)-F(9A)	107.4(9)
F(8A)-C(47)-C(43)	110.7(7)	F(9A)-C(47)-C(43)	111.3(6)
F(9A)-C(47)-F(8A)	109.5(9)	F(7B)-C(47)-C(43)	115.5(9)
F(7B)-C(47)-F(8B)	99.9(14)	F(8B)-C(47)-C(43)	114.2(12)
F(9B)-C(47)-C(43)	116.6(12)	F(9B)-C(47)-F(7B)	107.8(15)
F(9B)-C(47)-F(8B)	100.7(16)	F(7C)-C(47)-C(43)	107.9(8)
F(8C)-C(47)-C(43)	115.0(12)	F(8C)-C(47)-F(7C)	108.2(13)
F(8C)-C(47)-F(9C)	102.4(15)	F(9C)-C(47)-C(43)	114.7(10)
F(9C)-C(47)-F(7C)	108.2(13)	C(45)-C(48)-F(10B)	107.4(8)
F(10A)-C(48)-C(45)	115.3(9)	F(10A)-C(48)-F(11A)	110.4(9)
F(10A)-C(48)-F(12A)	90.4(8)	F(11A)-C(48)-C(45)	110.7(7)
F(12A)-C(48)-C(45)	114.6(8)	F(12A)-C(48)-F(11A)	114.1(8)
F(11B)-C(48)-C(45)	124.2(11)	F(11B)-C(48)-F(10B)	83.6(11)
F(11B)-C(48)-F(12B)	100.5(12)	F(12B)-C(48)-C(45)	107.6(8)
F(12B)-C(48)-F(10B)	133.7(9)	C(50)-C(49)-C(54)	115.0(5)
C(50)-C(49)-B(1)	121.7(6)	C(54)-C(49)-B(1)	123.2(5)
C(49)-C(50)-H(50)	118.6	C(49)-C(50)-C(51)	122.7(7)
C(51)-C(50)-H(50)	118.6	C(50)-C(51)-C(55)	118.8(7)
C(52)-C(51)-C(50)	120.3(7)	C(52)-C(51)-C(55)	120.9(7)
C(51)-C(52)-H(52)	120.7	C(53)-C(52)-C(51)	118.6(7)
C(53)-C(52)-H(52)	120.7	C(52)-C(53)-C(54)	120.6(7)
C(52)-C(53)-C(56)	120.9(7)	C(54)-C(53)-C(56)	118.5(7)
C(49)-C(54)-H(54)	118.6	C(53)-C(54)-C(49)	122.8(6)
C(53)-C(54)-H(54)	118.6	F(13A)-C(55)-C(51)	111.5(8)
F(13A)-C(55)-F(14A)	106.8(13)	F(14A)-C(55)-C(51)	106.3(8)

F(15A)-C(55)-C(51)	113.2(8)	F(15A)-C(55)-F(13A)	109.9(11)
F(15A)-C(55)-F(14A)	108.9(11)	F(13B)-C(55)-C(51)	118.1(11)
F(13B)-C(55)-F(14B)	97.7(17)	F(13B)-C(55)-F(15B)	97.9(16)
F(14B)-C(55)-C(51)	113.7(16)	F(14B)-C(55)-F(15B)	114.6(15)
F(15B)-C(55)-C(51)	113.1(12)	F(13C)-C(55)-C(51)	109.4(12)
F(13C)-C(55)-F(15C)	111.0(15)	F(14C)-C(55)-C(51)	124.6(14)
F(14C)-C(55)-F(13C)	101.9(17)	F(14C)-C(55)-F(15C)	98.9(16)
F(15C)-C(55)-C(51)	110.3(12)	C(53)-C(56)-F(17A)	107.1(9)
C(53)-C(56)-F(16B)	107.7(8)	F(16A)-C(56)-C(53)	121.9(9)
F(16A)-C(56)-F(17A)	96.9(9)	F(16A)-C(56)-F(18A)	115.4(11)
F(18A)-C(56)-C(53)	116.3(6)	F(18A)-C(56)-F(17A)	90.8(8)
F(17B)-C(56)-C(53)	113.7(8)	F(17B)-C(56)-F(16B)	95.4(8)
F(18B)-C(56)-C(53)	112.6(10)	F(18B)-C(56)-F(16B)	112.8(11)
F(18B)-C(56)-F(17B)	113.4(11)	C(58)-C(57)-C(62)	115.2(5)
C(58)-C(57)-B(1)	122.5(4)	C(62)-C(57)-B(1)	122.0(5)
C(57)-C(58)-H(58)	118.8	C(57)-C(58)-C(59)	122.3(5)
C(59)-C(58)-H(58)	118.8	C(58)-C(59)-C(63A)	118.6(6)
C(58)-C(59)-C(63B)	117.6(9)	C(60)-C(59)-C(58)	121.3(6)
C(60)-C(59)-C(63A)	120.1(6)	C(60)-C(59)-C(63B)	120.8(9)
C(59)-C(60)-H(60)	121.1	C(59)-C(60)-C(61)	117.7(6)
C(61)-C(60)-H(60)	121.1	C(60)-C(61)-C(64)	118.1(6)
C(62)-C(61)-C(60)	120.9(5)	C(62)-C(61)-C(64)	121.0(6)
C(57)-C(62)-H(62)	118.7	C(61)-C(62)-C(57)	122.6(5)
C(61)-C(62)-H(62)	118.7	F(22A)-C(64)-C(61)	110.1(9)
F(23A)-C(64)-C(61)	114.1(8)	F(23A)-C(64)-F(22A)	103.1(12)
F(24A)-C(64)-C(61)	116.0(9)	F(24A)-C(64)-F(22A)	107.6(13)
F(24A)-C(64)-F(23A)	105.0(11)	F(22B)-C(64)-C(61)	113.9(8)
F(22B)-C(64)-F(23B)	108.6(12)	F(22B)-C(64)-F(24B)	106.5(13)
F(23B)-C(64)-C(61)	113.5(9)	F(23B)-C(64)-F(24B)	104.8(12)
F(24B)-C(64)-C(61)	109.0(9)	F(22C)-C(64)-C(61)	113.9(9)
F(23C)-C(64)-C(61)	114.6(13)	F(23C)-C(64)-F(22C)	114.9(17)
F(23C)-C(64)-F(24C)	99.6(19)	F(24C)-C(64)-C(61)	108.9(13)
F(24C)-C(64)-F(22C)	103.0(17)	C(33)-B(1)-C(41)	109.1(4)
C(33)-B(1)-C(57)	110.3(4)	C(49)-B(1)-C(33)	110.4(4)
C(49)-B(1)-C(41)	109.5(4)	C(49)-B(1)-C(57)	107.5(4)
C(57)-B(1)-C(41)	110.1(4)	F(19A)-C(63A)-C(59)	117.2(11)
F(19A)-C(63A)-F(20A)	101.4(10)	F(19A)-C(63A)-F(21A)	110.4(12)
F(20A)-C(63A)-C(59)	110.7(11)	F(21A)-C(63A)-C(59)	112.2(8)

F(21A)-C(63A)-F(20A)	103.5(11)	F(19B)-C(63B)-C(59)	115(2)
F(19B)-C(63B)-F(21B)	112(2)	F(20B)-C(63B)-C(59)	115(2)
F(20B)-C(63B)-F(19B)	99(2)	F(20B)-C(63B)-F(21B)	102(2)
F(21B)-C(63B)-C(59)	111.7(19)		

NOTE: The following X-ray data of structure **7, 8, 9a** and **11** presented here has been previously communicated in the thesis[4]. These structures were already uploaded at the CCDC prior to this communication.

9.4 Single crystal structure analysis of tris(3,5-di-t-butylphenyl)bismuth dichloride (7)

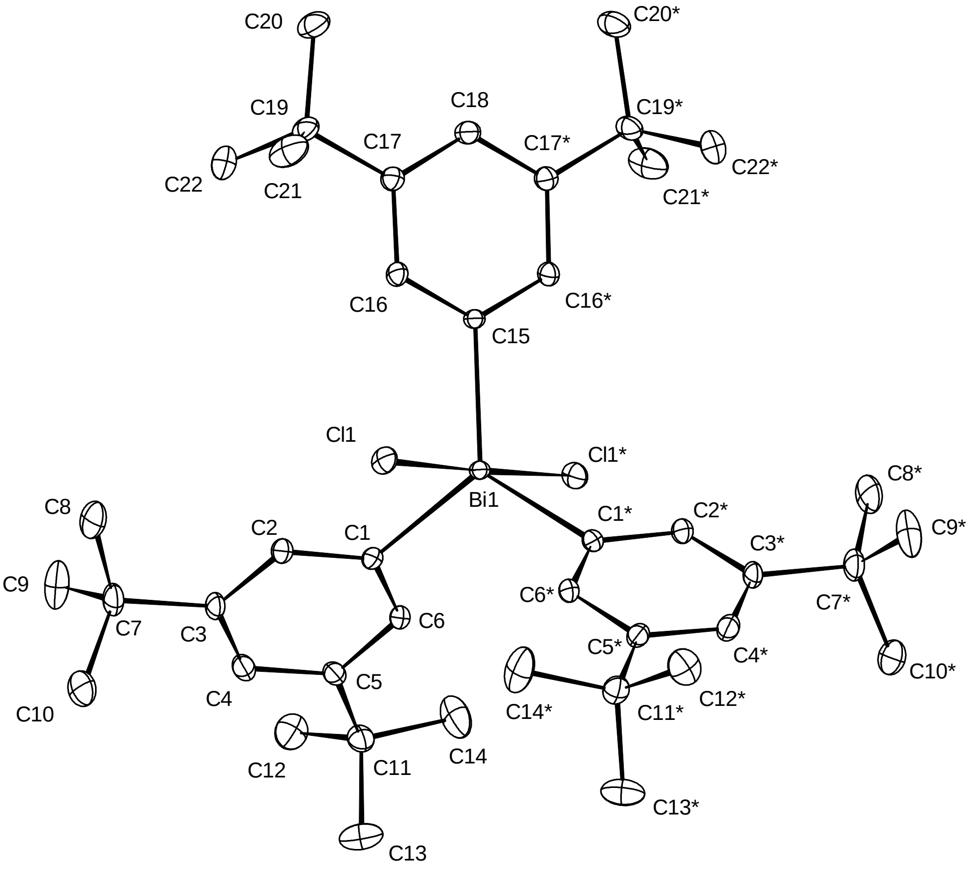

Figure S20: The solid state structure of **7**. H atoms have been removed for clarity.

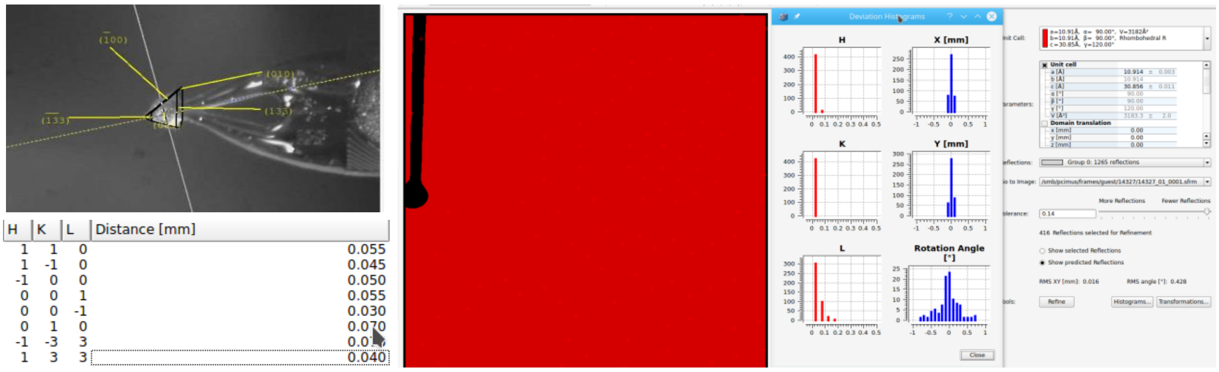

Figure S21: Crystal faces and unit cell determination/refinement of **7**.

INTENSITY STATISTICS FOR DATASET # 1 14327sadabs.raw

Resolution	#Data	#Theory	%Complete	Redundancy	Mean I	Mean I/s	Rmerge	Rsigma
Inf - 2.41	120	121	99.2	49.07	120.43	159.17	0.0484	0.0063
2.41 - 1.53	285	286	99.7	67.24	81.79	172.09	0.0422	0.0047
1.53 - 1.19	399	399	100.0	72.58	50.87	151.72	0.0402	0.0046
1.19 - 1.03	413	414	99.8	63.39	40.33	130.54	0.0455	0.0052
1.03 - 0.93	398	398	100.0	50.34	30.34	102.80	0.0468	0.0063
0.93 - 0.86	408	408	100.0	44.07	25.27	88.11	0.0470	0.0072
0.86 - 0.81	370	370	100.0	41.02	22.12	81.30	0.0523	0.0081
0.81 - 0.76	479	479	100.0	38.92	19.81	74.25	0.0549	0.0089
0.76 - 0.73	343	343	100.0	37.41	17.22	67.69	0.0665	0.0099
0.73 - 0.70	425	425	100.0	34.99	14.83	61.94	0.0663	0.0114
0.70 - 0.67	463	463	100.0	34.04	12.80	54.71	0.0773	0.0129
0.67 - 0.65	392	392	100.0	31.80	12.08	50.82	0.0761	0.0143
0.65 - 0.63	415	415	100.0	31.34	10.62	45.97	0.0836	0.0160
0.63 - 0.61	486	486	100.0	29.04	10.47	43.93	0.0883	0.0173
0.61 - 0.60	249	249	100.0	28.00	9.09	37.85	0.0988	0.0203
0.60 - 0.58	586	586	100.0	17.27	8.57	27.78	0.1030	0.0289
0.58 - 0.57	314	314	100.0	13.72	7.10	20.65	0.1143	0.0387
0.57 - 0.56	349	349	100.0	13.26	7.00	20.40	0.1188	0.0401
0.56 - 0.55	360	360	100.0	13.02	6.52	18.96	0.1288	0.0439
0.55 - 0.54	388	388	100.0	12.27	5.93	16.68	0.1363	0.0497
0.54 - 0.53	313	397	78.8	4.38	5.35	8.57	0.1592	0.1193
0.63 - 0.53	3045	3129	97.3	16.40	7.67	25.17	0.1044	0.0377
Inf - 0.53	7955	8042	98.9	33.88	20.80	64.82	0.0515	0.0115

Complete .cif-data of the compound are available under the CCDC number CCDC-**2289340**.

Table S8. Crystal data and structure refinement.

Identification code	14327sadabs	
Empirical formula	C42 H63 Bi Cl2	
Color	colourless	
Formula weight	847.80 g·mol^{-1}	
Temperature	100(2) K	
Wavelength	0.71073 Å	
Crystal system	Trigonal	
Space group	P 3_1 2 1, (no. 152)	
Unit cell dimensions	a = 10.7948(4) Å	α= 90°.
	b = 10.7948(4) Å	β= 90°.
	c = 30.6951(19) Å	γ = 120°.
Volume	3097.6(3) Å^3	
Z	3	
Density (calculated)	1.363 Mg·m^{-3}	
Absorption coefficient	4.424 mm^{-1}	
F(000)	1296 e	
Crystal size	0.172 x 0.118 x 0.10 mm^3	
θ range for data collection	1.990 to 38.567°.	
Index ranges	-18 ≤ h ≤ 18, -18≤ k ≤ 18, -53 ≤ l ≤ 53	
Reflections collected	256310	
Independent reflections	11669 [R_{int} = 0.0338]	
Reflections with I>2σ(I)	11488	
Completeness to θ = 25.242°	100.0 %	
Absorption correction	Gaussian	
Max. and min. transmission	0.73065 and 0.60834	
Refinement method	Full-matrix least-squares on F^2	
Data / restraints / parameters	11669 / 0 / 215	
Goodness-of-fit on F^2	1.006	
Final R indices [I>2σ(I)]	R_1 = 0.0241	wR^2 = 0.0475
R indices (all data)	R_1 = 0.0248	wR^2 = 0.0478
Absolute structure parameter	0.045(5)	
Extinction coefficient	n/a	
Largest diff. peak and hole	1.913 and -1.628 e·Å^{-3}	

Table S9. Bond lengths [Å] and angles [°].

Bi(1)-Cl(1)	2.5954(5)	Bi(1)-Cl(1)#1	2.5953(5)
Bi(1)-C(1)#1	2.221(2)	Bi(1)-C(1)	2.221(2)
Bi(1)-C(15)	2.212(3)	C(1)-C(2)	1.383(3)
C(1)-C(6)	1.384(3)	C(2)-H(2)	0.9500
C(2)-C(3)	1.398(3)	C(3)-C(4)	1.397(4)
C(3)-C(7)	1.530(3)	C(4)-H(4)	0.9500
C(4)-C(5)	1.397(3)	C(5)-C(6)	1.397(3)
C(5)-C(11)	1.528(3)	C(6)-H(6)	0.9500
C(7)-C(8)	1.525(4)	C(7)-C(9)	1.538(4)
C(7)-C(10)	1.531(4)	C(8)-H(8A)	0.9800
C(8)-H(8B)	0.9800	C(8)-H(8C)	0.9800
C(9)-H(9A)	0.9800	C(9)-H(9B)	0.9800
C(9)-H(9C)	0.9800	C(10)-H(10A)	0.9800
C(10)-H(10B)	0.9800	C(10)-H(10C)	0.9800
C(11)-C(12)	1.535(4)	C(11)-C(13)	1.528(4)
C(11)-C(14)	1.529(4)	C(12)-H(12A)	0.9800
C(12)-H(12B)	0.9800	C(12)-H(12C)	0.9800
C(13)-H(13A)	0.9800	C(13)-H(13B)	0.9800
C(13)-H(13C)	0.9800	C(14)-H(14A)	0.9800
C(14)-H(14B)	0.9800	C(14)-H(14C)	0.9800
C(15)-C(16)	1.382(3)	C(15)-C(16)#1	1.382(3)
C(16)-H(16)	0.9500	C(16)-C(17)	1.396(3)
C(17)-C(18)	1.401(3)	C(17)-C(19)	1.531(3)
C(18)-H(18)	0.9500	C(19)-C(20)	1.532(4)
C(19)-C(21)	1.541(4)	C(19)-C(22)	1.537(4)
C(20)-H(20A)	0.9800	C(20)-H(20B)	0.9800
C(20)-H(20C)	0.9800	C(21)-H(21A)	0.9800
C(21)-H(21B)	0.9800	C(21)-H(21C)	0.9800
C(22)-H(22A)	0.9800	C(22)-H(22B)	0.9800
C(22)-H(22C)	0.9800		
Cl(1)#1-Bi(1)-Cl(1)	178.59(3)	C(1)-Bi(1)-Cl(1)	89.96(6)
C(1)#1-Bi(1)-Cl(1)#1	89.96(6)	C(1)#1-Bi(1)-Cl(1)	90.78(6)
C(1)-Bi(1)-Cl(1)#1	90.78(6)	C(1)#1-Bi(1)-C(1)	116.83(12)
C(15)-Bi(1)-Cl(1)#1	89.293(15)	C(15)-Bi(1)-Cl(1)	89.293(14)
C(15)-Bi(1)-C(1)	121.59(6)	C(15)-Bi(1)-C(1)#1	121.58(6)

C(2)-C(1)-Bi(1)	119.89(16)	C(2)-C(1)-C(6)	123.1(2)
C(6)-C(1)-Bi(1)	116.94(16)	C(1)-C(2)-H(2)	120.5
C(1)-C(2)-C(3)	119.0(2)	C(3)-C(2)-H(2)	120.5
C(2)-C(3)-C(7)	122.5(2)	C(4)-C(3)-C(2)	117.7(2)
C(4)-C(3)-C(7)	119.8(2)	C(3)-C(4)-H(4)	118.3
C(3)-C(4)-C(5)	123.4(2)	C(5)-C(4)-H(4)	118.3
C(4)-C(5)-C(11)	121.0(2)	C(6)-C(5)-C(4)	117.8(2)
C(6)-C(5)-C(11)	121.1(2)	C(1)-C(6)-C(5)	118.9(2)
C(1)-C(6)-H(6)	120.5	C(5)-C(6)-H(6)	120.5
C(3)-C(7)-C(9)	109.1(2)	C(3)-C(7)-C(10)	110.0(2)
C(8)-C(7)-C(3)	112.4(2)	C(8)-C(7)-C(9)	107.9(3)
C(8)-C(7)-C(10)	108.4(3)	C(10)-C(7)-C(9)	108.9(3)
C(7)-C(8)-H(8A)	109.5	C(7)-C(8)-H(8B)	109.5
C(7)-C(8)-H(8C)	109.5	H(8A)-C(8)-H(8B)	109.5
H(8A)-C(8)-H(8C)	109.5	H(8B)-C(8)-H(8C)	109.5
C(7)-C(9)-H(9A)	109.5	C(7)-C(9)-H(9B)	109.5
C(7)-C(9)-H(9C)	109.5	H(9A)-C(9)-H(9B)	109.5
H(9A)-C(9)-H(9C)	109.5	H(9B)-C(9)-H(9C)	109.5
C(7)-C(10)-H(10A)	109.5	C(7)-C(10)-H(10B)	109.5
C(7)-C(10)-H(10C)	109.5	H(10A)-C(10)-H(10B)	109.5
H(10A)-C(10)-H(10C)	109.5	H(10B)-C(10)-H(10C)	109.5
C(5)-C(11)-C(12)	110.7(2)	C(5)-C(11)-C(14)	112.1(2)
C(13)-C(11)-C(5)	108.8(2)	C(13)-C(11)-C(12)	109.0(2)
C(13)-C(11)-C(14)	108.7(3)	C(14)-C(11)-C(12)	107.5(2)
C(11)-C(12)-H(12A)	109.5	C(11)-C(12)-H(12B)	109.5
C(11)-C(12)-H(12C)	109.5	H(12A)-C(12)-H(12B)	109.5
H(12A)-C(12)-H(12C)	109.5	H(12B)-C(12)-H(12C)	109.5
C(11)-C(13)-H(13A)	109.5	C(11)-C(13)-H(13B)	109.5
C(11)-C(13)-H(13C)	109.5	H(13A)-C(13)-H(13B)	109.5
H(13A)-C(13)-H(13C)	109.5	H(13B)-C(13)-H(13C)	109.5
C(11)-C(14)-H(14A)	109.5	C(11)-C(14)-H(14B)	109.5
C(11)-C(14)-H(14C)	109.5	H(14A)-C(14)-H(14B)	109.5
H(14A)-C(14)-H(14C)	109.5	H(14B)-C(14)-H(14C)	109.5
C(16)#1-C(15)-Bi(1)	118.38(14)	C(16)-C(15)-Bi(1)	118.38(14)
C(16)#1-C(15)-C(16)	123.2(3)	C(15)-C(16)-H(16)	120.5
C(15)-C(16)-C(17)	118.9(2)	C(17)-C(16)-H(16)	120.5
C(16)-C(17)-C(18)	118.2(2)	C(16)-C(17)-C(19)	119.1(2)
C(18)-C(17)-C(19)	122.8(2)	C(17)#1-C(18)-C(17)	122.6(3)

C(17)-C(18)-H(18)	118.7	C(17)#1-C(18)-H(18)	118.7
C(17)-C(19)-C(20)	112.1(2)	C(17)-C(19)-C(21)	109.3(2)
C(17)-C(19)-C(22)	108.7(2)	C(20)-C(19)-C(21)	108.2(2)
C(20)-C(19)-C(22)	108.8(2)	C(22)-C(19)-C(21)	109.8(2)
C(19)-C(20)-H(20A)	109.5	C(19)-C(20)-H(20B)	109.5
C(19)-C(20)-H(20C)	109.5	H(20A)-C(20)-H(20B)	109.5
H(20A)-C(20)-H(20C)	109.5	H(20B)-C(20)-H(20C)	109.5
C(19)-C(21)-H(21A)	109.5	C(19)-C(21)-H(21B)	109.5
C(19)-C(21)-H(21C)	109.5	H(21A)-C(21)-H(21B)	109.5
H(21A)-C(21)-H(21C)	109.5	H(21B)-C(21)-H(21C)	109.5
C(19)-C(22)-H(22A)	109.5	C(19)-C(22)-H(22B)	109.5
C(19)-C(22)-H(22C)	109.5	H(22A)-C(22)-H(22B)	109.5
H(22A)-C(22)-H(22C)	109.5	H(22B)-C(22)-H(22C)	109.5

Symmetry transformations used to generate equivalent atoms:

#1 x-y+1,-y+2,-z+2/3

9.5 Single crystal structure analysis of tris(2,4,6-trimethylphenyl)-λ^5-bismuth dichloride (8)

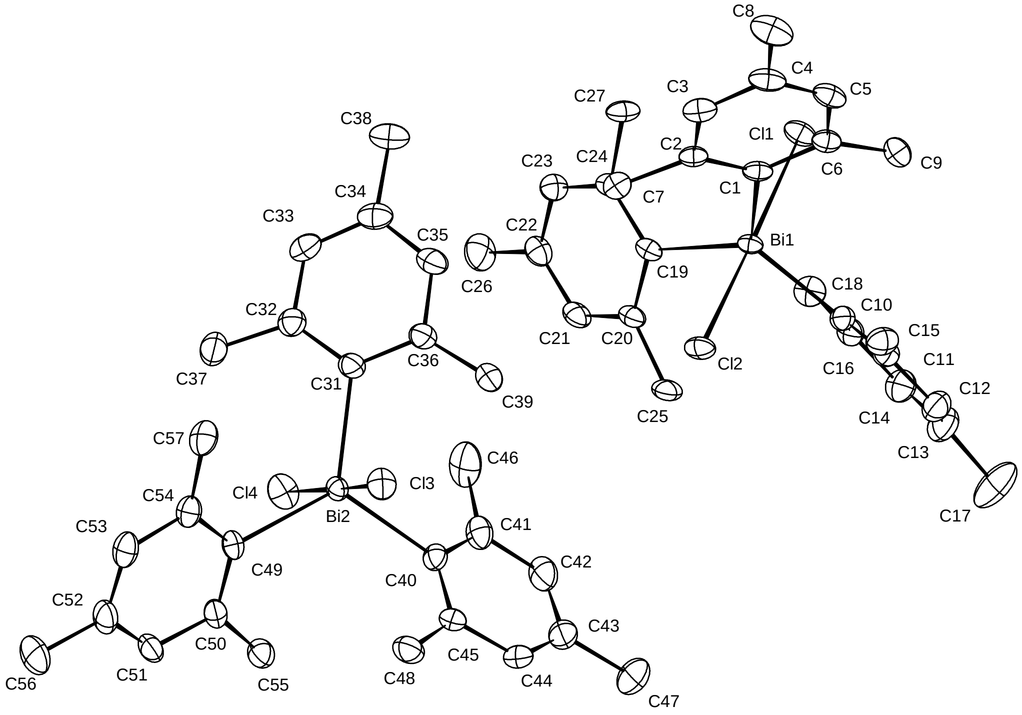

Figure S22: The solid state structure of **8**. H atoms have been removed for clarity.

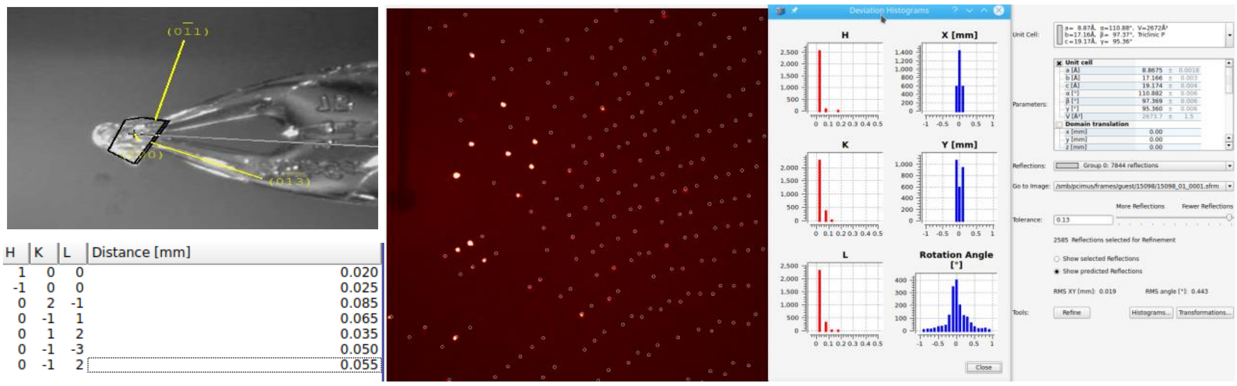

Figure S23: Crystal faces and unit cell determination/refinement of **8**.

INTENSITY STATISTICS FOR DATASET # 1 15098sadabs.raw

Resolution	#Data	#Theory	%Complete	Redundancy	Mean I	Mean I/s	Rmerge	Rsigma
Inf - 2.47	356	357	99.7	8.68	130.57	44.48	0.0363	0.0208
2.47 - 1.65	848	848	100.0	8.94	91.06	41.96	0.0342	0.0209
1.65 - 1.31	1184	1184	100.0	8.92	60.26	38.84	0.0357	0.0214
1.31 - 1.15	1146	1146	100.0	8.64	44.02	34.93	0.0396	0.0226
1.15 - 1.04	1247	1247	100.0	7.08	37.16	30.13	0.0466	0.0269
1.04 - 0.96	1288	1288	100.0	5.16	28.03	22.16	0.0537	0.0347
0.96 - 0.91	1072	1072	100.0	4.35	23.18	18.74	0.0617	0.0406
0.91 - 0.86	1297	1297	100.0	3.89	20.33	16.39	0.0679	0.0456
0.86 - 0.82	1316	1316	100.0	3.69	17.83	14.49	0.0744	0.0525
0.82 - 0.79	1118	1118	100.0	3.53	16.02	13.36	0.0751	0.0579
0.79 - 0.77	909	909	100.0	3.39	14.32	12.40	0.0866	0.0649
0.77 - 0.74	1473	1475	99.9	3.35	12.38	11.37	0.0908	0.0731
0.74 - 0.72	1142	1143	99.9	3.17	10.95	10.22	0.1007	0.0863
0.72 - 0.70	1266	1266	100.0	3.04	9.90	9.29	0.1070	0.0967
0.70 - 0.68	1388	1401	99.1	2.97	8.73	8.59	0.1142	0.1071
0.68 - 0.67	783	791	99.0	2.91	8.65	8.34	0.1155	0.1164
0.67 - 0.65	1689	1712	98.7	2.76	7.05	6.87	0.1392	0.1449
0.65 - 0.64	918	932	98.5	2.73	6.98	6.86	0.1470	0.1384
0.64 - 0.63	973	988	98.5	2.63	6.39	6.20	0.1583	0.1668
0.63 - 0.62	1040	1066	97.6	2.58	5.77	5.69	0.1723	0.1810
0.62 - 0.61	1073	1374	78.1	1.77	5.28	4.89	0.1872	0.2171
0.71 - 0.61	8510	8910	95.5	2.64	7.22	6.98	0.1360	0.1410
Inf - 0.61	23526	23930	98.3	4.27	22.61	16.32	0.0491	0.0465

Complete .cif-data of the compound are available under the CCDC number CCDC-**2296787.**

Table S10. Crystal data and structure refinement.

Identification code	15098	
Empirical formula	$C_{27} H_{33} Bi Cl_2$	
Color	colourless	
Formula weight	637.41 g·mol^{-1}	
Temperature	100(2) K	
Wavelength	0.71073 Å	
Crystal system	Triclinic	
Space group	*P*-1, (no. 2)	
Unit cell dimensions	a = 8.7522(10) Å	α= 110.973(4)°.
	b = 16.9260(19) Å	β= 97.457(4)°.
	c = 18.920(2) Å	γ = 95.362(4)°.
Volume	2565.2(5) Å^3	
Z	4	
Density (calculated)	1.650 Mg·m^{-3}	
Absorption coefficient	7.093 mm^{-1}	
F(000)	1248 e	
Crystal size	0.197 x 0.11 x 0.052 mm^3	
θ range for data collection	1.303 to 30.505°.	
Index ranges	$-12 \le h \le 12$, $-24 \le k \le 24$, $-27 \le l \le 27$	
Reflections collected	80609	
Independent reflections	15659 [R_{int} = 0.0452]	
Reflections with I>2σ(I)	13657	
Completeness to θ = 25.242°	100.0 %	
Absorption correction	Gaussian	
Max. and min. transmission	0.73960 and 0.37248	
Refinement method	Full-matrix least-squares on F^2	
Data / restraints / parameters	15659 / 0 / 559	
Goodness-of-fit on F^2	1.078	
Final R indices [I>2σ(I)]	R_1 = 0.0269	wR^2 = 0.0664
R indices (all data)	R_1 = 0.0341	wR^2 = 0.0689
Extinction coefficient	n/a	
Largest diff. peak and hole	2.351 and -1.569 e·Å^{-3}	

Table S11. Bond lengths [Å] and angles [°].

Bi(1)-Cl(1)	2.6029(7)	Bi(1)-Cl(2)	2.6037(7)
Bi(1)-C(1)	2.233(3)	Bi(1)-C(10)	2.237(3)
Bi(1)-C(19)	2.233(3)	C(1)-C(2)	1.394(4)
C(1)-C(6)	1.395(4)	C(2)-C(3)	1.388(4)
C(2)-C(7)	1.505(4)	C(3)-H(3)	0.9500
C(3)-C(4)	1.387(5)	C(4)-C(5)	1.377(5)
C(4)-C(8)	1.509(4)	C(5)-H(5)	0.9500
C(5)-C(6)	1.399(4)	C(6)-C(9)	1.503(4)
C(7)-H(7A)	0.9800	C(7)-H(7B)	0.9800
C(7)-H(7C)	0.9800	C(8)-H(8A)	0.9800
C(8)-H(8B)	0.9800	C(8)-H(8C)	0.9800
C(9)-H(9A)	0.9800	C(9)-H(9B)	0.9800
C(9)-H(9C)	0.9800	C(10)-C(11)	1.396(4)
C(10)-C(15)	1.403(4)	C(11)-C(12)	1.395(5)
C(11)-C(16)	1.509(5)	C(12)-H(12)	0.9500
C(12)-C(13)	1.386(5)	C(13)-C(14)	1.380(5)
C(13)-C(17)	1.512(5)	C(14)-H(14)	0.9500
C(14)-C(15)	1.392(4)	C(15)-C(18)	1.514(4)
C(16)-H(16A)	0.9800	C(16)-H(16B)	0.9800
C(16)-H(16C)	0.9800	C(17)-H(17A)	0.9800
C(17)-H(17B)	0.9800	C(17)-H(17C)	0.9800
C(18)-H(18A)	0.9800	C(18)-H(18B)	0.9800
C(18)-H(18C)	0.9800	C(19)-C(20)	1.401(4)
C(19)-C(24)	1.389(4)	C(20)-C(21)	1.393(4)
C(20)-C(25)	1.504(4)	C(21)-H(21)	0.9500
C(21)-C(22)	1.382(4)	C(22)-C(23)	1.383(4)
C(22)-C(26)	1.509(4)	C(23)-H(23)	0.9500
C(23)-C(24)	1.397(4)	C(24)-C(27)	1.501(4)
C(25)-H(25A)	0.9800	C(25)-H(25B)	0.9800
C(25)-H(25C)	0.9800	C(26)-H(26A)	0.9800
C(26)-H(26B)	0.9800	C(26)-H(26C)	0.9800
C(27)-H(27A)	0.9800	C(27)-H(27B)	0.9800
C(27)-H(27C)	0.9800	Bi(2)-Cl(3)	2.5925(8)
Bi(2)-Cl(4)	2.6283(8)	Bi(2)-C(31)	2.245(3)
Bi(2)-C(40)	2.226(3)	Bi(2)-C(49)	2.237(3)
C(31)-C(32)	1.392(4)	C(31)-C(36)	1.403(4)

C(32)-C(33)	1.397(5)	C(32)-C(37)	1.498(5)
C(33)-H(33)	0.9500	C(33)-C(34)	1.383(5)
C(34)-C(35)	1.385(5)	C(34)-C(38)	1.500(5)
C(35)-H(35)	0.9500	C(35)-C(36)	1.390(4)
C(36)-C(39)	1.504(4)	C(37)-H(37A)	0.9800
C(37)-H(37B)	0.9800	C(37)-H(37C)	0.9800
C(38)-H(38A)	0.9800	C(38)-H(38B)	0.9800
C(38)-H(38C)	0.9800	C(39)-H(39A)	0.9800
C(39)-H(39B)	0.9800	C(39)-H(39C)	0.9800
C(40)-C(41)	1.406(5)	C(40)-C(45)	1.398(4)
C(41)-C(42)	1.397(5)	C(41)-C(46)	1.505(5)
C(42)-H(42)	0.9500	C(42)-C(43)	1.372(6)
C(43)-C(44)	1.374(6)	C(43)-C(47)	1.507(5)
C(44)-H(44)	0.9500	C(44)-C(45)	1.400(5)
C(45)-C(48)	1.492(5)	C(46)-H(46A)	0.9800
C(46)-H(46B)	0.9800	C(46)-H(46C)	0.9800
C(47)-H(47A)	0.9800	C(47)-H(47B)	0.9800
C(47)-H(47C)	0.9800	C(48)-H(48A)	0.9800
C(48)-H(48B)	0.9800	C(48)-H(48C)	0.9800
C(49)-C(50)	1.400(4)	C(49)-C(54)	1.396(4)
C(50)-C(51)	1.398(4)	C(50)-C(55)	1.506(4)
C(51)-H(51)	0.9500	C(51)-C(52)	1.380(5)
C(52)-C(53)	1.380(5)	C(52)-C(56)	1.510(5)
C(53)-H(53)	0.9500	C(53)-C(54)	1.393(5)
C(54)-C(57)	1.502(5)	C(55)-H(55A)	0.9800
C(55)-H(55B)	0.9800	C(55)-H(55C)	0.9800
C(56)-H(56A)	0.9800	C(56)-H(56B)	0.9800
C(56)-H(56C)	0.9800	C(57)-H(57A)	0.9800
C(57)-H(57B)	0.9800	C(57)-H(57C)	0.9800
Cl(1)-Bi(1)-Cl(2)	175.65(2)	C(1)-Bi(1)-Cl(1)	87.72(7)
C(1)-Bi(1)-Cl(2)	87.95(7)	C(1)-Bi(1)-C(10)	123.31(11)
C(10)-Bi(1)-Cl(1)	92.46(8)	C(10)-Bi(1)-Cl(2)	90.26(8)
C(19)-Bi(1)-Cl(1)	90.54(7)	C(19)-Bi(1)-Cl(2)	91.43(7)
C(19)-Bi(1)-C(1)	122.32(10)	C(19)-Bi(1)-C(10)	114.36(10)
C(2)-C(1)-Bi(1)	118.9(2)	C(2)-C(1)-C(6)	122.9(3)
C(6)-C(1)-Bi(1)	118.1(2)	C(1)-C(2)-C(7)	125.7(3)
C(3)-C(2)-C(1)	117.2(3)	C(3)-C(2)-C(7)	117.1(3)

C(2)-C(3)-H(3)	119.0	C(4)-C(3)-C(2)	122.0(3)
C(4)-C(3)-H(3)	119.0	C(3)-C(4)-C(8)	119.9(3)
C(5)-C(4)-C(3)	118.9(3)	C(5)-C(4)-C(8)	121.2(3)
C(4)-C(5)-H(5)	119.0	C(4)-C(5)-C(6)	122.0(3)
C(6)-C(5)-H(5)	119.0	C(1)-C(6)-C(5)	116.9(3)
C(1)-C(6)-C(9)	126.0(3)	C(5)-C(6)-C(9)	117.1(3)
C(2)-C(7)-H(7A)	109.5	C(2)-C(7)-H(7B)	109.5
C(2)-C(7)-H(7C)	109.5	H(7A)-C(7)-H(7B)	109.5
H(7A)-C(7)-H(7C)	109.5	H(7B)-C(7)-H(7C)	109.5
C(4)-C(8)-H(8A)	109.5	C(4)-C(8)-H(8B)	109.5
C(4)-C(8)-H(8C)	109.5	H(8A)-C(8)-H(8B)	109.5
H(8A)-C(8)-H(8C)	109.5	H(8B)-C(8)-H(8C)	109.5
C(6)-C(9)-H(9A)	109.5	C(6)-C(9)-H(9B)	109.5
C(6)-C(9)-H(9C)	109.5	H(9A)-C(9)-H(9B)	109.5
H(9A)-C(9)-H(9C)	109.5	H(9B)-C(9)-H(9C)	109.5
C(11)-C(10)-Bi(1)	119.4(2)	C(11)-C(10)-C(15)	122.2(3)
C(15)-C(10)-Bi(1)	118.1(2)	C(10)-C(11)-C(16)	125.4(3)
C(12)-C(11)-C(10)	117.0(3)	C(12)-C(11)-C(16)	117.5(3)
C(11)-C(12)-H(12)	118.7	C(13)-C(12)-C(11)	122.6(3)
C(13)-C(12)-H(12)	118.7	C(12)-C(13)-C(17)	121.0(4)
C(14)-C(13)-C(12)	118.3(3)	C(14)-C(13)-C(17)	120.7(4)
C(13)-C(14)-H(14)	118.9	C(13)-C(14)-C(15)	122.1(3)
C(15)-C(14)-H(14)	118.9	C(10)-C(15)-C(18)	125.6(3)
C(14)-C(15)-C(10)	117.6(3)	C(14)-C(15)-C(18)	116.9(3)
C(11)-C(16)-H(16A)	109.5	C(11)-C(16)-H(16B)	109.5
C(11)-C(16)-H(16C)	109.5	H(16A)-C(16)-H(16B)	109.5
H(16A)-C(16)-H(16C)	109.5	H(16B)-C(16)-H(16C)	109.5
C(13)-C(17)-H(17A)	109.5	C(13)-C(17)-H(17B)	109.5
C(13)-C(17)-H(17C)	109.5	H(17A)-C(17)-H(17B)	109.5
H(17A)-C(17)-H(17C)	109.5	H(17B)-C(17)-H(17C)	109.5
C(15)-C(18)-H(18A)	109.5	C(15)-C(18)-H(18B)	109.5
C(15)-C(18)-H(18C)	109.5	H(18A)-C(18)-H(18B)	109.5
H(18A)-C(18)-H(18C)	109.5	H(18B)-C(18)-H(18C)	109.5
C(20)-C(19)-Bi(1)	118.4(2)	C(24)-C(19)-Bi(1)	118.81(19)
C(24)-C(19)-C(20)	122.4(3)	C(19)-C(20)-C(25)	126.0(3)
C(21)-C(20)-C(19)	117.0(3)	C(21)-C(20)-C(25)	116.9(2)
C(20)-C(21)-H(21)	118.7	C(22)-C(21)-C(20)	122.6(3)
C(22)-C(21)-H(21)	118.7	C(21)-C(22)-C(23)	118.2(3)

C(21)-C(22)-C(26)	120.3(3)	C(23)-C(22)-C(26)	121.5(3)
C(22)-C(23)-H(23)	119.0	C(22)-C(23)-C(24)	122.1(3)
C(24)-C(23)-H(23)	119.0	C(19)-C(24)-C(23)	117.6(3)
C(19)-C(24)-C(27)	125.8(2)	C(23)-C(24)-C(27)	116.6(3)
C(20)-C(25)-H(25A)	109.5	C(20)-C(25)-H(25B)	109.5
C(20)-C(25)-H(25C)	109.5	H(25A)-C(25)-H(25B)	109.5
H(25A)-C(25)-H(25C)	109.5	H(25B)-C(25)-H(25C)	109.5
C(22)-C(26)-H(26A)	109.5	C(22)-C(26)-H(26B)	109.5
C(22)-C(26)-H(26C)	109.5	H(26A)-C(26)-H(26B)	109.5
H(26A)-C(26)-H(26C)	109.5	H(26B)-C(26)-H(26C)	109.5
C(24)-C(27)-H(27A)	109.5	C(24)-C(27)-H(27B)	109.5
C(24)-C(27)-H(27C)	109.5	H(27A)-C(27)-H(27B)	109.5
H(27A)-C(27)-H(27C)	109.5	H(27B)-C(27)-H(27C)	109.5
Cl(3)-Bi(2)-Cl(4)	176.23(3)	C(31)-Bi(2)-Cl(3)	90.16(8)
C(31)-Bi(2)-Cl(4)	89.62(8)	C(40)-Bi(2)-Cl(3)	90.10(8)
C(40)-Bi(2)-Cl(4)	93.38(8)	C(40)-Bi(2)-C(31)	116.00(11)
C(40)-Bi(2)-C(49)	119.05(11)	C(49)-Bi(2)-Cl(3)	89.59(8)
C(49)-Bi(2)-Cl(4)	87.44(8)	C(49)-Bi(2)-C(31)	124.95(11)
C(32)-C(31)-Bi(2)	119.2(2)	C(32)-C(31)-C(36)	122.3(3)
C(36)-C(31)-Bi(2)	118.4(2)	C(31)-C(32)-C(33)	117.4(3)
C(31)-C(32)-C(37)	125.2(3)	C(33)-C(32)-C(37)	117.4(3)
C(32)-C(33)-H(33)	118.8	C(34)-C(33)-C(32)	122.5(3)
C(34)-C(33)-H(33)	118.8	C(33)-C(34)-C(35)	117.8(3)
C(33)-C(34)-C(38)	121.8(3)	C(35)-C(34)-C(38)	120.4(3)
C(34)-C(35)-H(35)	118.6	C(34)-C(35)-C(36)	122.8(3)
C(36)-C(35)-H(35)	118.6	C(31)-C(36)-C(39)	125.9(3)
C(35)-C(36)-C(31)	117.1(3)	C(35)-C(36)-C(39)	117.0(3)
C(32)-C(37)-H(37A)	109.5	C(32)-C(37)-H(37B)	109.5
C(32)-C(37)-H(37C)	109.5	H(37A)-C(37)-H(37B)	109.5
H(37A)-C(37)-H(37C)	109.5	H(37B)-C(37)-H(37C)	109.5
C(34)-C(38)-H(38A)	109.5	C(34)-C(38)-H(38B)	109.5
C(34)-C(38)-H(38C)	109.5	H(38A)-C(38)-H(38B)	109.5
H(38A)-C(38)-H(38C)	109.5	H(38B)-C(38)-H(38C)	109.5
C(36)-C(39)-H(39A)	109.5	C(36)-C(39)-H(39B)	109.5
C(36)-C(39)-H(39C)	109.5	H(39A)-C(39)-H(39B)	109.5
H(39A)-C(39)-H(39C)	109.5	H(39B)-C(39)-H(39C)	109.5
C(41)-C(40)-Bi(2)	117.4(2)	C(45)-C(40)-Bi(2)	119.7(2)
C(45)-C(40)-C(41)	122.8(3)	C(40)-C(41)-C(46)	125.3(3)

C(42)-C(41)-C(40)	116.9(3)	C(42)-C(41)-C(46)	117.7(3)
C(41)-C(42)-H(42)	118.9	C(43)-C(42)-C(41)	122.2(4)
C(43)-C(42)-H(42)	118.9	C(42)-C(43)-C(44)	119.0(3)
C(42)-C(43)-C(47)	121.0(4)	C(44)-C(43)-C(47)	120.0(4)
C(43)-C(44)-H(44)	118.6	C(43)-C(44)-C(45)	122.8(3)
C(45)-C(44)-H(44)	118.6	C(40)-C(45)-C(44)	116.4(3)
C(40)-C(45)-C(48)	126.2(3)	C(44)-C(45)-C(48)	117.4(3)
C(41)-C(46)-H(46A)	109.5	C(41)-C(46)-H(46B)	109.5
C(41)-C(46)-H(46C)	109.5	H(46A)-C(46)-H(46B)	109.5
H(46A)-C(46)-H(46C)	109.5	H(46B)-C(46)-H(46C)	109.5
C(43)-C(47)-H(47A)	109.5	C(43)-C(47)-H(47B)	109.5
C(43)-C(47)-H(47C)	109.5	H(47A)-C(47)-H(47B)	109.5
H(47A)-C(47)-H(47C)	109.5	H(47B)-C(47)-H(47C)	109.5
C(45)-C(48)-H(48A)	109.5	C(45)-C(48)-H(48B)	109.5
C(45)-C(48)-H(48C)	109.5	H(48A)-C(48)-H(48B)	109.5
H(48A)-C(48)-H(48C)	109.5	H(48B)-C(48)-H(48C)	109.5
C(50)-C(49)-Bi(2)	118.1(2)	C(54)-C(49)-Bi(2)	119.2(2)
C(54)-C(49)-C(50)	122.7(3)	C(49)-C(50)-C(55)	125.6(3)
C(51)-C(50)-C(49)	116.9(3)	C(51)-C(50)-C(55)	117.5(3)
C(50)-C(51)-H(51)	118.8	C(52)-C(51)-C(50)	122.3(3)
C(52)-C(51)-H(51)	118.8	C(51)-C(52)-C(56)	120.8(3)
C(53)-C(52)-C(51)	118.5(3)	C(53)-C(52)-C(56)	120.7(4)
C(52)-C(53)-H(53)	118.7	C(52)-C(53)-C(54)	122.5(3)
C(54)-C(53)-H(53)	118.7	C(49)-C(54)-C(57)	125.8(3)
C(53)-C(54)-C(49)	117.1(3)	C(53)-C(54)-C(57)	117.1(3)
C(50)-C(55)-H(55A)	109.5	C(50)-C(55)-H(55B)	109.5
C(50)-C(55)-H(55C)	109.5	H(55A)-C(55)-H(55B)	109.5
H(55A)-C(55)-H(55C)	109.5	H(55B)-C(55)-H(55C)	109.5
C(52)-C(56)-H(56A)	109.5	C(52)-C(56)-H(56B)	109.5
C(52)-C(56)-H(56C)	109.5	H(56A)-C(56)-H(56B)	109.5
H(56A)-C(56)-H(56C)	109.5	H(56B)-C(56)-H(56C)	109.5
C(54)-C(57)-H(57A)	109.5	C(54)-C(57)-H(57B)	109.5
C(54)-C(57)-H(57C)	109.5	H(57A)-C(57)-H(57B)	109.5
H(57A)-C(57)-H(57C)	109.5	H(57B)-C(57)-H(57C)	109.5

9.6 Single crystal structure analysis of (µ-chloro)-dichloro-hexaphenyl-di-bismuth tetrakis(3,5-bis(trifluoromethyl)phenyl)borate (9a)

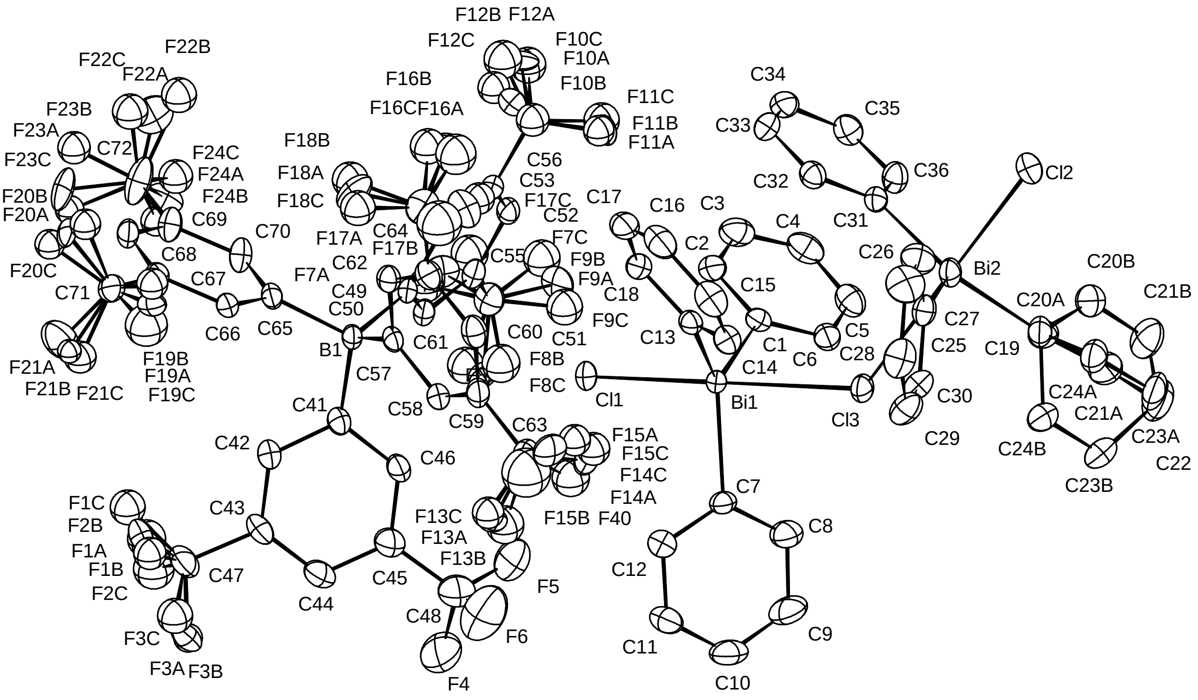

Figure S24: The solid state structure of **9a**. H atoms have been removed for clarity and disordered parts shown in grey.

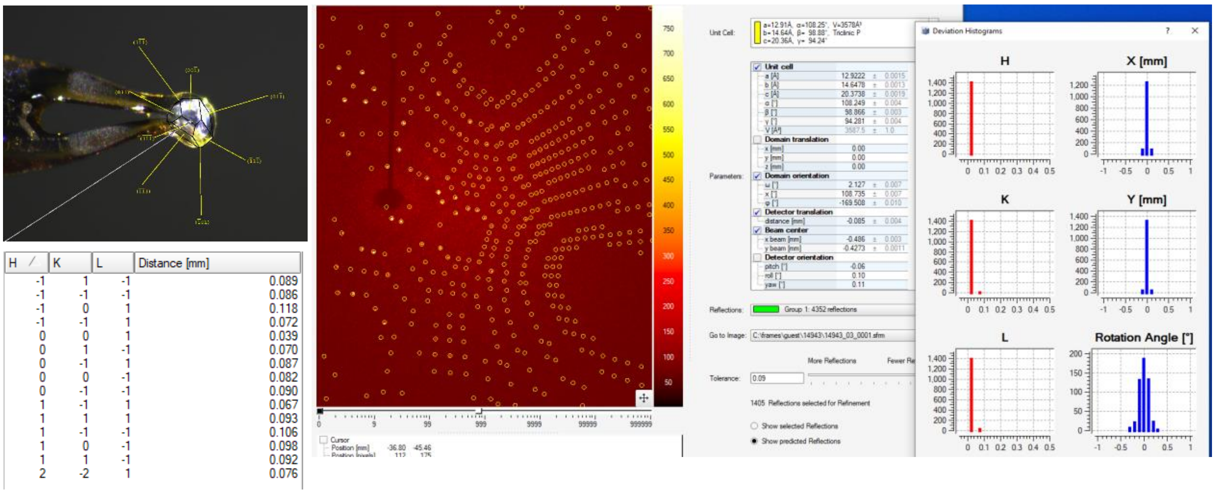

Figure S25: Crystal faces and unit cell determination/refinement of **9a**.

INTENSITY STATISTICS FOR DATASET # 1 14943sadabs.raw

Resolution	#Data	#Theory	%Complete	Redundancy	Mean I	Mean I/s	Rmerge	Rsigma
Inf - 2.62	412	418	98.6	26.70	145.34	60.26	0.0378	0.0380
2.62 - 1.76	955	957	99.8	28.28	111.01	63.09	0.0411	0.0276
1.76 - 1.40	1360	1360	100.0	29.95	70.61	62.09	0.0431	0.0169
1.40 - 1.22	1391	1391	100.0	31.11	51.99	59.28	0.0466	0.0132
1.22 - 1.11	1349	1349	100.0	30.06	42.87	53.47	0.0507	0.0135
1.11 - 1.03	1388	1388	100.0	29.14	34.69	47.08	0.0581	0.0148
1.03 - 0.97	1340	1340	100.0	28.45	28.19	41.79	0.0677	0.0165
0.97 - 0.92	1384	1384	100.0	27.98	25.38	38.49	0.0737	0.0177
0.92 - 0.88	1403	1403	100.0	26.92	19.68	32.96	0.0890	0.0207
0.88 - 0.84	1655	1655	100.0	25.58	17.71	30.06	0.1008	0.0233
0.84 - 0.81	1437	1437	100.0	25.22	15.67	27.38	0.1116	0.0258
0.81 - 0.79	1148	1148	100.0	24.69	14.00	25.45	0.1230	0.0286
0.79 - 0.77	1168	1168	100.0	24.10	12.26	22.95	0.1333	0.0319
0.77 - 0.75	1358	1358	100.0	23.49	10.82	20.50	0.1438	0.0355
0.75 - 0.73	1487	1487	100.0	22.97	10.15	19.27	0.1519	0.0385
0.73 - 0.71	1700	1700	100.0	22.17	9.31	17.40	0.1596	0.0427
0.71 - 0.70	882	882	100.0	21.50	8.45	16.15	0.1717	0.0475
0.70 - 0.68	1978	1978	100.0	21.06	7.38	14.05	0.1839	0.0537
0.68 - 0.67	1103	1103	100.0	19.94	6.80	12.67	0.1926	0.0604
0.67 - 0.66	1160	1160	100.0	19.88	6.35	11.82	0.2049	0.0653
0.66 - 0.65	1188	1254	94.7	17.42	5.22	9.61	0.2288	0.0897
0.75 - 0.65	9498	9564	99.3	20.85	7.80	14.68	0.1763	0.0525
Inf - 0.65	27246	27320	99.7	25.04	26.12	31.47	0.0759	0.0248

Complete .cif-data of the compound are available under the CCDC number CCDC-**2289343**.

Table S12. Crystal data and structure refinement.

Identification code	14943sadabs	
Empirical formula	C68 H42 B Bi2 Cl3 F24	
Color	colourless	
Formula weight	1850.13 g·mol^{-1}	
Temperature	100(2) K	
Wavelength	0.71073 Å	
Crystal system	Triclinic	
Space group	P-1, (no. 2)	
Unit cell dimensions	a = 12.9043(18) Å	α= 108.297(6)°.
	b = 14.639(2) Å	β= 98.848(7)°.
	c = 20.359(3) Å	γ = 94.297(7)°.
Volume	3576.9(9) Å^3	
Z	2	
Density (calculated)	1.718 Mg·m^{-3}	
Absorption coefficient	5.126 mm^{-1}	
F(000)	1776 e	
Crystal size	0.202 x 0.185 x 0.173 mm^3	
θ range for data collection	1.760 to 27.500°.	
Index ranges	-16 ≤ h ≤ 16, -19≤ k ≤ 19, -26 ≤ l ≤ 26	
Reflections collected	453473	
Independent reflections	16412 [R_{int} = 0.0637]	
Reflections with I>2σ(I)	15218	
Completeness to θ = 25.242°	99.9 %	
Absorption correction	Gaussian	
Max. and min. transmission	0.9604 and 0.4447	
Refinement method	Full-matrix least-squares on F^2	
Data / restraints / parameters	16412 / 90 / 1087	
Goodness-of-fit on F^2	1.028	
Final R indices [I>2σ(I)]	R_1 = 0.0217	wR^2 = 0.0505
R indices (all data)	R_1 = 0.0244	wR^2 = 0.0522
Extinction coefficient	n/a	
Largest diff. peak and hole	1.331 and -1.677 e·Å^{-3}	

Table S13. Bond lengths [Å] and angles [°].

Bi(1)-Cl(1)	2.4856(7)	Bi(1)-Cl(3)	2.7995(7)
Bi(1)-C(1)	2.212(3)	Bi(1)-C(7)	2.204(2)
Bi(1)-C(13)	2.211(3)	Bi(2)-Cl(2)	2.4996(8)
Bi(2)-Cl(3)	2.8274(7)	Bi(2)-C(19)	2.187(3)
Bi(2)-C(25)	2.189(3)	Bi(2)-C(31)	2.185(3)
F(1A)-C(47)	1.290(6)	F(1B)-C(47)	1.318(12)
F(1C)-C(47)	1.544(10)	F(2A)-C(47)	1.351(11)
F(2B)-C(47)	1.27(2)	F(2C)-C(47)	1.28(3)
F(3A)-C(47)	1.384(5)	F(3B)-C(47)	1.417(9)
F(3C)-C(47)	1.293(9)	F(4)-C(48)	1.335(5)
F(5)-C(48)	1.257(5)	F(6)-C(48)	1.392(6)
F(7A)-C(55)	1.341(7)	F(7B)-C(55)	1.41(2)
F(7C)-C(55)	1.301(12)	F(8A)-C(55)	1.392(7)
F(8B)-C(55)	1.192(17)	F(8C)-C(55)	1.323(16)
F(9A)-C(55)	1.339(5)	F(9B)-C(55)	1.343(13)
F(9C)-C(55)	1.393(14)	F(10A)-C(56)	1.341(6)
F(10B)-C(56)	1.455(16)	F(10C)-C(56)	1.252(11)
F(11A)-C(56)	1.369(6)	F(11B)-C(56)	1.307(11)
F(11C)-C(56)	1.337(14)	F(12A)-C(56)	1.329(5)
F(12B)-C(56)	1.320(10)	F(12C)-C(56)	1.435(10)
F(13A)-C(63)	1.331(5)	F(13B)-C(63)	1.469(11)
F(13C)-C(63)	1.329(9)	F(14A)-C(63)	1.352(5)
F(14C)-C(63)	1.271(10)	F(15A)-C(63)	1.371(5)
F(15B)-C(63)	1.299(16)	F(15C)-C(63)	1.352(9)
F(16A)-C(64)	1.422(10)	F(16B)-C(64)	1.21(2)
F(16C)-C(64)	1.28(2)	F(17A)-C(64)	1.356(6)
F(17B)-C(64)	1.335(16)	F(17C)-C(64)	1.359(16)
F(18A)-C(64)	1.328(7)	F(18B)-C(64)	1.402(18)
F(18C)-C(64)	1.343(18)	F(19A)-C(71)	1.354(5)
F(19B)-C(71)	1.3792(11)	F(19C)-C(71)	1.3796(11)
F(20A)-C(71)	1.329(4)	F(20B)-C(71)	1.3798(10)
F(20C)-C(71)	1.3796(10)	F(21A)-C(71)	1.350(6)
F(21B)-C(71)	1.3787(10)	F(21C)-C(71)	1.3799(10)
F(22A)-C(72)	1.231(8)	F(22B)-C(72)	1.720(11)
F(22C)-C(72)	1.314(15)	F(23A)-C(72)	1.382(6)
F(23B)-C(72)	1.322(9)	F(23C)-C(72)	1.391(12)

F(24A)-C(72)	1.333(6)	F(24B)-C(72)	1.472(12)
F(24C)-C(72)	1.247(13)	F(40)-C(63)	1.383(12)
C(1)-C(2)	1.386(4)	C(1)-C(6)	1.384(4)
C(2)-H(2)	0.9500	C(2)-C(3)	1.386(4)
C(3)-H(3)	0.9500	C(3)-C(4)	1.384(5)
C(4)-H(4)	0.9500	C(4)-C(5)	1.383(5)
C(5)-H(5)	0.9500	C(5)-C(6)	1.387(4)
C(6)-H(6)	0.9500	C(7)-C(8)	1.378(4)
C(7)-C(12)	1.378(4)	C(8)-H(8)	0.9500
C(8)-C(9)	1.389(4)	C(9)-H(9)	0.9500
C(9)-C(10)	1.374(5)	C(10)-H(10)	0.9500
C(10)-C(11)	1.369(5)	C(11)-H(11)	0.9500
C(11)-C(12)	1.391(4)	C(12)-H(12)	0.9500
C(13)-C(14)	1.381(4)	C(13)-C(18)	1.387(4)
C(14)-H(14)	0.9500	C(14)-C(15)	1.394(4)
C(15)-H(15)	0.9500	C(15)-C(16)	1.386(5)
C(16)-H(16)	0.9500	C(16)-C(17)	1.386(5)
C(17)-H(17)	0.9500	C(17)-C(18)	1.391(4)
C(18)-H(18)	0.9500	C(19)-C(20A)	1.343(5)
C(19)-C(20B)	1.343(11)	C(19)-C(24A)	1.383(5)
C(19)-C(24B)	1.558(11)	C(20A)-H(20A)	0.9500
C(20A)-C(21A)	1.394(6)	C(20B)-H(20B)	0.9500
C(20B)-C(21B)	1.392(15)	C(21A)-H(21A)	0.9500
C(21A)-C(22)	1.343(7)	C(21B)-H(21B)	0.9500
C(21B)-C(22)	1.182(13)	C(22)-H(22)	0.9500
C(22)-H(22A)	0.9500	C(22)-C(23A)	1.455(7)
C(22)-C(23B)	1.504(13)	C(23A)-H(23A)	0.9500
C(23A)-C(24A)	1.389(6)	C(23B)-H(23B)	0.9500
C(23B)-C(24B)	1.387(15)	C(24A)-H(24A)	0.9500
C(24B)-H(24B)	0.9500	C(25)-C(26)	1.378(4)
C(25)-C(30)	1.384(4)	C(26)-H(26)	0.9500
C(26)-C(27)	1.389(5)	C(27)-H(27)	0.9500
C(27)-C(28)	1.380(5)	C(28)-H(28)	0.9500
C(28)-C(29)	1.379(5)	C(29)-H(29)	0.9500
C(29)-C(30)	1.386(4)	C(30)-H(30)	0.9500
C(31)-C(32)	1.383(4)	C(31)-C(36)	1.390(4)
C(32)-H(32)	0.9500	C(32)-C(33)	1.390(4)
C(33)-H(33)	0.9500	C(33)-C(34)	1.383(4)

C(34)-H(34)	0.9500	C(34)-C(35)	1.384(4)
C(35)-H(35)	0.9500	C(35)-C(36)	1.383(4)
C(36)-H(36)	0.9500	C(41)-C(42)	1.404(3)
C(41)-C(46)	1.410(4)	C(41)-B(1)	1.639(4)
C(42)-H(42)	0.9500	C(42)-C(43)	1.398(4)
C(43)-C(44)	1.373(4)	C(43)-C(47)	1.495(4)
C(44)-H(44)	0.9500	C(44)-C(45)	1.391(4)
C(45)-C(46)	1.386(4)	C(45)-C(48)	1.509(5)
C(46)-H(46)	0.9500	C(49)-C(50)	1.400(4)
C(49)-C(54)	1.410(3)	C(49)-B(1)	1.653(4)
C(50)-H(50)	0.9500	C(50)-C(51)	1.397(4)
C(51)-C(52)	1.382(4)	C(51)-C(55)	1.496(4)
C(52)-H(52)	0.9500	C(52)-C(53)	1.387(4)
C(53)-C(54)	1.389(4)	C(53)-C(56)	1.497(4)
C(54)-H(54)	0.9500	C(57)-C(58)	1.399(4)
C(57)-C(62)	1.404(4)	C(57)-B(1)	1.657(4)
C(58)-H(58)	0.9500	C(58)-C(59)	1.395(4)
C(59)-C(60)	1.386(4)	C(59)-C(63)	1.493(4)
C(60)-H(60)	0.9500	C(60)-C(61)	1.381(4)
C(61)-C(62)	1.391(4)	C(61)-C(64)	1.493(4)
C(62)-H(62)	0.9500	C(65)-C(66)	1.404(3)
C(65)-C(70)	1.396(4)	C(65)-B(1)	1.640(4)
C(66)-H(66)	0.9500	C(66)-C(67)	1.390(3)
C(67)-C(68)	1.388(4)	C(67)-C(71)	1.488(4)
C(68)-H(68)	0.9500	C(68)-C(69)	1.377(4)
C(69)-C(70)	1.398(4)	C(69)-C(72)	1.494(4)
C(70)-H(70)	0.9500		

Cl(1)-Bi(1)-Cl(3)	179.25(2)	C(1)-Bi(1)-Cl(1)	93.53(7)
C(1)-Bi(1)-Cl(3)	87.09(7)	C(7)-Bi(1)-Cl(1)	94.22(7)
C(7)-Bi(1)-Cl(3)	85.90(7)	C(7)-Bi(1)-C(1)	115.63(10)
C(7)-Bi(1)-C(13)	123.94(10)	C(13)-Bi(1)-Cl(1)	92.44(7)
C(13)-Bi(1)-Cl(3)	86.88(7)	C(13)-Bi(1)-C(1)	119.38(9)
Cl(2)-Bi(2)-Cl(3)	177.70(2)	C(19)-Bi(2)-Cl(2)	95.06(8)
C(19)-Bi(2)-Cl(3)	83.90(8)	C(19)-Bi(2)-C(25)	115.09(10)
C(25)-Bi(2)-Cl(2)	93.91(8)	C(25)-Bi(2)-Cl(3)	88.39(7)
C(31)-Bi(2)-Cl(2)	92.99(7)	C(31)-Bi(2)-Cl(3)	85.84(7)
C(31)-Bi(2)-C(19)	122.14(11)	C(31)-Bi(2)-C(25)	121.35(10)

Bi(1)-Cl(3)-Bi(2)	128.10(2)	C(2)-C(1)-Bi(1)	117.84(19)
C(6)-C(1)-Bi(1)	119.68(19)	C(6)-C(1)-C(2)	122.3(3)
C(1)-C(2)-H(2)	120.9	C(1)-C(2)-C(3)	118.1(3)
C(3)-C(2)-H(2)	120.9	C(2)-C(3)-H(3)	119.8
C(4)-C(3)-C(2)	120.5(3)	C(4)-C(3)-H(3)	119.8
C(3)-C(4)-H(4)	119.8	C(5)-C(4)-C(3)	120.4(3)
C(5)-C(4)-H(4)	119.8	C(4)-C(5)-H(5)	119.9
C(4)-C(5)-C(6)	120.1(3)	C(6)-C(5)-H(5)	119.9
C(1)-C(6)-C(5)	118.5(3)	C(1)-C(6)-H(6)	120.8
C(5)-C(6)-H(6)	120.8	C(8)-C(7)-Bi(1)	119.0(2)
C(12)-C(7)-Bi(1)	118.6(2)	C(12)-C(7)-C(8)	122.3(3)
C(7)-C(8)-H(8)	121.0	C(7)-C(8)-C(9)	118.0(3)
C(9)-C(8)-H(8)	121.0	C(8)-C(9)-H(9)	119.7
C(10)-C(9)-C(8)	120.7(3)	C(10)-C(9)-H(9)	119.7
C(9)-C(10)-H(10)	119.9	C(11)-C(10)-C(9)	120.3(3)
C(11)-C(10)-H(10)	119.9	C(10)-C(11)-H(11)	119.7
C(10)-C(11)-C(12)	120.5(3)	C(12)-C(11)-H(11)	119.7
C(7)-C(12)-C(11)	118.2(3)	C(7)-C(12)-H(12)	120.9
C(11)-C(12)-H(12)	120.9	C(14)-C(13)-Bi(1)	120.26(19)
C(14)-C(13)-C(18)	122.8(3)	C(18)-C(13)-Bi(1)	116.9(2)
C(13)-C(14)-H(14)	121.0	C(13)-C(14)-C(15)	118.1(3)
C(15)-C(14)-H(14)	121.0	C(14)-C(15)-H(15)	119.8
C(16)-C(15)-C(14)	120.3(3)	C(16)-C(15)-H(15)	119.8
C(15)-C(16)-H(16)	119.8	C(15)-C(16)-C(17)	120.3(3)
C(17)-C(16)-H(16)	119.8	C(16)-C(17)-H(17)	119.8
C(16)-C(17)-C(18)	120.4(3)	C(18)-C(17)-H(17)	119.8
C(13)-C(18)-C(17)	118.0(3)	C(13)-C(18)-H(18)	121.0
C(17)-C(18)-H(18)	121.0	C(20A)-C(19)-Bi(2)	120.1(2)
C(20A)-C(19)-C(24A)	125.7(3)	C(20B)-C(19)-Bi(2)	125.0(5)
C(20B)-C(19)-C(24B)	113.2(7)	C(24A)-C(19)-Bi(2)	113.8(3)
C(24B)-C(19)-Bi(2)	121.0(4)	C(19)-C(20A)-H(20A)	121.6
C(19)-C(20A)-C(21A)	116.8(4)	C(21A)-C(20A)-H(20A)	121.6
C(19)-C(20B)-H(20B)	117.4	C(19)-C(20B)-C(21B)	125.1(10)
C(21B)-C(20B)-H(20B)	117.4	C(20A)-C(21A)-H(21A)	118.6
C(22)-C(21A)-C(20A)	122.9(5)	C(22)-C(21A)-H(21A)	118.6
C(20B)-C(21B)-H(21B)	120.9	C(22)-C(21B)-C(20B)	118.2(10)
C(22)-C(21B)-H(21B)	120.9	C(21A)-C(22)-H(22)	121.0
C(21A)-C(22)-C(23A)	117.9(4)	C(21B)-C(22)-H(22A)	117.3

C(21B)-C(22)-C(23B)	125.4(8)	C(23A)-C(22)-H(22)	121.0
C(23B)-C(22)-H(22A)	117.3	C(22)-C(23A)-H(23A)	120.0
C(24A)-C(23A)-C(22)	120.0(4)	C(24A)-C(23A)-H(23A)	120.0
C(22)-C(23B)-H(23B)	123.8	C(24B)-C(23B)-C(22)	112.4(9)
C(24B)-C(23B)-H(23B)	123.8	C(19)-C(24A)-C(23A)	116.3(4)
C(19)-C(24A)-H(24A)	121.8	C(23A)-C(24A)-H(24A)	121.8
C(19)-C(24B)-H(24B)	120.2	C(23B)-C(24B)-C(19)	119.6(9)
C(23B)-C(24B)-H(24B)	120.2	C(26)-C(25)-Bi(2)	118.8(2)
C(26)-C(25)-C(30)	122.6(3)	C(30)-C(25)-Bi(2)	118.5(2)
C(25)-C(26)-H(26)	120.9	C(25)-C(26)-C(27)	118.2(3)
C(27)-C(26)-H(26)	120.9	C(26)-C(27)-H(27)	119.9
C(28)-C(27)-C(26)	120.3(3)	C(28)-C(27)-H(27)	119.9
C(27)-C(28)-H(28)	119.8	C(29)-C(28)-C(27)	120.4(3)
C(29)-C(28)-H(28)	119.8	C(28)-C(29)-H(29)	119.8
C(28)-C(29)-C(30)	120.5(3)	C(30)-C(29)-H(29)	119.8
C(25)-C(30)-C(29)	118.0(3)	C(25)-C(30)-H(30)	121.0
C(29)-C(30)-H(30)	121.0	C(32)-C(31)-Bi(2)	118.63(19)
C(32)-C(31)-C(36)	122.4(2)	C(36)-C(31)-Bi(2)	118.96(19)
C(31)-C(32)-H(32)	120.9	C(31)-C(32)-C(33)	118.1(3)
C(33)-C(32)-H(32)	120.9	C(32)-C(33)-H(33)	119.8
C(34)-C(33)-C(32)	120.5(3)	C(34)-C(33)-H(33)	119.8
C(33)-C(34)-H(34)	119.8	C(33)-C(34)-C(35)	120.3(3)
C(35)-C(34)-H(34)	119.8	C(34)-C(35)-H(35)	119.8
C(36)-C(35)-C(34)	120.4(3)	C(36)-C(35)-H(35)	119.8
C(31)-C(36)-H(36)	120.8	C(35)-C(36)-C(31)	118.3(3)
C(35)-C(36)-H(36)	120.8	C(42)-C(41)-C(46)	114.9(2)
C(42)-C(41)-B(1)	123.1(2)	C(46)-C(41)-B(1)	121.7(2)
C(41)-C(42)-H(42)	118.9	C(43)-C(42)-C(41)	122.3(3)
C(43)-C(42)-H(42)	118.9	C(42)-C(43)-C(47)	119.5(3)
C(44)-C(43)-C(42)	121.2(3)	C(44)-C(43)-C(47)	119.3(3)
C(43)-C(44)-H(44)	121.0	C(43)-C(44)-C(45)	118.1(3)
C(45)-C(44)-H(44)	121.0	C(44)-C(45)-C(48)	119.5(3)
C(46)-C(45)-C(44)	120.8(3)	C(46)-C(45)-C(48)	119.6(3)
C(41)-C(46)-H(46)	118.7	C(45)-C(46)-C(41)	122.6(3)
C(45)-C(46)-H(46)	118.7	F(1A)-C(47)-F(2A)	107.2(7)
F(1A)-C(47)-F(3A)	106.1(4)	F(1A)-C(47)-C(43)	114.2(3)
F(1B)-C(47)-F(3B)	108.4(6)	F(1B)-C(47)-C(43)	112.3(6)
F(2A)-C(47)-F(3A)	106.7(8)	F(2A)-C(47)-C(43)	111.2(6)

F(2B)-C(47)-F(1B)	112.7(13)	F(2B)-C(47)-F(3B)	100.4(12)
F(2B)-C(47)-C(43)	112.4(12)	F(2C)-C(47)-F(1C)	97.3(12)
F(2C)-C(47)-F(3C)	115.9(14)	F(2C)-C(47)-C(43)	116.6(13)
F(3A)-C(47)-C(43)	111.1(3)	F(3B)-C(47)-C(43)	109.9(4)
F(3C)-C(47)-F(1C)	98.8(6)	F(3C)-C(47)-C(43)	114.1(5)
C(43)-C(47)-F(1C)	110.9(4)	F(4)-C(48)-F(6)	106.8(4)
F(4)-C(48)-C(45)	111.6(3)	F(5)-C(48)-F(4)	110.3(4)
F(5)-C(48)-F(6)	101.2(4)	F(5)-C(48)-C(45)	115.1(3)
F(6)-C(48)-C(45)	111.1(4)	C(50)-C(49)-C(54)	115.4(2)
C(50)-C(49)-B(1)	123.8(2)	C(54)-C(49)-B(1)	120.8(2)
C(49)-C(50)-H(50)	118.8	C(51)-C(50)-C(49)	122.4(2)
C(51)-C(50)-H(50)	118.8	C(50)-C(51)-C(55)	120.2(3)
C(52)-C(51)-C(50)	120.8(2)	C(52)-C(51)-C(55)	119.0(2)
C(51)-C(52)-H(52)	120.9	C(51)-C(52)-C(53)	118.2(2)
C(53)-C(52)-H(52)	120.9	C(52)-C(53)-C(54)	120.9(2)
C(52)-C(53)-C(56)	118.3(2)	C(54)-C(53)-C(56)	120.8(3)
C(49)-C(54)-H(54)	118.9	C(53)-C(54)-C(49)	122.2(2)
C(53)-C(54)-H(54)	118.9	F(7A)-C(55)-F(8A)	105.0(6)
F(7A)-C(55)-C(51)	112.4(4)	F(7B)-C(55)-C(51)	107.5(8)
F(7C)-C(55)-F(8C)	108.3(8)	F(7C)-C(55)-F(9C)	103.5(7)
F(7C)-C(55)-C(51)	114.8(6)	F(8A)-C(55)-C(51)	111.8(3)
F(8B)-C(55)-F(7B)	106.6(11)	F(8B)-C(55)-F(9B)	112.6(9)
F(8B)-C(55)-C(51)	114.2(9)	F(8C)-C(55)-F(9C)	101.0(8)
F(8C)-C(55)-C(51)	117.0(7)	F(9A)-C(55)-F(7A)	108.5(5)
F(9A)-C(55)-F(8A)	105.7(5)	F(9A)-C(55)-C(51)	113.0(3)
F(9B)-C(55)-F(7B)	103.9(8)	F(9B)-C(55)-C(51)	111.2(6)
F(9C)-C(55)-C(51)	110.5(6)	F(10A)-C(56)-F(11A)	103.5(5)
F(10A)-C(56)-C(53)	113.1(4)	F(10B)-C(56)-C(53)	107.3(6)
F(10C)-C(56)-F(11C)	108.9(8)	F(10C)-C(56)-F(12C)	104.0(7)
F(10C)-C(56)-C(53)	116.7(5)	F(11A)-C(56)-C(53)	110.6(3)
F(11B)-C(56)-F(10B)	109.9(8)	F(11B)-C(56)-F(12B)	109.4(6)
F(11B)-C(56)-C(53)	115.3(5)	F(11C)-C(56)-F(12C)	104.2(7)
F(11C)-C(56)-C(53)	110.7(6)	F(12A)-C(56)-F(10A)	107.4(5)
F(12A)-C(56)-F(11A)	107.4(4)	F(12A)-C(56)-C(53)	114.2(3)
F(12B)-C(56)-F(10B)	101.5(7)	F(12B)-C(56)-C(53)	112.6(5)
F(12C)-C(56)-C(53)	111.4(5)	C(58)-C(57)-C(62)	114.9(2)
C(58)-C(57)-B(1)	124.2(2)	C(62)-C(57)-B(1)	120.6(2)
C(57)-C(58)-H(58)	118.8	C(59)-C(58)-C(57)	122.3(3)

C(59)-C(58)-H(58)	118.8	C(58)-C(59)-C(63)	119.0(3)
C(60)-C(59)-C(58)	121.4(3)	C(60)-C(59)-C(63)	119.6(3)
C(59)-C(60)-H(60)	121.3	C(61)-C(60)-C(59)	117.3(3)
C(61)-C(60)-H(60)	121.3	C(60)-C(61)-C(62)	121.2(3)
C(60)-C(61)-C(64)	120.2(3)	C(62)-C(61)-C(64)	118.5(3)
C(57)-C(62)-H(62)	118.7	C(61)-C(62)-C(57)	122.7(2)
C(61)-C(62)-H(62)	118.7	F(13A)-C(63)-F(14A)	106.6(4)
F(13A)-C(63)-F(15A)	105.5(4)	F(13A)-C(63)-C(59)	112.4(3)
F(13B)-C(63)-C(59)	112.4(5)	F(13C)-C(63)-F(15C)	104.8(6)
F(13C)-C(63)-F(40)	109.4(7)	F(13C)-C(63)-C(59)	111.5(4)
F(14A)-C(63)-F(15A)	108.8(4)	F(14A)-C(63)-C(59)	111.7(3)
F(14C)-C(63)-F(13B)	102.2(6)	F(14C)-C(63)-F(15B)	107.8(9)
F(14C)-C(63)-C(59)	114.9(5)	F(15A)-C(63)-C(59)	111.6(3)
F(15B)-C(63)-F(13B)	103.2(8)	F(15B)-C(63)-C(59)	114.9(7)
F(15C)-C(63)-F(40)	104.3(6)	F(15C)-C(63)-C(59)	114.3(5)
F(40)-C(63)-C(59)	112.1(5)	F(16A)-C(64)-C(61)	110.2(4)
F(16B)-C(64)-F(17B)	114.1(10)	F(16B)-C(64)-F(18B)	102.3(11)
F(16B)-C(64)-C(61)	114.3(10)	F(16C)-C(64)-F(17C)	100.3(10)
F(16C)-C(64)-F(18C)	109.3(10)	F(16C)-C(64)-C(61)	115.2(9)
F(17A)-C(64)-F(16A)	106.9(7)	F(17A)-C(64)-C(61)	113.3(4)
F(17B)-C(64)-F(18B)	103.8(9)	F(17B)-C(64)-C(61)	108.8(7)
F(17C)-C(64)-C(61)	113.4(7)	F(18A)-C(64)-F(16A)	106.3(7)
F(18A)-C(64)-F(17A)	106.0(5)	F(18A)-C(64)-C(61)	113.7(4)
F(18B)-C(64)-C(61)	112.9(8)	F(18C)-C(64)-F(17C)	109.7(9)
F(18C)-C(64)-C(61)	108.6(8)	C(66)-C(65)-B(1)	121.2(2)
C(70)-C(65)-C(66)	115.6(2)	C(70)-C(65)-B(1)	122.9(2)
C(65)-C(66)-H(66)	118.9	C(67)-C(66)-C(65)	122.2(2)
C(67)-C(66)-H(66)	118.9	C(66)-C(67)-C(71)	119.9(2)
C(68)-C(67)-C(66)	121.0(2)	C(68)-C(67)-C(71)	119.0(2)
C(67)-C(68)-H(68)	121.1	C(69)-C(68)-C(67)	117.8(2)
C(69)-C(68)-H(68)	121.1	C(68)-C(69)-C(70)	121.2(3)
C(68)-C(69)-C(72)	118.4(3)	C(70)-C(69)-C(72)	120.4(3)
C(65)-C(70)-C(69)	122.1(3)	C(65)-C(70)-H(70)	118.9
C(69)-C(70)-H(70)	118.9	F(19A)-C(71)-C(67)	113.3(3)
F(19B)-C(71)-F(20B)	105.4(7)	F(19B)-C(71)-C(67)	115.5(7)
F(19C)-C(71)-F(20C)	112.4(6)	F(19C)-C(71)-F(21C)	110.0(9)
F(19C)-C(71)-C(67)	112.3(6)	F(20A)-C(71)-F(19A)	107.9(4)
F(20A)-C(71)-F(21A)	98.7(4)	F(20A)-C(71)-C(67)	113.7(3)

F(20B)-C(71)-C(67)	112.7(4)	F(20C)-C(71)-F(21C)	101.1(7)
F(20C)-C(71)-C(67)	111.9(4)	F(21A)-C(71)-F(19A)	113.0(4)
F(21A)-C(71)-C(67)	109.4(4)	F(21B)-C(71)-F(19B)	80.6(9)
F(21B)-C(71)-F(20B)	120.3(8)	F(21B)-C(71)-C(67)	117.3(5)
F(21C)-C(71)-C(67)	108.6(7)	F(22A)-C(72)-F(23A)	108.6(5)
F(22A)-C(72)-F(24A)	100.6(5)	F(22A)-C(72)-C(69)	114.3(4)
F(22C)-C(72)-F(23C)	107.8(7)	F(22C)-C(72)-C(69)	110.8(6)
F(23A)-C(72)-C(69)	109.8(4)	F(23B)-C(72)-F(22B)	87.9(6)
F(23B)-C(72)-F(24B)	117.0(7)	F(23B)-C(72)-C(69)	122.7(5)
F(23C)-C(72)-C(69)	107.7(6)	F(24A)-C(72)-F(23A)	107.6(4)
F(24A)-C(72)-C(69)	115.3(3)	F(24B)-C(72)-F(22B)	112.4(6)
F(24B)-C(72)-C(69)	107.3(5)	F(24C)-C(72)-F(22C)	102.9(8)
F(24C)-C(72)-F(23C)	109.4(7)	F(24C)-C(72)-C(69)	117.9(6)
C(69)-C(72)-F(22B)	107.4(5)	C(41)-B(1)-C(49)	111.4(2)
C(41)-B(1)-C(57)	109.4(2)	C(41)-B(1)-C(65)	108.8(2)
C(49)-B(1)-C(57)	106.48(19)	C(65)-B(1)-C(49)	110.9(2)
C(65)-B(1)-C(57)	109.7(2)		

9.7 Single crystal structure analysis of (μ-chloro)-hexakis(3,5-di-t-butylphenyl)-dichloro-di-bismuth tetrakis(3,5-bis(trifluoromethyl)phenyl)borate (11)

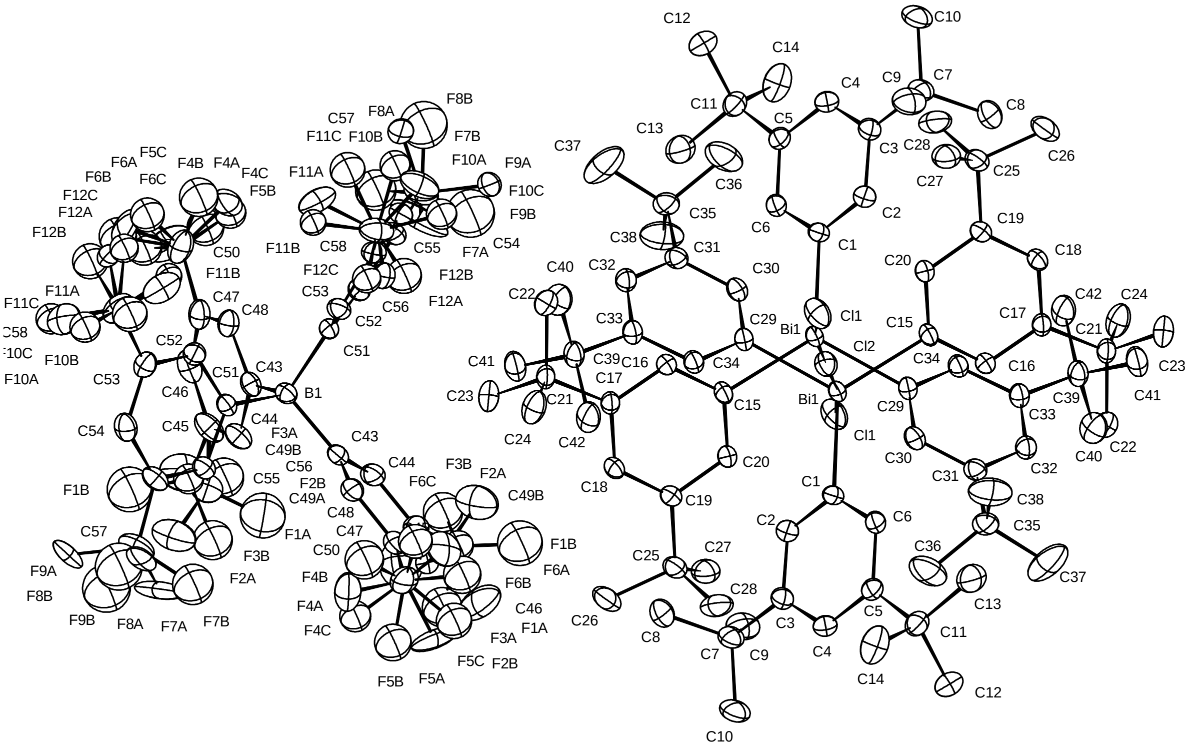

Figure S26: The solid state structure of **11**. H atoms have been removed for clarity and disordered parts shown in grey.

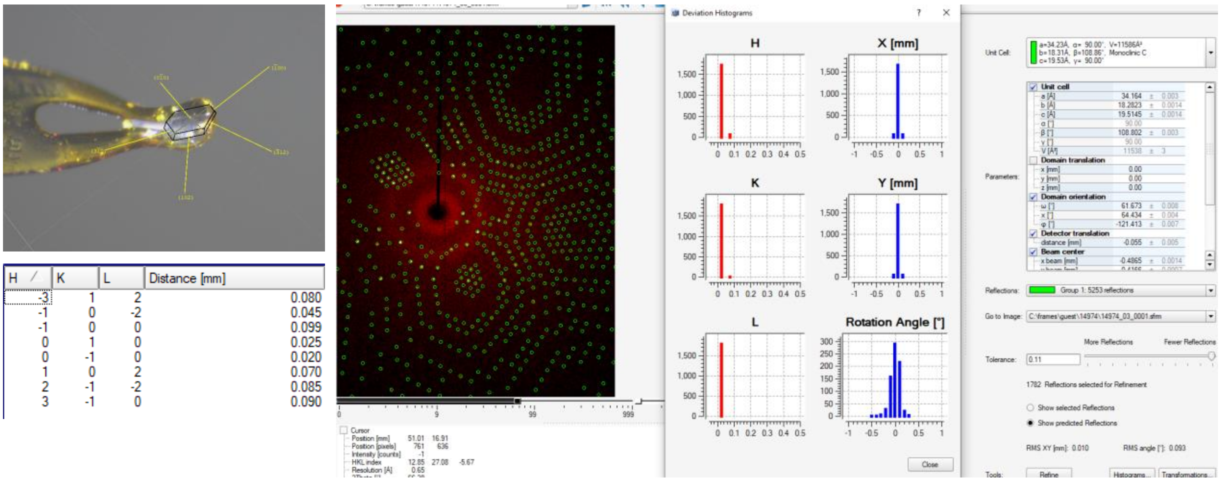

Figure S27: Crystal faces and unit cell determination/refinement of **11**.

INTENSITY STATISTICS FOR DATASET # 1 14974sadabs.raw

Resolution	#Data	#Theory	%Complete	Redundancy	Mean I	Mean I/s	Rmerge	Rsigma
Inf - 2.86	283	289	97.9	35.33	95.42	64.49	0.0405	0.0249
2.86 - 1.90	661	661	100.0	45.06	73.96	72.13	0.0380	0.0173
1.90 - 1.50	948	948	100.0	49.22	45.64	66.60	0.0442	0.0109
1.50 - 1.31	929	929	100.0	47.68	34.73	58.43	0.0562	0.0119
1.31 - 1.19	934	934	100.0	42.46	28.99	49.97	0.0657	0.0139
1.19 - 1.10	981	981	100.0	36.54	23.18	41.91	0.0761	0.0165
1.10 - 1.03	1006	1006	100.0	33.86	21.42	37.76	0.0831	0.0184
1.03 - 0.98	912	912	100.0	31.20	16.74	32.12	0.0987	0.0219
0.98 - 0.94	888	888	100.0	30.42	15.36	29.55	0.1071	0.0237
0.94 - 0.90	1029	1029	100.0	29.28	13.68	27.09	0.1172	0.0264
0.90 - 0.87	896	896	100.0	27.41	11.85	23.31	0.1321	0.0304
0.87 - 0.84	1033	1033	100.0	27.55	10.27	21.33	0.1467	0.0338
0.84 - 0.82	810	810	100.0	27.22	9.45	19.99	0.1585	0.0367
0.82 - 0.79	1313	1313	100.0	26.12	8.53	17.78	0.1726	0.0414
0.79 - 0.77	994	994	100.0	23.78	8.13	15.90	0.1828	0.0475
0.77 - 0.76	542	542	100.0	23.57	6.64	13.64	0.2097	0.0558
0.76 - 0.74	1155	1155	100.0	23.11	6.80	13.41	0.2101	0.0567
0.74 - 0.73	634	634	100.0	22.74	6.39	12.56	0.2257	0.0618
0.73 - 0.71	1367	1367	100.0	22.00	5.62	10.85	0.2477	0.0713
0.71 - 0.70	752	752	100.0	19.91	5.38	9.61	0.2647	0.0810
0.70 - 0.69	691	717	96.4	17.22	4.91	8.12	0.2945	0.1101
0.79 - 0.69	6135	6161	99.6	21.90	6.31	12.12	0.2241	0.0653
Inf - 0.69	18758	18790	99.8	30.36	18.37	29.28	0.0818	0.0247

Complete .cif-data of the compound are available under the CCDC number CCDC-**2296786.**

Table S14. Crystal data and structure refinement.

Identification code	14974	
Empirical formula	$C_{116} H_{138} B Bi_2 Cl_3 F_{24}$	
Color	colourless	
Formula weight	2523.38 g·mol^{-1}	
Temperature	100(2) K	
Wavelength	0.71073 Å	
Crystal system	Monoclinic	
Space group	*C*2/*c*, (no. 15)	
Unit cell dimensions	a = 34.1234(13) Å	α= 90°.
	b = 18.2693(7) Å	β= 108.812(2)°.
	c = 19.4972(8) Å	γ = 90°.
Volume	11505.5(8) Å^3	
Z	4	
Density (calculated)	1.457 Mg·m^{-3}	
Absorption coefficient	3.209 mm^{-1}	
F(000)	5088 e	
Crystal size	0.288 x 0.140 x 0.050 mm^3	
θ range for data collection	2.160 to 30.508°.	
Index ranges	$-48 \le h \le 48$, $-26 \le k \le 26$, $-27 \le l \le 27$	
Reflections collected	549469	
Independent reflections	17558 [R_{int} = 0.0832]	
Reflections with I>2σ(I)	15018	
Completeness to θ = 25.242°	100.0 %	
Absorption correction	Semi-empirical from equivalents	
Max. and min. transmission	0.8626 and 0.6094	
Refinement method	Full-matrix least-squares on F^2	
Data / restraints / parameters	17558 / 71 / 754	
Goodness-of-fit on F^2	1.043	
Final R indices [I>2σ(I)]	R_1 = 0.0301	wR^2 = 0.0739
R indices (all data)	R_1 = 0.0394	wR^2 = 0.0796
Extinction coefficient	n/a	
Largest diff. peak and hole	1.741 and -1.087 e·Å^{-3}	

Table S15. Bond lengths [Å] and angles [°].

Bi(1)-Cl(1)	2.4857(7)	Bi(1)-Cl(2)	2.80068(13)
Bi(1)-C(1)	2.194(2)	Bi(1)-C(15)	2.195(2)
Bi(1)-C(29)	2.188(2)	C(1)-C(2)	1.377(4)
C(1)-C(6)	1.388(4)	C(2)-H(2)	0.9500
C(2)-C(3)	1.403(4)	C(3)-C(4)	1.390(4)
C(3)-C(7)	1.534(4)	C(4)-H(4)	0.9500
C(4)-C(5)	1.409(4)	C(5)-C(6)	1.392(4)
C(5)-C(11)	1.546(4)	C(6)-H(6)	0.9500
C(7)-C(8)	1.533(4)	C(7)-C(9)	1.536(5)
C(7)-C(10)	1.536(4)	C(8)-H(8A)	0.9800
C(8)-H(8B)	0.9800	C(8)-H(8C)	0.9800
C(9)-H(9A)	0.9800	C(9)-H(9B)	0.9800
C(9)-H(9C)	0.9800	C(10)-H(10A)	0.9800
C(10)-H(10B)	0.9800	C(10)-H(10C)	0.9800
C(11)-C(12)	1.530(4)	C(11)-C(13)	1.529(4)
C(11)-C(14)	1.537(4)	C(12)-H(12A)	0.9800
C(12)-H(12B)	0.9800	C(12)-H(12C)	0.9800
C(13)-H(13A)	0.9800	C(13)-H(13B)	0.9800
C(13)-H(13C)	0.9800	C(14)-H(14A)	0.9800
C(14)-H(14B)	0.9800	C(14)-H(14C)	0.9800
C(15)-C(16)	1.398(3)	C(15)-C(20)	1.375(4)
C(16)-H(16)	0.9500	C(16)-C(17)	1.395(3)
C(17)-C(18)	1.400(4)	C(17)-C(21)	1.532(3)
C(18)-H(18)	0.9500	C(18)-C(19)	1.391(3)
C(19)-C(20)	1.405(3)	C(19)-C(25)	1.530(4)
C(20)-H(20)	0.9500	C(21)-C(22)	1.524(4)
C(21)-C(23)	1.534(4)	C(21)-C(24)	1.528(4)
C(22)-H(22A)	0.9800	C(22)-H(22B)	0.9800
C(22)-H(22C)	0.9800	C(23)-H(23A)	0.9800
C(23)-H(23B)	0.9800	C(23)-H(23C)	0.9800
C(24)-H(24A)	0.9800	C(24)-H(24B)	0.9800
C(24)-H(24C)	0.9800	C(25)-C(26)	1.536(4)
C(25)-C(27)	1.533(4)	C(25)-C(28)	1.530(4)
C(26)-H(26A)	0.9800	C(26)-H(26B)	0.9800
C(26)-H(26C)	0.9800	C(27)-H(27A)	0.9800
C(27)-H(27B)	0.9800	C(27)-H(27C)	0.9800

C(28)-H(28A)	0.9800	C(28)-H(28B)	0.9800
C(28)-H(28C)	0.9800	C(29)-C(30)	1.377(4)
C(29)-C(34)	1.385(3)	C(30)-H(30)	0.9500
C(30)-C(31)	1.399(4)	C(31)-C(32)	1.394(3)
C(31)-C(35)	1.534(4)	C(32)-H(32)	0.9500
C(32)-C(33)	1.400(3)	C(33)-C(34)	1.393(3)
C(33)-C(39)	1.536(3)	C(34)-H(34)	0.9500
C(35)-C(36)	1.522(4)	C(35)-C(37)	1.518(5)
C(35)-C(38)	1.536(5)	C(36)-H(36A)	0.9800
C(36)-H(36B)	0.9800	C(36)-H(36C)	0.9800
C(37)-H(37A)	0.9800	C(37)-H(37B)	0.9800
C(37)-H(37C)	0.9800	C(38)-H(38A)	0.9800
C(38)-H(38B)	0.9800	C(38)-H(38C)	0.9800
C(39)-C(40)	1.538(4)	C(39)-C(41)	1.533(4)
C(39)-C(42)	1.527(4)	C(40)-H(40A)	0.9800
C(40)-H(40B)	0.9800	C(40)-H(40C)	0.9800
C(41)-H(41A)	0.9800	C(41)-H(41B)	0.9800
C(41)-H(41C)	0.9800	C(42)-H(42A)	0.9800
C(42)-H(42B)	0.9800	C(42)-H(42C)	0.9800
C(43)-C(44)	1.401(4)	C(43)-C(48)	1.402(4)
C(43)-B(1)	1.646(3)	C(44)-H(44)	0.9500
C(44)-C(45)	1.392(4)	C(45)-C(46)	1.381(5)
C(45)-C(49A)	1.557(8)	C(45)-C(49B)	1.425(16)
C(46)-H(46)	0.9500	C(46)-C(47)	1.380(4)
C(47)-C(48)	1.391(4)	C(47)-C(50)	1.500(4)
C(48)-H(48)	0.9500	C(50)-F(4A)	1.334(6)
C(50)-F(5A)	1.372(6)	C(50)-F(6A)	1.330(5)
C(50)-F(4B)	1.395(13)	C(50)-F(5B)	1.297(12)
C(50)-F(6B)	1.306(13)	C(50)-F(4C)	1.270(12)
C(50)-F(5C)	1.318(15)	C(50)-F(6C)	1.323(13)
C(51)-C(52)	1.403(4)	C(51)-C(56)	1.400(3)
C(51)-B(1)	1.638(3)	C(52)-H(52)	0.9500
C(52)-C(53)	1.399(3)	C(53)-C(54)	1.379(4)
C(53)-C(58)	1.494(4)	C(54)-H(54)	0.9500
C(54)-C(55)	1.384(4)	C(55)-C(56)	1.386(4)
C(55)-C(57)	1.491(4)	C(56)-H(56)	0.9500
C(57)-F(7A)	1.266(4)	C(57)-F(8A)	1.319(4)
C(57)-F(9A)	1.423(4)	C(57)-F(7B)	

1.3825(10)	C(57)-F(8B)	1.3790(10)	C(57)-
F(9B)	1.3786(10)	C(58)-F(10A)	1.315(5)
C(58)-F(11A)	1.405(6)	C(58)-F(12A)	1.284(5)
C(58)-F(10B)	1.3789(10)	C(58)-F(11B)	1.3821(10)
C(58)-F(12B)	1.3791(10)	C(58)-F(10C)	1.3824(10)
C(58)-F(11C)	1.3778(10)	C(58)-F(12C)	1.3795(10)
F(1A)-C(49A)	1.297(11)	F(2A)-C(49A)	1.296(9)
F(3A)-C(49A)	1.313(8)	F(1B)-C(49B)	1.261(16)
F(2B)-C(49B)	1.391(16)	F(3B)-C(49B)	1.409(16)

Cl(1)-Bi(1)-Cl(2)	173.722(17)	C(1)-Bi(1)-Cl(1)	93.38(7)
C(1)-Bi(1)-Cl(2)	87.21(7)	C(1)-Bi(1)-C(15)	123.32(9)
C(15)-Bi(1)-Cl(1)	96.00(7)	C(15)-Bi(1)-Cl(2)	88.88(7)
C(29)-Bi(1)-Cl(1)	91.23(7)	C(29)-Bi(1)-Cl(2)	82.95(7)
C(29)-Bi(1)-C(1)	116.80(9)	C(29)-Bi(1)-C(15)	118.71(9)
Bi(1)#1-Cl(2)-Bi(1)	180.0	C(2)-C(1)-Bi(1)	114.50(19)
C(2)-C(1)-C(6)	123.7(2)	C(6)-C(1)-Bi(1)	121.76(18)
C(1)-C(2)-H(2)	120.5	C(1)-C(2)-C(3)	119.1(2)
C(3)-C(2)-H(2)	120.5	C(2)-C(3)-C(7)	118.1(2)
C(4)-C(3)-C(2)	117.5(2)	C(4)-C(3)-C(7)	124.3(2)
C(3)-C(4)-H(4)	118.4	C(3)-C(4)-C(5)	123.3(2)
C(5)-C(4)-H(4)	118.4	C(4)-C(5)-C(11)	119.8(2)
C(6)-C(5)-C(4)	118.2(2)	C(6)-C(5)-C(11)	122.0(2)
C(1)-C(6)-C(5)	118.3(2)	C(1)-C(6)-H(6)	120.9
C(5)-C(6)-H(6)	120.9	C(3)-C(7)-C(9)	108.7(2)
C(3)-C(7)-C(10)	111.9(2)	C(8)-C(7)-C(3)	109.9(2)
C(8)-C(7)-C(9)	109.1(3)	C(8)-C(7)-C(10)	108.6(3)
C(10)-C(7)-C(9)	108.7(3)	C(7)-C(8)-H(8A)	109.5
C(7)-C(8)-H(8B)	109.5	C(7)-C(8)-H(8C)	109.5
H(8A)-C(8)-H(8B)	109.5	H(8A)-C(8)-H(8C)	109.5
H(8B)-C(8)-H(8C)	109.5	C(7)-C(9)-H(9A)	109.5
C(7)-C(9)-H(9B)	109.5	C(7)-C(9)-H(9C)	109.5
H(9A)-C(9)-H(9B)	109.5	H(9A)-C(9)-H(9C)	109.5
H(9B)-C(9)-H(9C)	109.5	C(7)-C(10)-H(10A)	109.5
C(7)-C(10)-H(10B)	109.5	C(7)-C(10)-H(10C)	109.5
H(10A)-C(10)-H(10B)	109.5	H(10A)-C(10)-H(10C)	109.5
H(10B)-C(10)-H(10C)	109.5	C(12)-C(11)-C(5)	110.5(2)
C(12)-C(11)-C(14)	108.8(3)	C(13)-C(11)-C(5)	111.8(2)

C(13)-C(11)-C(12)	108.2(2)	C(13)-C(11)-C(14)	109.2(3)
C(14)-C(11)-C(5)	108.3(2)	C(11)-C(12)-H(12A)	109.5
C(11)-C(12)-H(12B)	109.5	C(11)-C(12)-H(12C)	109.5
H(12A)-C(12)-H(12B)	109.5	H(12A)-C(12)-H(12C)	109.5
H(12B)-C(12)-H(12C)	109.5	C(11)-C(13)-H(13A)	109.5
C(11)-C(13)-H(13B)	109.5	C(11)-C(13)-H(13C)	109.5
H(13A)-C(13)-H(13B)	109.5	H(13A)-C(13)-H(13C)	109.5
H(13B)-C(13)-H(13C)	109.5	C(11)-C(14)-H(14A)	109.5
C(11)-C(14)-H(14B)	109.5	C(11)-C(14)-H(14C)	109.5
H(14A)-C(14)-H(14B)	109.5	H(14A)-C(14)-H(14C)	109.5
H(14B)-C(14)-H(14C)	109.5	C(16)-C(15)-Bi(1)	119.42(18)
C(20)-C(15)-Bi(1)	116.55(17)	C(20)-C(15)-C(16)	123.9(2)
C(15)-C(16)-H(16)	121.0	C(17)-C(16)-C(15)	118.0(2)
C(17)-C(16)-H(16)	121.0	C(16)-C(17)-C(18)	118.1(2)
C(16)-C(17)-C(21)	122.3(2)	C(18)-C(17)-C(21)	119.6(2)
C(17)-C(18)-H(18)	118.2	C(19)-C(18)-C(17)	123.6(2)
C(19)-C(18)-H(18)	118.2	C(18)-C(19)-C(20)	117.8(2)
C(18)-C(19)-C(25)	123.0(2)	C(20)-C(19)-C(25)	119.2(2)
C(15)-C(20)-C(19)	118.6(2)	C(15)-C(20)-H(20)	120.7
C(19)-C(20)-H(20)	120.7	C(17)-C(21)-C(23)	110.3(2)
C(22)-C(21)-C(17)	112.1(2)	C(22)-C(21)-C(23)	108.4(2)
C(22)-C(21)-C(24)	108.8(3)	C(24)-C(21)-C(17)	108.8(2)
C(24)-C(21)-C(23)	108.3(2)	C(21)-C(22)-H(22A)	109.5
C(21)-C(22)-H(22B)	109.5	C(21)-C(22)-H(22C)	109.5
H(22A)-C(22)-H(22B)	109.5	H(22A)-C(22)-H(22C)	109.5
H(22B)-C(22)-H(22C)	109.5	C(21)-C(23)-H(23A)	109.5
C(21)-C(23)-H(23B)	109.5	C(21)-C(23)-H(23C)	109.5
H(23A)-C(23)-H(23B)	109.5	H(23A)-C(23)-H(23C)	109.5
H(23B)-C(23)-H(23C)	109.5	C(21)-C(24)-H(24A)	109.5
C(21)-C(24)-H(24B)	109.5	C(21)-C(24)-H(24C)	109.5
H(24A)-C(24)-H(24B)	109.5	H(24A)-C(24)-H(24C)	109.5
H(24B)-C(24)-H(24C)	109.5	C(19)-C(25)-C(26)	111.7(2)
C(19)-C(25)-C(27)	109.8(2)	C(19)-C(25)-C(28)	108.9(2)
C(27)-C(25)-C(26)	107.2(3)	C(28)-C(25)-C(26)	109.3(3)
C(28)-C(25)-C(27)	109.9(2)	C(25)-C(26)-H(26A)	109.5
C(25)-C(26)-H(26B)	109.5	C(25)-C(26)-H(26C)	109.5
H(26A)-C(26)-H(26B)	109.5	H(26A)-C(26)-H(26C)	109.5
H(26B)-C(26)-H(26C)	109.5	C(25)-C(27)-H(27A)	109.5

C(25)-C(27)-H(27B)	109.5	C(25)-C(27)-H(27C)	109.5
H(27A)-C(27)-H(27B)	109.5	H(27A)-C(27)-H(27C)	109.5
H(27B)-C(27)-H(27C)	109.5	C(25)-C(28)-H(28A)	109.5
C(25)-C(28)-H(28B)	109.5	C(25)-C(28)-H(28C)	109.5
H(28A)-C(28)-H(28B)	109.5	H(28A)-C(28)-H(28C)	109.5
H(28B)-C(28)-H(28C)	109.5	C(30)-C(29)-Bi(1)	118.90(18)
C(30)-C(29)-C(34)	123.9(2)	C(34)-C(29)-Bi(1)	117.07(18)
C(29)-C(30)-H(30)	120.6	C(29)-C(30)-C(31)	118.7(2)
C(31)-C(30)-H(30)	120.6	C(30)-C(31)-C(35)	120.3(2)
C(32)-C(31)-C(30)	117.5(2)	C(32)-C(31)-C(35)	122.1(2)
C(31)-C(32)-H(32)	118.2	C(31)-C(32)-C(33)	123.6(2)
C(33)-C(32)-H(32)	118.2	C(32)-C(33)-C(39)	120.3(2)
C(34)-C(33)-C(32)	117.9(2)	C(34)-C(33)-C(39)	121.8(2)
C(29)-C(34)-C(33)	118.3(2)	C(29)-C(34)-H(34)	120.8
C(33)-C(34)-H(34)	120.8	C(31)-C(35)-C(38)	108.3(2)
C(36)-C(35)-C(31)	110.8(3)	C(36)-C(35)-C(38)	108.1(3)
C(37)-C(35)-C(31)	112.5(2)	C(37)-C(35)-C(36)	108.5(3)
C(37)-C(35)-C(38)	108.5(3)	C(35)-C(36)-H(36A)	109.5
C(35)-C(36)-H(36B)	109.5	C(35)-C(36)-H(36C)	109.5
H(36A)-C(36)-H(36B)	109.5	H(36A)-C(36)-H(36C)	109.5
H(36B)-C(36)-H(36C)	109.5	C(35)-C(37)-H(37A)	109.5
C(35)-C(37)-H(37B)	109.5	C(35)-C(37)-H(37C)	109.5
H(37A)-C(37)-H(37B)	109.5	H(37A)-C(37)-H(37C)	109.5
H(37B)-C(37)-H(37C)	109.5	C(35)-C(38)-H(38A)	109.5
C(35)-C(38)-H(38B)	109.5	C(35)-C(38)-H(38C)	109.5
H(38A)-C(38)-H(38B)	109.5	H(38A)-C(38)-H(38C)	109.5
H(38B)-C(38)-H(38C)	109.5	C(33)-C(39)-C(40)	108.1(2)
C(41)-C(39)-C(33)	109.4(2)	C(41)-C(39)-C(40)	110.2(2)
C(42)-C(39)-C(33)	112.0(2)	C(42)-C(39)-C(40)	109.0(2)
C(42)-C(39)-C(41)	108.2(2)	C(39)-C(40)-H(40A)	109.5
C(39)-C(40)-H(40B)	109.5	C(39)-C(40)-H(40C)	109.5
H(40A)-C(40)-H(40B)	109.5	H(40A)-C(40)-H(40C)	109.5
H(40B)-C(40)-H(40C)	109.5	C(39)-C(41)-H(41A)	109.5
C(39)-C(41)-H(41B)	109.5	C(39)-C(41)-H(41C)	109.5
H(41A)-C(41)-H(41B)	109.5	H(41A)-C(41)-H(41C)	109.5
H(41B)-C(41)-H(41C)	109.5	C(39)-C(42)-H(42A)	109.5
C(39)-C(42)-H(42B)	109.5	C(39)-C(42)-H(42C)	109.5
H(42A)-C(42)-H(42B)	109.5	H(42A)-C(42)-H(42C)	109.5

H(42B)-C(42)-H(42C)	109.5	C(44)-C(43)-C(48)	115.1(2)
C(44)-C(43)-B(1)	123.1(2)	C(48)-C(43)-B(1)	121.7(2)
C(43)-C(44)-H(44)	118.8	C(45)-C(44)-C(43)	122.3(3)
C(45)-C(44)-H(44)	118.8	C(44)-C(45)-C(49A)	116.6(4)
C(44)-C(45)-C(49B)	125.0(7)	C(46)-C(45)-C(44)	121.1(3)
C(46)-C(45)-C(49A)	121.9(4)	C(46)-C(45)-C(49B)	113.3(6)
C(45)-C(46)-H(46)	121.0	C(47)-C(46)-C(45)	117.9(3)
C(47)-C(46)-H(46)	121.0	C(46)-C(47)-C(48)	120.9(3)
C(46)-C(47)-C(50)	119.9(3)	C(48)-C(47)-C(50)	119.2(3)
C(43)-C(48)-H(48)	118.7	C(47)-C(48)-C(43)	122.6(3)
C(47)-C(48)-H(48)	118.7	F(4A)-C(50)-C(47)	112.6(3)
F(4A)-C(50)-F(5A)	107.6(5)	F(5A)-C(50)-C(47)	111.7(4)
F(6A)-C(50)-C(47)	111.9(3)	F(6A)-C(50)-F(4A)	108.1(5)
F(6A)-C(50)-F(5A)	104.5(5)	F(4B)-C(50)-C(47)	114.7(6)
F(5B)-C(50)-C(47)	115.6(6)	F(5B)-C(50)-F(4B)	103.3(8)
F(5B)-C(50)-F(6B)	105.5(8)	F(6B)-C(50)-C(47)	115.0(6)
F(6B)-C(50)-F(4B)	101.1(8)	F(4C)-C(50)-C(47)	111.9(6)
F(4C)-C(50)-F(5C)	104.7(8)	F(4C)-C(50)-F(6C)	108.8(8)
F(5C)-C(50)-C(47)	111.5(7)	F(5C)-C(50)-F(6C)	107.0(8)
F(6C)-C(50)-C(47)	112.5(6)	C(52)-C(51)-B(1)	123.0(2)
C(56)-C(51)-C(52)	115.4(2)	C(56)-C(51)-B(1)	121.6(2)
C(51)-C(52)-H(52)	118.8	C(53)-C(52)-C(51)	122.3(2)
C(53)-C(52)-H(52)	118.8	C(52)-C(53)-C(58)	119.5(2)
C(54)-C(53)-C(52)	120.7(3)	C(54)-C(53)-C(58)	119.6(2)
C(53)-C(54)-H(54)	121.0	C(53)-C(54)-C(55)	118.0(2)
C(55)-C(54)-H(54)	121.0	C(54)-C(55)-C(56)	121.3(2)
C(54)-C(55)-C(57)	119.5(3)	C(56)-C(55)-C(57)	119.1(3)
C(51)-C(56)-H(56)	118.9	C(55)-C(56)-C(51)	122.2(2)
C(55)-C(56)-H(56)	118.9	F(7A)-C(57)-C(55)	114.8(3)
F(7A)-C(57)-F(8A)	116.3(4)	F(7A)-C(57)-F(9A)	102.5(3)
F(8A)-C(57)-C(55)	111.9(3)	F(8A)-C(57)-F(9A)	101.1(3)
F(9A)-C(57)-C(55)	108.5(3)	F(7B)-C(57)-C(55)	108.1(5)
F(8B)-C(57)-C(55)	123.2(7)	F(8B)-C(57)-F(7B)	91.5(7)
F(9B)-C(57)-C(55)	122.7(6)	F(9B)-C(57)-F(7B)	93.2(7)
F(9B)-C(57)-F(8B)	108.1(8)	F(10A)-C(58)-C(53)	116.5(3)
F(10A)-C(58)-F(11A)	102.4(4)	F(11A)-C(58)-C(53)	111.0(3)
F(12A)-C(58)-C(53)	115.6(3)	F(12A)-C(58)-F(10A)	106.6(4)
F(12A)-C(58)-F(11A)	103.1(4)	F(10B)-C(58)-C(53)	113.0(4)

F(10B)-C(58)-F(11B)	96.7(6)	F(10B)-C(58)-F(12B)	118.5(7)
F(11B)-C(58)-C(53)	108.8(4)	F(12B)-C(58)-C(53)	113.0(7)
F(12B)-C(58)-F(11B)	104.6(7)	F(10C)-C(58)-C(53)	103.6(4)
F(11C)-C(58)-C(53)	111.9(5)	F(11C)-C(58)-F(10C)	100.5(6)
F(11C)-C(58)-F(12C)	119.9(7)	F(12C)-C(58)-C(53)	115.1(6)
F(12C)-C(58)-F(10C)	102.6(6)	C(43)#2-B(1)-C(43)	111.9(3)
C(51)#2-B(1)-C(43)	111.27(12)	C(51)#2-B(1)-C(43)#2	105.96(12)
C(51)-B(1)-C(43)#2	111.27(12)	C(51)-B(1)-C(43)	105.96(12)
C(51)-B(1)-C(51)#2	110.6(3)	F(1A)-C(49A)-C(45)	115.1(6)
F(1A)-C(49A)-F(3A)	105.5(7)	F(2A)-C(49A)-C(45)	110.6(5)
F(2A)-C(49A)-F(1A)	102.2(7)	F(2A)-C(49A)-F(3A)	112.3(7)
F(3A)-C(49A)-C(45)	110.9(6)	F(1B)-C(49B)-C(45)	123.4(11)
F(1B)-C(49B)-F(2B)	107.3(12)	F(1B)-C(49B)-F(3B)	104.5(12)
F(2B)-C(49B)-C(45)	110.9(11)	F(2B)-C(49B)-F(3B)	95.1(9)
F(3B)-C(49B)-C(45)	111.8(11)		

Symmetry transformations used to generate equivalent atoms:

#1 -x+3/2,-y+1/2,-z+1 #2 -x+1,y,-z+3/2

10. References

(1) Kuziola, J.; Magre, M.; Nöthling, N.; Cornella, J., *Organometallics* 2022, **41**, 1754.

(2) Yakubenko, A. A.; Puzyk, A. M.; Korostelev, V. O.; Mulloyarova, V. V.; Tupikina, E. Yu.; Tolstoy, P. M.; Antonov, A. S., *Phys. Chem. Chem. Phys.* 2022, **24**, 7882.

(3) Wenger, J. S.; Getahun, A.; Johnstone, T. C., *Polyhedron* 2024, **247**, 116730.

(4) Kuziola, J. Dissertation: Synthesis and Characterization of Mono-and Bimetallic Organobismuth(V) Compounds, Ruhr-Universität Bochum, Max-Planck Institut für Kohlenforschung, 2024.

(5) Martin, G. J.; Martin, M. L.; Gouesnard, J.-P.; Diehl, P.; Fluck, E.; Kosfeld, R.; Series Eds.; Springer Berlin Heidelberg: Berlin, Heidelberg, 1981, **18**. (Please see page 129)

(6) Wagner, J. P.; Schreiner, P. R., *Angew. Chem. Int. Ed.* 2015, **54**, 12274.

(7) Rösel, S.; Becker, J.; Allen, W. D.; Schreiner, P. R. *J. Am. Chem. Soc.* 2018, **140**, 14421–14432.

(8) Grimme, S.; Schreiner, P. R., *Angew. Chem. Int. Ed.* 2011, **50**, 12639–12642.

(9) Kratzert, D.; Holstein, J. J.; Krossing, I., *J. Appl. Crystallogr.* 2015, **48**, 933.

(10) Kratzert, D.; Krossing, I., *J. Appl. Crystallogr.* 2018, **51**, 928.

11. NMR Data

^{1}H NMR (300 MHz, $CDCl_3$)

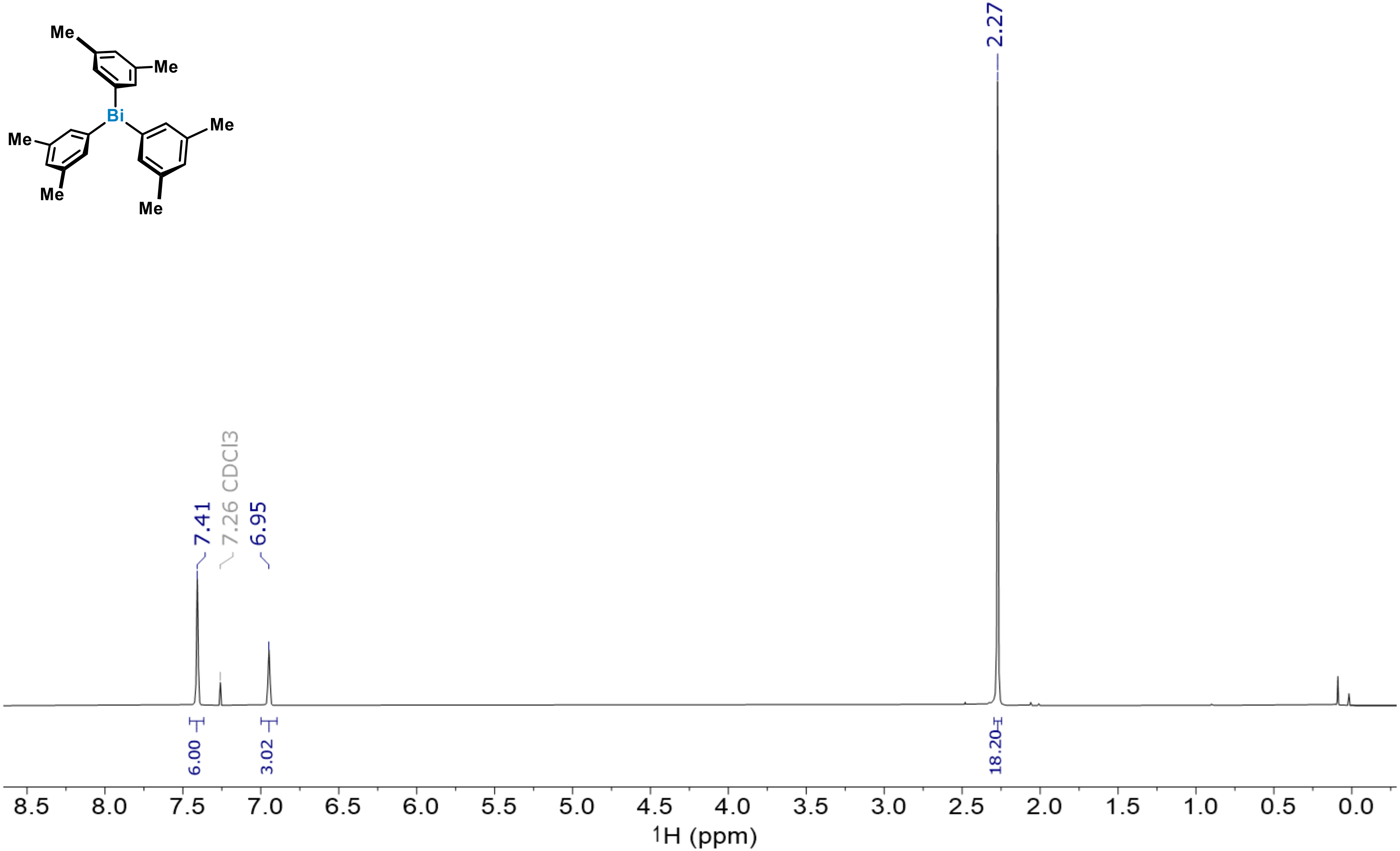

^{13}C NMR (75 MHz, $CDCl_3$) δ

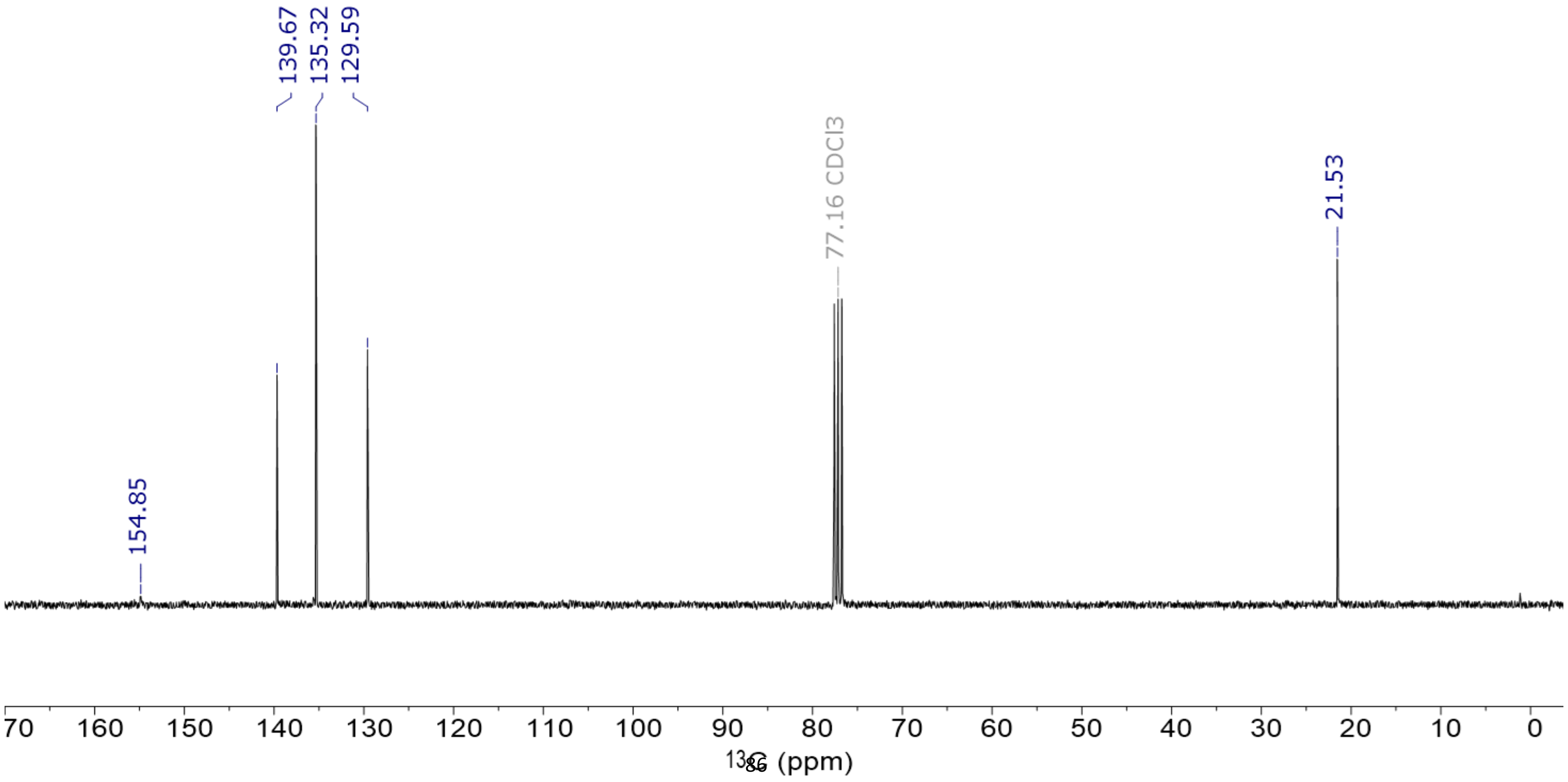

^{1}H NMR (300 MHz, $CDCl_3$)

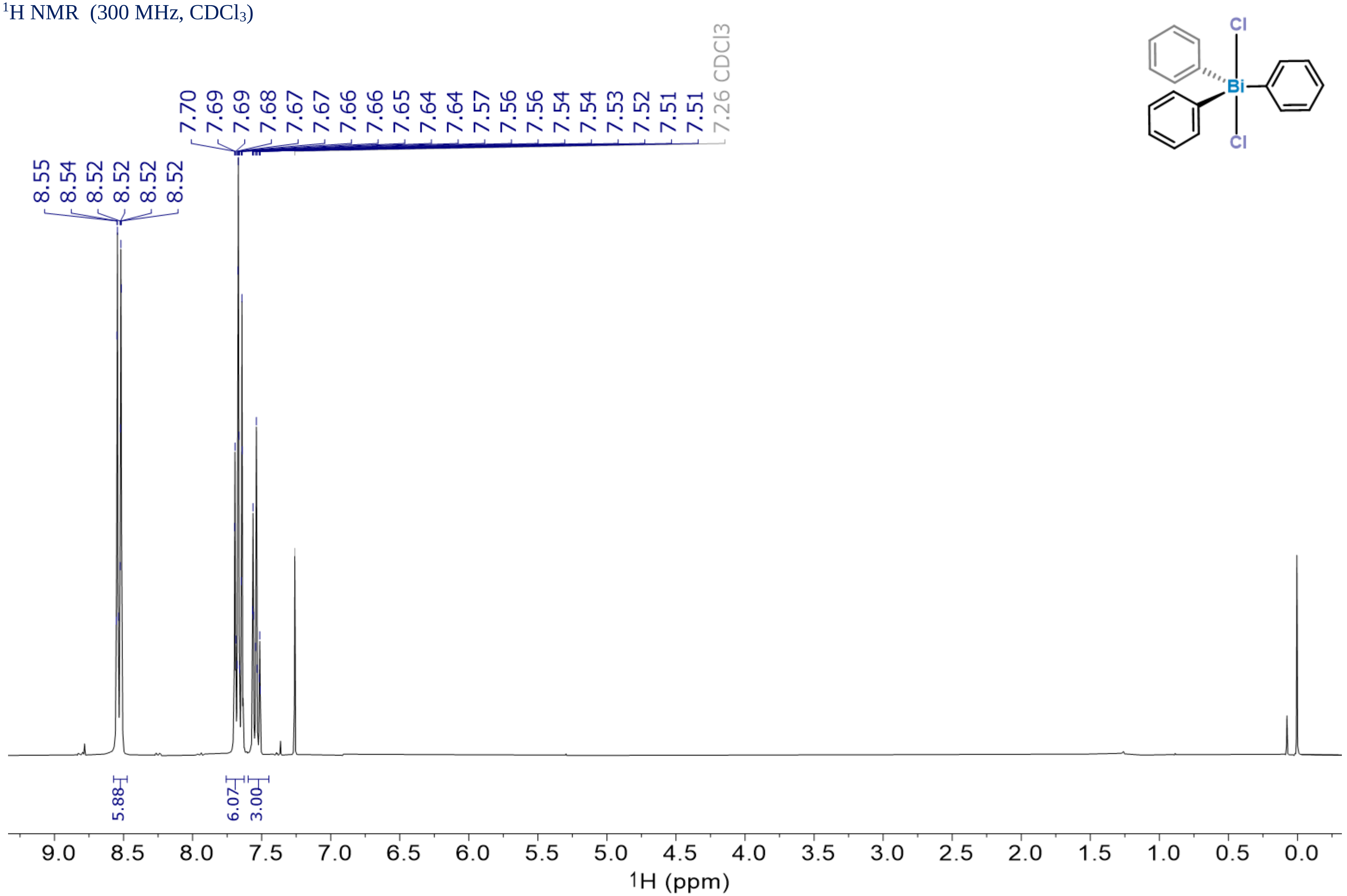

^{13}C NMR (75 MHz, $CDCl_3$)

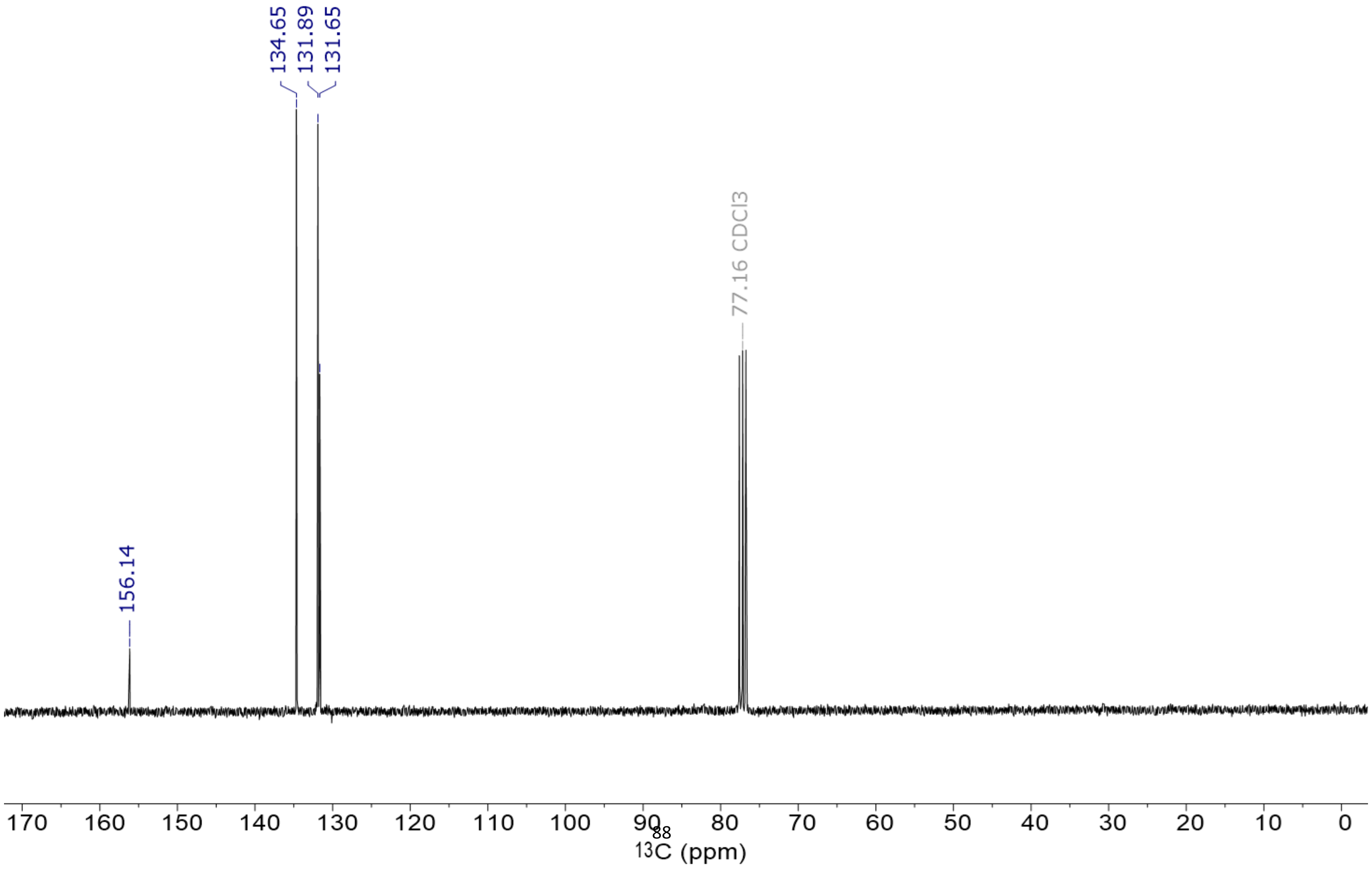

^1H NMR (500 MHz, $CDCl_3$)

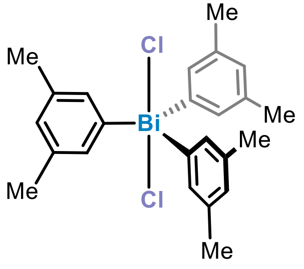

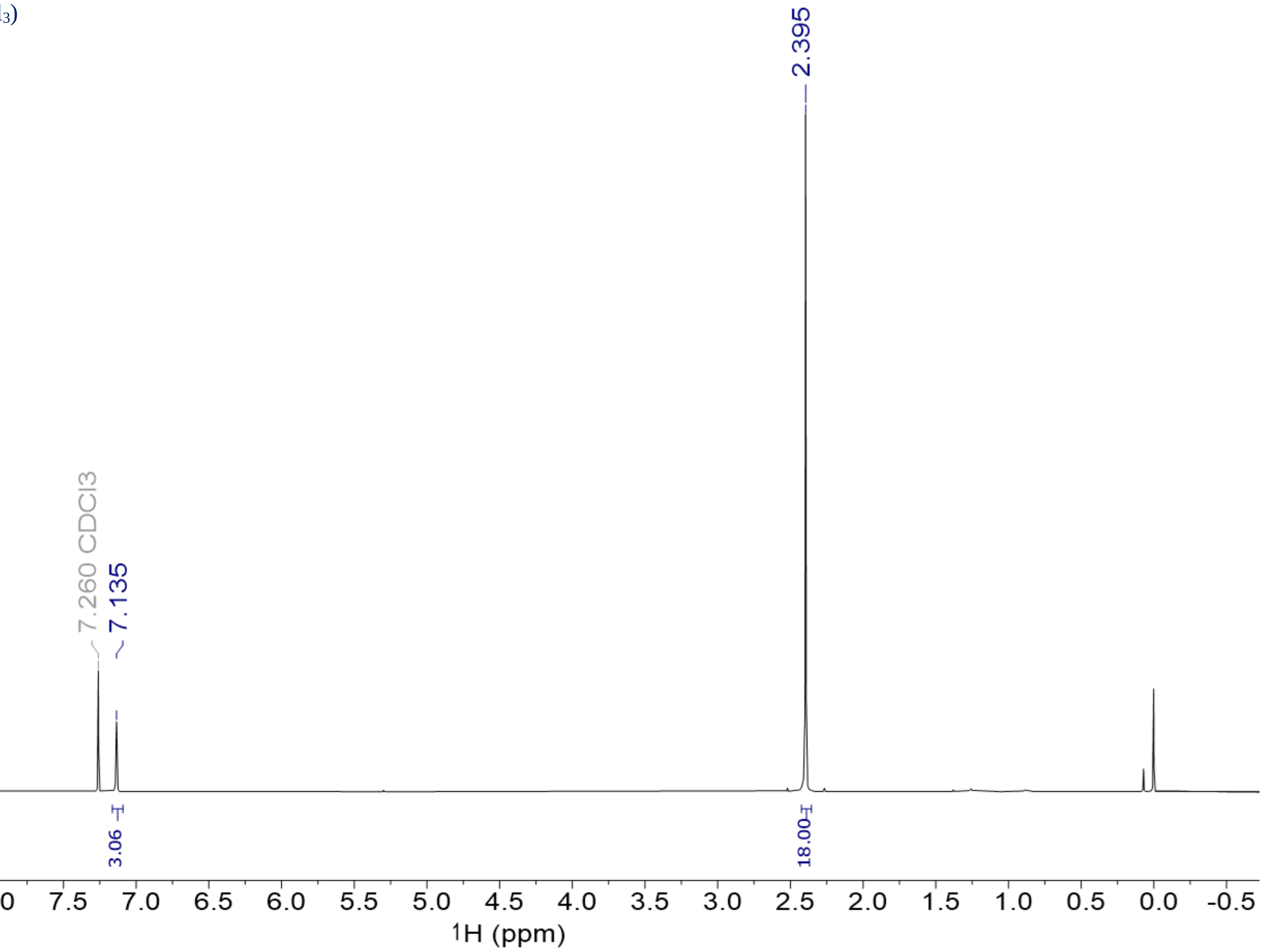

^{13}C NMR (101 MHz, $CDCl_3$)

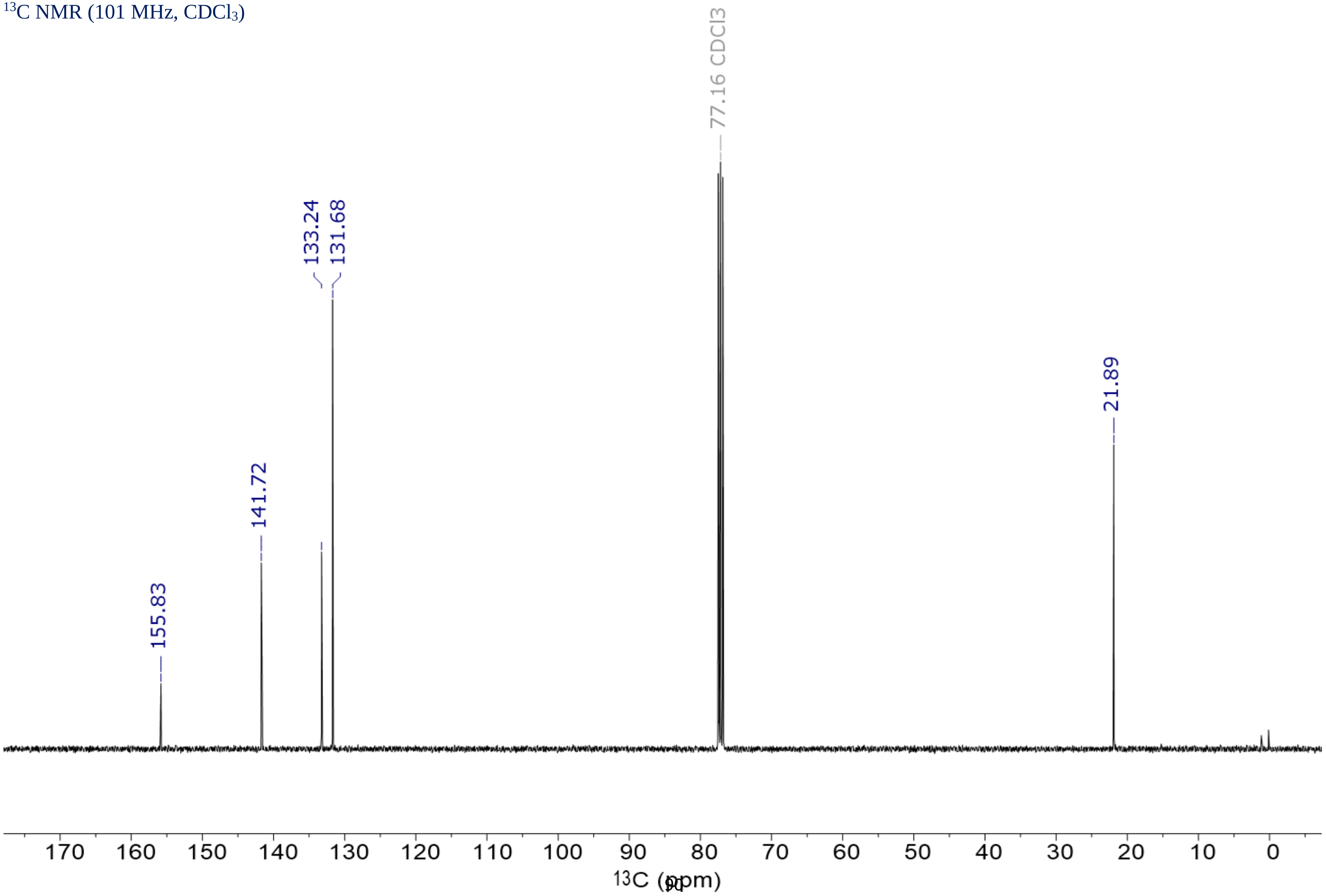

^1H NMR (300 MHz, $CDCl_3$)

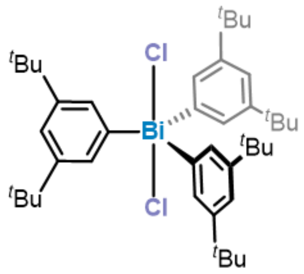

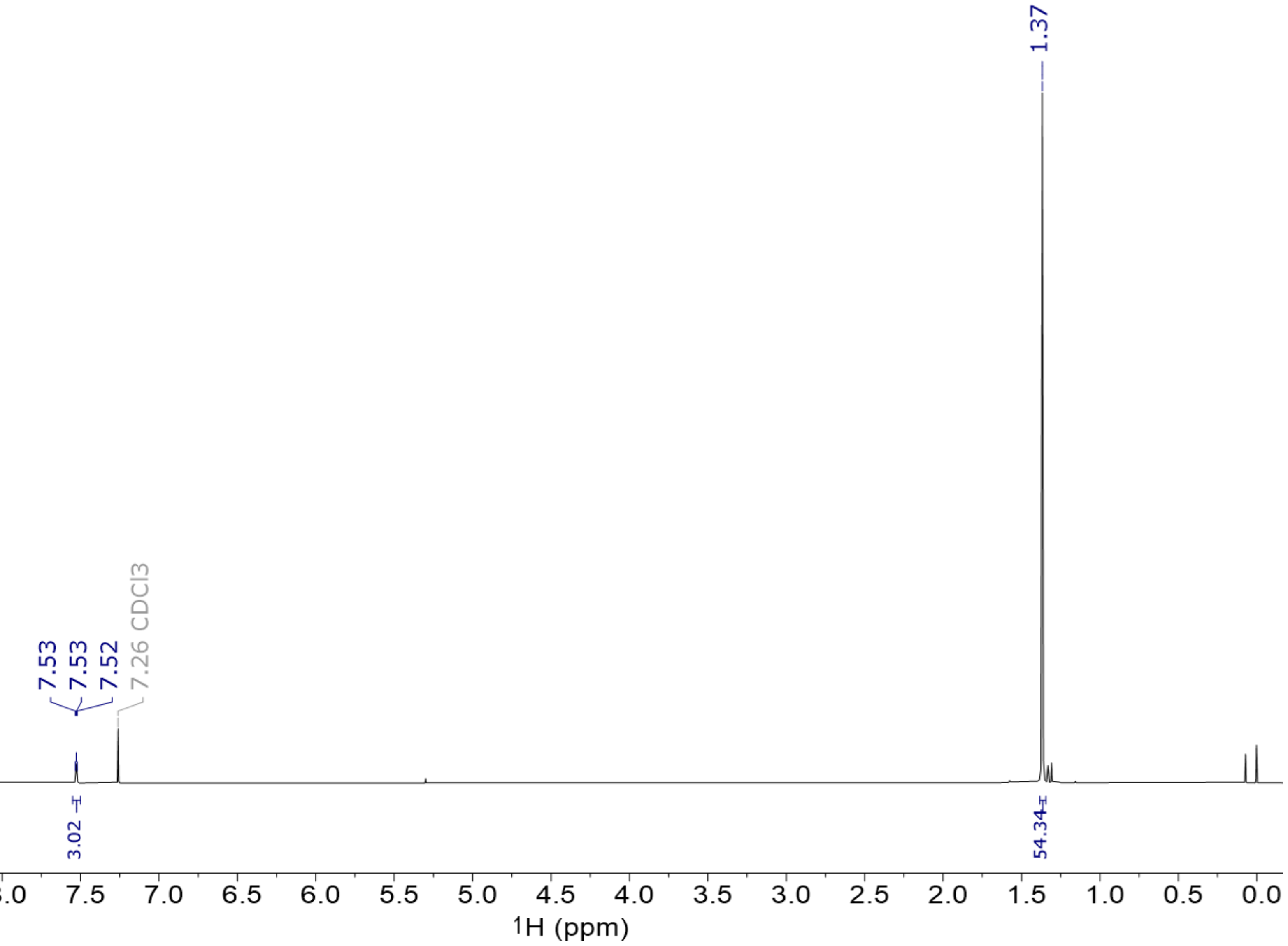

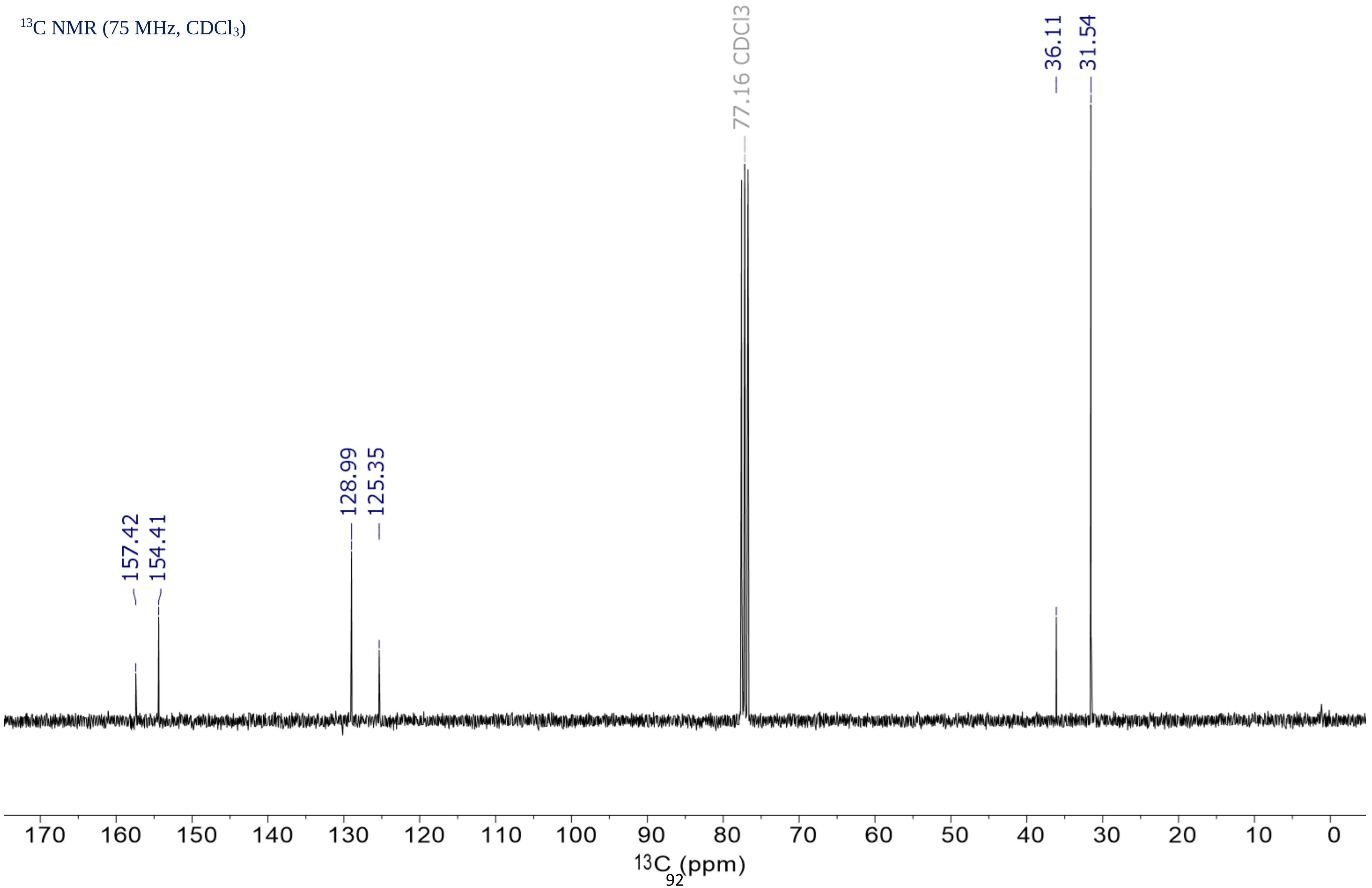
^{13}C NMR (75 MHz, $CDCl_3$)
157.42
154.41
128.99
125.35
77.16 CDCl3
36.11
31.54
170 160 150 140 130 120 110 100 90 80 70 60 50 40 30 20 10 0
13C (ppm)

◦^{1}H NMR (400 MHz, $CDCl_3$)

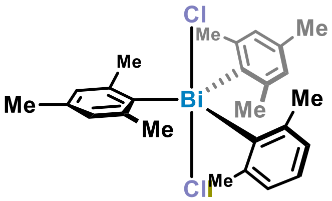

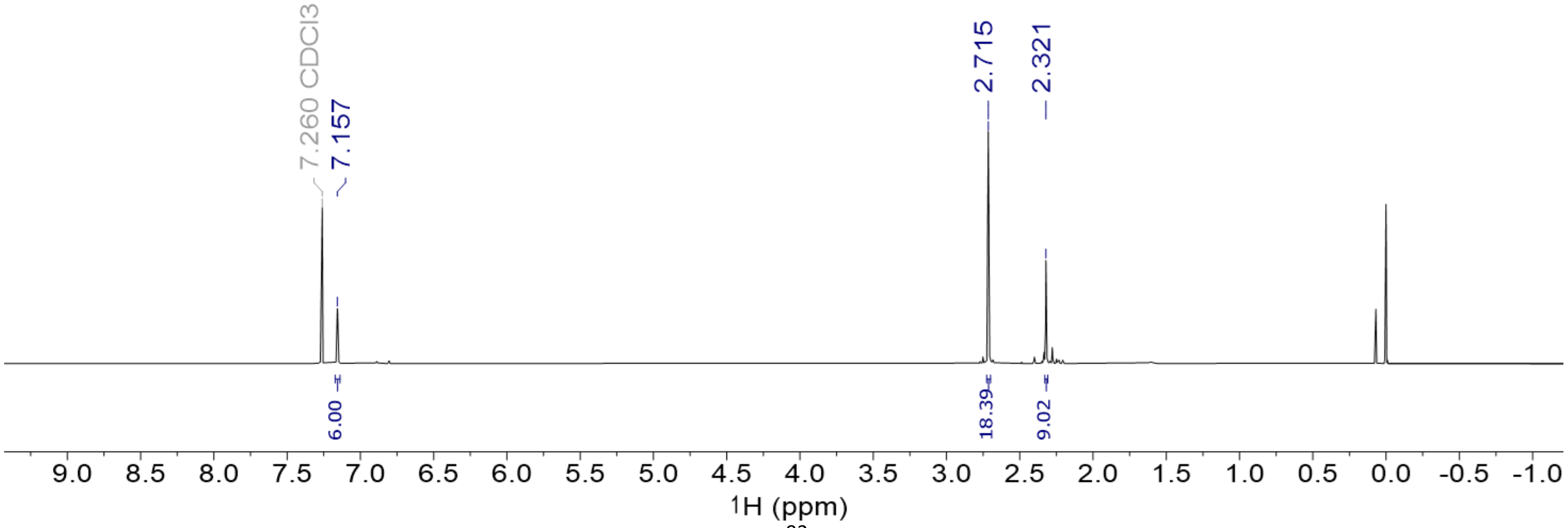

^{13}C NMR (75 MHz, $CDCl_3$)

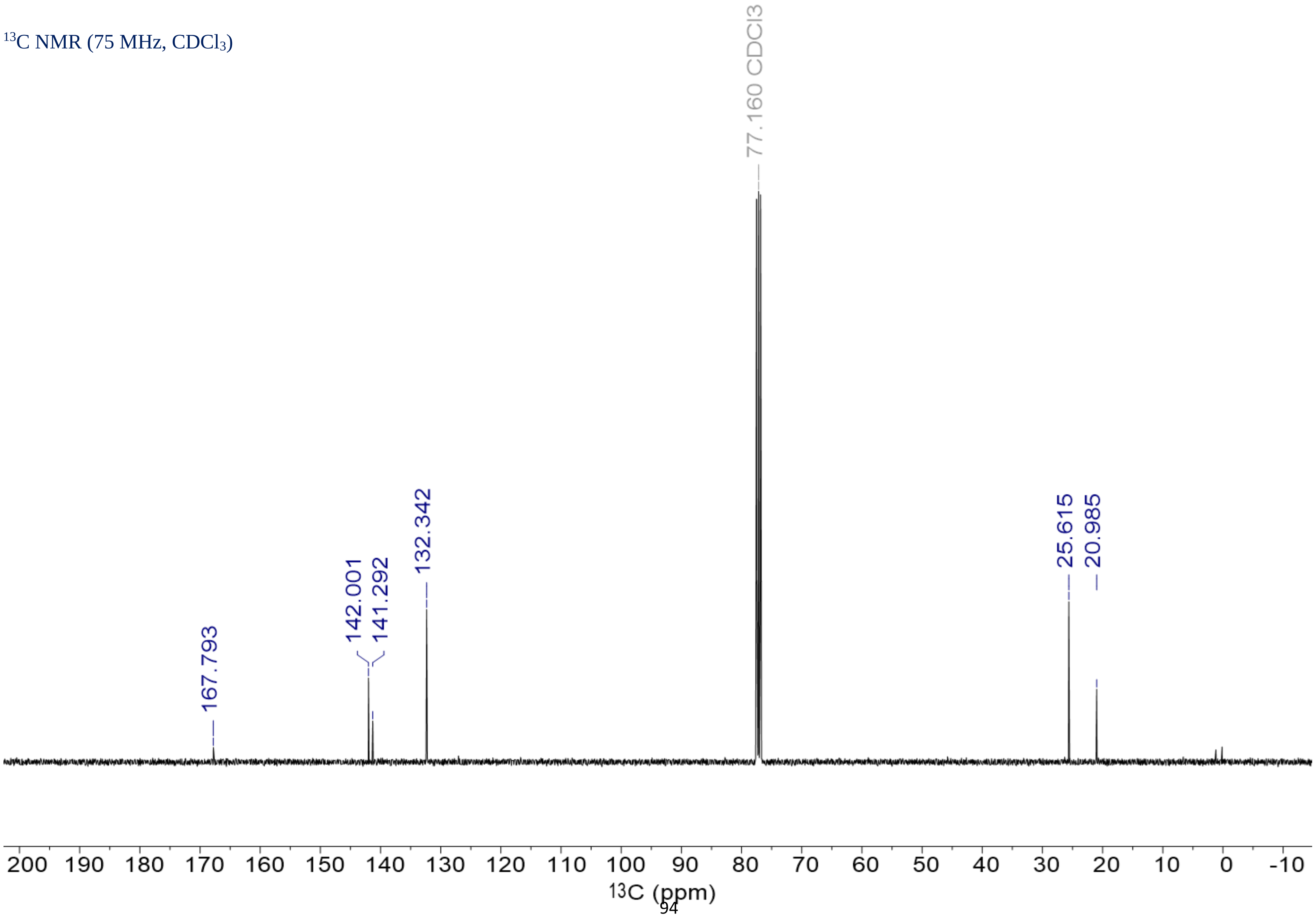

^{1}H NMR (300 MHz, $CDCl_3$)

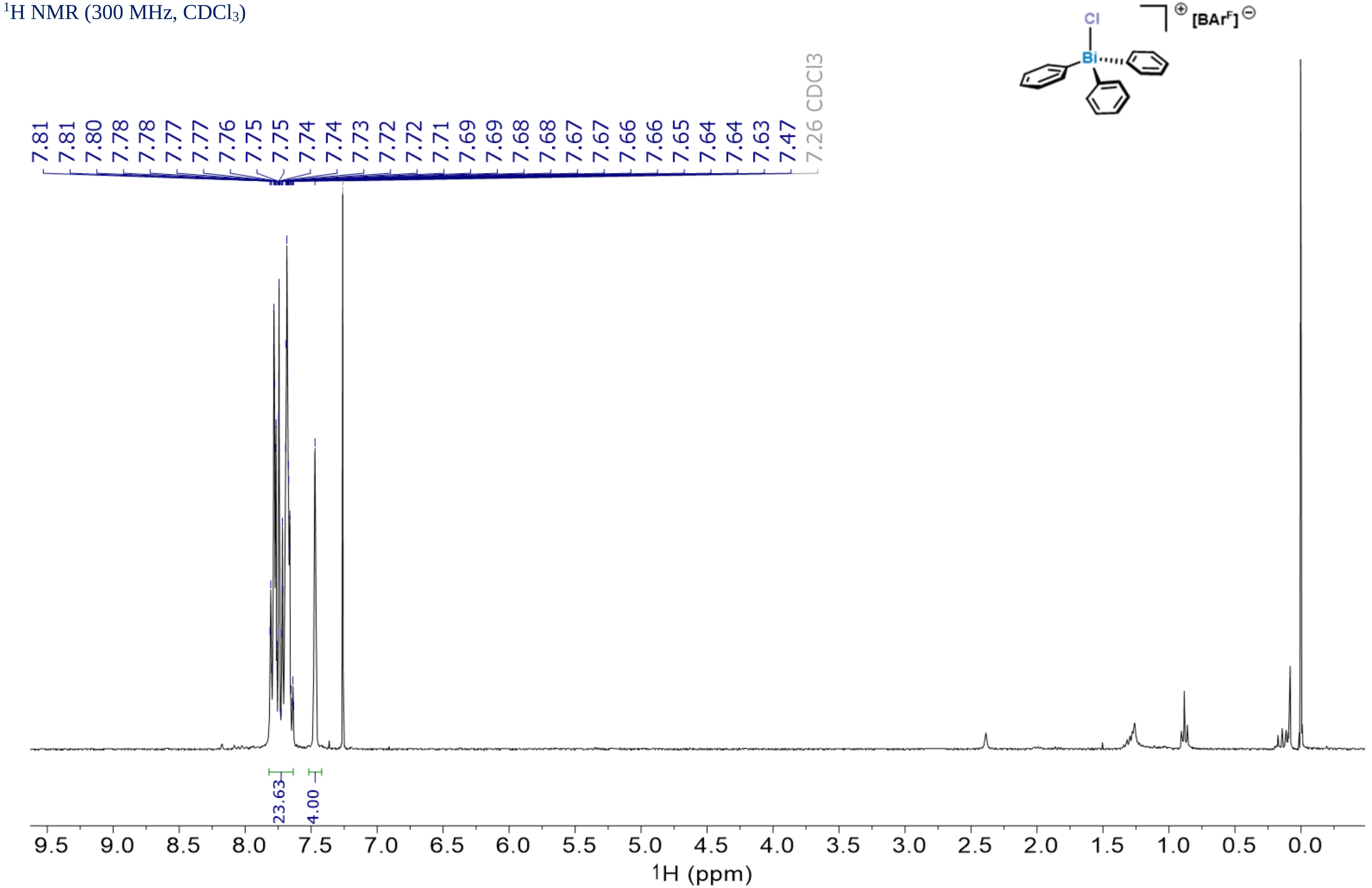

^{13}C NMR (101 MHz, $CDCl_3$)

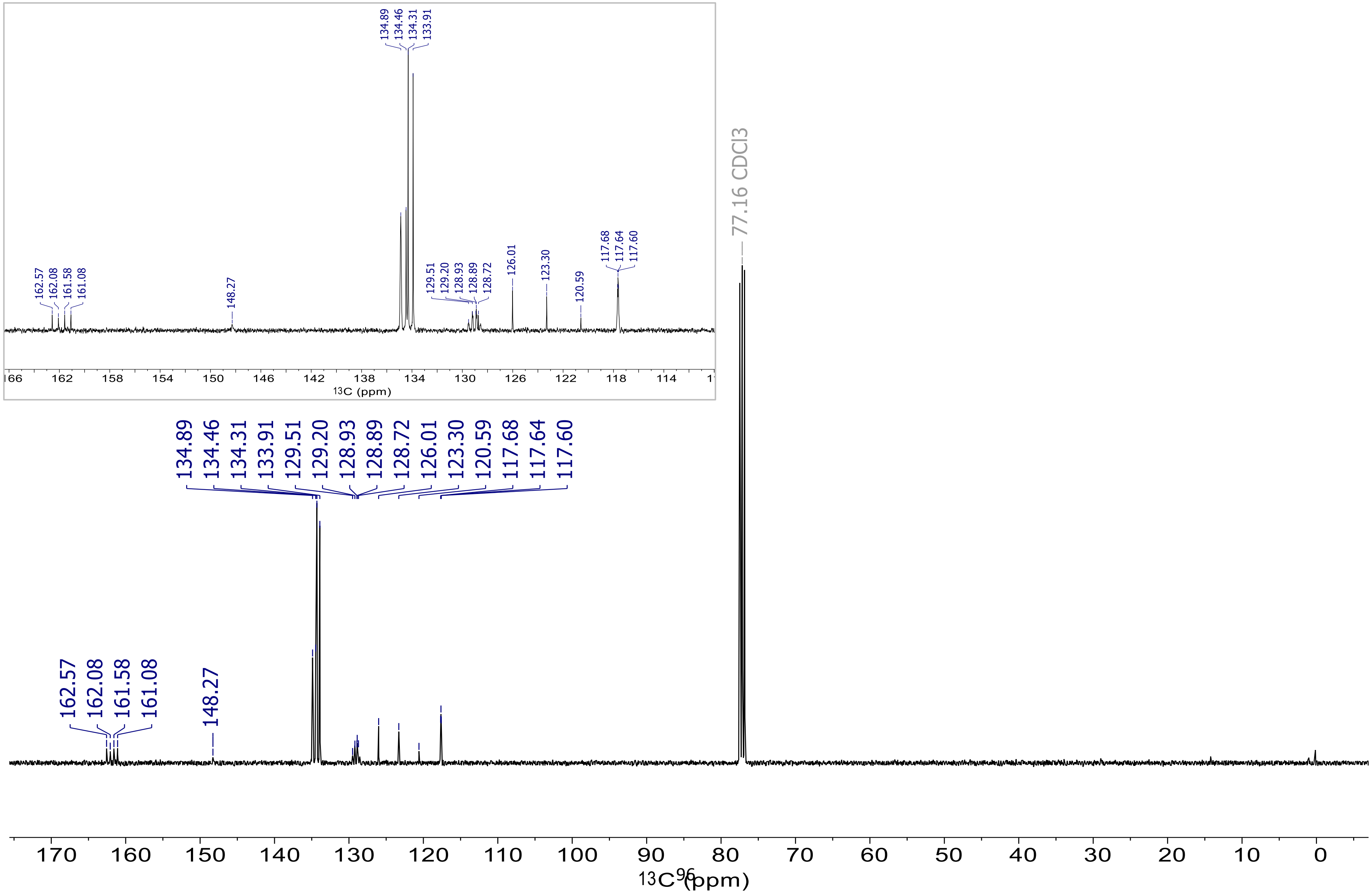

^{19}F NMR (282 MHz, $CDCl_3$)

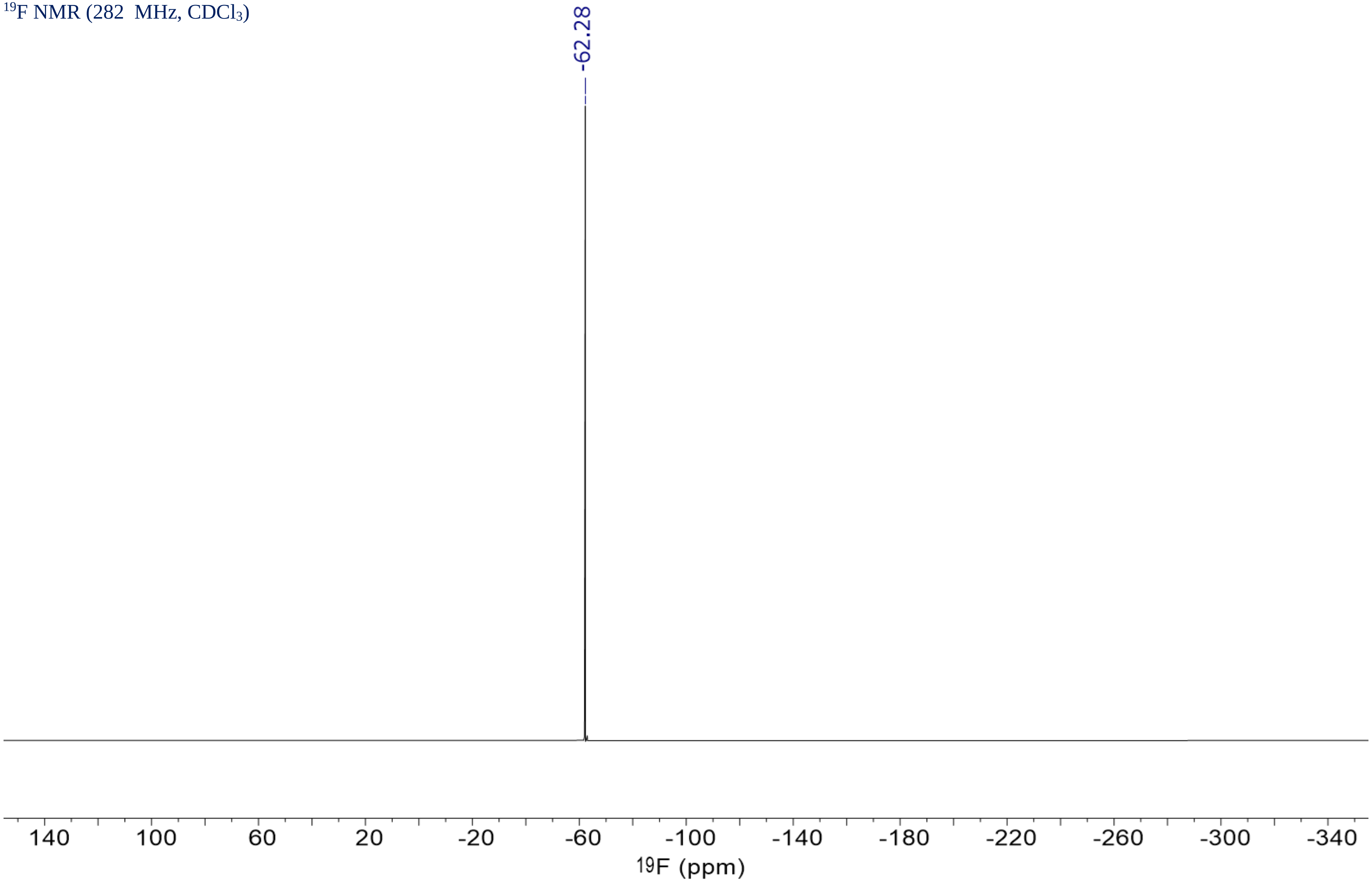

^{1}H NMR (600 MHz, $CDCl_3$)

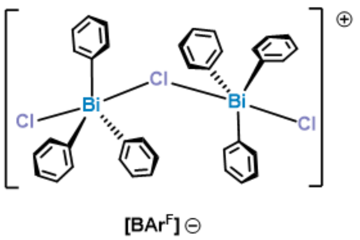

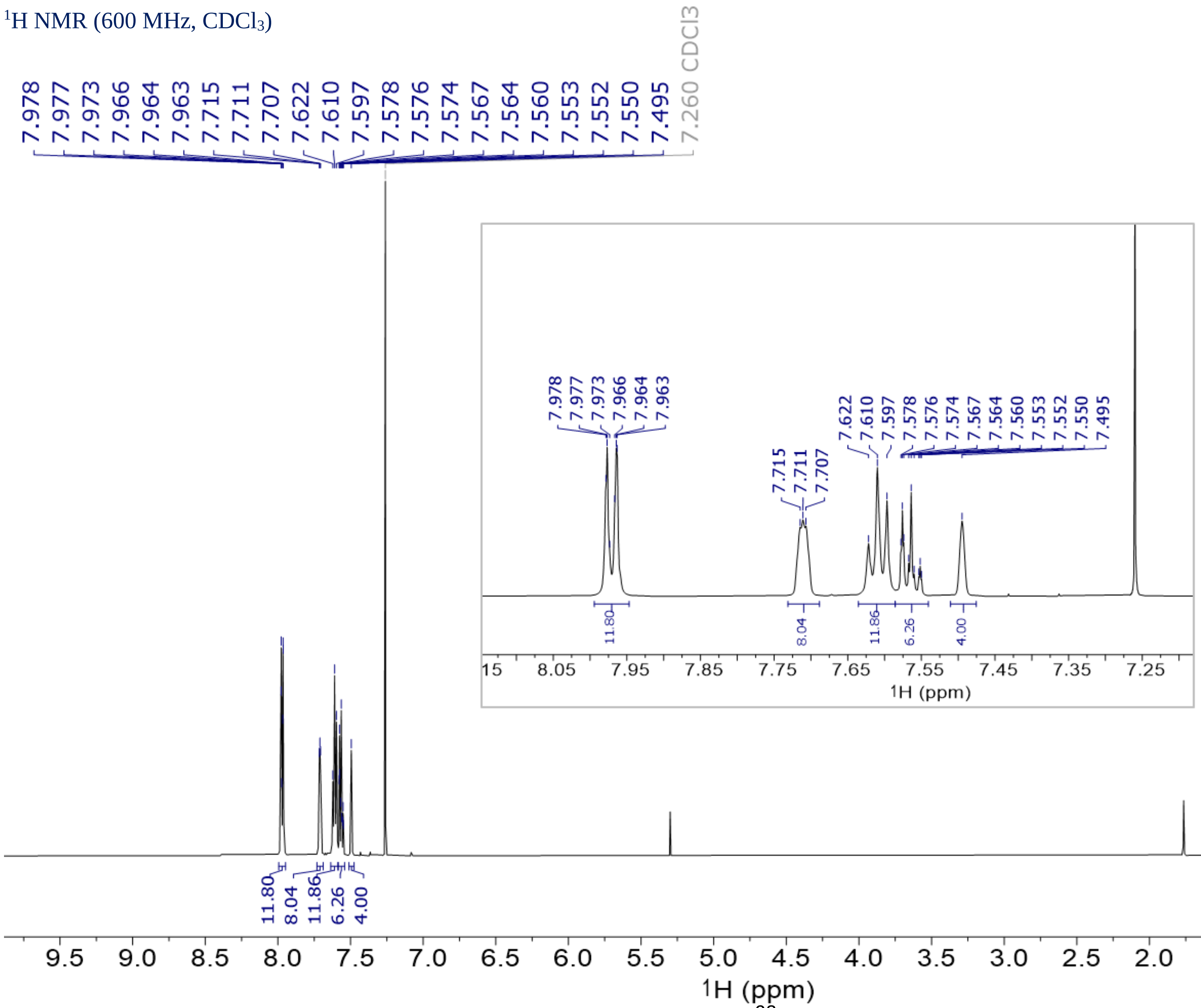

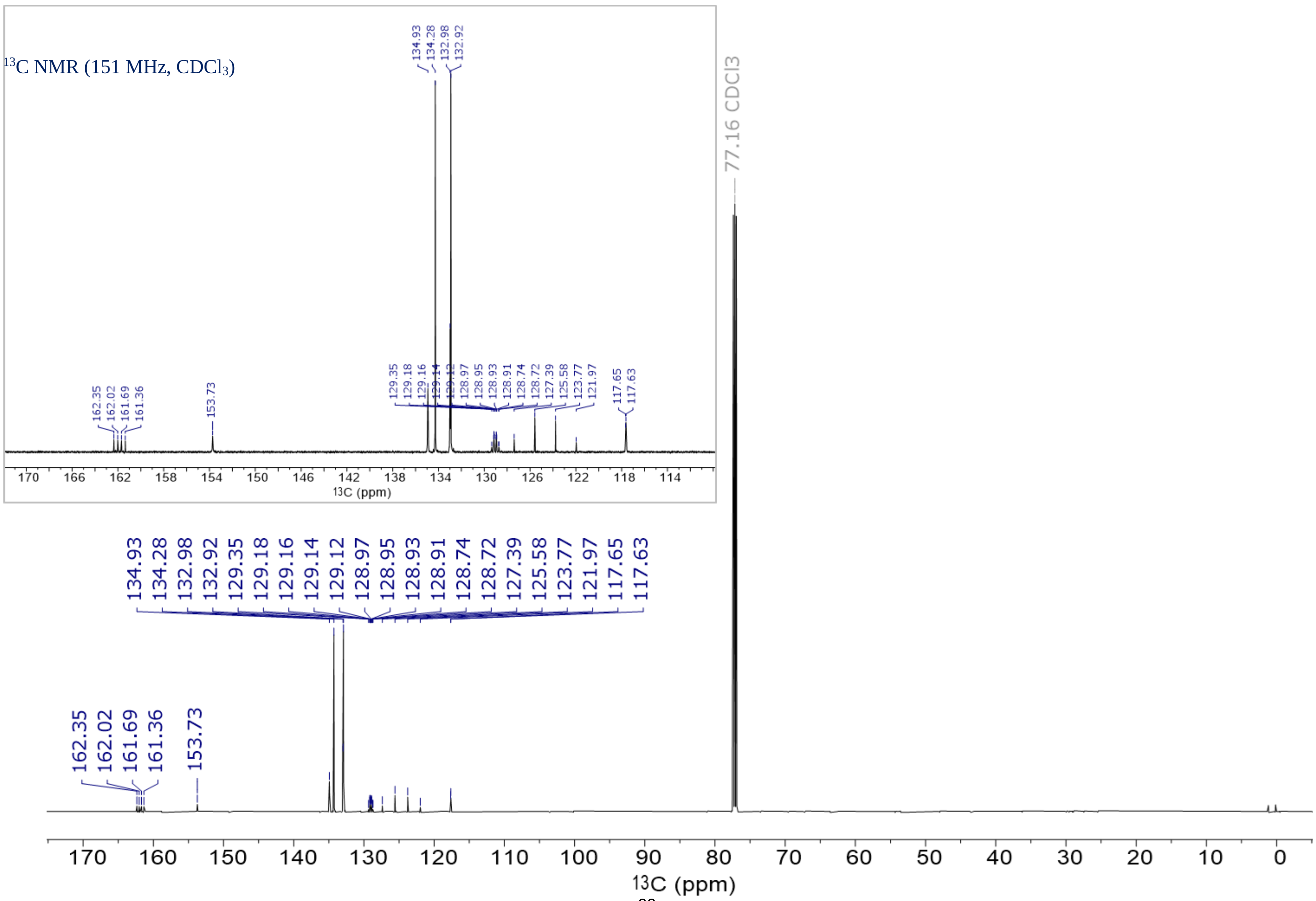

13C NMR (151 MHz, CDCl3)
162.35
162.02
161.69
161.36
153.73
134.93
134.28
132.98
132.92
129.35
129.18
129.16
129.14
129.12
128.97
128.95
128.93
128.91
128.74
128.72
127.39
125.58
123.77
121.97
117.65
117.63
77.16 CDCl3
13C (ppm)

^{19}F NMR (565 MHz, $CDCl_3$)

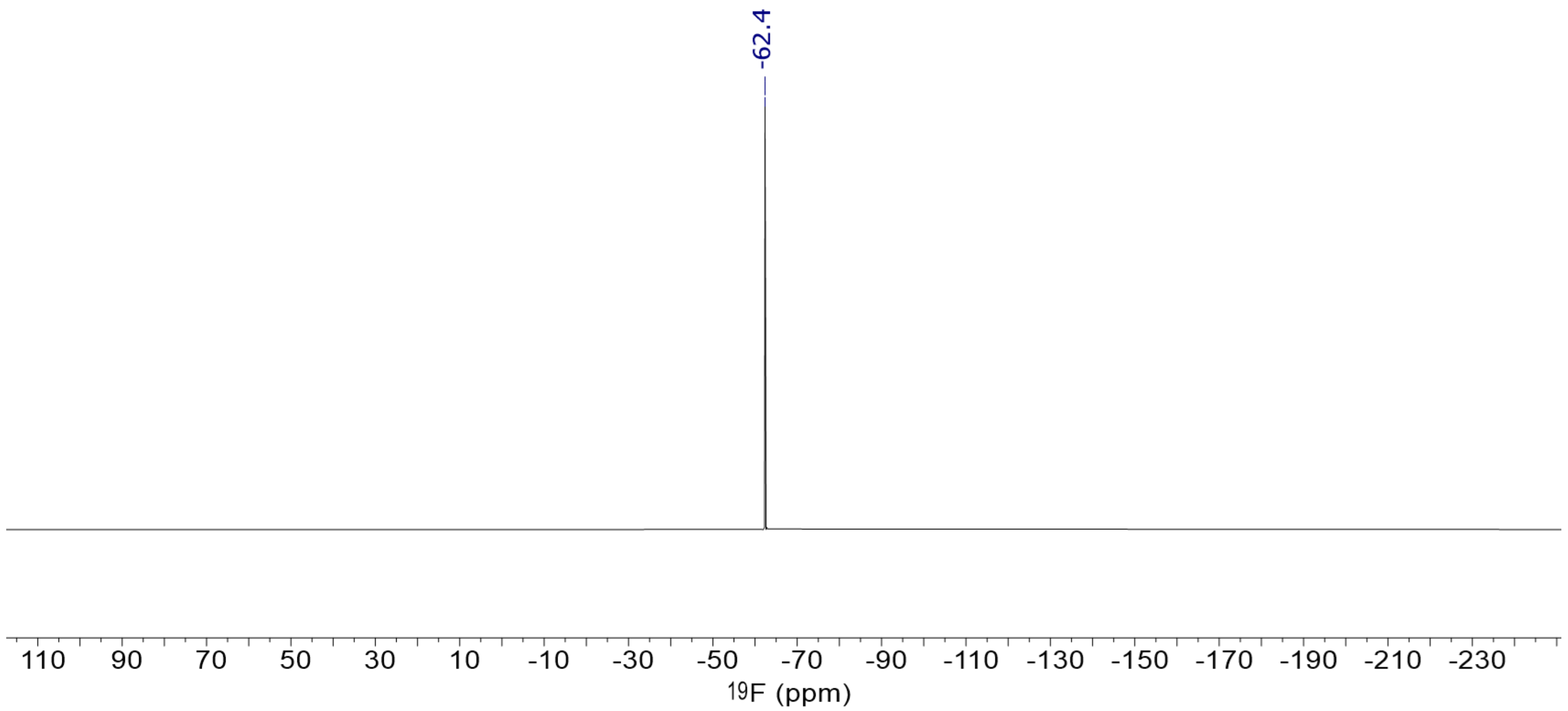

^{1}H NMR (600 MHz, CDCl$_3$)

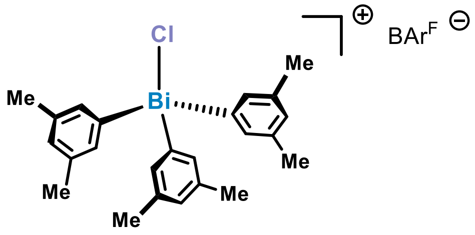

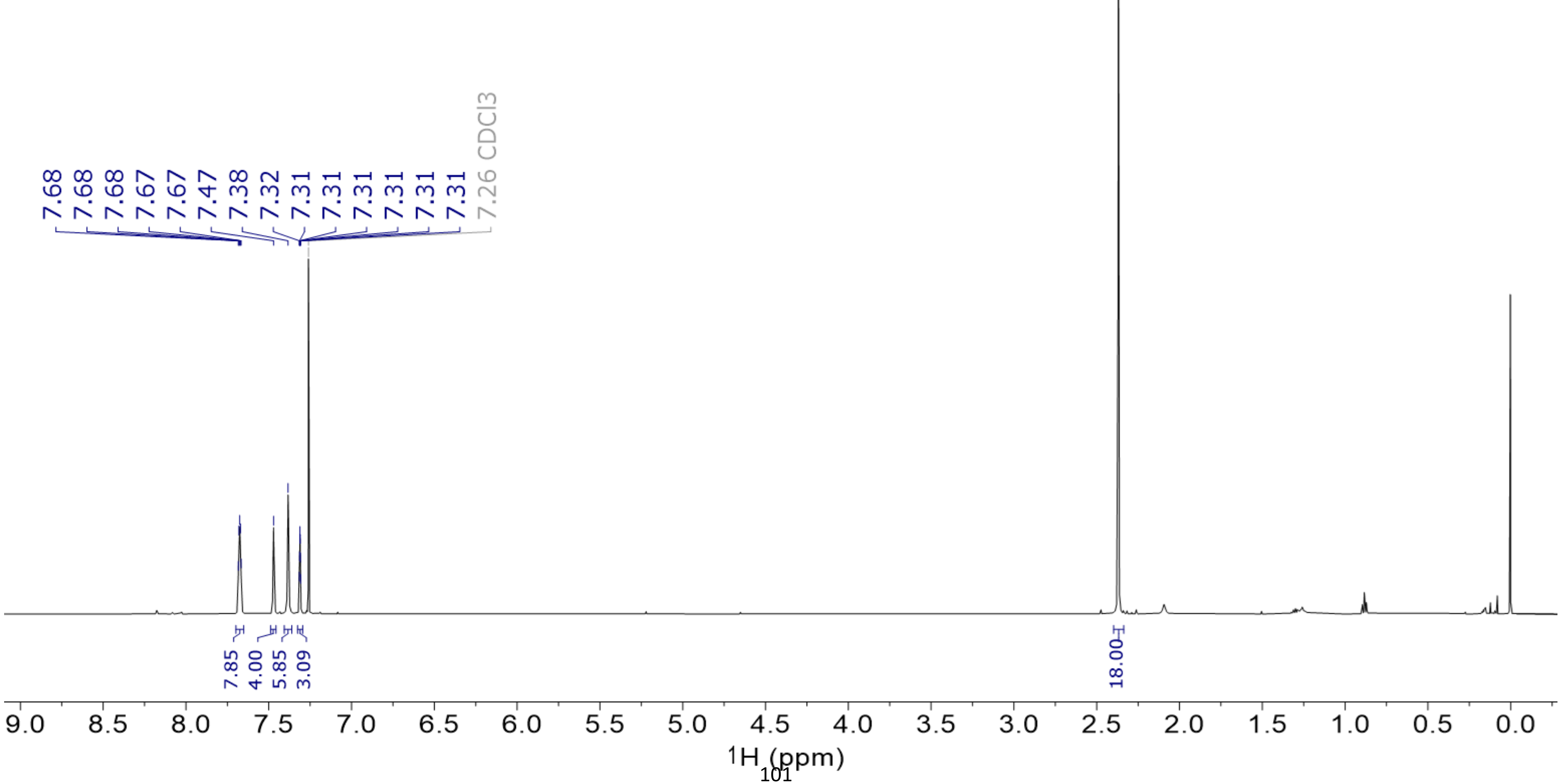

^{13}C NMR (151 MHz, $CDCl_3$)

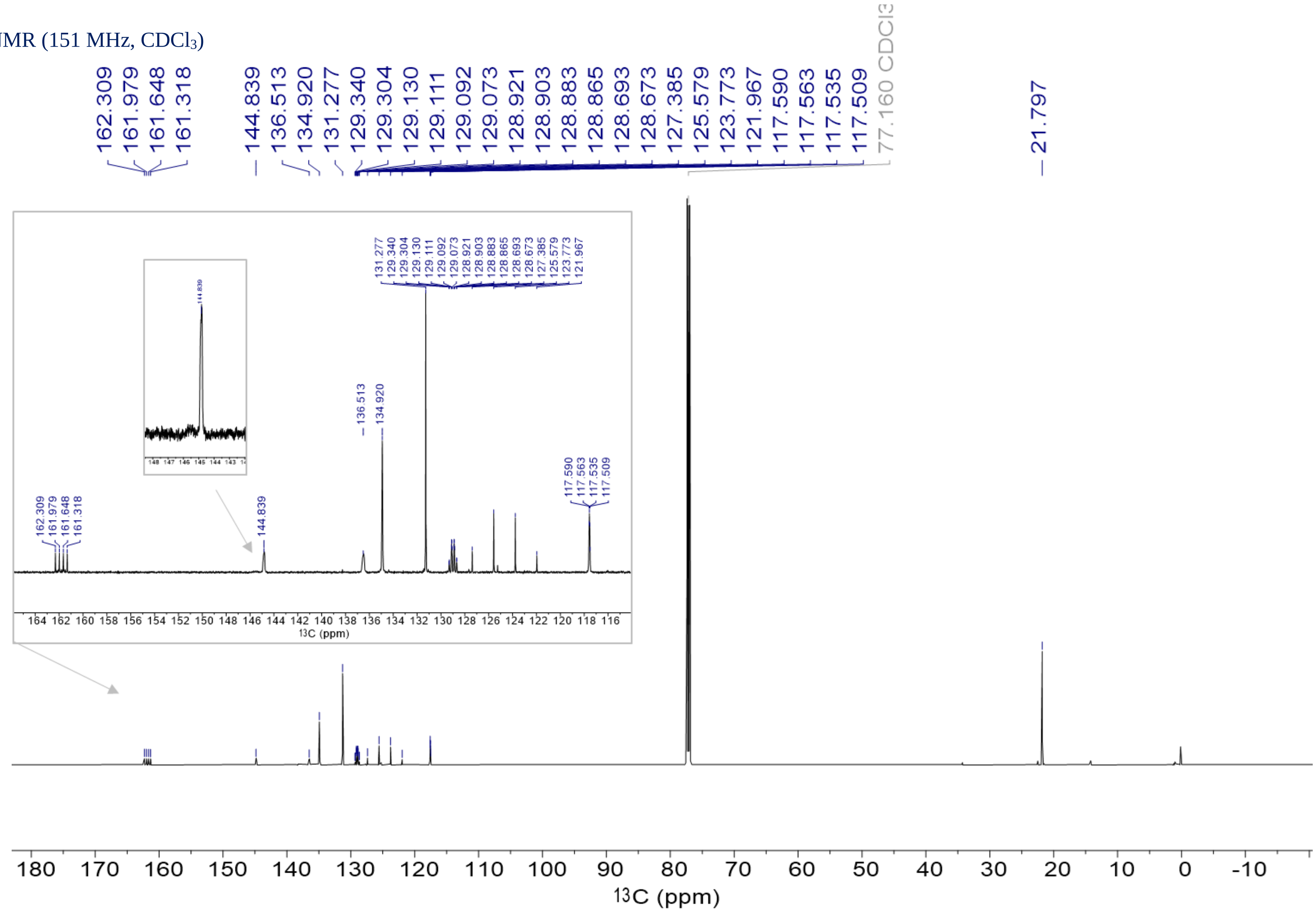

^{19}F NMR (564 MHz, $CDCl_3$)

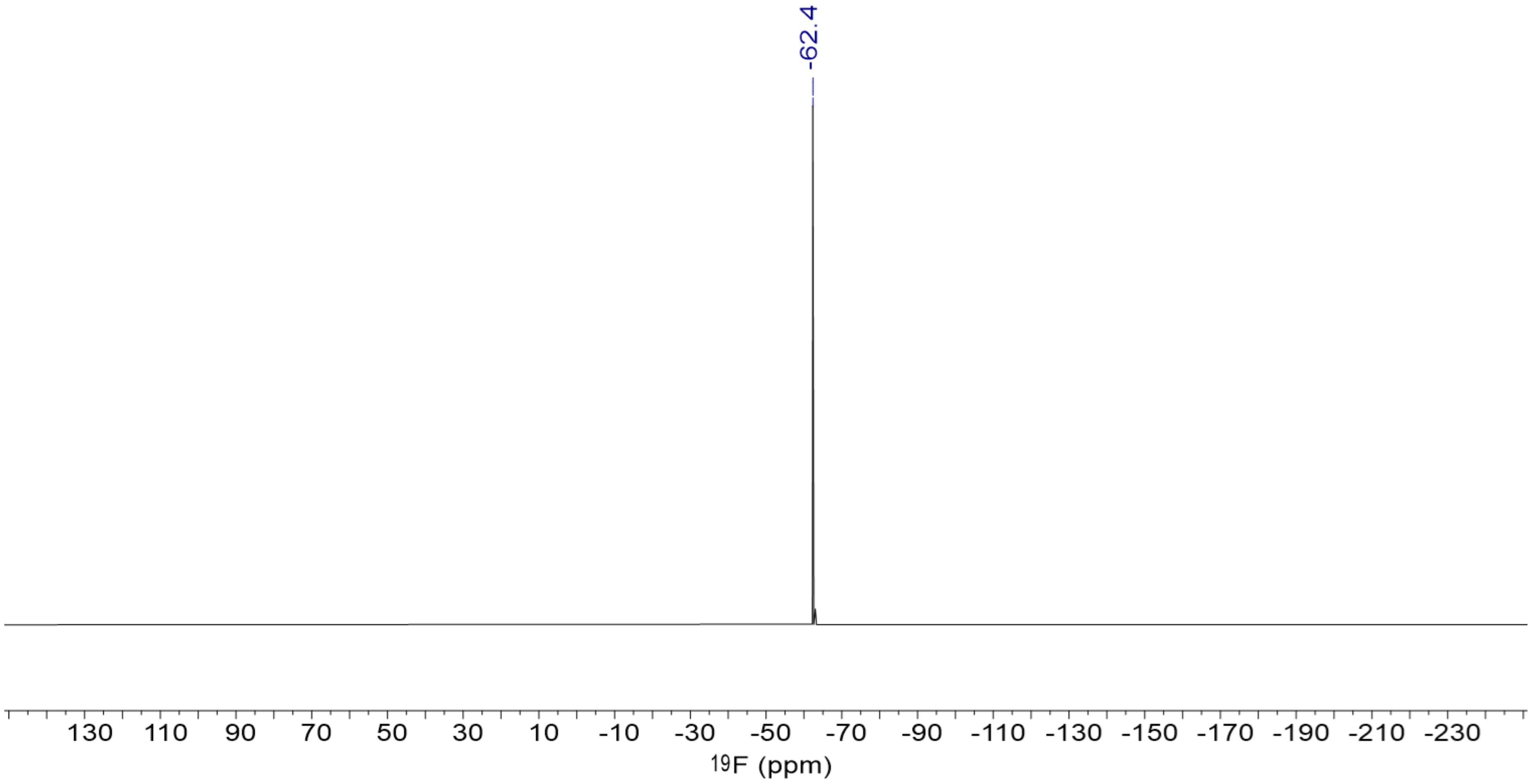

^{1}H NMR (400 MHz, $CDCl_3$)

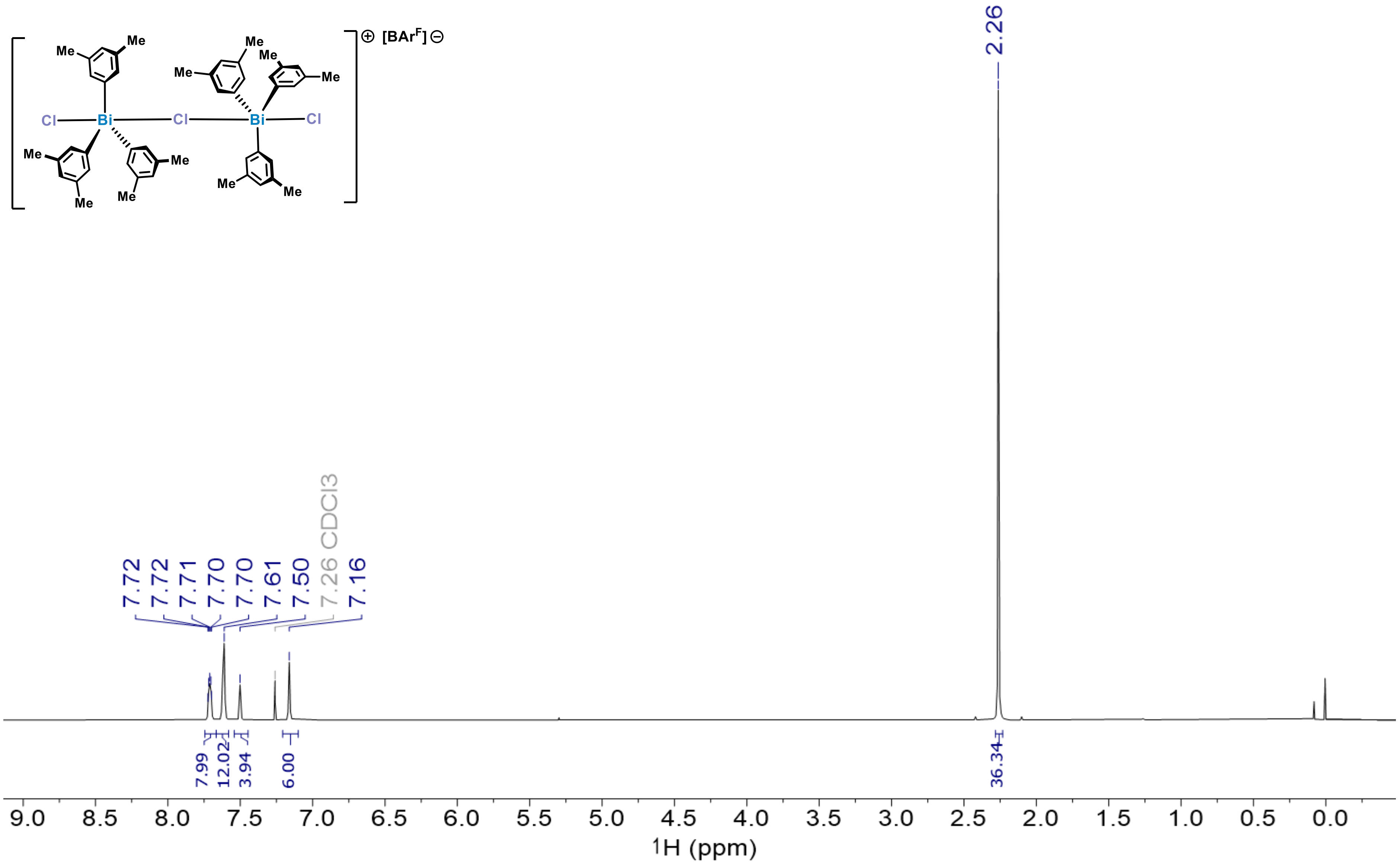

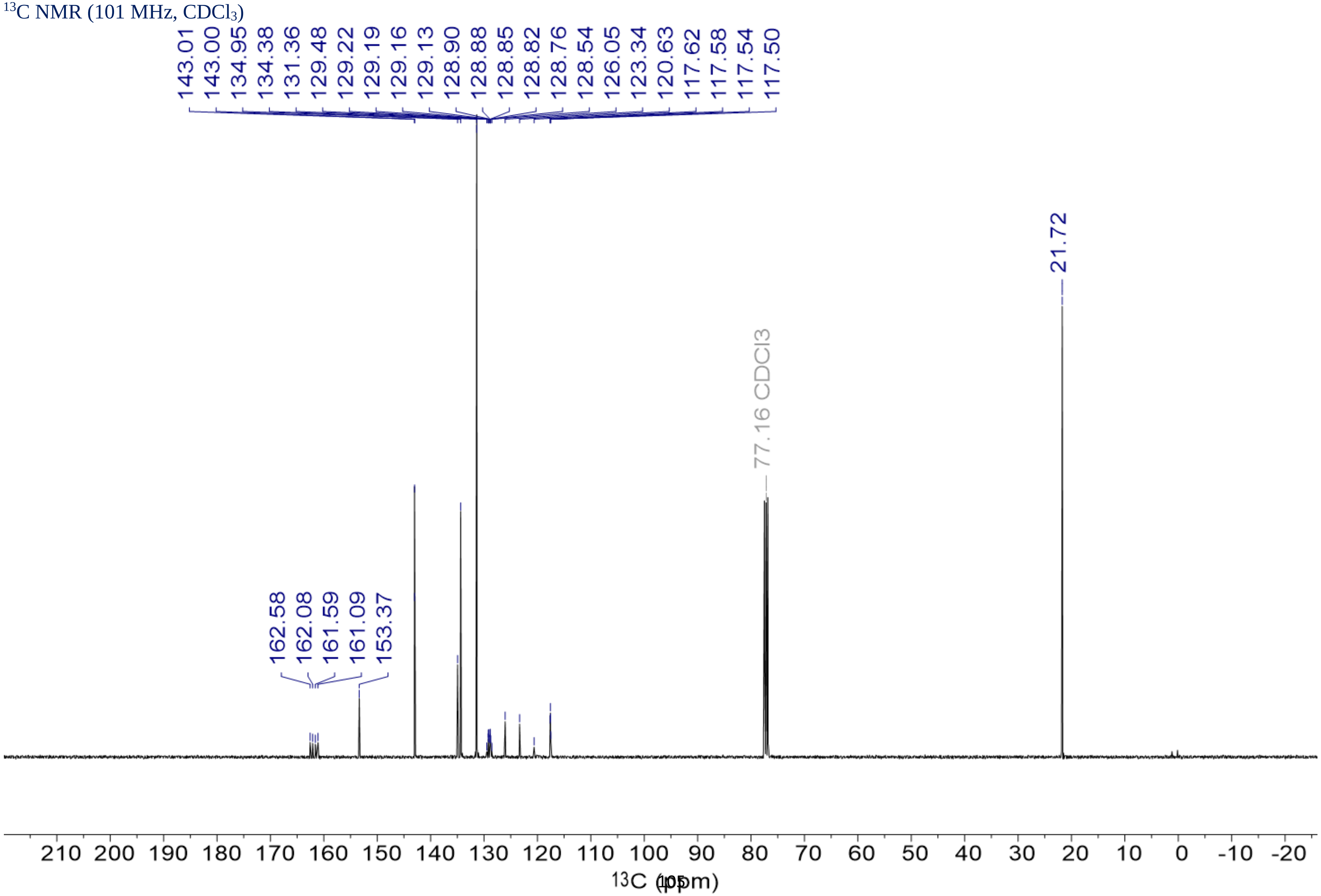

^{13}C NMR (101 MHz, $CDCl_3$)
143.01
143.00
134.95
134.38
131.36
129.48
129.22
129.19
129.16
129.13
128.90
128.88
128.85
128.82
128.76
128.54
126.05
123.34
120.63
117.62
117.58
117.54
117.50
21.72
77.16 CDCl3
162.58
162.08
161.59
161.09
153.37
210 200 190 180 170 160 150 140 130 120 110 100 90 80 70 60 50 40 30 20 10 0 -10 -20
^{13}C (ppm)

^{19}F NMR (282 MHz, $CDCl_3$)

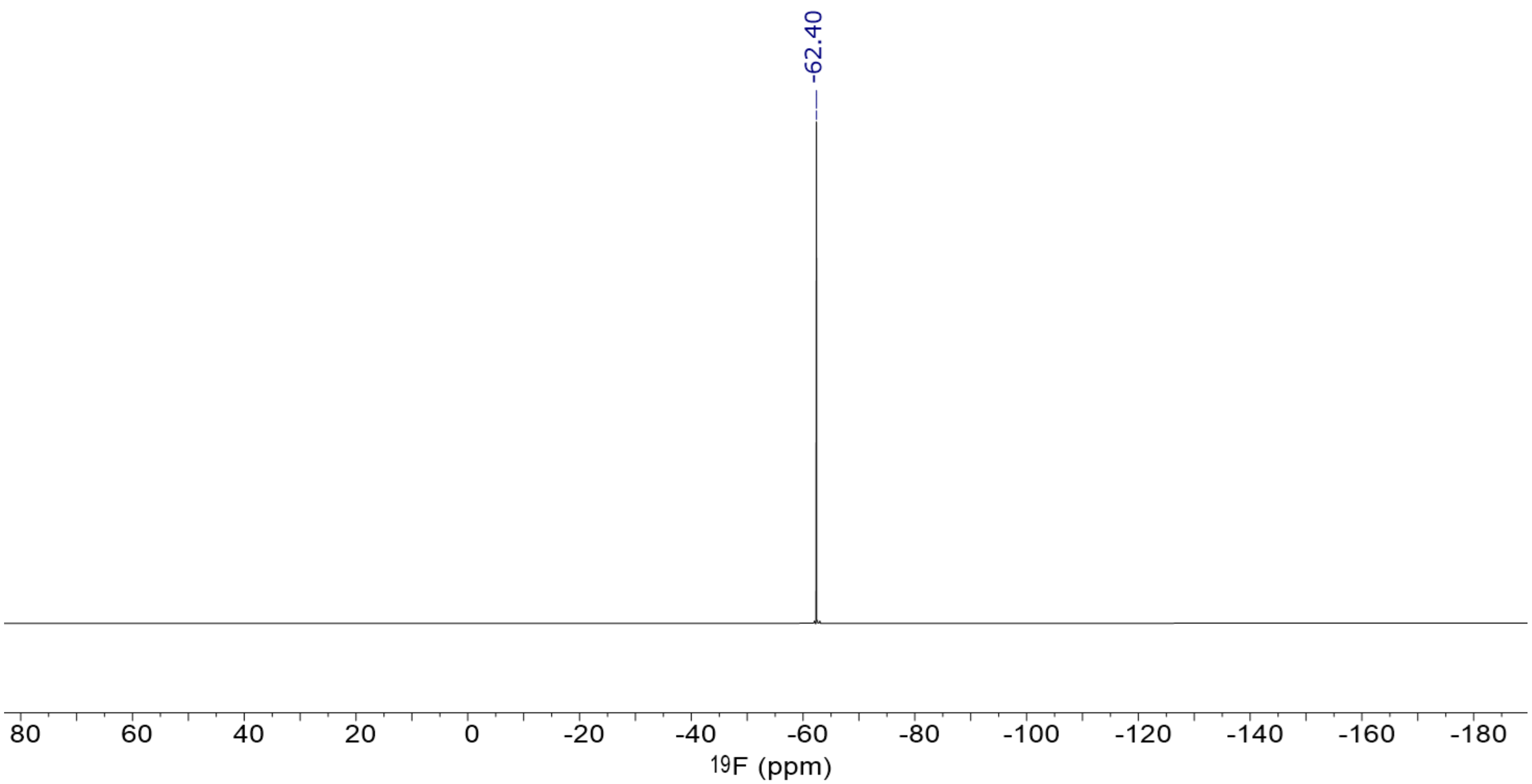

^1H NMR (300 MHz, $CDCl_3$) 1.0 equiv.

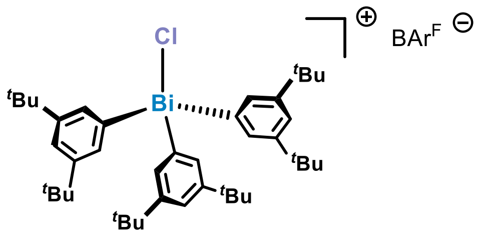

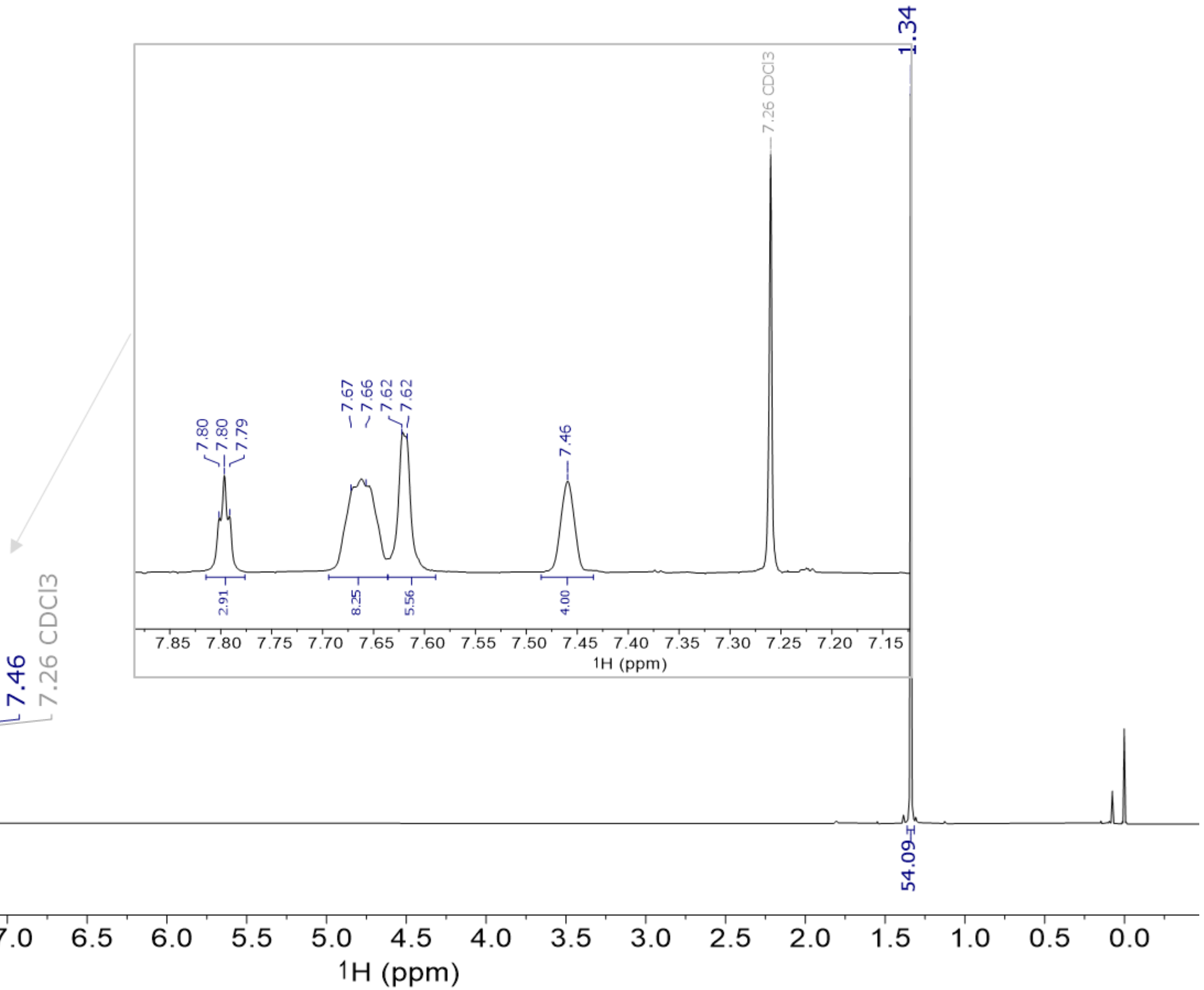

^{13}C NMR (151 MHz, $CDCl_3$)

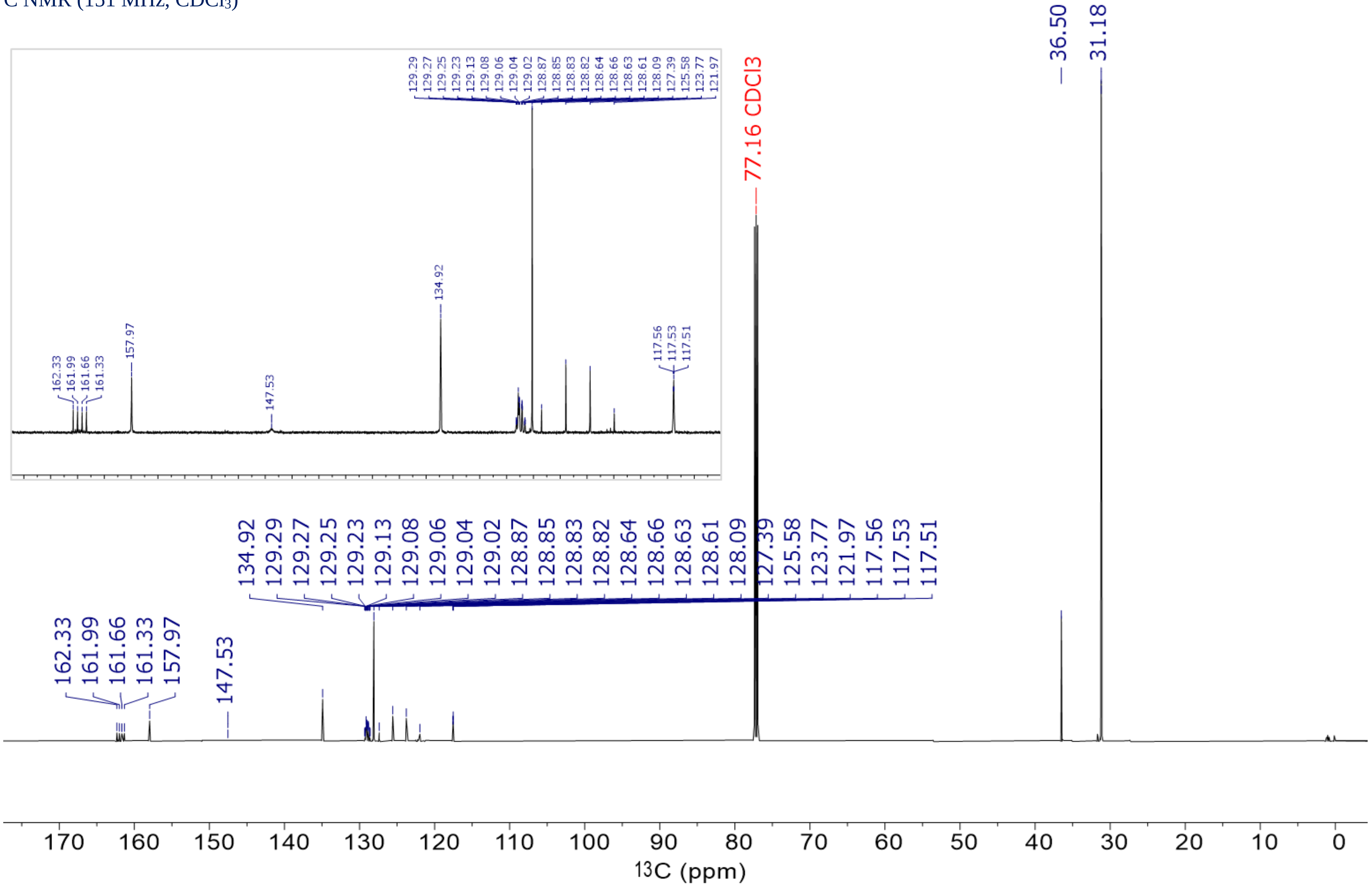

^{19}F NMR (564 MHz, $CDCl_3$)

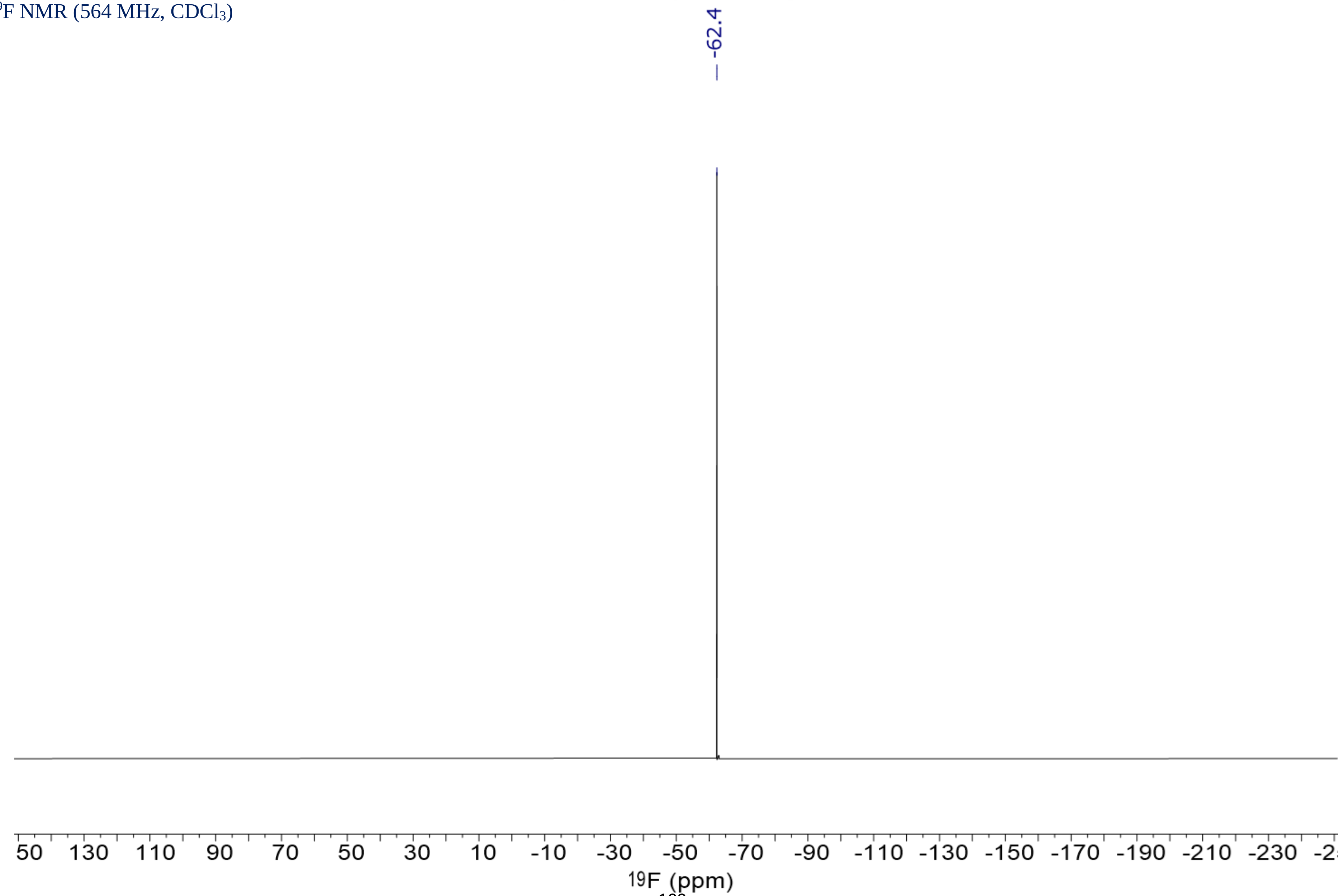

¹H NMR (600 MHz, $CDCl_3$) 0.5 equiv.

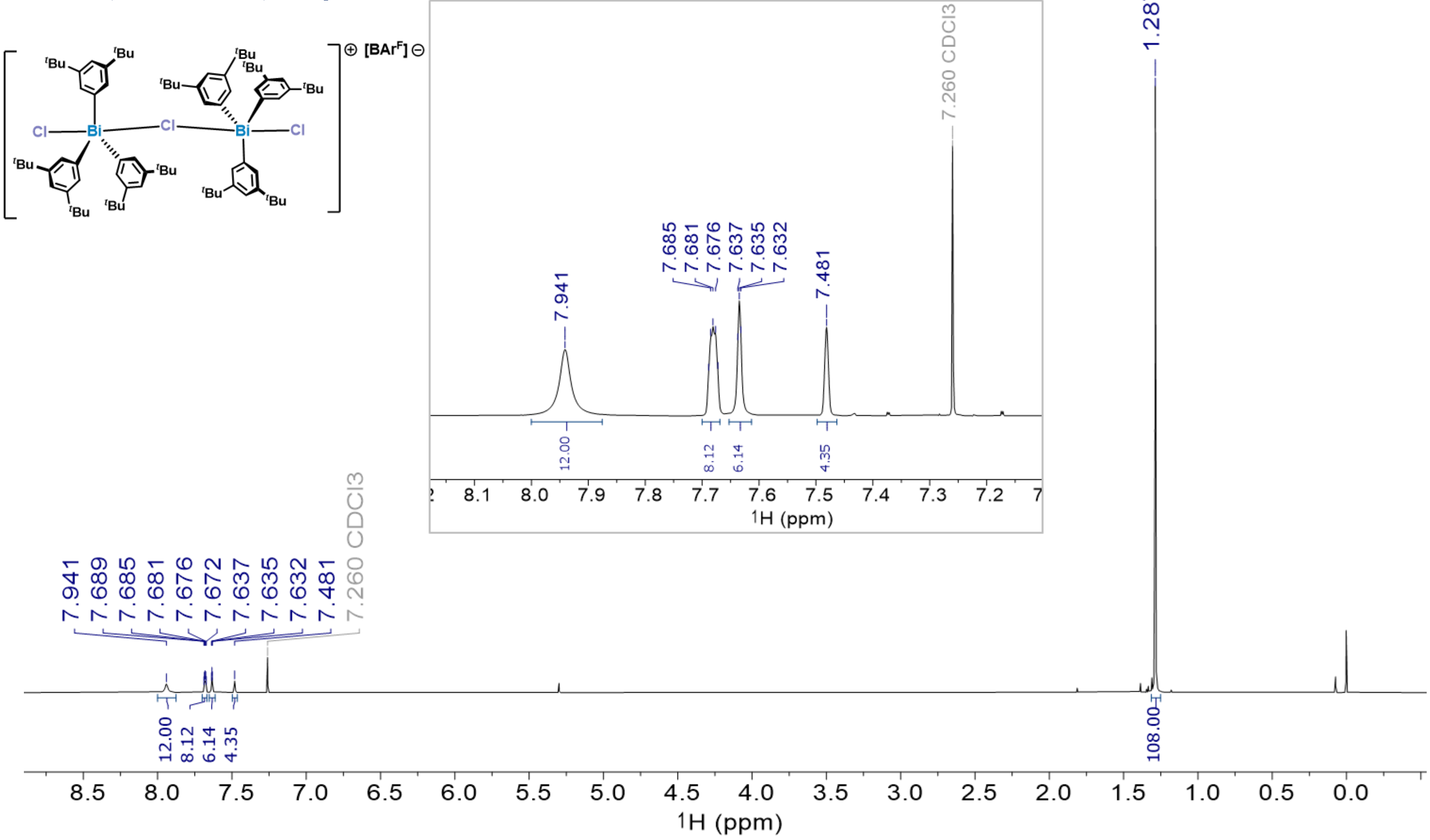

^{13}C NMR (151 MHz, $CDCl_3$)

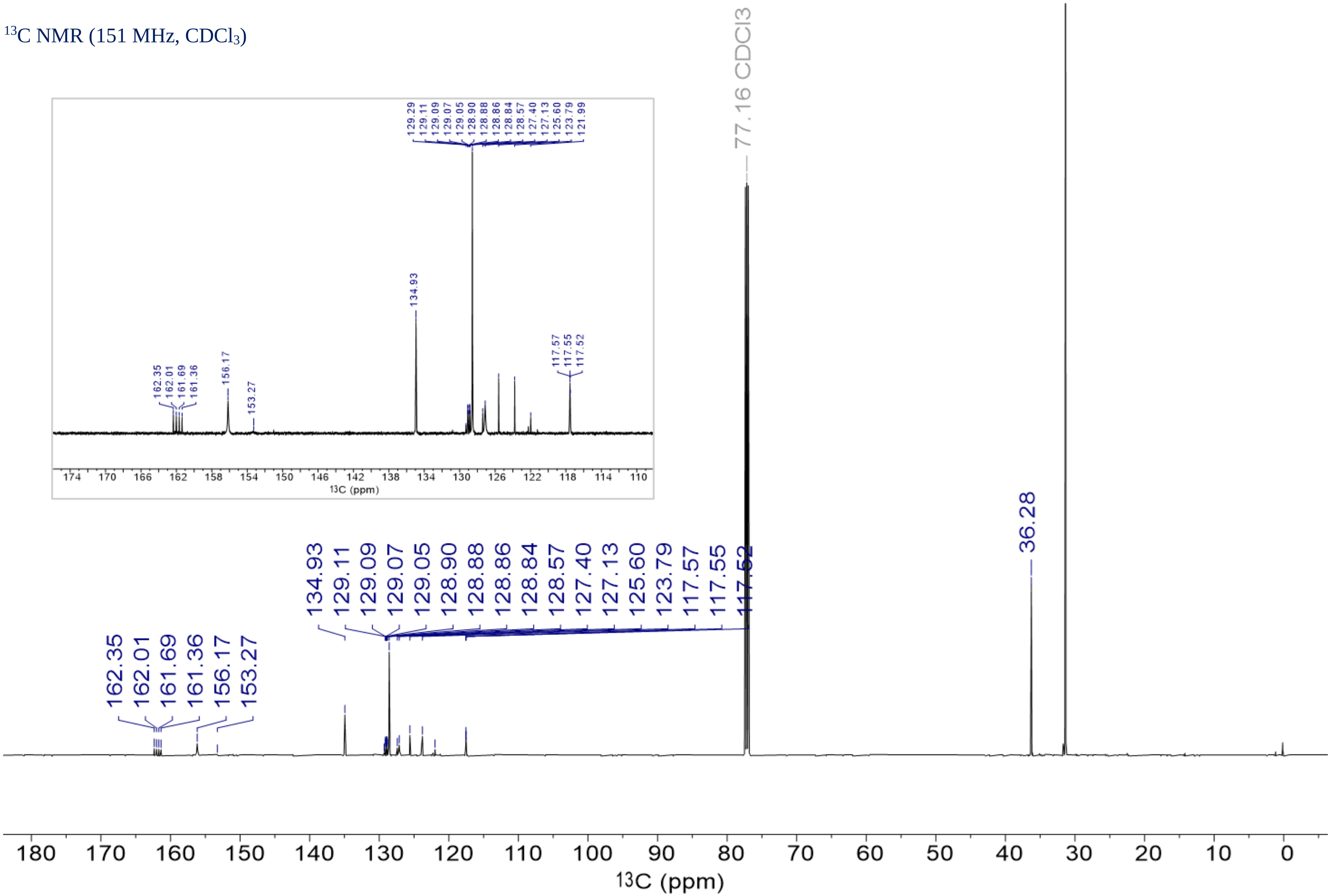

^{19}F NMR (564 MHz, $CDCl_3$)

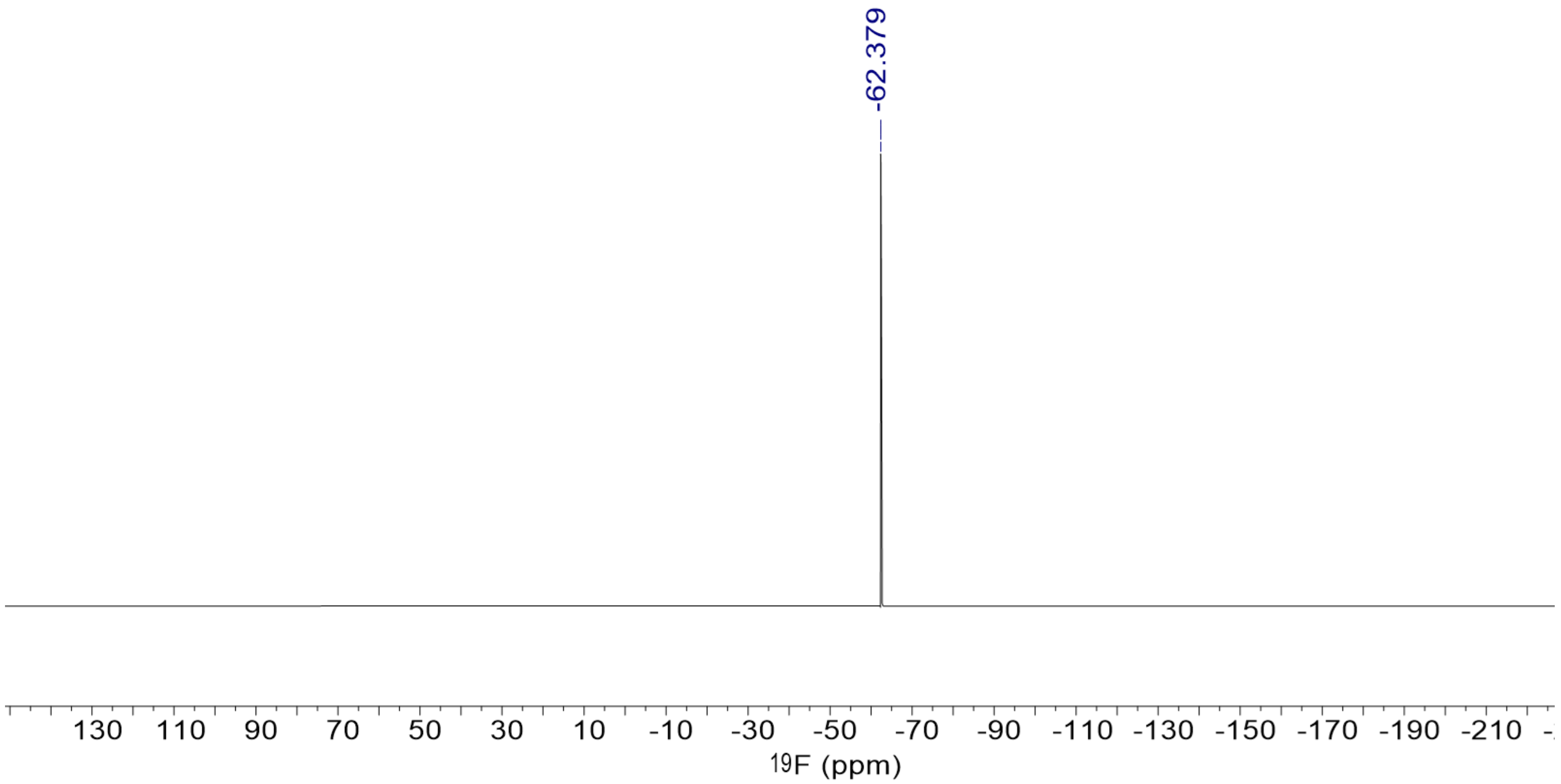

^{1}H NMR (400 MHz, $CDCl_3$)

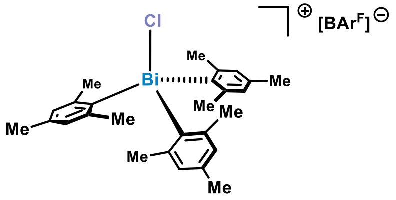

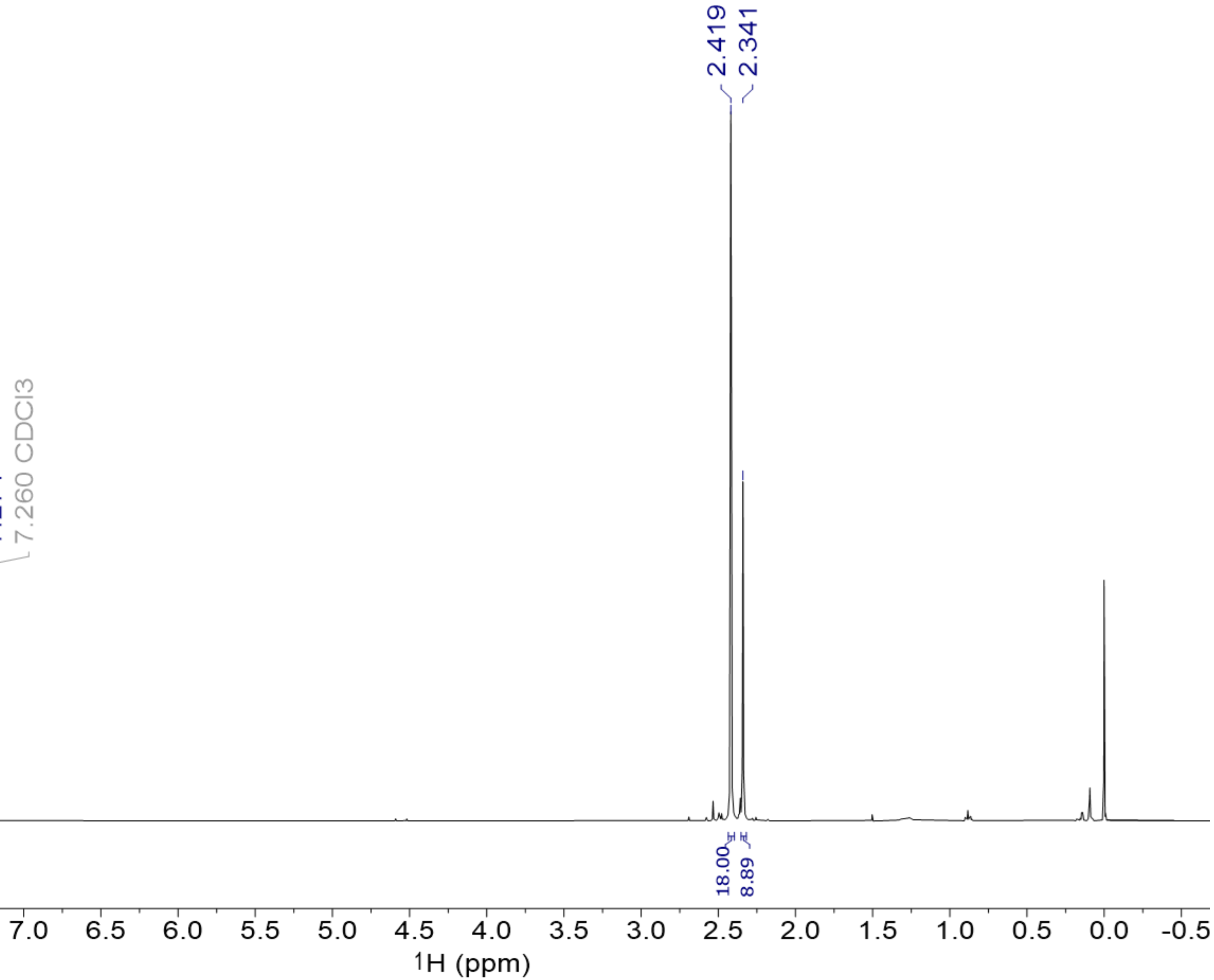

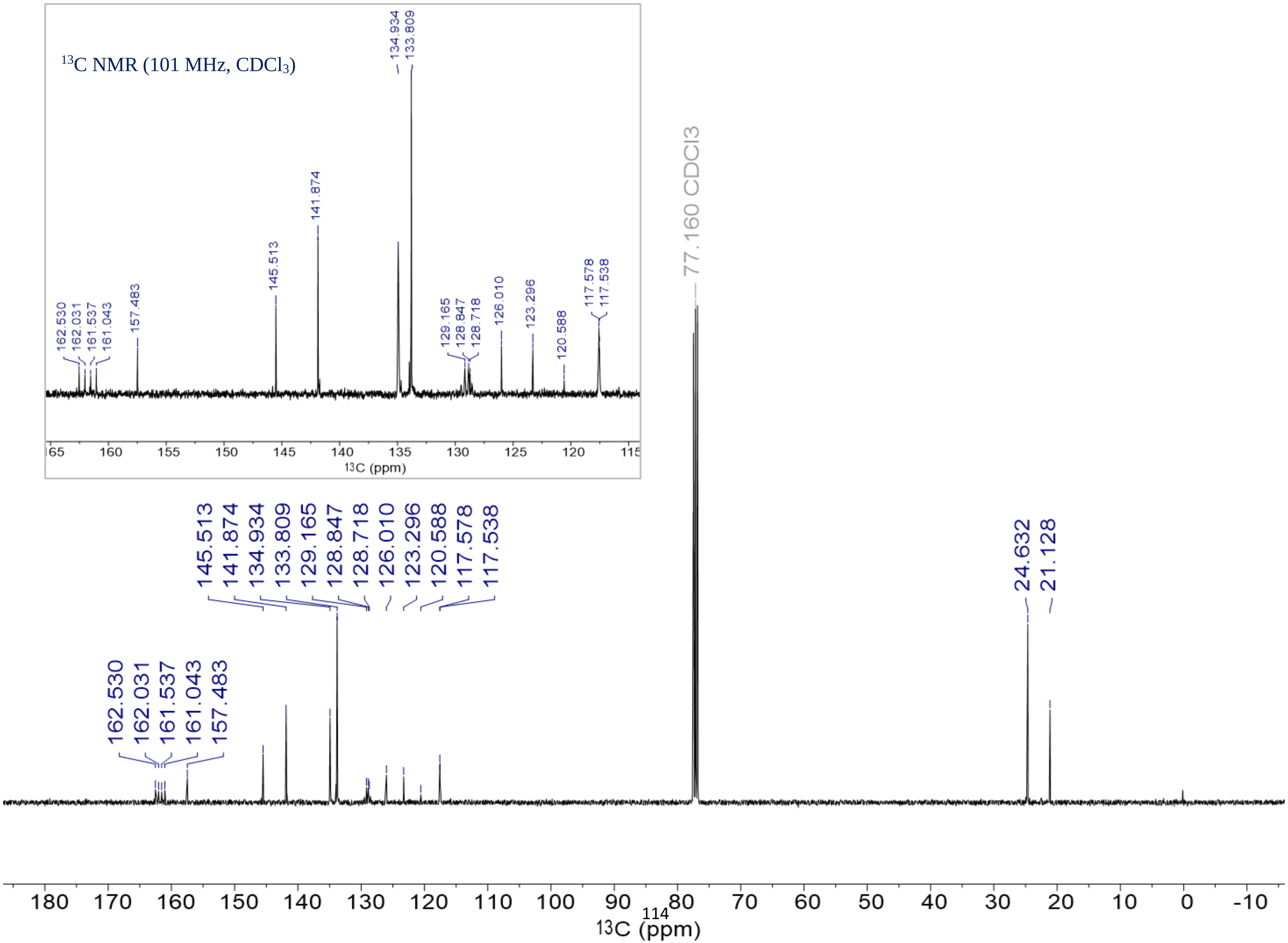

^{13}C NMR (101 MHz, CDCl$_3$)
162.530
162.031
161.537
161.043
157.483
145.513
141.874
134.934
133.809
129.165
128.847
128.718
126.010
123.296
120.588
117.578
117.538
77.160 CDCl3
24.632
21.128
13C (ppm)

^{19}F NMR (282 MHz, $CDCl_3$)

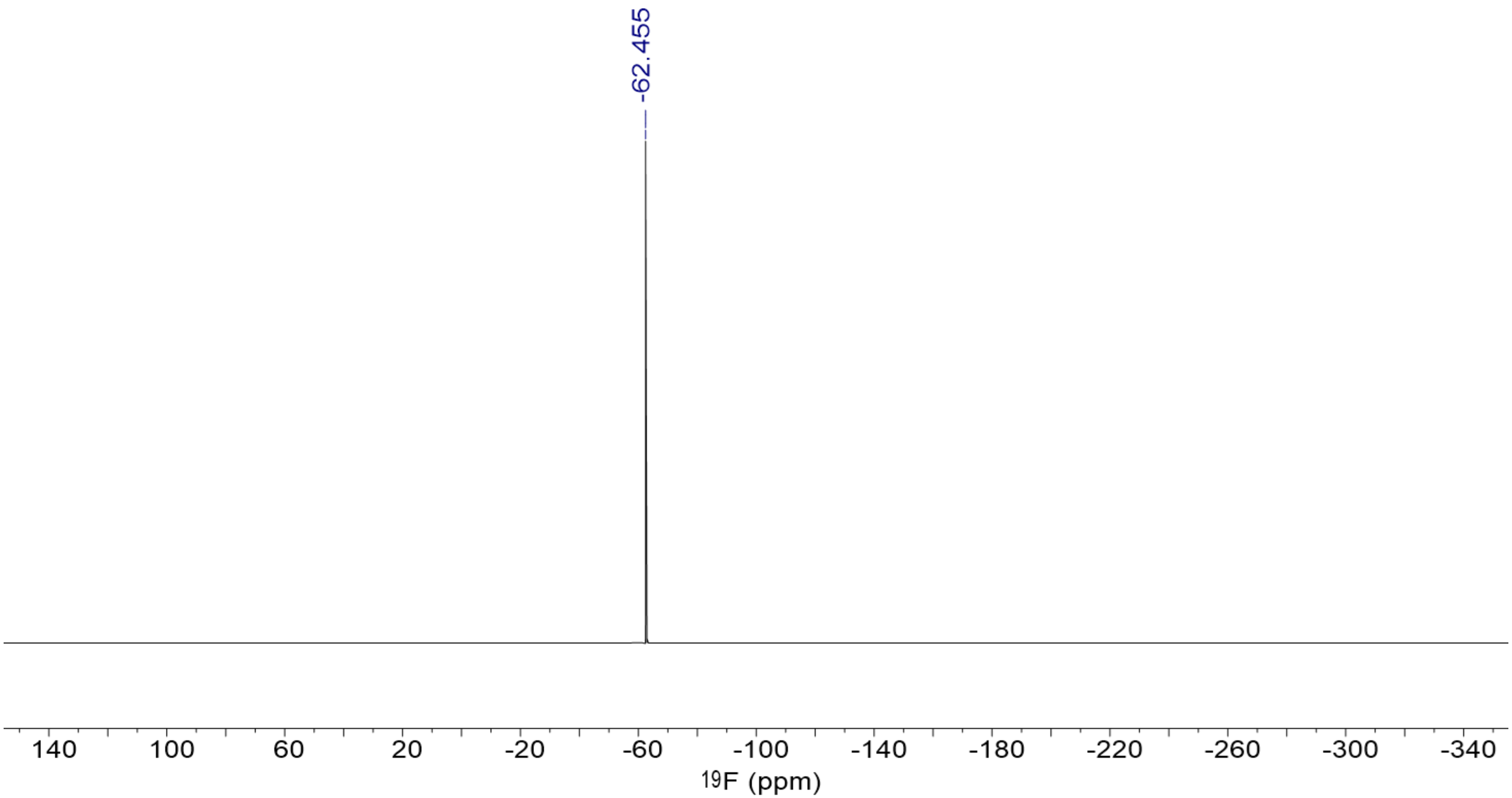

^{1}H NMR (600 MHz, $CDCl_3$)

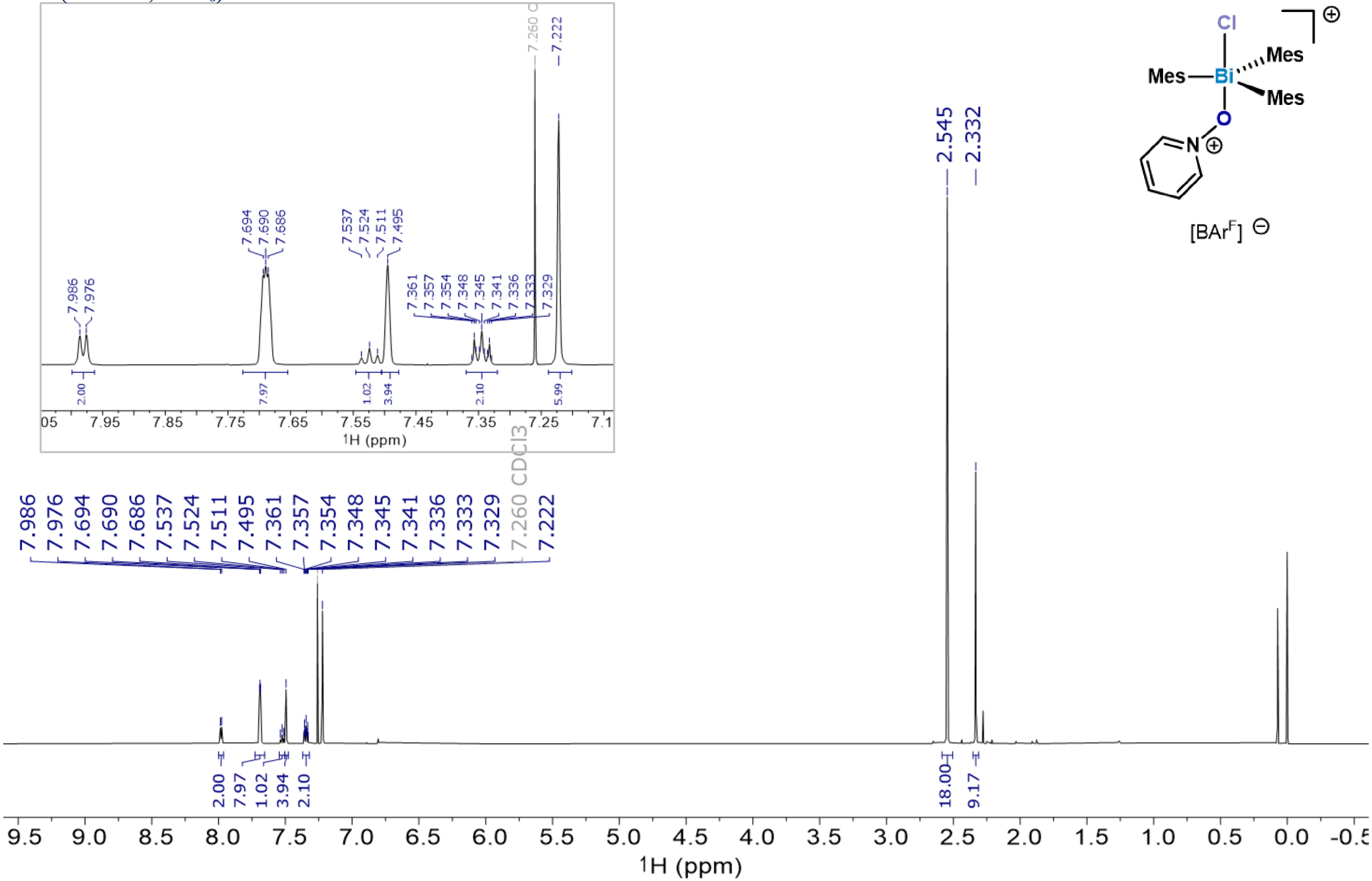

^{13}C NMR (151 MHz, $CDCl_3$)

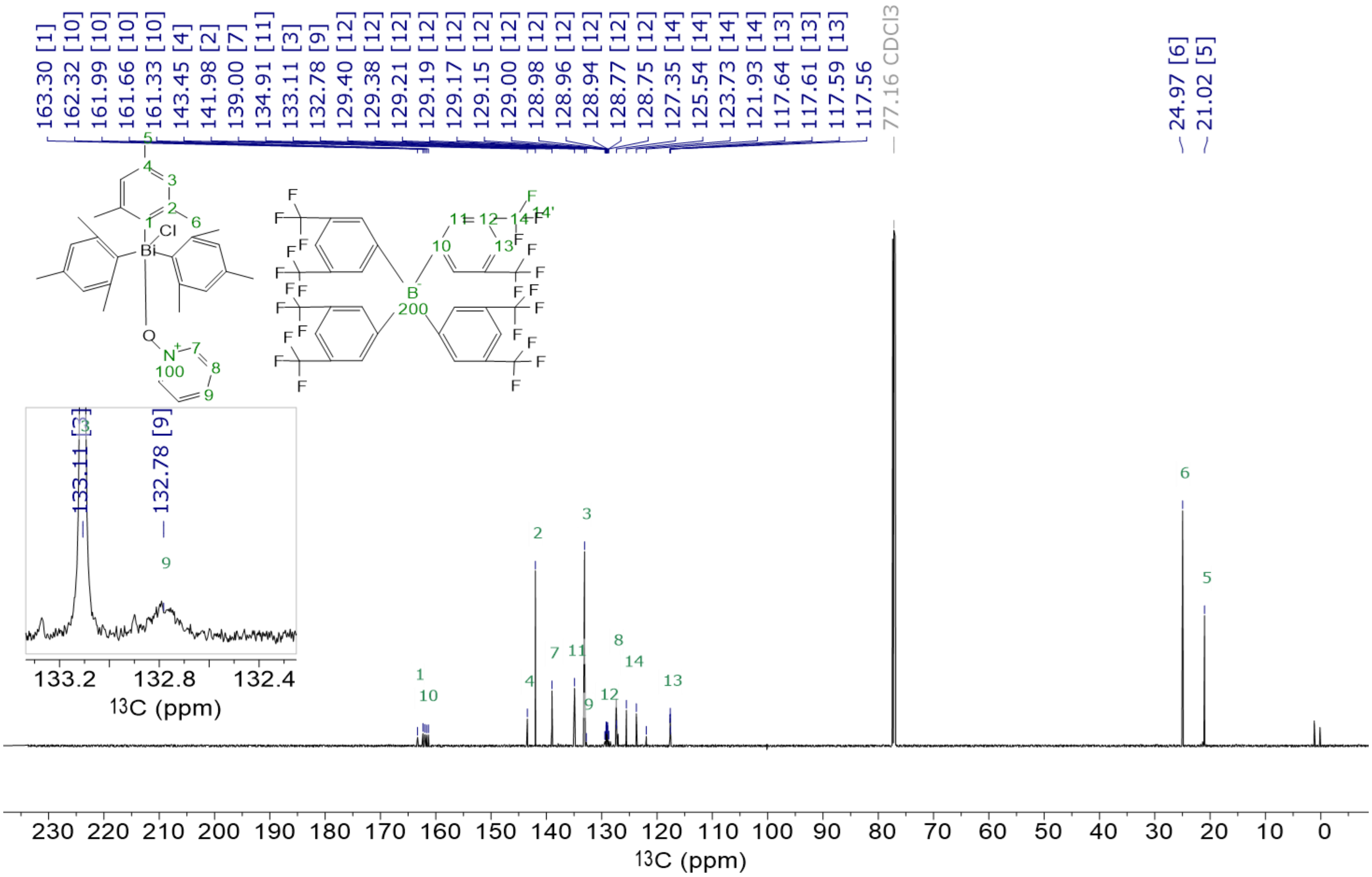

^{1}H-^{1}H NOESY (600 MHz, $CDCl_3$)

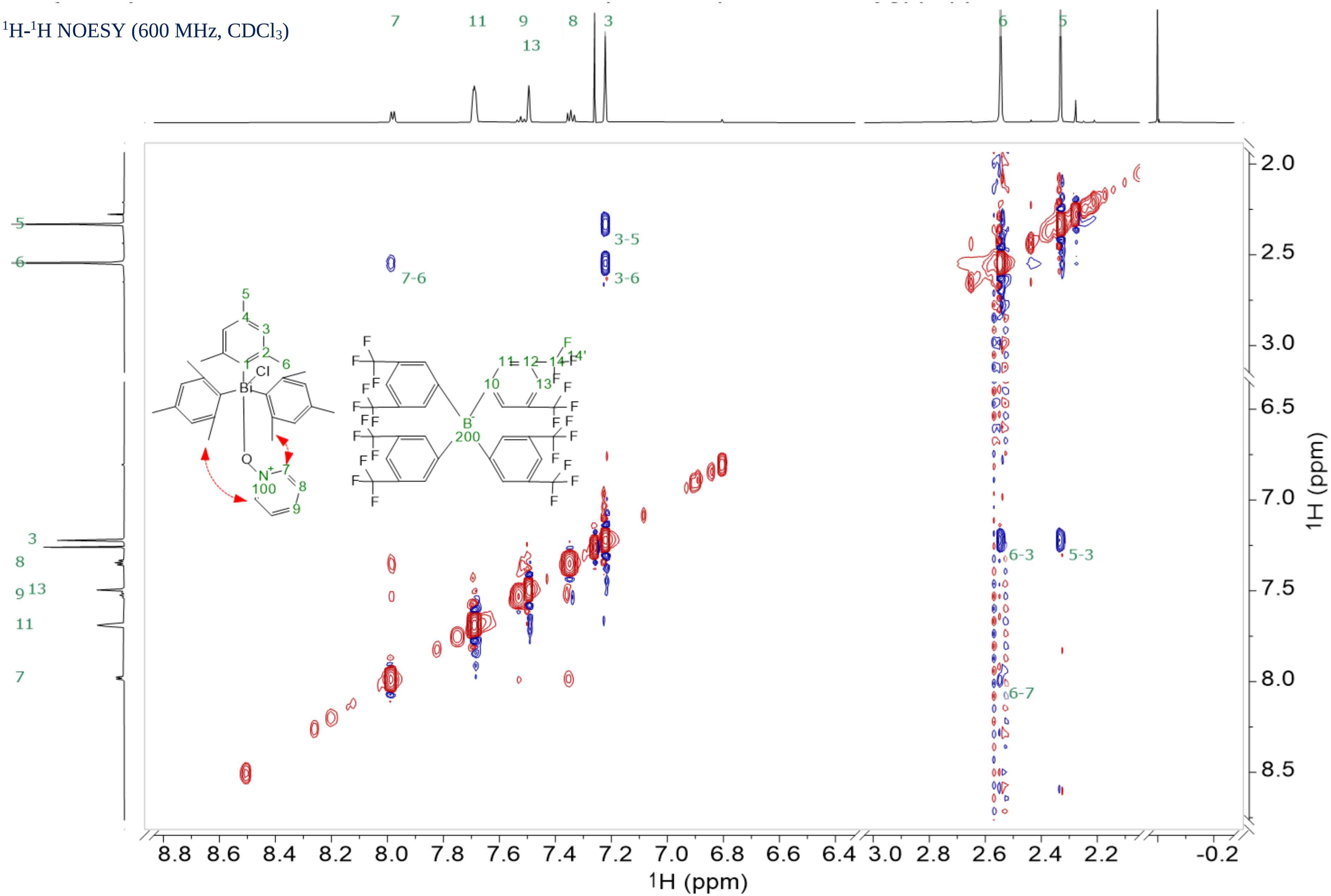

^{1}H NMR (300 MHz, CD_2Cl_2)

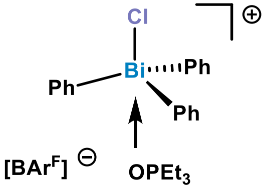

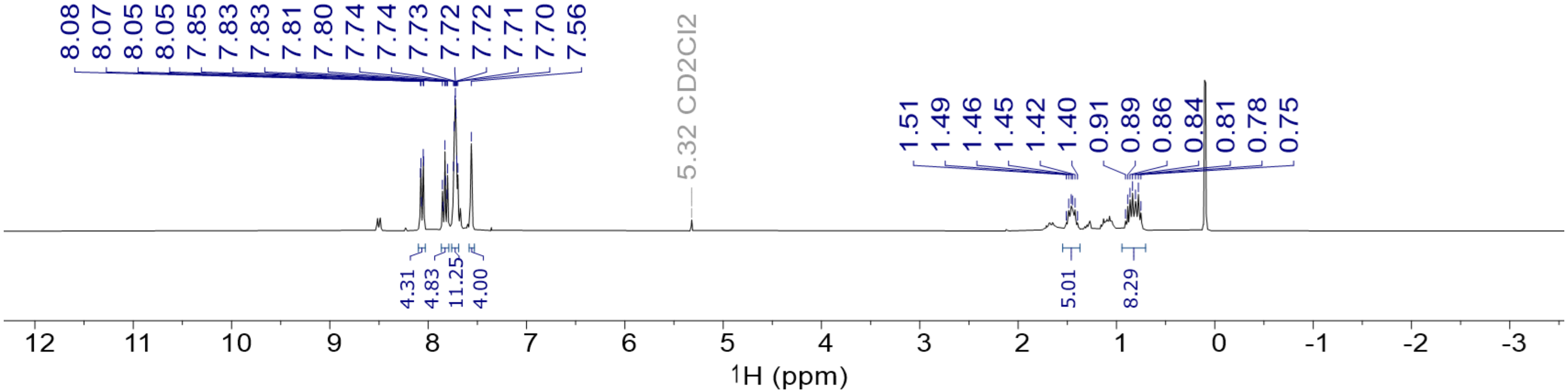

^{31}P NMR (121.5 MHz, CD_2Cl_2)

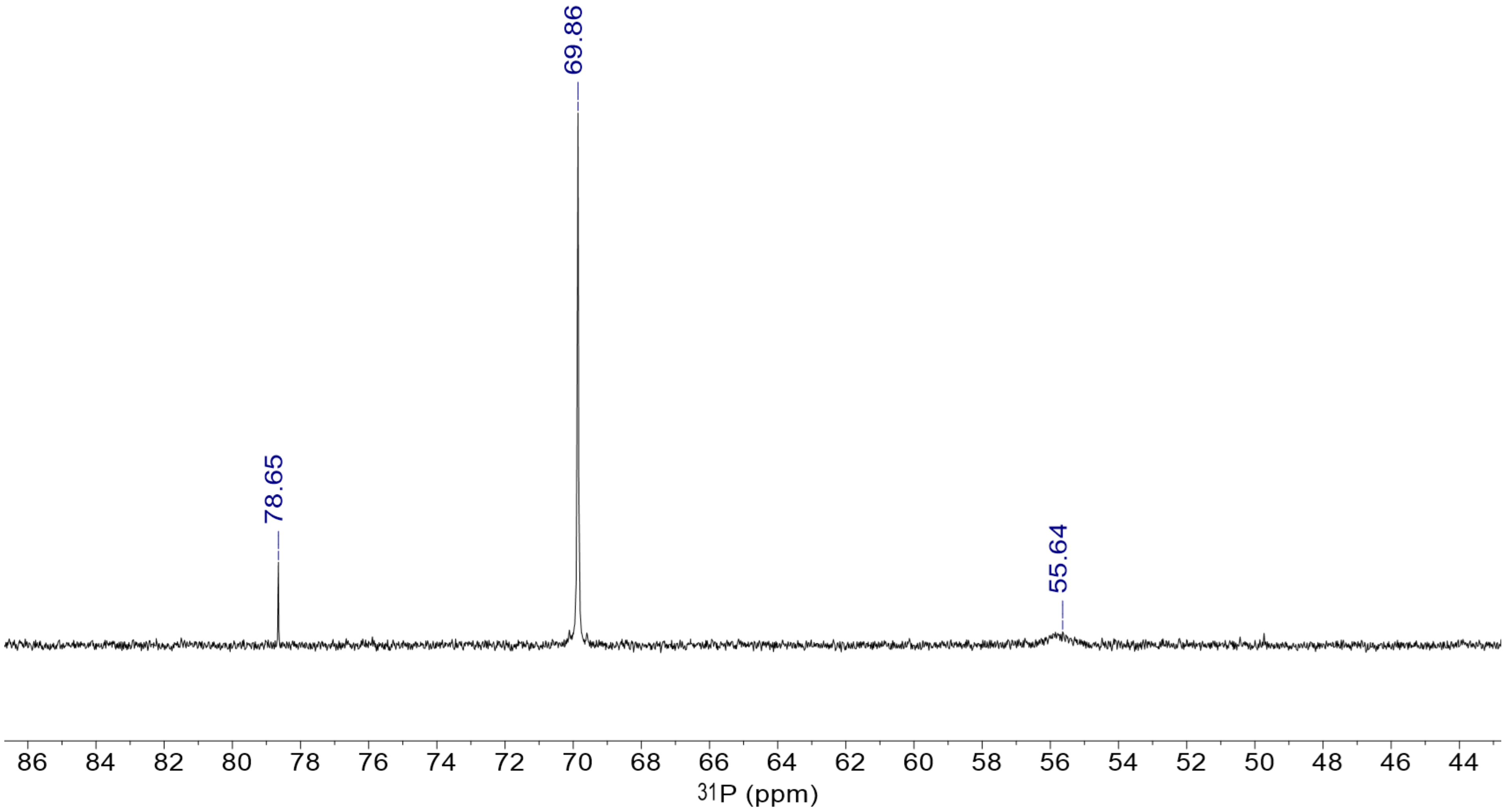

^{1}H NMR (300 MHz, CD_2Cl_2)

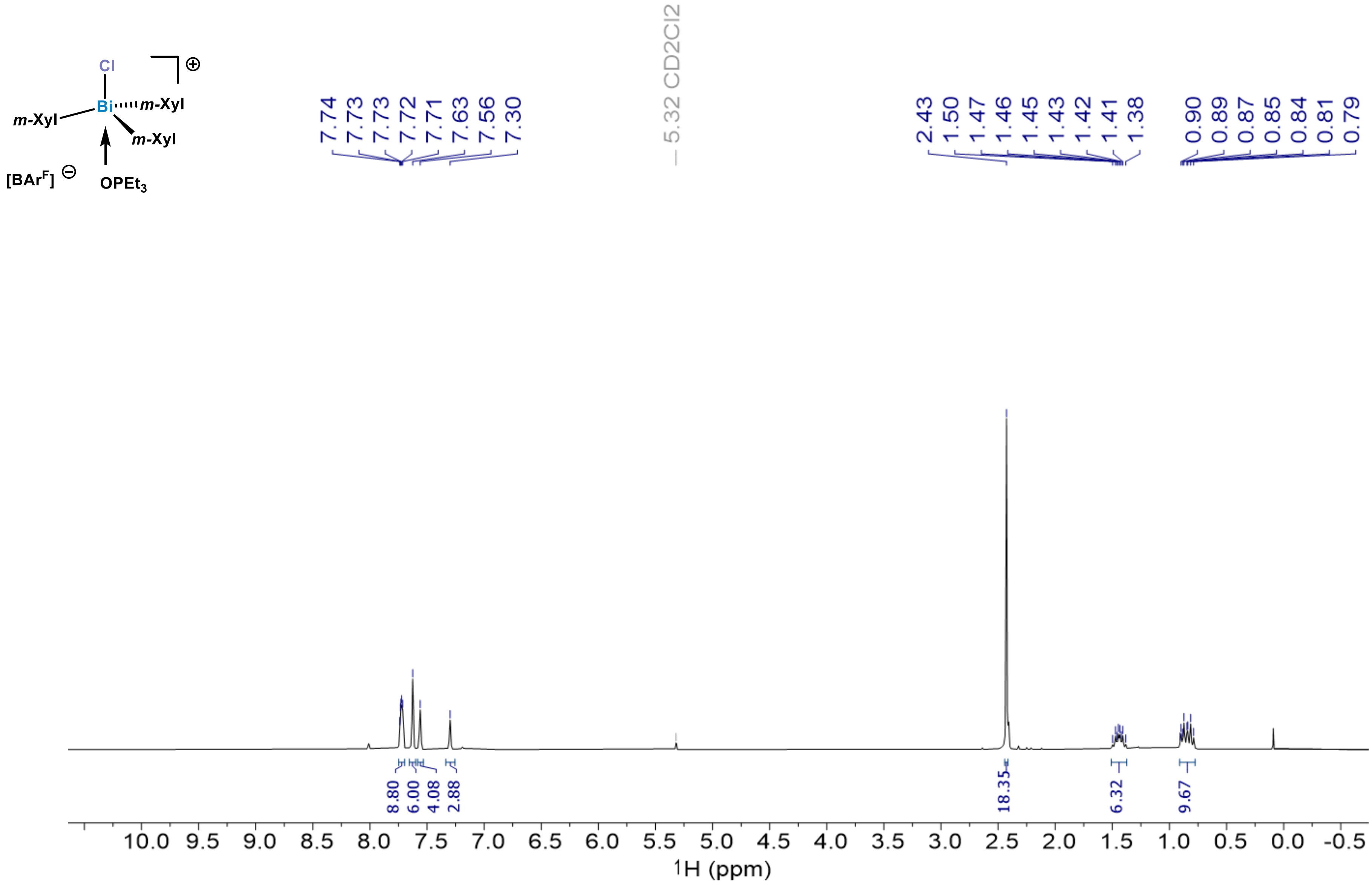

^{31}P NMR (121.5 MHz, CD_2Cl_2)

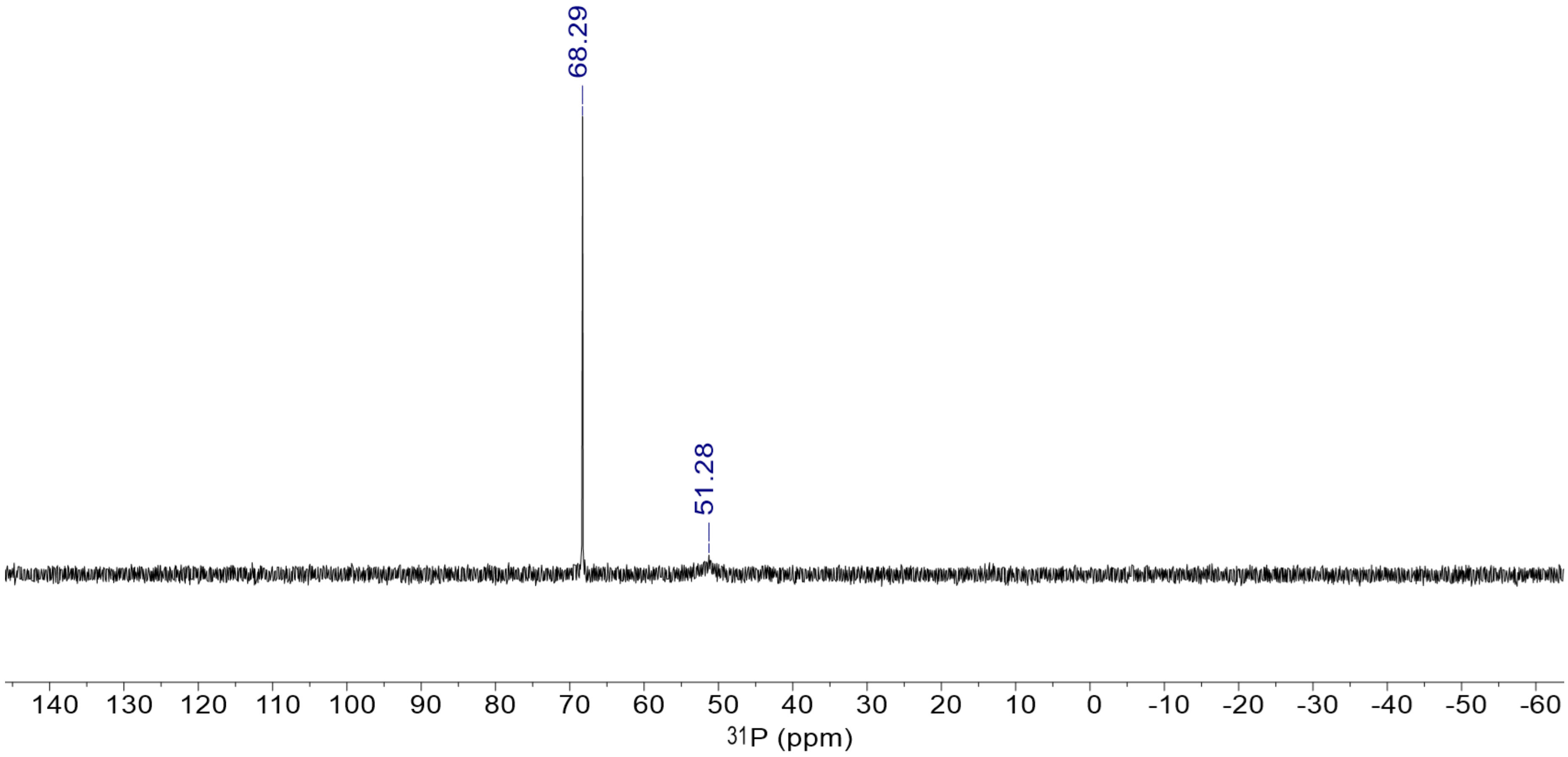

^{1}H NMR (300 MHz, CD_2Cl_2)

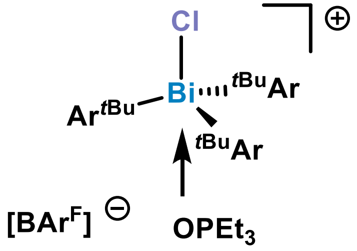

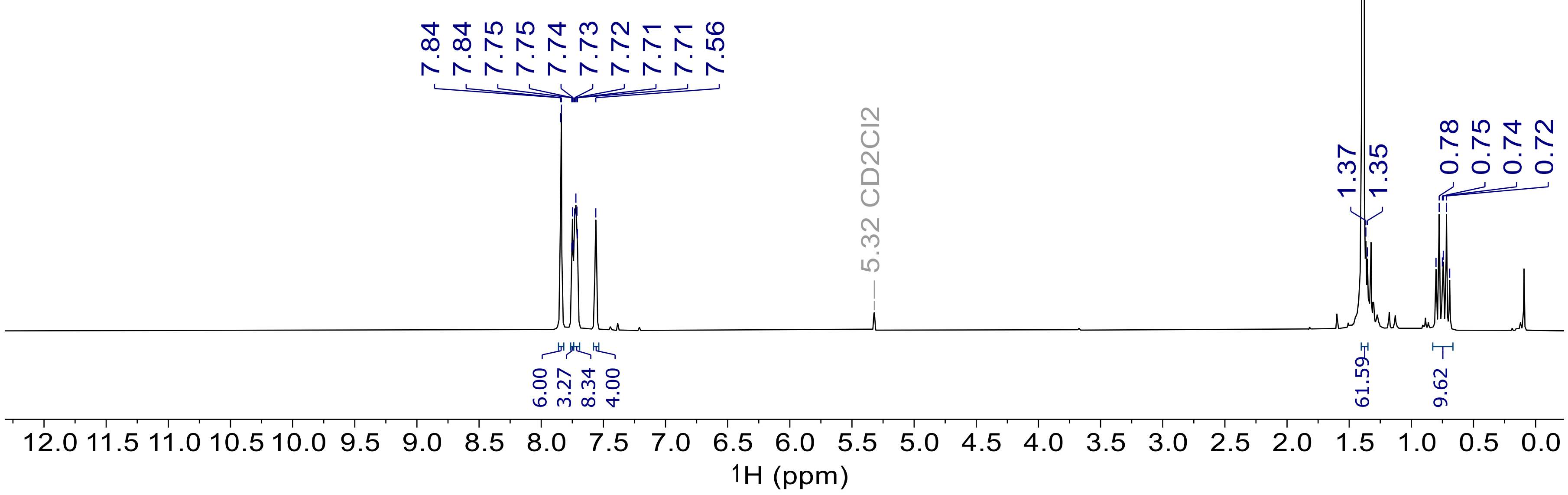

^{31}P NMR (121.5 MHz, CD_2Cl_2)

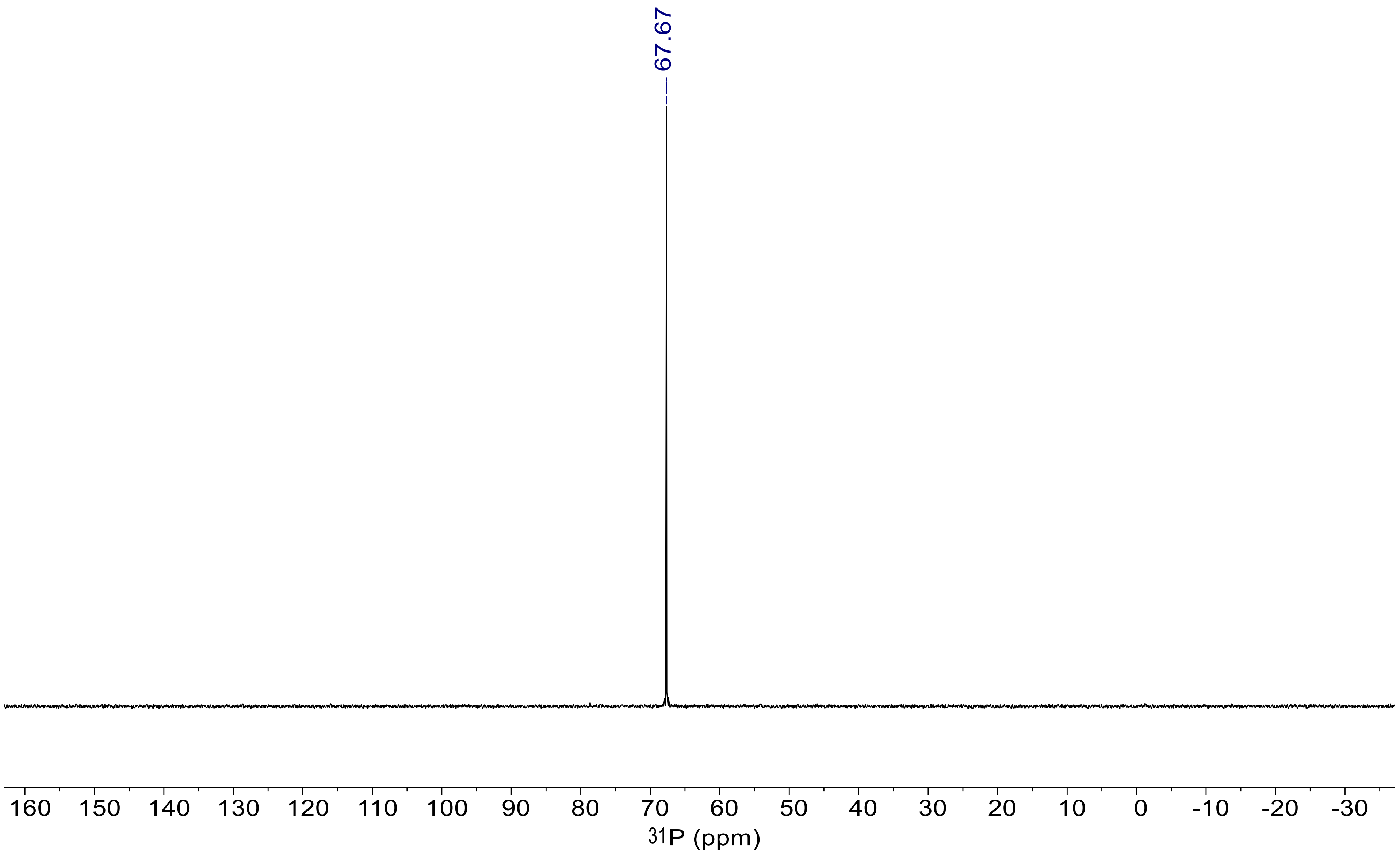

^1H NMR (300 MHz, CD_2Cl_2)

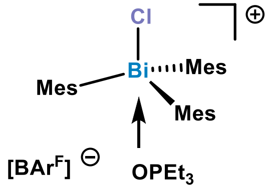

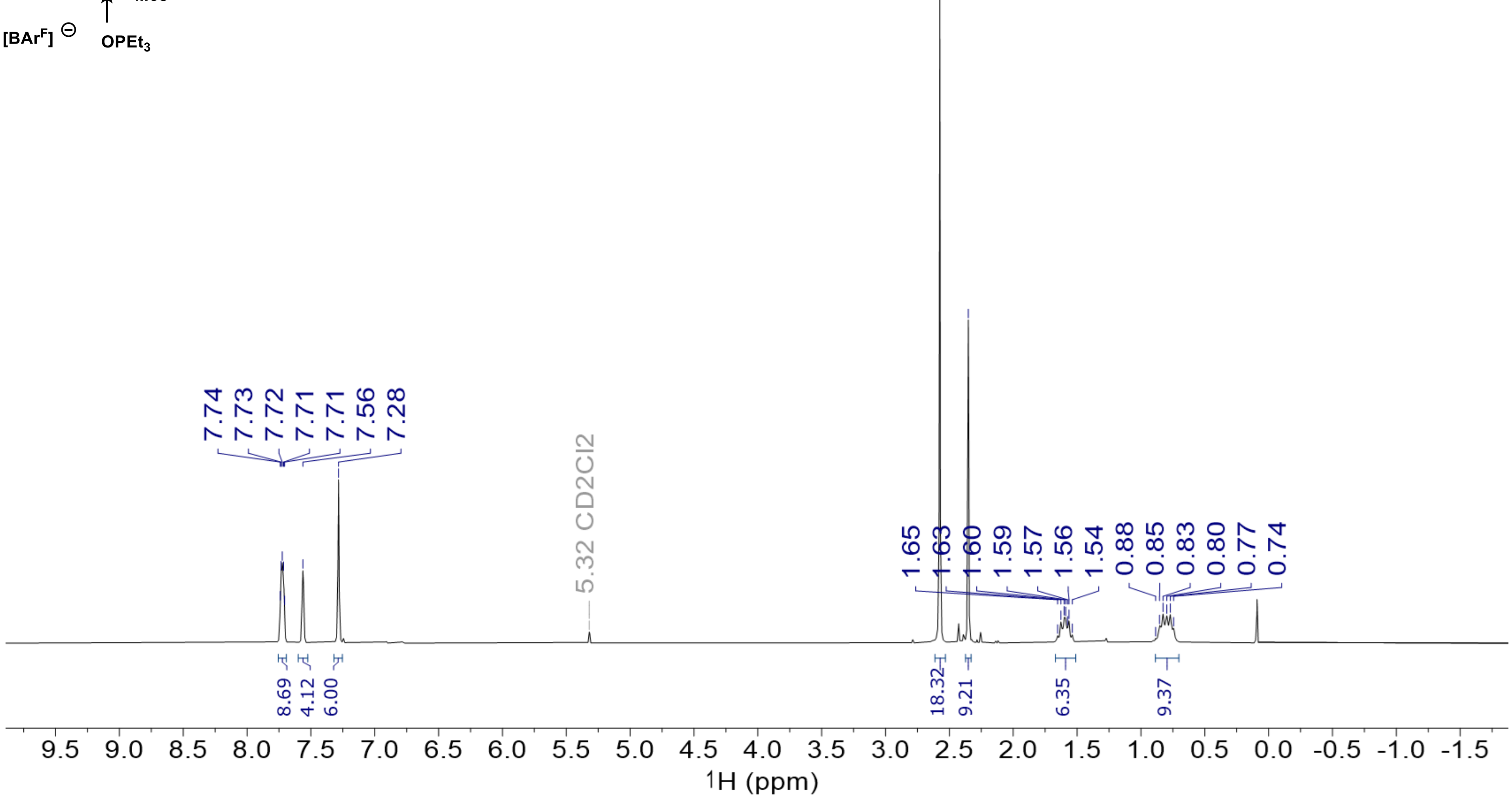

^{31}P NMR (121.5 MHz, CD_2Cl_2)

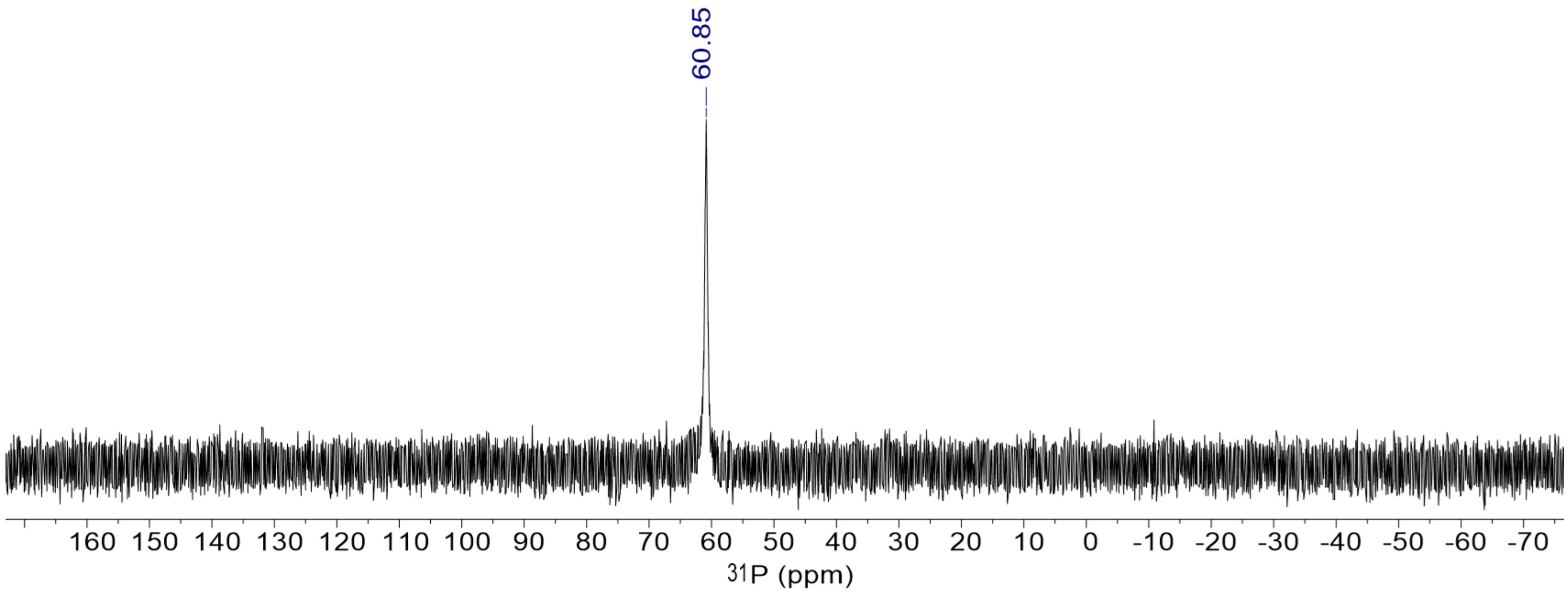

Supplement: CC-060-D4CC03364G-s001 [file CC-060-D4CC03364G-s001.pdf]
